# The serum proteome of Atlantic salmon, *Salmo salar*, during pancreas disease (PD) following infection with salmonid alphavirus subtype 3 (SAV3)^☆^

**DOI:** 10.1016/j.jprot.2013.10.016

**Published:** 2013-12-06

**Authors:** M. Braceland, R. Bickerdike, J. Tinsley, D. Cockerill, M.F. Mcloughlin, D.A. Graham, R.J. Burchmore, W. Weir, C. Wallace, P.D. Eckersall

**Affiliations:** a Institute of Biodiversity, Animal Health and Comparative Medicine, University of Glasgow, Bearsden Rd, Glasgow, G61 1QH, Scotland, UK; b BioMar Ltd., North Shore Road, Grangemouth Docks, Grangemouth, FK3 8UL, Scotland, UK; c Marine Harvest Scotland, Farms Office Blar Mhor Industrial Estate, Fort William, PH33 7PT, Scotland, UK; d Aquatic Vet Services, 35 Cherryvalley Pk, Belfast, BT5 6PN, Northern Ireland, UK; e Fish Diseases Unit, Agri-food and Biosciences Institute, Stoney Rd, Stormont, Belfast, BT4 3SD, Northern Ireland, UK; f Institute of Infection, Immunity & Inflammation, University of Glasgow, Bearsden Rd Glasgow, G61 1QH, Scotland, UK; g VESO Vikan, Aquamedical Contract Research, Vikan, N-7800 Namsos, Norway

**Keywords:** Atlantic salmon, Alpha virus, Pathology, 2-Dimension electrophoresis, Serum proteome, Biomarkers

## Abstract

Salmonid alphavirus is the aetological agent of pancreas disease (PD) in marine Atlantic salmon, *Salmo salar*, and rainbow trout, *Oncorhynchus mykiss*, with most outbreaks in Norway caused by SAV subtype 3 (SAV3). This atypical alphavirus is transmitted horizontally causing a significant economic impact on the aquaculture industry. This histopathological and proteomic study, using an established cohabitational experimental model, investigated the correlation between tissue damage during PD and a number of serum proteins associated with these pathologies in Atlantic salmon. The proteins were identified by two-dimensional electrophoresis, trypsin digest and peptide MS/MS fingerprinting. A number of humoral components of immunity which may act as biomarkers of the disease were also identified. For example, creatine kinase, enolase and malate dehydrogenase serum concentrations were shown to correlate with pathology during PD. In contrast, hemopexin, transferrin, and apolipoprotein, amongst others, altered during later stages of the disease and did not correlate with tissue pathologies. This approach has given new insight into not only PD but also fish disease as a whole, by characterisation of the protein response to infection, through pathological processes to tissue recovery.

**Biological significance:**

Salmonid alphavirus causes pancreas disease (PD) in Atlantic salmon, *Salmo salar*, and has a major economic impact on the aquaculture industry. A proteomic investigation of the change to the serum proteome during PD has been made with an established experimental model of the disease. Serum proteins were identified by two-dimensional electrophoresis, trypsin digest and peptide MS/MS fingerprinting with 72 protein spots being shown to alter significantly over the 12 week period of the infection. The concentrations of certain proteins in serum such as creatine kinase, enolase and malate dehydrogenase were shown to correlate with tissue pathology while other proteins such as hemopexin, transferrin, and apolipoprotein, altered in concentration during later stages of the disease and did not correlate with tissue pathologies. The protein response to infection may be used to monitor disease progression and enhance understanding of the pathology of PD.

## Highlights

• A virological, histological and proteomic study of salmon alpha virus infection
• The serum proteome response to infection determined by two-dimensional electrophoresis
• 72 protein spots show significant change during infection.
• Certain serum protein changes related to tissue pathology using a general linear model
• Other serum protein change related to response to infection

## Graphical abstract

**Figure:**
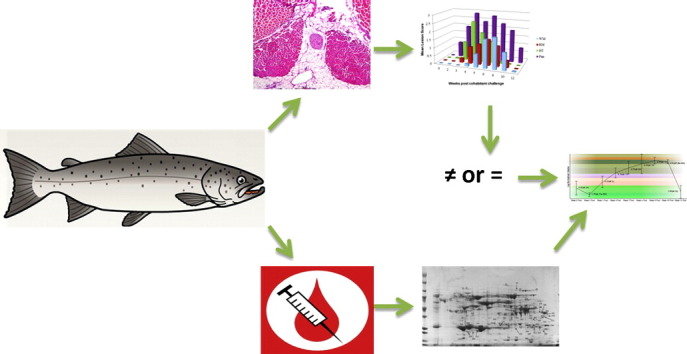

## 1 Introduction

First described in farmed Atlantic salmon, *Salmo salar* L., from Scotland, in 1976, pancreas disease (PD) is characterized by lethargy and other behavioural modifications, sequential acute necrosis of the pancreatic acinar cells, cardiomyopathy and skeletal muscle necrosis, fibrosis and degeneration whilst damage to the kidney, liver and brain can also be observed in some individuals [1–3]. Subsequent to the initial histopathological characterization of PD in Scotland the disease was described in other regions including: North America [4], Norway [5], Ireland [6], France and Spain [7]. It was not until 1995, however, that the aetiological agent of the disease was discovered and given the name salmon pancreas disease virus (SPDV) [8]. Two years later, the aetiological agent of sleeping disease (SD), which shares the same pathogenesis as PD, in freshwater rainbow trout, *Oncorhynchus mykiss*, was also isolated and named sleeping disease virus (SDV) [9]. Further analysis of these viruses revealed that both SDV and SPDV possessed phenotypic and genotypic similarity with serological cross-reactivity also being observed [10]. Thus SDV and SPDV were identified as two related isolates of the same virus and the species name salmonid alphavirus (SAV) proposed.

Knowledge of SAVs has grown considerably since their identification. For instance, there are at present six closely related subtypes defined [11,12]. These differ not only in geographical location [13,14] but also between aquatic environments. For example, SAV 2 is the only subtype commonly detected in freshwater systems, causing SD in freshwater trout, though it has also been identified in salmon in the marine environment [13]. In addition, recent work has suggested that there may be differences between strains in the infection dynamics [15] and minor differences in prevalence and severity of tissue damage [16].

Natural outbreaks of PD in Atlantic salmon have only been reported in the seawater phase of production [3]. In light of this, it would seem that if vertical transmission does occur its impact would be negligible [17], with horizontal transmission being by far the most important means of virus spread [18,19] with shedding of mucus and faeces being recently described as transmission routes for SAV [15].

Proteomics is a well-established post-genomic tool which allows investigation of complex biological systems involved in pathology and physiology in model organisms and livestock, such as; fish, cattle and pigs [20–22]. However, despite some notable exceptions, aquaculture research using this methodology has had limited application in fish biology [23]. Furthermore, there have been fewer attempts to determine the relationship between pathology and proteome. Therefore, this study aims to investigate the modification of the Atlantic salmon serum proteome profile caused by PD, using SAV3 as the aetiological agent, in order to identify serum biomarkers of the disease in relation to tissue damage as assessed by histopathology. In addition, this study explores serum proteome changes over a 12 week period to highlight biomarkers of the most critical period of pathology and disease recovery. Virology, serology and RT-PCR were used to experimentally demonstrate the horizontal spread of SAV and to confirm the virus was the aetiological agent of observed pathology.

## 2 Materials and methods

### 2.1 Fish husbandry and challenge

The following experimental procedure was approved by the Norwegian National Animal Research Authority (NARA) prior to the trial commencing.

Seven hundred Atlantic salmon (*Salmo salar*) parr of mean weight 30 g, (< 15% CV) were randomly distributed into duplicate 1 m^3^ tanks (= 1400 total fish). Following six week acclimatisation the fish were fed to a target of 1.5% body weight per day. Commercial formulated feed was offered to the fish throughout the experiment (CPK 2 mm; 3 mm, BioMar AS, Denmark). Water temperature was maintained at 12–14 ± 1 °C, water flow 0.8 l/kg min, and light/dark regime 12:12 h. After 42 days, 60 fish from each duplicate tank were transferred into triplicate 0.6 m^3^ tanks and water temperature was increased to 14 ± 1 °C. Additional fish from the duplicate tanks were maintained separately to be used as Trojan shedders i.e. twelve challenge tanks all containing 120 fish (with Trojan fish to be added later). Naïve fish to be used as Trojans were marked by clipping their adipose fin and injected with SAV 3 infected CHSE cell culture supernatant at ca. 10^5^ TCID/fish into their intraperitoneal cavity. Thirty inoculated Trojans were added to each of the challenge tanks 6 days after their assembly. The challenge and time course of sampling were staggered between replicate tanks over three consecutive days and kept constant at each sampling. Cohabitant fish were sampled at 0, 2, 3, 4, 5, 6, 8, 10 and 12 weeks post challenge (wpc). At each time point 9 fish per tank were killed by lethal overdose of anaesthetic benzocaine chloride (Apotekproduksjon AS, Oslo, Norway), 1 g/10 L water for 5 min being used and blood collected in non-heparinised vacutainers for analysis of serum biochemistry and serological and virological analysis. From 6 of these fish, pyloric caecae and pancreas (hereafter referred to as pancreas), heart and skeletal muscle tissue were processed from standardised locations for histology. Fish sampled at time point 0 were removed from the tanks before the addition of Trojan shedders.

### 2.2 Virological, serological and RT-PCR testing

Assessment of virology was carried out in order to confirm that SAV was absent at the start of the trial and subsequently to confirm that any changes in histopathology and the serum proteome were caused by SAV infection and thus PD. Virus neutralization (VN) testing, virus isolation and real-time RT-PCR testing were carried out as previously described [15].

### 2.3 Histopathology

Tissues for histology from the salmon were immediately fixed in 3.5% v/v formaldehyde in buffered saline pH 7.0 (4.0 g NaH_2_PO_4._2H_2_O, 6.5 g Na_2_HPO_4._2H_2_O) prior to further processing by standard paraffin wax techniques, sectioned and stained with haematoxylin and eosin (H & E). The tissue sections were examined by an experienced pathologist and a scoring system was used to semi-quantify the distribution and severity of the tissue lesions in the pancreas, heart and skeletal muscle as used in previous studies [16,24]. A mean score was calculated at each time point for tissues to firstly demonstrate the pathological damage caused by SAV infection/PD and also to examine the relationship between tissue damage and the serum proteome (see Section 2.5 for further information).

### 2.4 Sample preparation and two-dimensional electrophoresis (2DE)

One microlitre of each serum sample collected from each fish sampled at each time point was pooled according to week to create pooled samples for the analysis of changing protein composition throughout the time course. The protein concentration of the pooled samples was determined by Bradford assay, using Bradford Reagent (Sigma-Aldrich, Poole UK), in accordance with the manufacturer's protocol. Concentrations were used to allow dilution of samples to an equal protein loading (of 208 μg) for 2DE protein separation by isoelectric focusing based on isoelectric point (pI) and sodium dodecyl sulphate polyacrylamide gel electrophoresis (SDS-PAGE) based on molecular weight (Mw). Three replicate 2DE gels were run of the pool of samples from each time point. Separation by pI was carried out using 11 cm immobilized pH Gradient (IPG) strip with a pH range of 3 to 10 (BioRad, Hemel Hempstead, UK). After protein loading of the IPG strips, with serum diluted in a rehydration buffer (8 M Urea, 2% CHAPS, 50 mM DTT, 0.2% Bio-Lyte®) (BioRad, Hemel Hempstead, UK) and covered in 500 μl of mineral oil, a combined rehydration and focusing step was carried out over 17 h with a total of 35,000 V-h. The IPG strips were removed, oil drained and then treated with two equilibration buffers both made from a stock solution comprised of 6 M urea, 0.375 M Tris–HCl, pH 8.8, 2% (w/v) SDS, 20% (v/v) glycerol, the first of these containing 2% (w/v) dithiothreitol (Sigma-Aldrich, Poole, UK) to reduce the proteins and subsequently the alkylating agent iodoacetamide at 2.5% (w/v) (Sigma-Aldrich, Poole, UK). IPG strips were then placed onto Criterion SDS-PAGE gels and submerged in XT Mops running buffer and subjected to electrophoresis at 200 V for one hour (Bio-Rad, Hemel Hempstead UK). Subsequently gels were stained in Coomassie brilliant blue G-250 dye 0.1% (w/v) in de-stain solution for 1 h and then de-stained using a solution of methanol:water:acetic acid, (4:5:1) overnight, scanned and saved in 16-bit grey TIFF format images for gel image analysis.

### 2.5 Gel image analysis

Images were uploaded onto ‘Nonlinear Progenesis SameSpots’ 2D gel image analysis software (Nonlinear Dynamics, Newcastle, UK) which was used to identify protein spots which were differentially expressed through time (inferred by the programme by normalised spot intensities). Initial results were filtered using the programme's statistical analysis function, with only those with a power value of > 80% and ANOVA significance score of < 0.05 between groups of replicate gels, being chosen for protein identification. To investigate the relationship among different time-points and different proteins with respect to spot intensity, the dataset was analysed using cluster analysis. ArrayStar software (DNASTAR, Madison, WI, USA) was used to perform Hierarchical Cluster Analysis based on Euclidean distance and the results were illustrated in the form of a heat map.

Spot information (profiles) was also used in a general linear model procedure in SAS version 9.3 (SAS Institute, Cary, N. Carolina) for regression analysis. Each spot was regressed on the mean value of each tissue's histopathological score at each sampling time point in a separate model. Therefore, the probability that a protein spot increased or decreased in intensity in association with tissue damage as determined by histopathology was determined.

### 2.6 Spot preparation and mass-spectrometry

Chosen protein spots were excised manually by scalpel and placed in individual vials to be subjected to in-gel digestion for protein extraction prior to identification via mass spectrometry analysis. Gel pieces were washed with 100 mM NH_4_HCO_3_ for 30 min and then for a further hour with 100 mM NH_4_HCO_3_ in 50% (v/v) acetonitrile. After each wash all solvent was discarded. Gel plugs were then dehydrated with 100% acetonitrile for 10 min prior to solvent being removed and dried completely by vacuum centrifugation. Dry gel pieces were then rehydrated with 10 μl trypsin at a concentration of 20 ng/μl in 25 mM NH_4_HCO_3_ (Cat No. V5111, Promega, Madison, WI, USA) and proteins allowed to digest overnight at 37 °C. This liquid was transferred to a fresh tube, and gel pieces washed for 10 min with 10 μl of 50% acetonitrile. This wash was pooled with the first extract, and the tryptic peptides dried to completion. Tryptic peptides were solubilized in 0.5% (v/v) formic acid and fractionated on a nanoflow uHPLC system (Thermo RSLCnano) before analysis by electrospray ionisation (ESI) mass spectrometry on an Amazon ion trap MS/MS (Bruker Daltonics). Peptide separation was performed on a Pepmap C18 reversed phase column (LC Packings), using a 5–85% v/v acetonitrile gradient (in 0.5% v/v formic acid) run over 45 min. at a flow rate of 0.2μl/min. Mass spectrometric (MS) analysis was performed using a continuous duty cycle of survey MS scan followed by up to five MS/MS analyses of the most abundant peptides, choosing the most intense multiply-charged ions with dynamic exclusion for 120 s. MS data were processed using Data Analysis software (Bruker) and the automated Matrix Science Mascot Daemon server (v2.1.06). Protein identifications were assigned using the Mascot search engine to interrogate protein sequences in the NCBI databases restricting the search to teleostei, allowing a mass tolerance of 0.4 Da for both MS and MS/MS analyses. In addition, the search consisted of a carbamidomethyl fixed modification and a variable oxidation.

## 3 Results

### 3.1 Virology, serology and RT-PCR

Cohabitant fish were SAV free before introduction of Trojan shedders, as determined by virus isolation, virus neutralization and RT-PCR (Table 1). Post introduction there was a subsequent horizontal spread of the viral infection with the majority of sampled fish being infected by week 4.

### 3.2 Histopathology of pancreas disease

**Fig. 1:**
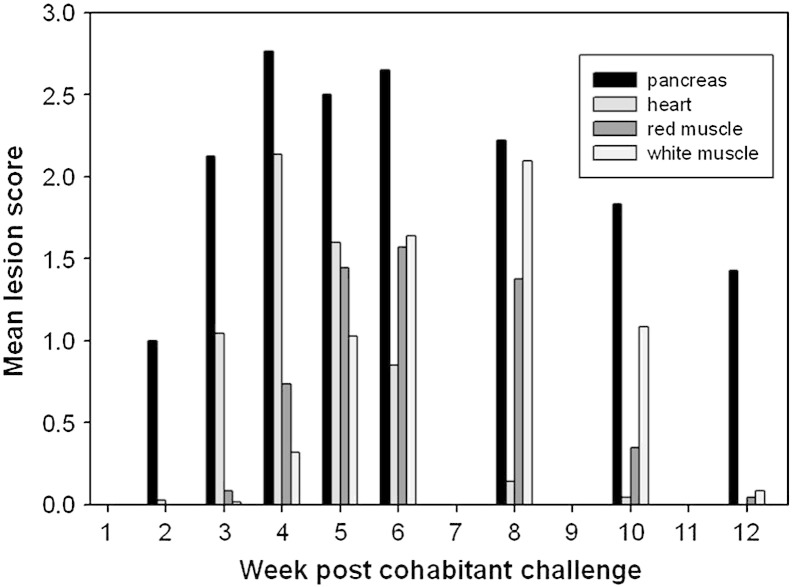
Histopathological scoring of mean lesion scores in relation to week post challenge with SAV in pancreas (Pan), heart (HT), red muscle (RM) and white muscle (WM) of salmon (n = 9 per time point).

Negligible mortality was observed pre-and post trial. The development of lesions over time is illustrated in Fig. 1, which shows mean lesion scores for each tissue at each sampling point. The pancreas was the first tissue to develop lesions at week 2 and was also the slowest to recover, with a minority of samples still not fully recovered by week 12. Conversely, the heart demonstrates an extremely quick recovery, with a peak in lesion severity in fish sampled in week 4 and then a rapid recovery. Since this study was based upon a cohabitation model individuals were likely to be at different stages in the disease process at each sampling point due to variation in the time of infection. The histopathological damage to red and white muscle was more delayed with the peak damage occurring at 6 and 8 weeks respectively.

### 3.3 Profiling changes in the serum proteome

**Fig. 2:**
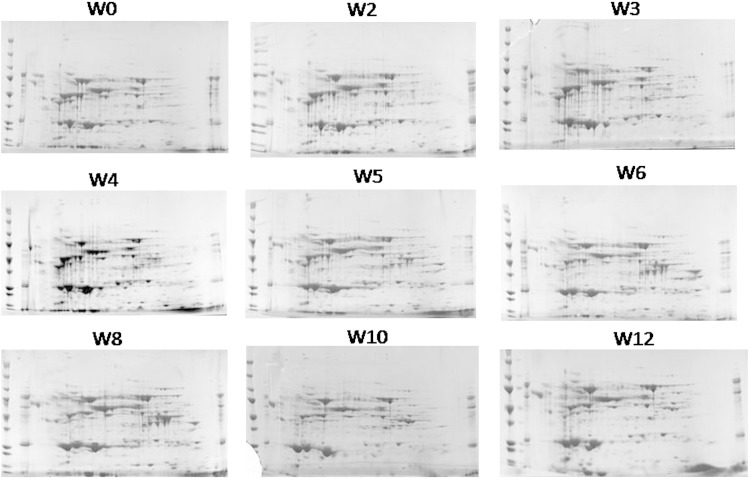
Sample images of 2-dimension electrophoresis gels from all sampling time points from week 0 to 12 post challenge. From left to right: Top row = Week 0, 2 and 3 images. Middle row = Week 4, 5 and 6 images. Bottom row = Week 8, 10 and 12 images.

**Fig. 3:**
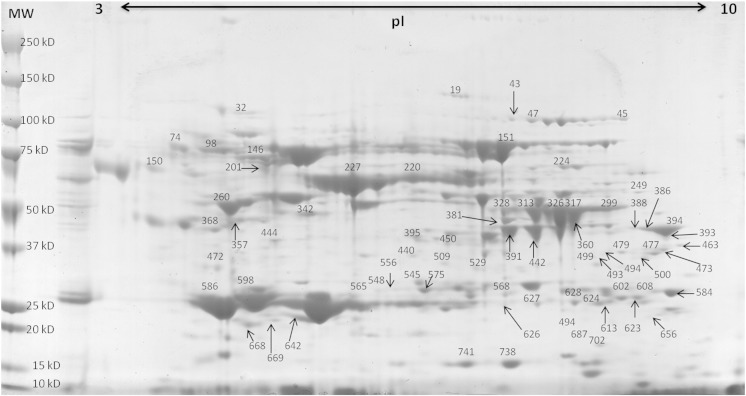
Protein spots identified on 2-dimension electrophoresis as being as being differentially expressed following infection superimposed on a week 0 image. Molecular weight (MW) is indicated on protein standard ladder in kilodalton (kD) and isoelectric point (pI) range also shown.

**Fig. 4:**
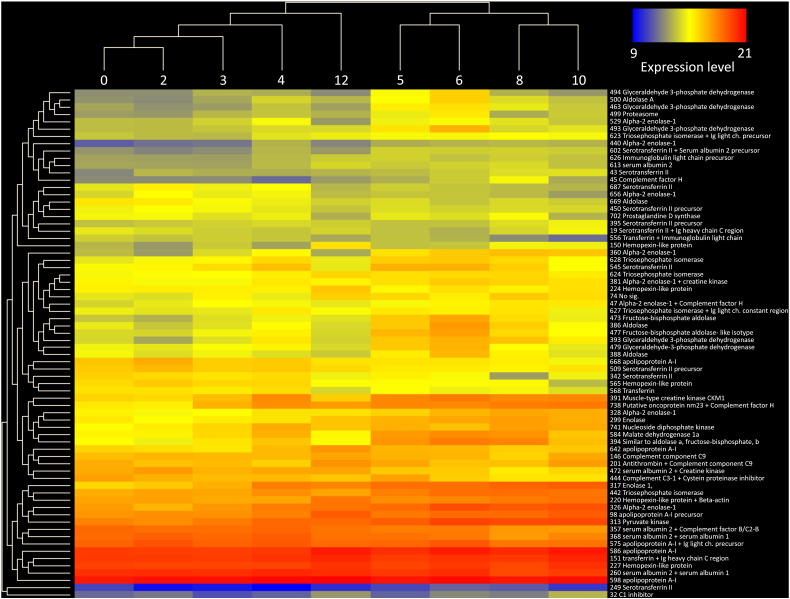
Arraystar heat map representing the results of Hierarchical Clustering of spot intensities. The relationships among different sampling time-points are illustrated as a dendrogram at the top of the diagram. The right-hand side gives spot number and corresponding identity from MS/MS which are grouped by profile similarity with a dendrogram showing the relationships on left-hand side. The heat map is on a colour scale where low protein abundance is represented in blue and high abundance in red.

There were a number of clearly visible differences in the serum proteome over the 12 week period of the trial, illustrated in Fig. 2 where sample gels from each of the nine sampling time points can be seen. However, to quantify and identify changes, scanned 2D-PAGE gel images from each sampling time point were compared using ‘SameSpots’ software to identify protein spots which were differentially expressed in the serum as a result of PD. In total, 894 spots were identified by the SameSpot software of which 72 spots were found to differ significantly over the course of infection (Fig. 3). These were excised for peptide mass fingerprinting from the gel where they showed greatest intensity. Protein identification following DEAMON/MASCOT searching is given in Table 2. Spot intensities at each time-point were analysed using Hierarchical Cluster Analysis to more clearly identify whether an association with disease progression over time could be identified (Fig. 1) and to group proteins which possessed similar expression profiles (Fig. 4). Fig. 4 also lists spot numbers and their corresponding identities obtained by ion trap mass spectrometry analysis of excised spots.

The dendrogram at the top of Fig. 4 shows the relationship of the spot intensities at the nine time points illustrating progression of the infection. There was a clear separation between those recorded from 0 to 4 wpc and those recorded from 5 wpc onwards. Comparison of the proteome at 12 wpc with those prior to 4 wpc indicated a return to homeostasis. The overall fold increase or decrease in spot intensities from basal level (week 0) for upregulated and downregulated proteins respectively is shown in Table 2. Whilst most proteins and enzymes increased in their abundance there were also a number of proteins that declined in abundance (e.g. albumin).

### 3.4 The relationship between tissue pathology and the serum proteome

The relationship between disease pathology and protein abundance, given by spot intensities, was examined by multilinear regression general linear model (GLM) analysis of mean pathological scoring and all mean spot intensities at each sampling time point. Table 3, which lists spot numbers and their corresponding protein identity, also indicates the probability (Pr > F) that a given spot expression profile is linked with a particular tissue pathology, with values < 0.05 being regarded as significant.

**Fig. 5:**
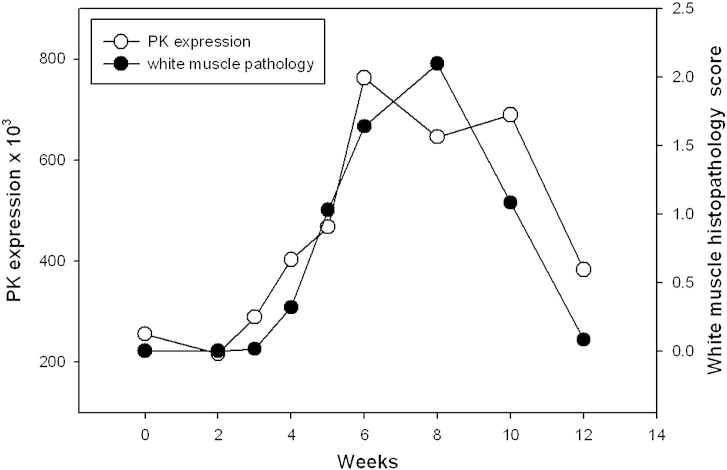
Plotting spot 313 (pyruvate kinase) mean intensity and mean histopathological scoring of white muscle against sampling time points. Open circles plot histopathology scores and filled circles plot spot intensity at a given time point.

In addition, expression profiles were plotted on a graph for each protein (see Supplementary Information) against the mean pathology pattern of each tissue sampled. Fig. 5 illustrates the relationship between mean white muscle histopathology results and the spot intensity of protein spot 313, identified as pyruvate kinase between weeks 2 and 12. Week 0 was removed from this analysis as white muscle showed no lesions at this time point. Graphs for other proteins are given as extra material.

The alterations in serum proteins as a result of PD fell into either of two categories. The first of these were proteins which demonstrated a change in serum abundance (spot intensity) that was associated with damage to a particular tissue or tissues where an increase in intensity was significantly related to the damage. In contrast there were proteins for which the abundance change was not associated directly with tissue damage and were likely to be present in serum as humoral components of host defence (Table 3). Among the group of proteins, the concentration of which was associated with tissue pathology, were a number of enzymes described by ontology as being involved in intracellular pathways. These include creatine kinase, enolases, triosephosphate isomerase, and malate dehydrogenase 1a. The second group of proteins, alteration of which were not related to tissue damage, included a number of well defined (in other systems) humoral constituents of the immune response such as a number of complement components, hemopexin, transferrin, and apolipoprotein.

## 4 Discussion

### 4.1 Monitoring pancreas disease via proteomics

The analysis of spot profiles at weekly sampling points (Fig. 3) demonstrated that the serum proteome of salmon was altered markedly in response to SAV3. In addition, the heat map of proteome responses following the 2-DE showed a distinct change in the serum proteome between pre and post week 4 (Fig. 4), with the exception of week 12, which clustered with samples collected between weeks 1 and 4; thus, indicating the near return of homeostasis. These results corresponded with the histopathological results (Fig. 1) indicating that 2DE could be used as a useful investigative approach to monitor PD. Furthermore, using both histopathology and proteomic approaches allowed proteomic results to be separated into proteins and enzymes which rise or fall in association with tissue damage and those which were part of the host response to SAV3 (Table 3) as their alteration in intensity was unrelated to the histopathology score. The use of hierarchical clustering to analyse spot intensities effectively highlighted the common responses within groups of protein spots and clearly illustrated a temporal trend in the dataset. It is important to note that this analysis was carried out using pooled samples, determined by time point. Whilst, there is an argument for using biological replicates using individual fish it was considered that pooling samples prior to electrophoresis was the optimal approach. As this study was a cohabitation trial there are, due to infection dynamics, fish at various disease stages which would, when using an individual fish approach, potentially require many replicates and pooling of samples was thus required to make the study feasible. Moreover, as this experimental methodology simulates conditions during an outbreak of PD at an aquaculture site it was decided that pooling would allow for the analysis of changes at a site as a whole.

### 4.2 Biomarkers of tissue pathologies

The use of histopathology in conjunction with a 2-DE proteomic approach allowed statistical analysis to be carried out to test the hypothesis that the expression profile of specific proteins was correlated with the pathology of examined tissues. Analysis of this relationship allowed the probable identification of the tissue source of the identified serum proteins.

The abundance of only one spot significantly correlated to the histopathology results of heart damage. This was spot 656 and was identified by MS as the Serotransferrin II. The expression profile of this protein was significantly related to the pathological damage to the heart. The lack of additional biomarkers of heart damage was most likely because the abundances of other potential protein biomarkers were not raised sufficiently in the serum to be detected by the proteomic methodology used.

The pathological damage to the pancreas as well as the white and red skeletal muscle was much more pronounced and longer lasting than that observed in the heart (Fig. 1), and there were more protein spots correlated with histopathology lesion scores (Table 3). Six spots were found to be correlated with damage to pancreas and also correlating to damage to white and red muscle. Three of the spots were identified as triosephosphate isomerase (spots 608, 627, and 628) and were in close proximity on 2-DE gels, whilst the remaining three spots were identified as creatine kinase, alpha-2 enolase-1, and serotransferrin (spots 391, 529 and 545 respectively). Enolase and creatine kinase are enzymes of glycolysis and were presumably derived from the damage to muscle. Transferrins are found in the fibroblasts of the pancreas and skeletal muscles thus it was possible that this serotransferrin was membrane bound/intracellular transferrin isoform that leaked into the circulation due to tissue damage. Only three spots were exclusively correlated with damage to the pancreas (Table 3). One was identified as albumin while the other two spots contained apolipoprotein (spot 260, 575 and 598 respectively). However, as their spot intensity during PD infection only increased by < 2 fold they were unlikely candidates as biomarkers of pancreas damage.

The two types of skeletal muscle studied in this investigation were white and red muscle. These muscle fibres are differentiated by two functional characteristics, specifically contractile speed and metabolic activity. White fibres (fast) possess a higher action potential due to the quicker generation of ATP by glycolysis compared to red (slow) fibres which in general terms generate ATP by oxidative (aerobic) processes. However, glycolysis does occur in red muscle fibres, which explains the finding that many glycolytic enzymes were found to rise in serum spot intensities at the peak of PD pathological damage to both muscle types (Table 3). Many of these glycolytic enzymes have been observed and studied in both muscle types in salmon [25], and have been found to possess higher activity levels in white muscles than in red [26,27]. Only two spots, identified as Glyceraldehyde-3-phosphatedehydrogenase and aldolase A (479 and 500 respectively), were exclusively identified as related to red muscle pathology. Rather than highlighting the metabolic differences between these two types of *Salmo salar* skeletal muscle these differences may indicate that red and white muscle fibres display a differential expression of multiple isozymes of these enzymes.

Conversely this study identified a number of possible unique biomarkers of white muscle damage due to SAV3 as spots 43, 45, 249, 299, 313, 326, 328, and 738 (for protein identification see Tables 2 or 3) were related to histopathological change in white muscle. An explanation of this observation is that in Atlantic salmon the white muscle mass is much greater than red. Complement factor H (CFH) was one of the proteins found in this study to be a possible biomarker of white muscle damage; in fact all three spots identified as this protein (45, 47 and 738) possess expression profiles that correlated significantly with white muscle pathology. This glycoprotein is an important component of the innate immune system with a number of known functions related to it being a regulator of the complement system alternative pathway [28] and acting to reduce local concentrations of toxic products of inflammation [29]. The expression profile of all three spots which contained CFH was that of a continuous rise in intensity until a peak at week 8wpc and then a sharp fall to near basal intensities in week 12.

### 4.3 Humoral components of the serum response during pancreas disease

Complement is a vital component of the immune system of all animals. However, fish are unique in that their complement components exhibit a greater diversity than that of those observed in the mammalian system [30]. In addition to Complement Factor H described above, other Complement components were identified by 2DE and found to change following SAV3 challenge in salmon but without a correlation to histopathology. Thus complement components C3, C9, complement factor B, and the complement inhibitor C1 (spots 444, 146, 357 and 32 respectively), which have been previously characterised as part of the fish innate immune system, were identified in protein spots on 2DE. Interestingly the complement membrane attack complex (MAC), of which C9 is a pivotal component, damages the envelope of enveloped viruses [31]. Moreover, it has been shown that salmonid antibodies are dependent on the presence of complement to neutralize viral hemorrhagic septicemia virus (VHSV) and infectious hematopoietic necrosis virus (IHNV) both of which are enveloped rhabdoviruses [30]. Given that SAV is also an enveloped virus it is possible that complement also plays a role in its neutralization by Atlantic salmon antibodies in vivo. The expression profiles of C3 and complement factor B fell significantly at 5wpc whilst at the same time fractions of immunoglobulins rose sharply, (for example spot 626), which may have indicated that as immunoglobulins were synthesised to combat SAV, complement components declined in their serum concentration. In contrast, the expression profile of C1 inhibitor was substantially different from these previously discussed complement components (Fig. 3). The late peak in serum abundance of this protein at 10 wpc may indicate that in the latter stages of disease recovery (Fig. 4) it was advantageous to inhibit complement activation due to the harmful effects of the alternative pathway and MAC can have on host tissues [32].

Hemopexin-like protein (spots 150, 220, 224, 227 and 565) was found to be significantly altered during SAV3 infection. In mammalian species hemopexin is an acute phase protein (APP), synthesised in hepatocytes and extra-hepatocytic sites to be secreted into circulation, and possesses a high affinity for free circulating haem thus facilitating its clearance [33]. Hemopexin in teleostei is usually referred to as hemopexin-like protein and has been studied at a genetic level by a number of groups. Its expression is highly up regulated during bacterial infections of Atlantic salmon [34] and rainbow trout [35] with the proposal that hemopexin-like protein is up regulated in order to clear free haem from the circulation which would be detrimental to the proliferation of bacteria. In this study the expression profiles of the hemopexin-like protein spots was also found to change following infection. The first four of these spots were in close proximity and exhibited very similar expression profiles remaining near basal week 0 levels until at 5wpc they increased sharply and reached their peak intensity at 10wpc and started to fall again by 12wpc. This may have indicated an increase in serum haem concentration during PD with higher levels of hemopexin being synthesised to aid its clearance. In other teleost species two isoforms of hemopexin-like protein have been identified. For instance, medaka (*Oryzias latipes*) possesses two hemopexin-like proteins which differ not only in their tissue expression locations but also in their ability to bind heam [36]. Interestingly, spot 565 showed the opposite from these previously discussed spots in terms of expression profile. Despite also displaying early stable expression levels it fell sharply in intensity at 5wpc and continued to fall until 10wpc before starting to rise. Therefore, it is possible that Atlantic salmon also possess two isoforms of hemopexin-like protein with the first contained in spots 150, 220, 224 and 227 and the second located in spot 565. However it is possible that alterations in the hemopexin-like protein spots, especially for the lower molecular weight spot 565, could be due to unspecific degradation of proteins of higher molecular weight. It was notable that the matched peptides profiles were similar between the hemopexin-like spots so that the differences in these protein spots could be due to post translational modification or degradation. However it is possible that there could be two homologous isoforms thus more work is required to ascertain if, as in goldfish, salmon possess multiple hemopexin isoforms.

A total of 13 protein spots, differentially expressed during PD, were identified as transferrin (Tf) (Fig. 3). This glycoprotein is one of several humoral proteins with an affinity to iron. This iron binding by Tf facilitates the transport of iron from the circulation to cells [37,38] thus preventing a potentially toxic iron build up, although other functions are known [39]. The intensities of 4 of the 13 Tf spots had a significant relationship with tissue damage (pancreas and skeletal muscle) and have been previously discussed. However, the change in expression of nine Tf spots did not exhibit such a relationship to tissue damage, and were thus deemed components of the immune system response to SAV infection. Of these Tf spots number 151 was by far the largest (in terms of area), migrated with the highest Mr (~ 68 kDa) and remained at a relatively constant expression level until between 8 and 10wpc at which point its expression increased sharply only to fall slightly by 12wpc. This spot was presumably the main protein constituent of Tf in the serum and is the full length isoform. Other spots identified as Tf were smaller in size with lower Mr than spot 151. These could have been degradation products, although Stafford et al. [39,40] have shown that certain transferrin fragments may not be just simply degradation products to be eliminated from the circulation, but play a significant role in the innate immune system of fish. These studies demonstrated the ability of transferrin fragments (but not full length transferrin) of goldfish (*Carassius auratus*) to induce nitric oxide (NO) activation of macrophages which is vital in viral disease recovery since NO is a potent antiviral-agent and immune system modulator [41]. This observation of multiple transferrin fragments with different expression profiles may also help explain the considerable variability in the reporting of transferrin as either a positive or negative APP [38] in the fish immune system as certain approaches in studies may or may not detect these fragments in terms of total transferrin. However, given the large number of spots (13 in total) which contained the protein it cannot be overlooked that, as with other proteins, many of these change in concentration due to unspecific degradation.

Interestingly the S-nitrosylation of glyceraldehyde 3-phosphate dehydrogenase (GAPDH) within cells can initiate a cascade ultimately ending in apoptosis of cells [42]. However, this study not only found GAPDH spots associated with tissue pathologies, but also two spots (493 and 494) which rose dramatically in intensity at 5wpc reaching their peak expression at 6wpc before falling to near basal intensities at 8wpc independently of tissue damage. Since GADPH is regarded as an intracellular enzyme involved in glycolysis this at first may be regarded as a surprising observation. However, endogenous authentic GADPH has previously been shown to be secreted outside of cells without causing cell lysis by Yamaji et. al. [43] leading to the hypothesis that it may possess a role in defence against pathogens. Therefore, GADPH may be classed as an example of a moonlight protein where a primarily intracellular protein is secreted by cells and exhibits very different functions extracellularly. Another intracellular enzyme found by 2DE that may possess ‘moonlighting’ functions extracellularly was the cytoplasmic glycolytic enzyme aldolase. This study found two spots of aldolase that showed no relationship with histopathology (Table 3) though whether there is a moonlighting function of this enzyme is currently unknown.

Other proteins found in this study which increased in serum concentration were the apolipoproteins. Apolipoprotein A-1 together with apolipoprotein A-II constitute the most abundant circulating protein observed in teleosts. Its primary function is the binding and transportation of lipids. However, other antiviral, antimicrobial and anti-inflammatory defensive functions are known [44,45]. This study identified four protein spots containing apolipoprotein which did not exhibit a relationship between expression profile and histology. Interestingly, despite apolipoprotein being widely defined as negative acute phase proteins in mammals all, except spot 668, exhibited a steady increase in intensity with a peak at approximately 6 or 8wpc confirming previous results which found apolipoprotein to be up regulated during fish disease [46,47]. This finding demonstrated a major difference in the expression of this protein during disease in fish and may indicate additional functions of the protein in fish that do not exist in mammalian apolipoprotein. Despite this, as with transferrin, the possibility of this expression profile being due to non specific degradation of protein cannot be overlooked.

Protein synthesis can also fall during disease as a shift due to a preferential synthesis of specific proteins. This is the widely accepted hypothesis of why albumin (an extremely abundant serum protein in all animals) is observed to act as a negative acute phase protein in most instances. This study also observed albumin to be down regulated following SAV3 challenge with the protein spots containing it showing a steady decline in expression. Albumin hepatic expression has also been shown to be reduced in other fish species during a number of diseases [48]. Whilst albumin is a well known negative acute phase protein two other proteins which declined in serum abundance during PD are not as well documented. These are antithrombin (spot 201) and prostaglandin-D synthase (spot 702). Antithrombin inactivates several enzymes involved in the coagulation system and thus down regulation may allow the benefits of coagulation during disease. Prostaglandin-D synthase in serum has not been studied to any extent in fish or other species immunity, although inhibition of the protein has been shown to correlate with muscular necrosis [49] which may have significance in PD and other viral diseases that cause necrosis of muscle fibres.

## 5 Conclusion

This histopathological and proteomic study of PD in Atlantic salmon, S*almo salar*, has identified numerous serum proteins which are altered in abundance during the disease. A correlation between pathology and changes in spot intensity of intracellular proteins and enzymes was established, including variations in a number of tissue specific isozymes. Furthermore alterations, not associated with histopathology, were identified for components of humoral immunity which were presumably involved in both limiting the establishment of PD and aiding the return to homeostasis. A number of proteins, including complement components, apolipoprotein, hemopexin, and transferrin were identified as increasing in serum concentration, whilst albumin and antithrombin levels appeared to decrease during PD. Prior to their use as biomarkers of either tissue damage or humoral response in PD, the diagnostic value of these proteins should be validated by complementary approaches, such as; western blotting, immunohistochemistry and ELISA. However, the proteomics approach described here could be used in to investigate other disease challenge models to look for points of similarity and difference between diseases of importance in aquaculture to identify protein change most associated with morbidity and death, and to deliver insights into disease aetiology and identify mitigation strategies.

## Figures and Tables

**Table 1:** The percentage of samples from salmon sampled at each time point that gave positive results for SAV infection detected by reverse transcriptase polymerase chain reaction (RT-PCR), virus identification (SAV) and virus neutralisation (VN). Where ‘–’ indicates that testing was not carried out.

Sampling time point	Positive samples (% of total)		
RT-PCR	SAV	VN
W0	–	0	0
W2	75	–	–
W3	87	–	–
W4	97	13	27
W5	88	2	67
W6	–	–	–
W8	–	–	–
W10	–	–	–
W12	–	–	–

**Table 2:** Protein spots showing maximum fold increase in spot intensity during the 12 weeks post infection, where negative symbol (-) indicates a fold decrease in spot intensity, compared to pre-infection on week 0.

Spot Number	Identification (Uniprot Reference)	Max. Fold Change	Estimated pI	Estimated MW (kD)	MOWSE Score	Peptide Matches	% coverage
19	Serotransferrin II + Ig heavy chain C region (TRF2SALSA + A46533)	3.1	6.91	112	821, 65	43, 2	38, 5
32	C1 inhibitor (Q70W32)	5.4	5.21	97	137	6	14
43	Serotransferrin II (TRF2SALSA)	4.5	7.36	91	527	14	19
45	Complement factor H (Q2L4Q6)	10.4	8.37	91	79	2	3
47	Alpha-2 enolase-1 + Complement factor H (Q9DDG6 + Q2L4Q6)	3.2	7.52	90	111, 94	3, 3	9, 3
74	No significant identification	4.4	4.33	81			
98	Apolipoprotein A-I precursor (JH0472)	2.6	4.96	76	113	5	23
146	Complement component C9 (Q4QZ25)	2.2	5.2	70	226	7	17
150	Hemopexin-like protein (P79825)	9.6	4.22	68	64	3	9
151	Transferrin + Ig heavy chain C region (T11749 + A46533)	1.5	7.27	68	967, 139	32, 4	35, 12
201	Antithrombin + Complement component C9 (Q9PTA8 + Q4QZ25)	- 2.8	5.43	59	156, 196	8, 6	14, 21
220	Hemopexin-like protein + Beta-actin (P79825 + Q4U1U5)	2.5	6.57	57	166, 107	3, 3	8, 12
224	Hemopexin-like protein (P79825)	2.2	7.5	56	150	7	16
227	Hemopexin-like protein (P79825)	1.5	6.06	56	128	11	14
249	Serotransferrin II (TRF2SALSA)	5.4	8.36	51	101	3	7
260	Serum albumin 2 + Serum albumin 1 (ABONS2 + ABONS1)	- 1.7	4.77	50	364, 320	14, 13	24, 20
299	Enolase (Q7ZZM5)	5	8.03	47	308	14	25
313	Pyruvatekinase (Q8QGU8)	3.5	7.58	46	155	6	11
317	Alpha-2 enolase-1 (Q9DDG6)	7.6	7.94	45	648	37	53
326	Alpha-2 enolase-1 (Q9DDG6)	5.6	7.78	45	560	23	42
328	Alpha-2 enolase-1 (Q9DDG6)	5.2	7.27	44	292	15	39
342	Serum albumin 2 (ABONS2)	- 11.1	5.54	44	666	32	31
357	Serum albumin 2 + Complement factorB/C2-B (ABONS2 + Q9DEC8)	2.4	5.2	41	302, 49	13, 3	18, 3
360	Alpha-2 enolase-1 (Q9DDG6)	15.3	7.77	41	624	28	44
368	Serum albumin 2 + Serum albumin 1(ABONS2 + ABONS1)	- 2.8	4.88	40	318, 295	15, 13	19, 16
381	Alpha-2 enolase-1 + Creatine kinase (Q9DDG6 + Q8JH38)	2.8	7.28	38	704, 101	30, 5	28, 14
386	Aldolase (Q804Y1)	13.8	8.72	38	108	3	23
388	Aldolase (Q804Y1)	8.1	8.62	38	94	1	9
391	Muscle-type creatine kinase CKM1 (Q8JH39)	5.1	7.35	38	252	9	17
393	Glyceraldehyde3-phosphate dehydrogenase (O42259)	15.7	8.79	38	102	9	36
394	Aldolase A (Q8JH72)	11.8	8.82	38	231	11	20
395	Serotransferrin II (TRF2SALSA)	2.2	6.56	38	210	5	9
440	Alpha-2 enolase-1(ABONS1)	4.5	6.64	36	164	3	9
442	Creatine kinase (Q98SS7)	3	7.61	36	218	6	17
444	Complement C3-1 + Cystein proteinase inhibitor protein (P98093 + Q70SU8)	- 3.1	5.4	36	156, 36	10, 2	6, 12
450	Serotransferrin II precursor (TRF2SALSA)	- 2.6	6.79	36	444	15	23
463	Glyceraldehyde3-phosphate dehydrogenase (Q90ZF1)	10.6	8.98	34	247	18	41
472	Serum albumin 2 (Q98SS7)	- 3.7	4.79	34	323	12	19
473	Fructose-bisphosphate aldolase (Q4RVI9)	16.9	8.81	34	98	1	3
477	Aldolase A (Q8JH72)	10.1	8.7	33	130	5	18
479	Glyceraldehyde 3-phosphate dehydrogenase (O42259)	7.1	8.43	33	190	20	34
493	Glyceraldehyde 3-phosphate dehydrogenase (O42259)	11.6	8.18	33	147	13	12
494	Glyceraldehyde 3-phosphate dehydrogenase (Q90ZF1)	15.3	8.2	33	280	13	45
499	Proteasome (Q7ZVP5)	8.2	8	32	35	1	1
500	Aldolase A (Q8JH72)	15.4	8.58	32	110	4	17
509	Serotransferrin II (TRF2SALSA)	- 2.6	6.78	31	250	11	18
529	Alpha-2 enolase-1 (Q9DDG6)	5	7.07	30	80	3	11
545	Serotransferrin II (TRF2SALSA)	- 5.7	6.57	28	64	3	5
548	Serum albumin 1 (P21848)	- 7	6.29	27	122	3	7
556	Serotransferrin 2 + Immunoglobulin light chain (TRF2 + AAG18369)	- 5.3	6.36	27	404,150	18, 7	16, 33
565	Hemopexin-like protein (P79825)	7.8	6.26	26	51	4	8
568	Transferrin (Q8AYG2)	- 3.6	7.36	26	285	12	36
575	Apolipoprotein A-I + Ig light chain precursor (JH0472 + AAG18369)	1.8	6.62	26	188, 76	8, 4	28, 24
584	Malatedehydrogenase1a (B8JMZ0)	5.8	8.88	25	70	1	4
586	Apolipoprotein A-I (JH0472)	1.9	4.76	25	262	8	27
598	Apolipoprotein A-I (JH0472)	1.5	5.15	24	257	19	42
602	No significant identification.	3.6	8.63	24			
608	Triosephosphate isomerase (Q70I40)	2.8	8.5	24	170	6	22
613	Serum albumin 2 (ABONS2)	2.4	8.1	24	488	19	23
623	Triosephosphate isomerase + Ig light chain (Q70I40 + AAG18369)	3.4	8.57	23	305, 109	10, 5	49, 19
624	Triosephosphate isomerase (Q70I40)	2.4	8.1	23	194	11	50
626	Ig light chain (AAG18369)	2.4	7.26	23	190	8	24
627	Triosephosphate isomerase + Ig light chain constant region (Q70I40 + AAN40739)	2.6	7.53	23	131, 87	5, 4	22, 25
628	Triosephosphate isomerase (Q70I40)	3.9	7.85	23	432	19	65
642	Apolipoprotein A-I (JH0472)	3.8	5.57	21	435	27	37
656	Serotransferrin II (TRF2SALSA)	- 5.3	8.63	20	251	6	10
668	Apolipoprotein A-I (JH0472)	- 4.8	5.17	19	325	19	34
669	Apolipoprotein A-1 (JH0472)	- 4.1	5.42	19	221	14	32
687	Serotransferrin II (TRF2SALSA)	4.6	7.93	18	199	7	12
702	Prostaglandin D synthase (Q9DFD7)	- 3.3	8.16	17	82	1	9
738	Putative oncoprotein nm 23 (Q2L4Q6)	7.2	7.3	14	58	11	22
741	Nucleoside diphosphate kinase (Q7ZZQ7)	4.6	6.9	14	58	8	22

**Table 3:** Probability (P) of relation between change in protein spot intensity and histopathology of tissues in salmon infected with SAV.

		Probability (P)			
Spot number	Identification	Pancreas	Heart	Red muscle	White muscle
19	Serotransferrin II + Ig heavy chain C region (TRF2SALSA + A46533)	0.4775	0.7044	0.642	0.8544
32	C1 inhibitor (Q70W32)	0.542	0.3536	0.9656	0.3992
43	Serotransferrin II (TRF2SALSA)	0.0904	0.8329	0.204	0.0166
45	Complement factor H (Q2L4Q6)	0.3102	0.5645	0.1132	0.0061
47	Alpha-2 enolase-1 + Complement factor H (Q9DDG6 + Q2L4Q6)	0.0241	0.7643	0.0663	0.0059
74	No significant identification	0.2794	0.5719	0.4255	0.1125
98	Apolipoprotein A-I precursor (JH0472)	0.2179	0.5856	0.474	0.1619
146	Complement component C9 (Q4QZ25)	0.1032	0.6175	0.8862	0.9013
150	Hemopexin-like protein (P79825)	0.872	0.3269	0.7833	0.804
151	Transferrin + Ig heavy chain C region (T11749 + A46533)	0.0786	0.8847	0.5434	0.3814
201	Antithrombin + Complement component C9 (Q9PTA8 + Q4QZ25)	0.5868	0.5314	0.5774	0.9455
220	Hemopexin-like protein + Beta-actin (P79825 + Q4U1U5)	0.2798	0.4227	0.5775	0.1481
224	Hemopexin-like protein (P79825)	0.2104	0.9728	0.857	0.7601
227	Hemopexin-like protein (P79825)	0.077	0.8458	0.5829	0.4513
249	Serotransferrin II (TRF2SALSA)	0.2894	0.5577	0.1483	0.0339
260	Serum albumin 2 + Serum albumin 1 (ABONS2 + ABONS1)	0.0291	0.1971	0.4523	0.8357
299	Enolase (Q7ZZM5)	0.1996	0.5102	0.1088	0.0032
313	Pyruvatekinase (Q8QGU8)	0.0806	0.8639	0.0559	0.0039
317	Enolase 1 (Alpha) (Q6GMI7)	0.1688	0.6918	0.0963	0.0042
326	Alpha-2 enolase-1 (Q9DDG6)	0.1489	0.6904	0.0853	0.0061
328	Alpha-2 enolase-1 (Q9DDG6)	0.2178	0.5131	0.1028	0.0013
342	Serum albumin 2 (ABONS2)	0.371	0.1541	0.8433	0.3281
357	Serum albumin 2 + Complement factorB/C2-B (ABONS2 + Q9DEC8)	0.1225	0.1539	0.71	0.6958
360	Alpha-2 enolase-1 (Q9DDG6)	0.1997	0.7136	0.047	0.0002
368	Serum albumin 2 + Serum albumin 1 (ABONS2 + ABONS1)	0.0766	0.0806	0.7414	0.6659
381	Alpha-2 enolase-1 + Creatine kinase (Q9DDG6 + Q8JH38)	0.0618	0.9464	0.0272	0.0041
386	Aldolase (Q804Y1)	0.1753	0.911	0.0229	0.0204
388	Aldolase (Q804Y1)	0.153	0.7466	0.0532	0.0752
391	Muscle-type creatine kinase CKM1 (Q8JH39)	0.0462	0.7088	0.004	0.0009
393	Glyceraldehyde3-phosphate dehydrogenase (O42259)	0.1131	0.9435	0.0028	0.0002
394	Aldolase A (Q8JH72)	0.1324	0.9747	0.0067	0.0003
395	Serotransferrin II (TRF2SALSA)	0.0732	0.7892	0.4174	0.1811
440	Alpha-2 enolase-1 (ABONS1)	0.0534	0.8544	0.0097	0.0006
442	Creatine kinase (Q98SS7)	0.0536	0.9545	0.0334	0.0007
444	Complement C3-1 + Cystein proteinase inhibitor protein (P98093 + Q70SU8)	0.3808	0.3852	0.8427	0.4569
450	Serotransferrin II precursor (TRF2SALSA)	0.0744	0.1147	0.8475	0.8393
463	Glyceraldehyde3-phosphatedehydrogenase (Q90ZF1)	0.1712	0.8982	0.0287	0.0277
472	Serum albumin 2 + Creatine kinase (ABONS2 + Q98SS7)	0.2774	0.2544	0.7464	0.3222
473	Fructose-bisphosphate aldolase (Q4RVI9)	0.1771	0.8644	0.0292	0.0336
477	Aldolase A (Q8JH72)	0.1181	0.7005	0.0143	0.0255
479	Glyceraldehyde 3-phosphatedehydrogenase (O42259)	0.0972	0.4788	0.0307	0.1032
493	Glyceraldehyde 3-phosphatedehydrogenase (O42259)	0.2358	0.7868	0.0975	0.1501
494	Glyceraldehyde 3-phosphate dehydrogenase (Q90ZF1)	0.271	0.8078	0.132	0.2332
499	Proteasome (Q7ZVP5)	0.1454	0.6439	0.0706	0.1262
500	Aldolase A (Q8JH72)	0.1836	0.7625	0.039	0.0693
509	Serotransferrin II (TRF2SALSA)	0.1302	0.1569	0.9378	0.5525
529	Alpha-2 enolase-1 (Q9DDG6)	0.0105	0.1837	0.0004	0.0232
545	Serotransferrin II (TRF2SALSA)	0.01	0.1764	0.0002	0.0215
548	Serumalbumin 1 (P21848)	0.381	0.1562	0.7586	0.2887
556	Transferrin + Immunoglobulin light chain (Q8AUU0 + AAG18369)	0.1617	0.063	0.7479	0.5452
565	Hemopexin-like protein (P79825)	0.1514	0.0657	0.7578	0.6317
568	Transferrin (Q8AYG2)	0.1625	0.079	0.6619	0.7647
575	Apolipoprotein A-I + Ig light chain precursor (JH0472 + AAG18369)	0.0124	0.115	0.3685	0.7495
584	Malate dehydrogenase 1a (B8JMZ0)	0.0555	0.7354	0.0006	0.0001
586	Apolipoprotein A-I (JH0472)	0.1272	0.781	0.3957	0.1807
598	Apolipoprotein A-I (JH0472)	0.018	0.3991	0.3503	0.4654
602	No significant identification	0.0931	0.8462	0.0318	0.0005
608	Triosephosphate isomerase (Q70I40)	0.0134	0.6271	0.0079	0.0029
613	Serum albumin 2 (ABONS2)	0.1367	0.6988	0.1639	0.0516
623	Triosephosphate isomerase + Ig light chain (Q70I40 + AAG18369)	0.1508	0.5987	0.0971	0.0068
624	Triosephosphate isomerase (Q70I40)	0.0893	0.8886	0.1385	0.0628
626	Ig light chain (AAG18369)	0.1144	0.7554	0.1929	0.0606
627	Triosephosphate isomerase + Ig light chain constant region (Q70I40 + AAN40739)	0.0396	0.6455	0.0066	0.0115
628	Triosephosphate isomerase (Q70I40)	0.0082	0.3003	0.0001	0.0047
642	Apolipoprotein A-I (JH0472)	0.4075	0.7725	0.675	0.6736
656	Serotransferrin II (TRF2SALSA)	0.0929	0.0296	0.7203	0.6526
668	Apolipoprotein A-I (JH0472)	0.2306	0.1865	0.8973	0.4736
669	Apolipoprotein A-1 (JH0472)	0.5808	0.4595	0.5278	0.3262
687	Serotransferrin II (TRF2SALSA)	0.1577	0.0552	0.8437	0.5952
702	Prostaglandin D synthase (Q9DFD7)	0.1102	0.356	0.2959	0.4512
738	Putative oncoprotein nm 23 (Q2L4Q6)	0.1136	0.9017	0.0695	0.0056
741	Nucleoside diphosphate kinase (Q7ZZQ7)	0.0736	0.8809	0.0467	0.007

## Appendix A Supplementary data

Supplementary data 1: Spot expression profiles for all spots identified by analysis to alter in serum abundance during pancreas disease.

Supplementary data 2: Supplementary data 2: Information on peptide matches made for each spot that was excised for identification.

## References

[1] Munro A.L.S., Ellis E.A.E., McVicar A.H., McLay H.A., Needham E.A. (1984). An exocrine pancreas disease of farmed Atlantic salmon in Scotland. Helgol Meeresun.

[2] McVicar A. (1987). Pancreas disease of farmed Atlantic salmon, Salmo salar, in Scotland: epidemiology and early pathology. Aquaculture.

[3] McLoughlin M., Graham D. (2007). Alphavirus infections in salmonids—a review. J Fish Dis.

[4] Kent M., Elston R. (1987). Pancreas disease in pen-reared Atlantic salmon in North America. Bull Eur Assoc Fish Pathol.

[5] Poppe T., Rimstad E., Hyllseth B. (1989). Pancreas disease in Atlantic salmon (Salmo salar) postsmolts infected with infectious pancreatic necrosis virus (IPNV). Bull Eur Assoc Fish Pathol.

[6] Murphy T.M., Rodger H.D., Drinan E.M., Gannon F., Kruse P., Korting W. (1992). The sequential pathology of pancreas disease in Atlantic salmon farms in Ireland. J Fish Dis.

[7] Raynard R., H.G.M.A.L.S. (1992). Pancreas disease of Atlantic salmon: proceedings of a European Commission Workshop.

[8] Nelson R.T., McLoughlin M.F., Rowley H.M., Platten M.A., McCormick J.I. (1995). Isolation of a toga-like virus from farmed Atlantic salmon Salmo salar with pancreas disease. Dis Aquat Org.

[9] Castric J., Baudin Laurencin F., Bremont M., Jeffroy J., Le Ven A., Bearxotti M. (1997). Isolation of the virus responsible for sleeping disease in experimentally infected rainbow trout (Oncorhynchus mykiss). Bull Eur Assoc Fish Pathol.

[10] Weston J., Villoing S., Bremont M., Castric J., Pfeffer M., Jewhurst V. (2002). Comparison of two aquatic alphaviruses, salmon pancreas disease virus and sleeping disease virus, by using genome sequence analysis, monoclonal reactivity, and cross-infection. J Virol.

[11] Fringuelli E., Rowley H.M., Wilson J.C., Hunter R., Rodger H., Graham D.A. (2008). Phylogenetic analyses and molecular epidemiology of European salmonid alphaviruses (SAV) based on partial E2 and nsP3 gene nucleotide sequences. J Fish Dis.

[12] Graham D.A., Fringuelli E., Wilson C., Rowley H.M., Brown A., Rodger H. (2010). Prospective longitudinal studies of salmonid alphavirus infections on two Atlantic salmon farms in Ireland; evidence for viral persistence. J Fish Dis.

[13] Graham D.A., Fringuelli E., Rowley H.M., Cockerill D., Cox D.I., Turnbull T. (2012). Geographical distribution of salmonid alphavirus subtypes in marine farmed Atlantic salmon, Salmo salar L., in Scotland and Ireland. J Fish Dis.

[14] Jansen M.D., Taksdal T., Wasmuth M.A., Gjerset B., Brun E., Olsen A.B. (2010). Salmonid alphavirus (SAV) and pancreas disease (PD) in Atlantic salmon, Salmo salar L., in freshwater and seawater sites in Norway from 2006 to 2008. J Fish Dis.

[15] Graham D.A., Frost P., McLaughlin K., Rowley H.M., Gabestad I., Gordon A. (2011). A comparative study of marine salmonid alphavirus subtypes 1–6 using an experimental cohabitation challenge model. J Fish Dis.

[16] Christie K.E., Graham D.A., McLoughlin M.F., Villoing S., Todd D., Knappskog D. (2007). Experimental infection of Atlantic salmon Salmo salar pre-smolts by ip injection with new Irish and Norwegian salmonid alphavirus (SAV) isolates: a comparative study. Dis Aquat Organ.

[17] Kongtorp R.T., Sten A., Andreassen P.A., Aspehaug V., Graham D.A., Lyngstad T.M. (2010). Lack of evidence for vertical transmission of SAV 3 using gametes of Atlantic salmon, Salmo salar L., exposed by natural and experimental routes. J Fish Dis.

[18] Rodger H., Mitchell S. (2007). Epidemiological observations of pancreas disease of farmed Atlantic salmon, Salmo salar L., in Ireland. J Fish Dis.

[19] Bratland A., Nylund A. (2009). Studies on the possibility of vertical transmission of Norwegian salmonid alphavirus in production of Atlantic salmon in Norway. J Aquat Anim Health.

[20] Bendixen E., Danielsen M., Hollung K., Gianazza E., Miller I. (2011). Farm animal proteomics—a review. J Proteomics.

[21] Forné I., Agulleiro M.J., Asensio E., Abián J., Cerdà J. (2009). 2-D DIGE analysis of Senegalese sole (*Solea senegalensis*) testis proteome in wild-caught and hormone-treated F1 fish. Proteomics.

[22] Eckersall P.D., Whitfield P.D. (2011). Methods in animal proteomics.

[23] Rodrigues P.M., Silva T.S., Dias J., Jessen F. (2012). Proteomics in aquaculture: applications and trends. J Proteomics.

[24] McLoughlin M.F., Graham D.A., Norris A., Matthews D., Foyle L., Rowley H.M. (2006). Virological, serological and histopathological evaluation of fish strain susceptibility to experimental infection with salmonid alphavirus. Dis Aquat Organ.

[25] Halver J.E., Halver J.E. (1972). Enzyme and systems of intermediary metabolism in “Fish nutrition”.

[26] Johnston I.A. (1977). A comparative study of glycolysis in red and white muscles of the trout (*Salmo gairdneri*) and mirror carp (*Cyprinus carpio*). J Fish Biol.

[27] Martínez M., Bédard M., Dutil J.D., Guderley H. (2004). Does condition of Atlantic cod (*Gadus morhua*) have a greater impact upon swimming performance at Ucrit or sprint speeds?. J Exp Biol.

[28] Meri S., Pangburn M.L. (1990). Discrimination between activators and nonactivators of the alternative pathway of complement: regulation via a sialic acid/polyanion binding site on factor H. Proc Natl Acad Sci.

[29] Józsi M., Manuelian T., Heinen S., Oppermann M., Zipfel P.F. (2004). Attachment of the soluble complement regulator factor H to cell and tissue surfaces: relevance for pathology. Histol Histopathol.

[30] Holland M.C.H., Lambris J.D. (2002). The complement system in teleosts. Fish Shellfish Immunol.

[31] Nakao M., Tsujikura M., Ichiki S., Vo T.K., Somamoto T. (2011). The complement system in teleost fish: progress of post-homolog-hunting researches. Dev Comp Immunol.

[32] Lorenzen N., Lapatra S.E. (1999). Immunity to rhabdoviruses in rainbow trout: the antibody response. Fish Shellfish Immunol.

[33] Wicher K.B., Fries E. (2010). Evolutionary aspects of hemoglobin scavengers. Antioxid Redox Signal.

[34] Tsoi S.C., Ewart K.V., Penny S., Melville K., Liebscher R.S., Brown L.L. (2004). Identification of immune-relevant genes from Atlantic salmon using suppression subtractive hybridization. Marine Biotechnol.

[35] Bayne C.J., Gerwick L., Fujiki K., Nakao M., Yano T. (2001). Immune-relevant (including acute phase) genes identified in the livers of rainbow trout, *Oncorhynchus mykiss*, by means of suppression subtractive hybridization. Dev Comp Immunol.

[36] Hirayama M., Kobiyama A., Kinoshita S., Watabe S. (2004). The occurrence of two types of hemopexin-like protein in medaka and differences in their affinity to heme. J Exp Biol.

[37] Ellis A. (2001). Innate host defense mechanisms of fish against viruses and bacteria. Dev Comp Immunol.

[38] Bayne C.J., Gerwick L. (2001). The acute phase response and innate immunity of fish. Dev Comp Immunol.

[39] Stafford J.L., Belosevic M. (2003). Transferrin and the innate immune response of fish: identification of a novel mechanism of macrophage activation. Dev Comp Immunol.

[40] Stafford J.L., Neumann N., Belosevic M. (2001). Products of proteolytic cleavage of transferrin induce nitric oxide response of goldfish macrophages. Dev Comp Immunol.

[41] Akaike T., Maeda H. (2000). Nitric oxide and virus infection. Immunology.

[42] Hara M.R., Agrawal N., Kim S.F., Cascio M.B., Fujimuro M., Ozeki Y. (2005). S-nitrosylated GAPDH initiates apoptotic cell death by nuclear translocation following Siah1 binding. Nat Cell Biol.

[43] Yamaji R., Chatani E., Harada N., Sugimoto K., Inui H., Nakano Y. (2005). Glyceraldehyde-3-phosphate dehydrogenase in the extracellular space inhibits cell spreading. Biochim Biophys Acta Gen Subj.

[44] Villarroel F., Bastías A., Casado A., Amthauer R., Concha M.I. (2007). Apolipoprotein AI, an antimicrobial protein in *Oncorhynchus mykiss*: evaluation of its expression in primary defence barriers and plasma levels in sick and healthy fish. Fish Shellfish Immunol.

[45] Whyte S.K. (2007). The innate immune response of finfish—a review of current knowledge. Fish Shellfish Immunol.

[46] Magnadóttir B. (2006). Innate immunity of fish (overview). Fish Shellfish Immunol.

[47] Russell S., Hayes M.A., Simko E., Lumsden J.S. (2006). Plasma proteomic analysis of the acute phase response of rainbow trout (Oncorhynchus mykiss) to intraperitoneal inflammation and LPS injection. Dev Comp Immunol.

[48] Gerwick L., Corley-Smith G., Bayne C.J. (2007). Gene transcript changes in individual rainbow trout livers following an inflammatory stimulus. Fish Shellfish Immunol.

[49] Mohri I., Aritake K., Taniguchi H., Sato Y., Kamauchi S., Nagata N. (2009). Inhibition of prostaglandin D synthase suppresses muscular necrosis. Am J Pathol.

