# Supplementary figures and images for: The serum proteome of Atlantic salmon, Salmo salar, during pancreas disease (PD) following infection with salmonid alphavirus subtype 3 (SAV3)
Source: J Proteomics. 2013 Dec 6;94:423–36. doi: 10.1016/j.jprot.2013.10.016 (PMC3878379; doi:10.1016/j.jprot.2013.10.016)

**Supplementary information for Proteomics Paper**

**Spot profiles:
19**


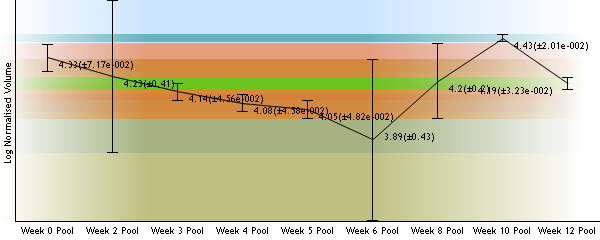


**32**


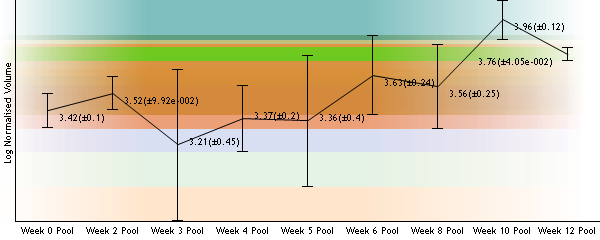


**43**


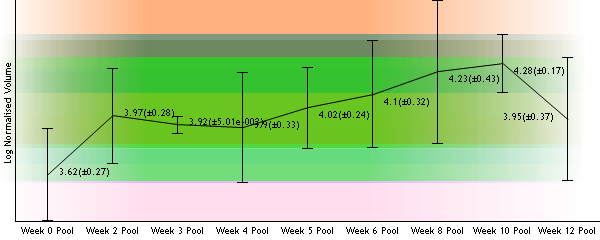


**45**


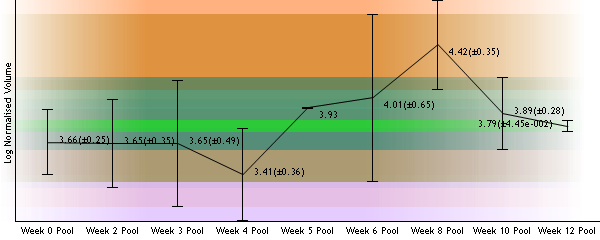


**47**


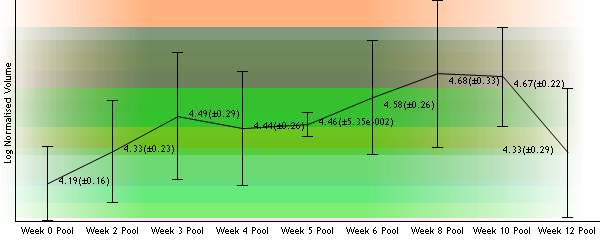


**74**


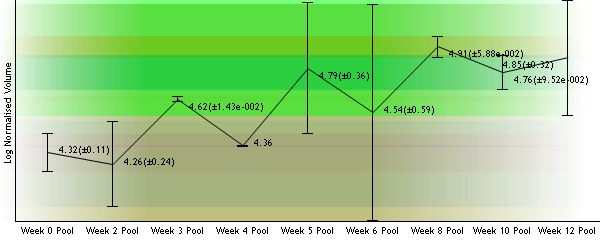


**98**


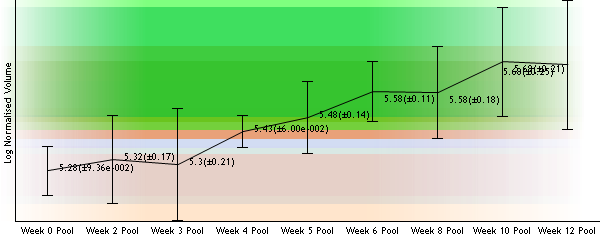


**146**


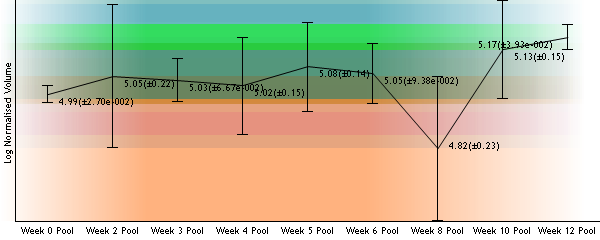


**150**


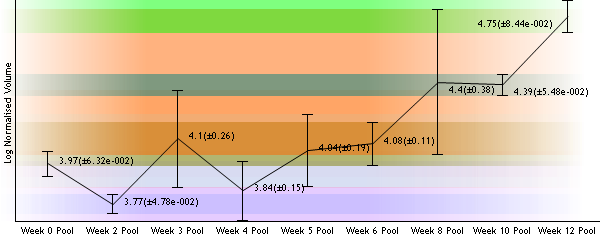


**151**


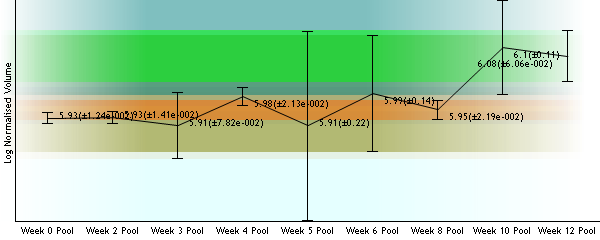


**201**


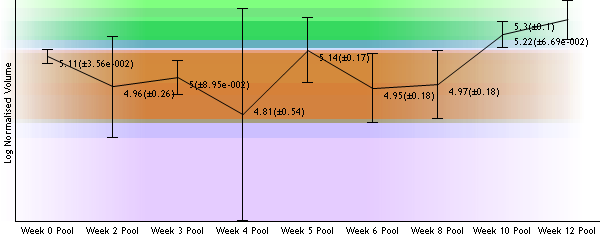


**220**


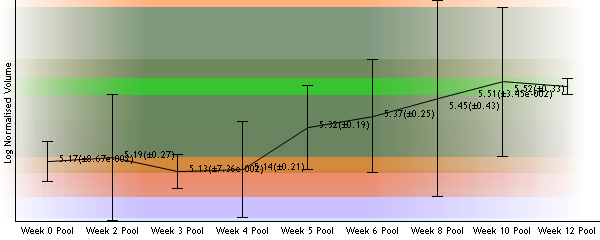


**224**


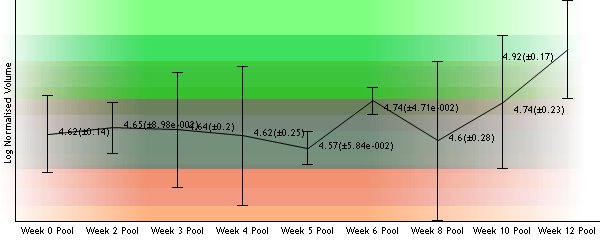


**227**


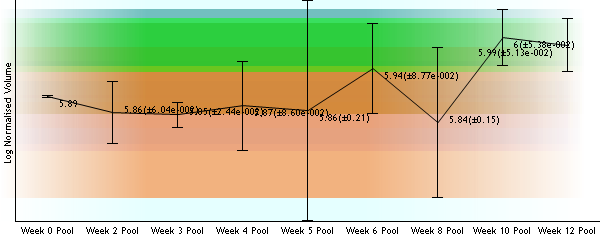


**249**


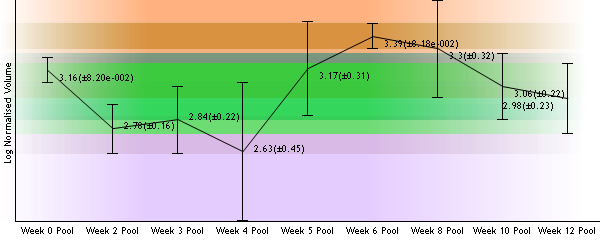


**260**


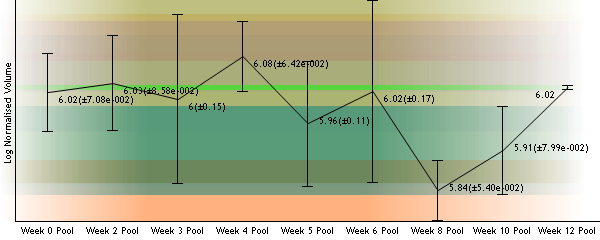


**299**


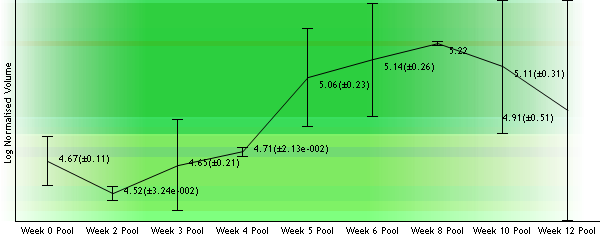


**313**


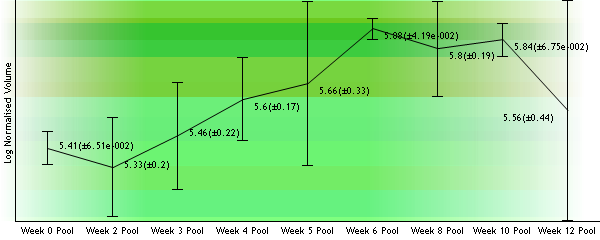


**317**


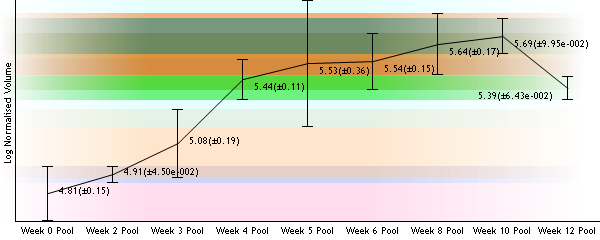


**326**


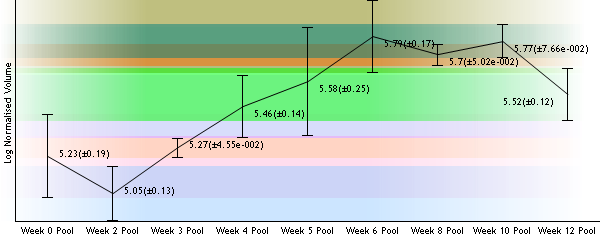


**328**


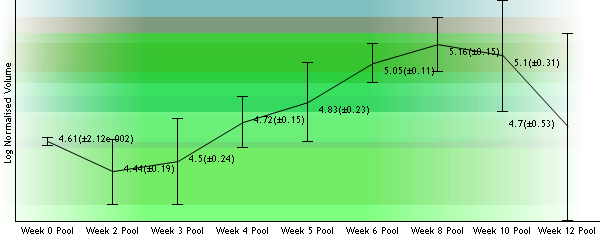


**342**


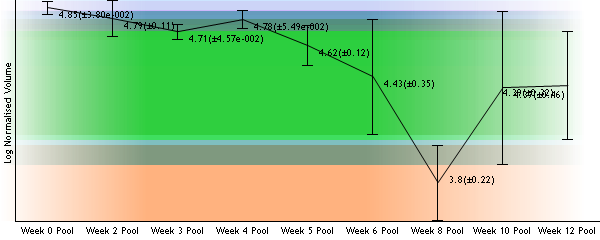


**357**


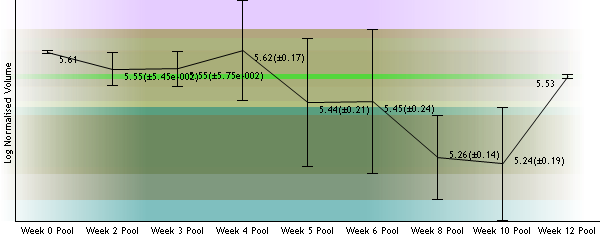


**360**


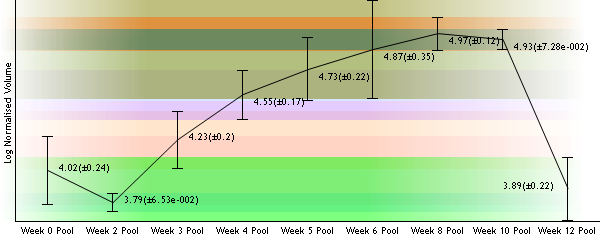


**368**


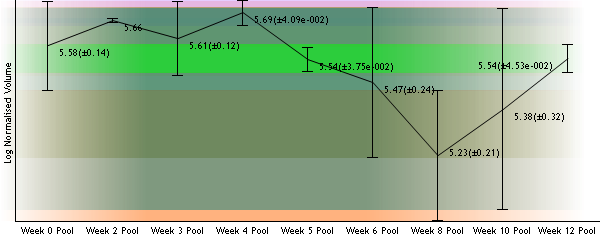


**381**


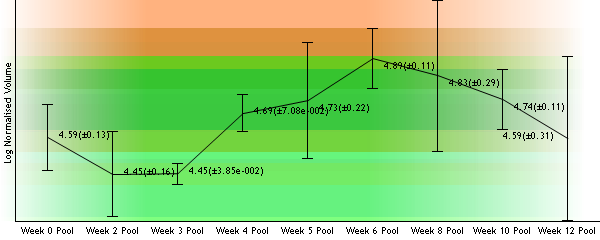


**386**


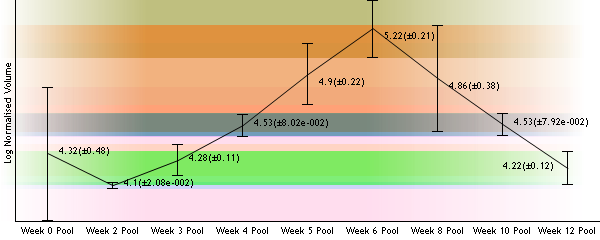


**388**


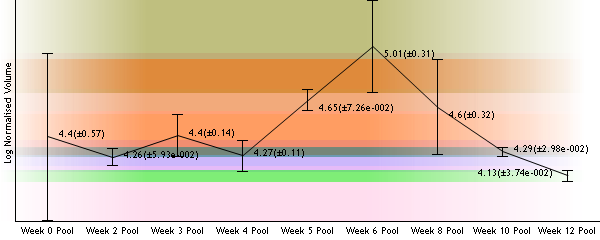


**391**


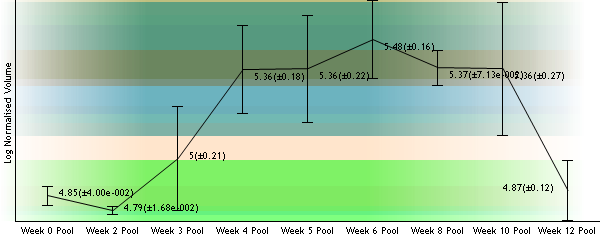


**393**


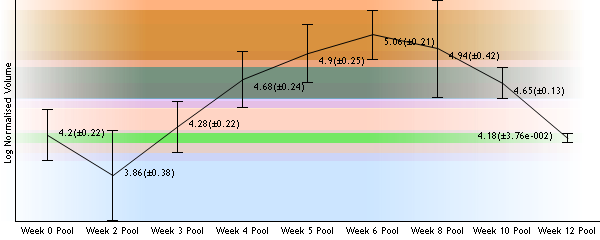


**394**


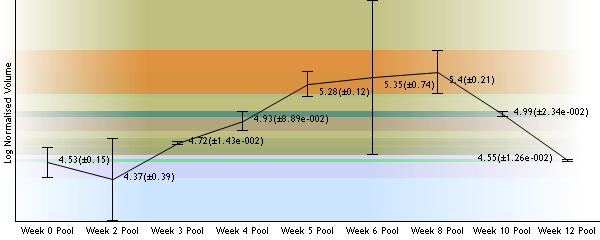


**395**


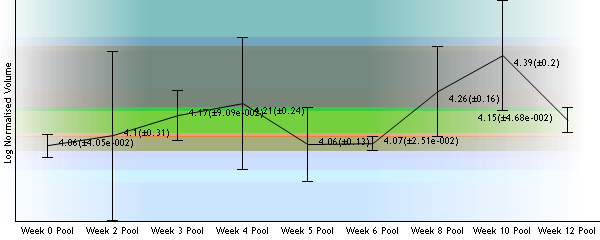


**440**


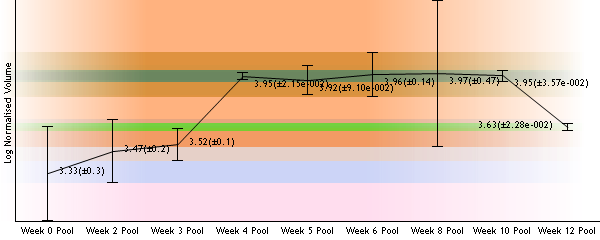


**442**


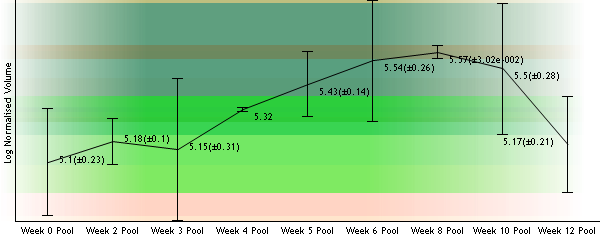


**444**


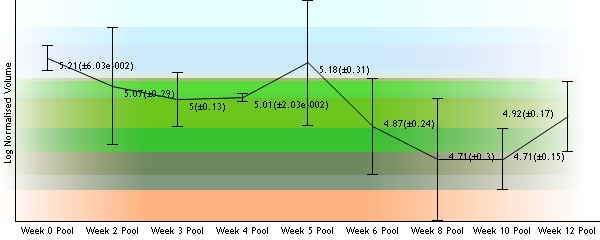


**450**


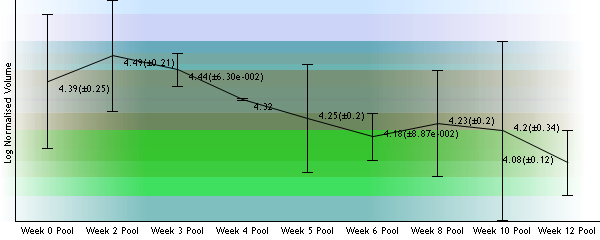


**463**


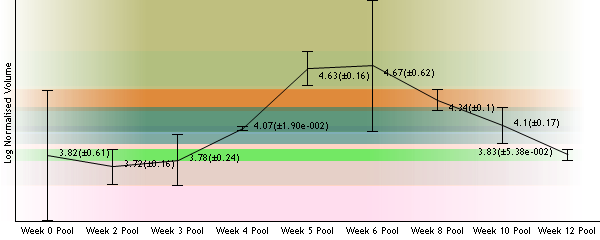


**472**


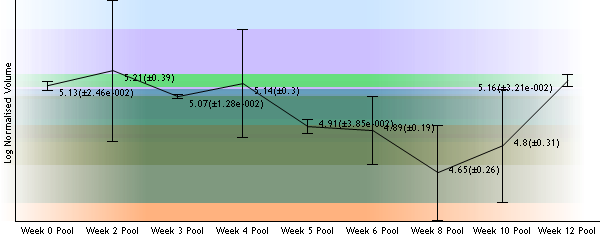


**473**


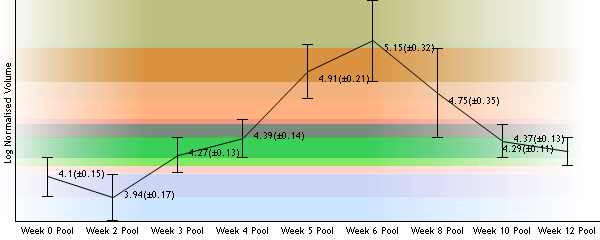


**477**


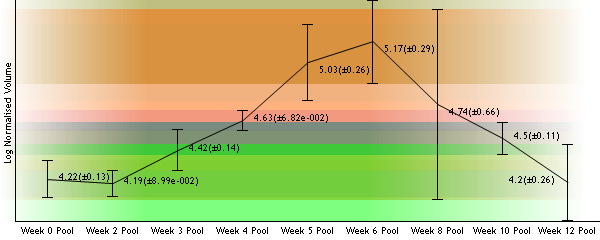


**479**


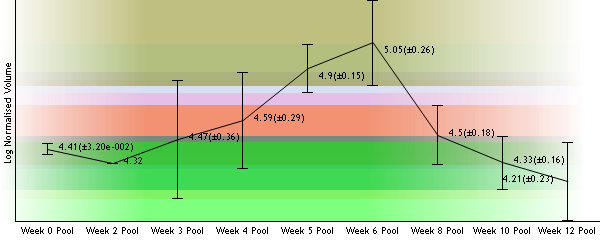


**493**


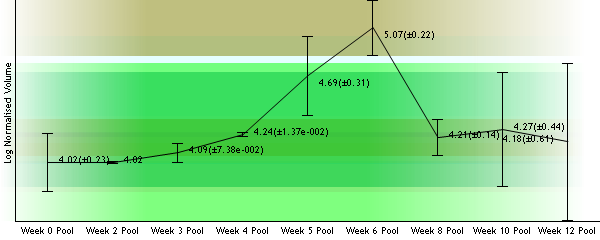


**494**


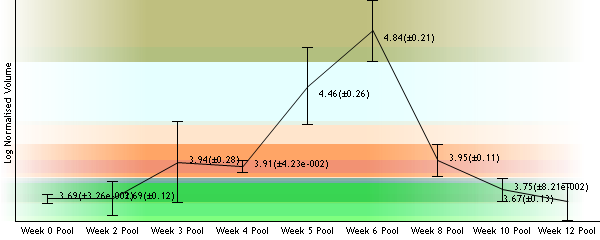


**499**


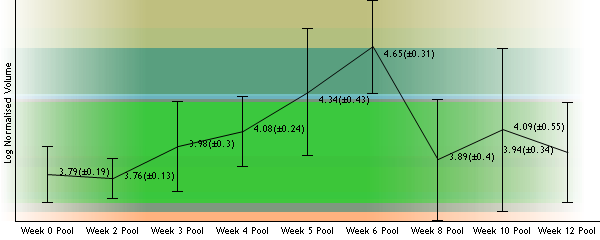


**500**


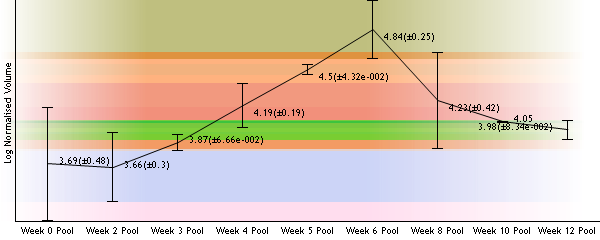


**509**


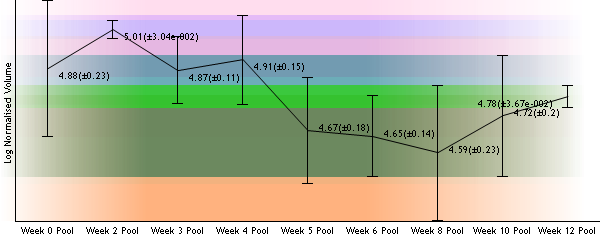


**529**


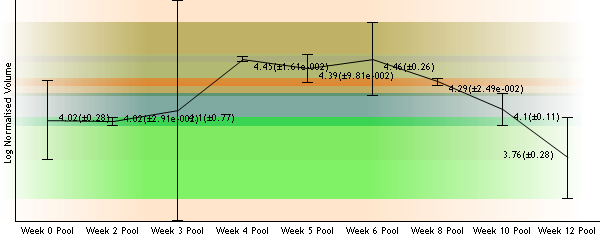


**545**


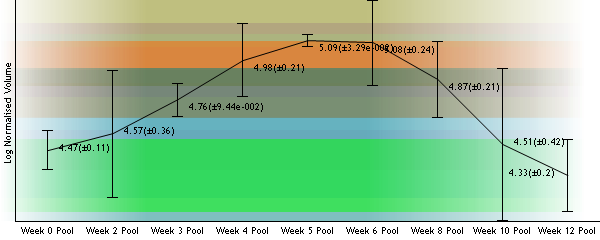


**548**


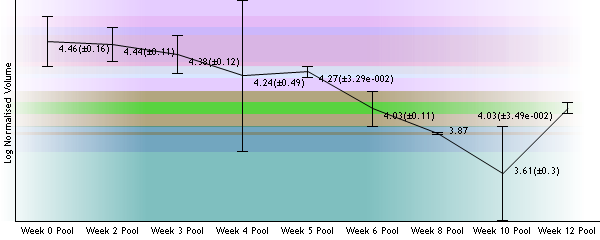


**556**


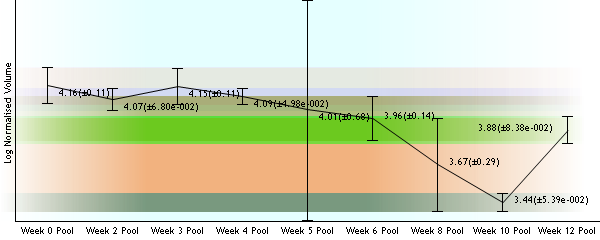


**565**


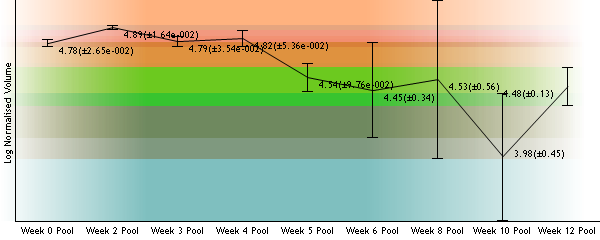


**568**


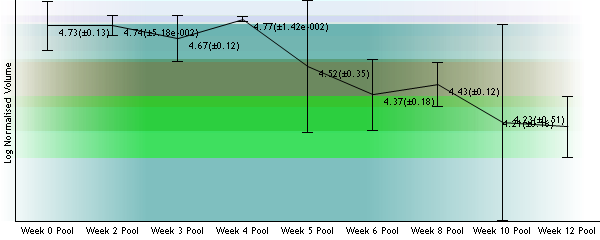
**575**


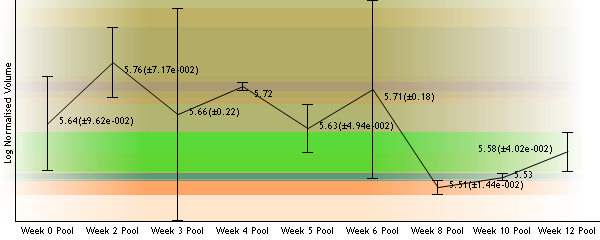


**584**


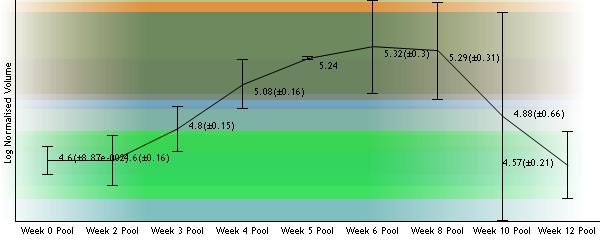


**586**


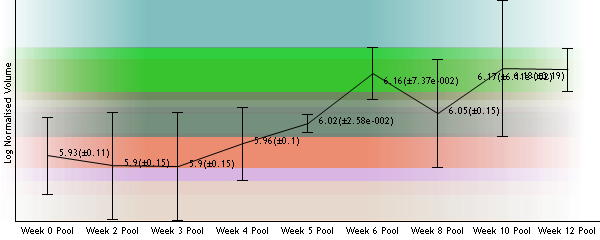


**598**


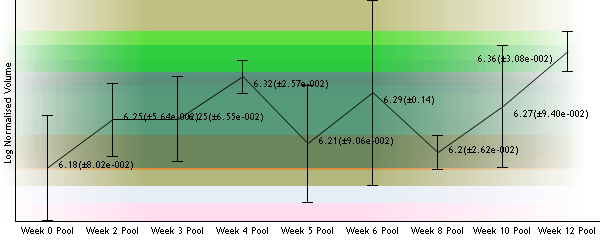


**602**


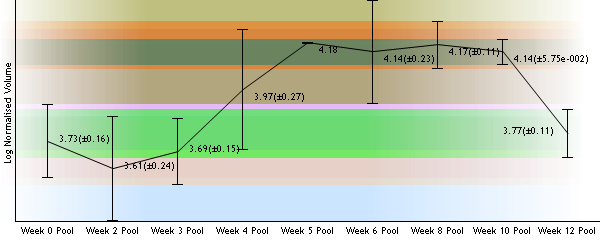


**608**


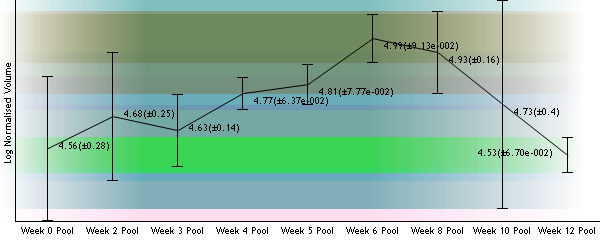


**613**


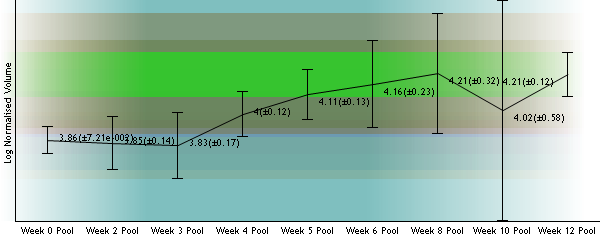


**623**


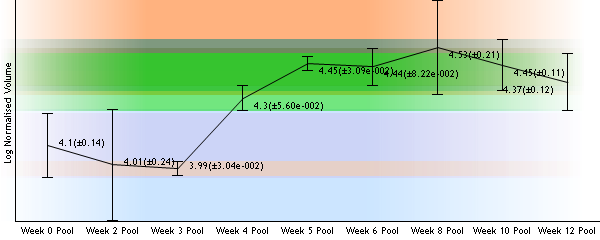


**624**


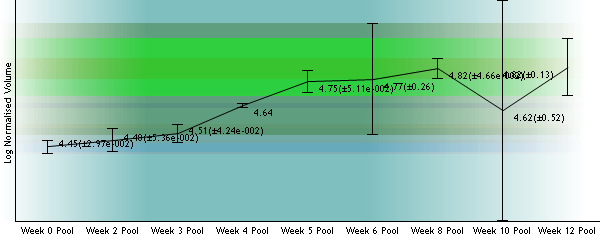


**626**


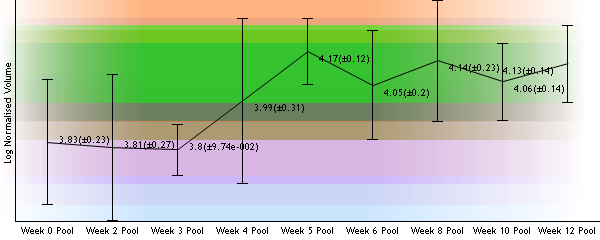


**627**


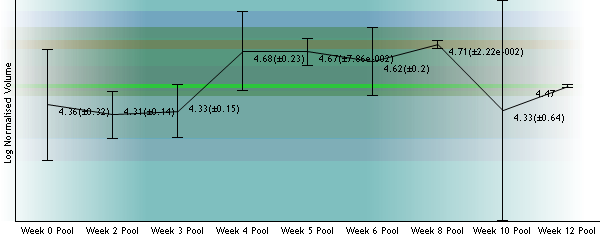


**628**


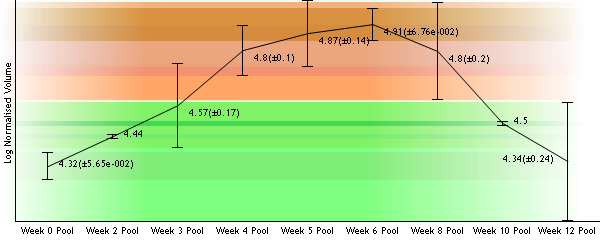


**642**


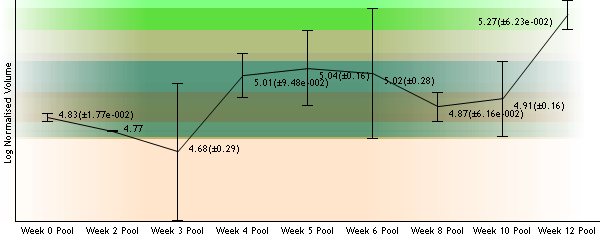


**656**


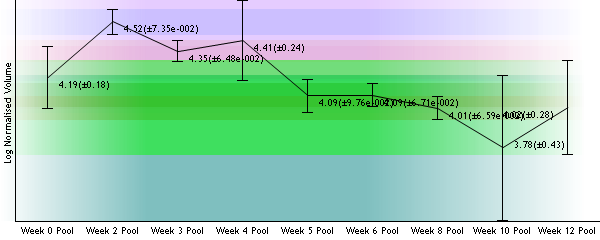


**668**


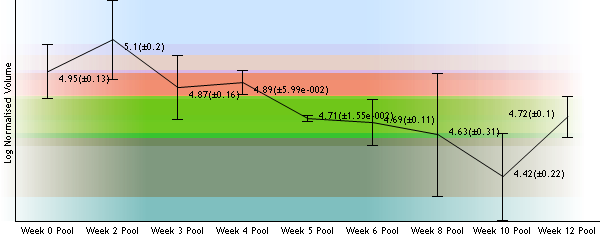


**669**


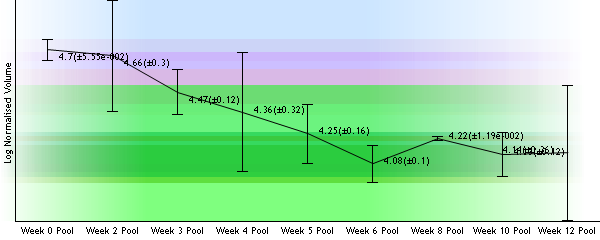


**687**


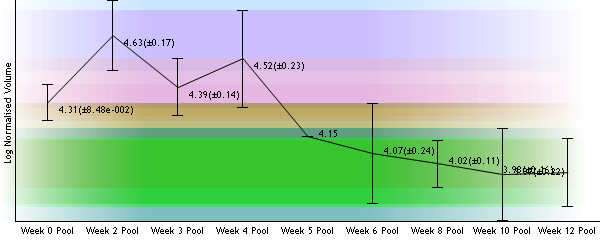


**702**


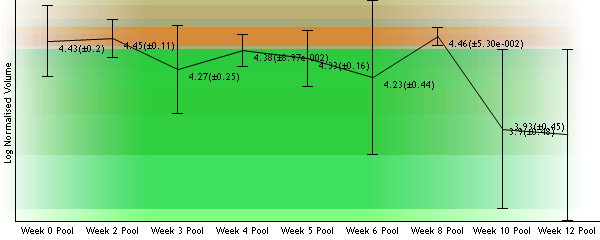


**738**


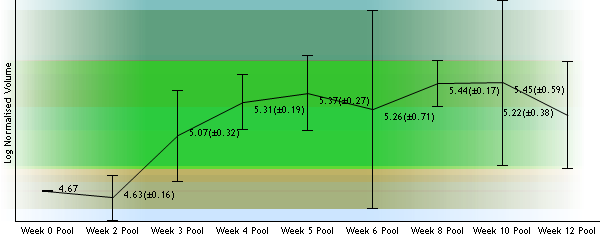


**741**


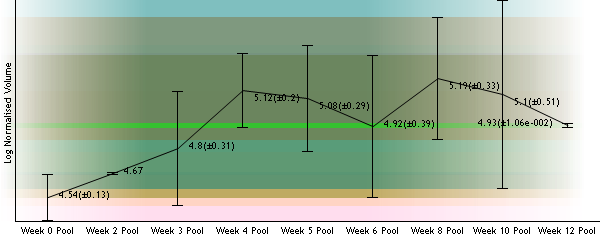

Supplement: Supplementary data 1: — Spot expression profiles for all spots identified by analysis to alter in serum abundance during pancreas disease. [file mmc1.docx]
