# Supplementary material for: The serum proteome of Atlantic salmon, Salmo salar, during pancreas disease (PD) following infection with salmonid alphavirus subtype 3 (SAV3)
Source: J Proteomics. 2013 Dec 6;94:423–36. doi: 10.1016/j.jprot.2013.10.016 (PMC3878379; doi:10.1016/j.jprot.2013.10.016)
Supplement: Supplementary data 2: — Information on peptide matches made for each spot that was excised for identification. [file mmc2.docx]

**SPOT # 494**

**Protein View: Q90ZF1_ONCMY**

**Glyceraldehyde 3-phosphate dehydrogenase (EC 1.2.1.12).- Oncorhynchus mykiss (Rainbow trout) (Salmo gairdneri).**

Top of Form

| **Database:** | MSDB |
| --- | --- |
| **Score:** | 280 |
| **Nominal mass (M_r_):** | 36055 |
| **Calculated pI:** | 8.63 |
| **Taxonomy:** | [Oncorhynchus mykiss](http://www.ncbi.nlm.nih.gov/Taxonomy/Browser/wwwtax.cgi?lvl=0&id=8022) |

This protein sequence matches the following other entries:

- BAB62189 from [Oncorhynchus mykiss](http://www.ncbi.nlm.nih.gov/Taxonomy/Browser/wwwtax.cgi?lvl=0&id=8022)

Sequence similarity is available as [an NCBI BLAST search of Q90ZF1_ONCMY against nr](http://www.ncbi.nlm.nih.gov/blast/Blast.cgi?ALIGNMENTS=50;ALIGNMENT_VIEW=Pairwise;AUTO_FORMAT=Semiauto;CDD_SEARCH=on;CLIENT=web;COMPOSITION_BASED_STATISTICS=on;DATABASE=nr;DESCRIPTIONS=100;ENTREZ_QUERY=%28none%29;EXPECT=10;FILTER=L;FORMAT_BLOCK_ON_RESPAGE=None;FORMAT_OBJECT=Alignment;FORMAT_TYPE=HTML;GAPCOSTS=11%201;I_THRESH=0.001;LAYOUT=TwoWindows;MATRIX_NAME=BLOSUM62;NCBI_GI=on;PAGE=Proteins;PROGRAM=blastp;QUERY=MVKVGVNGFGRIGRLVTRAAFHSKKGVEIVAINDPFIDLDYMVYMFKYDSTHGRFHGEVKAEGGKLVIDGHKITVFHERDPANIKWGDAGATYVVESTGVFTTIEKASTHLKGGAKRVVISAPSADAPMFVMGVNHEKYENSLKVVSNASCTTNCLAPLAKVIHDNYHIIEGLMSTVHAVTATQKTVDGPSGKLWRDGRGASQNIIPASTGAAKAVGKVIPELNGKITGMAFRVPTPNVSVVDLTVRLEKPASYDAIKKVVKAAADGPMKGILGYTEQQVVSSDFNGDTHSSIFDAGAGIALNDHFVKLVTWYDNEFGYSNRVIDLMAHMATKE;SERVICE=plain;SET_DEFAULTS.x=9;SET_DEFAULTS.y=5;SHOW_OVERVIEW=on;WORD_SIZE=3;END_OF_HTTPGET=Yes).

**Search parameters**

| **MS data file:** | H:\MBA1-G6\1_BA1_01_1633.mgf |
| --- | --- |
| **Enzyme:** | Trypsin: cuts C-term side of KR unless next residue is P. |
| **Fixed modifications:** | [Carbamidomethyl (C)](http://fun-gen1.ibls.gla.ac.uk/mascot/cgi/client.pl?modification;file=..%2Fdata%2F20110930%2FF112332.dat;mod_name=Carbamidomethyl%20%28C%29) |
| **Variable modifications:** | [Oxidation (M)](http://fun-gen1.ibls.gla.ac.uk/mascot/cgi/client.pl?modification;file=..%2Fdata%2F20110930%2FF112332.dat;mod_name=Oxidation%20%28M%29) |

**Protein sequence coverage: 45%**

Matched peptides shown in ***bold red***.

| **1** | MVKVGVNGFG | RIGRLVTRAA | FHSKKGVEIV | AINDPFIDLD | YMVYMFKYDS |
| --- | --- | --- | --- | --- | --- |
| **51** | THGRFHGEVK | AEGGKLVIDG | HK**ITVFHERD** | **PANIKWGDAG** | **ATYVVESTGV** |
| **101** | **FTTIEK**ASTH | LKGGAK**RVVI** | **SAPSADAPMF** | **VMGVNHEK**YE | NSLK**VVSNAS** |
| **151** | **CTTNCLAPLA** | **KVIHDNYHII** | **EGLMSTVHAV** | **TATQK**TVDGP | SGKLWRDGR**G** |
| **201** | **ASQNIIPAST** | **GAAK**AVGKVI | PELNGKITGM | AFR**VPTPNVS** | **VVDLTVRLEK** |
| **251** | **PASYDAIKK**V | VKAAADGPMK | GILGYTEQQV | VSSDFNGDTH | SSIFDAGAGI |
| **301** | ALNDHFVK**LV** | **TWYDNEFGYS** | **NR**VIDLMAHM | ATKE |  |

**SPOT # 360**

**Protein View: Q9DDG6_SALTR**

**Alpha-2 enolase-1 (Fragment).- Salmo trutta (Brown trout).**

Top of Form

| **Database:** | MSDB |
| --- | --- |
| **Score:** | 624 |
| **Nominal mass (M_r_):** | 39667 |
| **Calculated pI:** | 5.78 |
| **Taxonomy:** | [Salmo trutta](http://www.ncbi.nlm.nih.gov/Taxonomy/Browser/wwwtax.cgi?lvl=0&id=8032) |

This protein sequence matches the following other entries:

- AAG16311 from [Salmo trutta](http://www.ncbi.nlm.nih.gov/Taxonomy/Browser/wwwtax.cgi?lvl=0&id=8032)

Sequence similarity is available as [an NCBI BLAST search of Q9DDG6_SALTR against nr](http://www.ncbi.nlm.nih.gov/blast/Blast.cgi?ALIGNMENTS=50;ALIGNMENT_VIEW=Pairwise;AUTO_FORMAT=Semiauto;CDD_SEARCH=on;CLIENT=web;COMPOSITION_BASED_STATISTICS=on;DATABASE=nr;DESCRIPTIONS=100;ENTREZ_QUERY=%28none%29;EXPECT=10;FILTER=L;FORMAT_BLOCK_ON_RESPAGE=None;FORMAT_OBJECT=Alignment;FORMAT_TYPE=HTML;GAPCOSTS=11%201;I_THRESH=0.001;LAYOUT=TwoWindows;MATRIX_NAME=BLOSUM62;NCBI_GI=on;PAGE=Proteins;PROGRAM=blastp;QUERY=TKKGLFRAAVPSGASTGIYEALELRDNDKTRYLGKGVKRAVKHINEFLAPALCNQNVNVLEQEKVDKLMLDMDGTENKSKFGANAILGVSLAVCKAGAAEKGVPLYRHIADLAGNPNXILPCPAFNVINGGSHAGNKLAMQEFMILPIGASNFHEAMRIGAEVYHNLKNVIKAKYGKDATNVGDEGGFAPNILENNEALELLKSAIEKAGYPDKIIIGMDVAASEFYKAGKYDLDFKSPDDPARYITXDQLGDLYKSFIKGYPVQSIEDPFDQDDWAAWSKFTAAVDIQVVGDDLTVTNPKRIQQAVEKKACNCLLLKVNQIGSVTESIKACKLAQSNGWGVMVSHRSGETEDTFIADLVVGL;SERVICE=plain;SET_DEFAULTS.x=9;SET_DEFAULTS.y=5;SHOW_OVERVIEW=on;WORD_SIZE=3;END_OF_HTTPGET=Yes).

**Search parameters**

| **MS data file:** | H:\MBA1-G6\2_BA2_01_1634.mgf |
| --- | --- |
| **Enzyme:** | Trypsin: cuts C-term side of KR unless next residue is P. |
| **Fixed modifications:** | [Carbamidomethyl (C)](http://fun-gen1.ibls.gla.ac.uk/mascot/cgi/client.pl?modification;file=..%2Fdata%2F20110930%2FF112333.dat;mod_name=Carbamidomethyl%20%28C%29) |
| **Variable modifications:** | [Oxidation (M)](http://fun-gen1.ibls.gla.ac.uk/mascot/cgi/client.pl?modification;file=..%2Fdata%2F20110930%2FF112333.dat;mod_name=Oxidation%20%28M%29) |

**Protein sequence coverage: 44%**

Matched peptides shown in ***bold red***.

| **1** | TKKGLFRAAV | PSGASTGIYE | ALELRDNDKT | RYLGKGVKRA | VKHINEFLAP |
| --- | --- | --- | --- | --- | --- |
| **51** | ALCNQNVNVL | EQEKVDKLML | DMDGTENKSK | FGANAILGVS | LAVCKAGAAE |
| **101** | KGVPLYRHIA | DLAGNPNXIL | PCPAFNVING | GSHAGNK**LAM** | **QEFMILPIGA** |
| **151** | **SNFHEAMRIG** | **AEVYHNLK**NV | IKAKYGK**DAT** | **NVGDEGGFAP** | **NILENNEALE** |
| **201** | **LLK**SAIEKAG | YPDK**IIIGMD** | **VAASEFYK**AG | K**YDLDFKSPD** | **DPARYITXDQ** |
| **251** | **LGDLYK**SFIK | **GYPVQSIEDP** | **FDQDDWAAWS** | **KFTAAVDIQV** | **VGDDLTVTNP** |
| **301** | **K**RIQQAVEKK | ACNCLLLK**VN** | **QIGSVTESIK** | ACK**LAQSNGW** | **GVMVSHR**SGE |
| **351** | TEDTFIADLV | VGL |  |  |  |

**SPOT # 317**

**Protein View: Q9DDG6_SALTR**

**Alpha-2 enolase-1 (Fragment).- Salmo trutta (Brown trout).**

Top of Form

| **Database:** | MSDB |
| --- | --- |
| **Score:** | 648 |
| **Nominal mass (M_r_):** | 39667 |
| **Calculated pI:** | 5.78 |
| **Taxonomy:** | [Salmo trutta](http://www.ncbi.nlm.nih.gov/Taxonomy/Browser/wwwtax.cgi?lvl=0&id=8032) |

This protein sequence matches the following other entries:

- AAG16311 from [Salmo trutta](http://www.ncbi.nlm.nih.gov/Taxonomy/Browser/wwwtax.cgi?lvl=0&id=8032)

Sequence similarity is available as [an NCBI BLAST search of Q9DDG6_SALTR against nr](http://www.ncbi.nlm.nih.gov/blast/Blast.cgi?ALIGNMENTS=50;ALIGNMENT_VIEW=Pairwise;AUTO_FORMAT=Semiauto;CDD_SEARCH=on;CLIENT=web;COMPOSITION_BASED_STATISTICS=on;DATABASE=nr;DESCRIPTIONS=100;ENTREZ_QUERY=%28none%29;EXPECT=10;FILTER=L;FORMAT_BLOCK_ON_RESPAGE=None;FORMAT_OBJECT=Alignment;FORMAT_TYPE=HTML;GAPCOSTS=11%201;I_THRESH=0.001;LAYOUT=TwoWindows;MATRIX_NAME=BLOSUM62;NCBI_GI=on;PAGE=Proteins;PROGRAM=blastp;QUERY=TKKGLFRAAVPSGASTGIYEALELRDNDKTRYLGKGVKRAVKHINEFLAPALCNQNVNVLEQEKVDKLMLDMDGTENKSKFGANAILGVSLAVCKAGAAEKGVPLYRHIADLAGNPNXILPCPAFNVINGGSHAGNKLAMQEFMILPIGASNFHEAMRIGAEVYHNLKNVIKAKYGKDATNVGDEGGFAPNILENNEALELLKSAIEKAGYPDKIIIGMDVAASEFYKAGKYDLDFKSPDDPARYITXDQLGDLYKSFIKGYPVQSIEDPFDQDDWAAWSKFTAAVDIQVVGDDLTVTNPKRIQQAVEKKACNCLLLKVNQIGSVTESIKACKLAQSNGWGVMVSHRSGETEDTFIADLVVGL;SERVICE=plain;SET_DEFAULTS.x=9;SET_DEFAULTS.y=5;SHOW_OVERVIEW=on;WORD_SIZE=3;END_OF_HTTPGET=Yes).

**Search parameters**

| **MS data file:** | H:\MBA1-G6\3_BA3_01_1635.mgf |
| --- | --- |
| **Enzyme:** | Trypsin: cuts C-term side of KR unless next residue is P. |
| **Fixed modifications:** | [Carbamidomethyl (C)](http://fun-gen1.ibls.gla.ac.uk/mascot/cgi/client.pl?modification;file=..%2Fdata%2F20110930%2FF112334.dat;mod_name=Carbamidomethyl%20%28C%29) |
| **Variable modifications:** | [Oxidation (M)](http://fun-gen1.ibls.gla.ac.uk/mascot/cgi/client.pl?modification;file=..%2Fdata%2F20110930%2FF112334.dat;mod_name=Oxidation%20%28M%29) |

**Protein sequence coverage: 53%**

Matched peptides shown in ***bold red***.

| **1** | TKKGLFR**AAV** | **PSGASTGIYE** | **ALELR**DNDKT | RYLGKGVKRA | VKHINEFLAP |
| --- | --- | --- | --- | --- | --- |
| **51** | ALCNQNVNVL | EQEKVDKLML | DMDGTENKSK | **FGANAILGVS** | **LAVCK**AGAAE |
| **101** | KGVPLYRHIA | DLAGNPNXIL | PCPAFNVING | GSHAGNK**LAM** | **QEFMILPIGA** |
| **151** | **SNFHEAMRIG** | **AEVYHNLK**NV | IKAK**YGKDAT** | **NVGDEGGFAP** | **NILENNEALE** |
| **201** | **LLK**SAIEKAG | YPDK**IIIGMD** | **VAASEFYK**AG | KYDLDFKSPD | DPAR**YITXDQ** |
| **251** | **LGDLYK**SFIK | **GYPVQSIEDP** | **FDQDDWAAWS** | **KFTAAVDIQV** | **VGDDLTVTNP** |
| **301** | **K**RIQQAVEK**K** | **ACNCLLLKVN** | **QIGSVTESIK** | ACK**LAQSNGW** | **GVMVSHR**SGE |
| **351** | TEDTFIADLV | VGL |  |  |  |

Bottom of Form

**SPOT # 628**

**Protein View: Q70I40_ONCMY**

**Triosephosphate isomerase (Fragment).- Oncorhynchus mykiss (Rainbow trout) (Salmo gairdneri).**

Top of Form

| **Database:** | MSDB |
| --- | --- |
| **Score:** | 432 |
| **Nominal mass (M_r_):** | 22759 |
| **Calculated pI:** | 6.59 |
| **Taxonomy:** | [Oncorhynchus mykiss](http://www.ncbi.nlm.nih.gov/Taxonomy/Browser/wwwtax.cgi?lvl=0&id=8022) |

This protein sequence matches the following other entries:

- CAE45565 from [Oncorhynchus mykiss](http://www.ncbi.nlm.nih.gov/Taxonomy/Browser/wwwtax.cgi?lvl=0&id=8022)

Sequence similarity is available as [an NCBI BLAST search of Q70I40_ONCMY against nr](http://www.ncbi.nlm.nih.gov/blast/Blast.cgi?ALIGNMENTS=50;ALIGNMENT_VIEW=Pairwise;AUTO_FORMAT=Semiauto;CDD_SEARCH=on;CLIENT=web;COMPOSITION_BASED_STATISTICS=on;DATABASE=nr;DESCRIPTIONS=100;ENTREZ_QUERY=%28none%29;EXPECT=10;FILTER=L;FORMAT_BLOCK_ON_RESPAGE=None;FORMAT_OBJECT=Alignment;FORMAT_TYPE=HTML;GAPCOSTS=11%201;I_THRESH=0.001;LAYOUT=TwoWindows;MATRIX_NAME=BLOSUM62;NCBI_GI=on;PAGE=Proteins;PROGRAM=blastp;QUERY=GDKASLGELIKTLNSAKLDPNTEVVCGAPSIYLEFARAKLDPKIGVAAQNCYKVKGGAFTGEISPAMIKDVGVHWVILGHSERRWVFGETDELIGQKCAHALENGLGVIACIGEKLDEREAGITEKVINAQTKHFADNIKDWSKVVLAYEPVWAIGTGKTASPAQAQDVHDKLRQWVKANVSEAVANSVRIIYGGSVTGGTCKELGGMKD;SERVICE=plain;SET_DEFAULTS.x=9;SET_DEFAULTS.y=5;SHOW_OVERVIEW=on;WORD_SIZE=3;END_OF_HTTPGET=Yes).

**Search parameters**

| **MS data file:** | H:\MBA1-G6\4_BA4_01_1636.mgf |
| --- | --- |
| **Enzyme:** | Trypsin: cuts C-term side of KR unless next residue is P. |
| **Fixed modifications:** | [Carbamidomethyl (C)](http://fun-gen1.ibls.gla.ac.uk/mascot/cgi/client.pl?modification;file=..%2Fdata%2F20110930%2FF112335.dat;mod_name=Carbamidomethyl%20%28C%29) |
| **Variable modifications:** | [Oxidation (M)](http://fun-gen1.ibls.gla.ac.uk/mascot/cgi/client.pl?modification;file=..%2Fdata%2F20110930%2FF112335.dat;mod_name=Oxidation%20%28M%29) |

**Protein sequence coverage: 65%**

Matched peptides shown in ***bold red***.

| **1** | GDK**ASLGELI** | **K**TLNSAK**LDP** | **NTEVVCGAPS** | **IYLEFAR**AKL | DPKIGVAAQN |
| --- | --- | --- | --- | --- | --- |
| **51** | CYKVK**GGAFT** | **GEISPAMIKD** | **VGVHWVILGH** | **SER**RWVFGET | DELIGQK**CAH** |
| **101** | **ALENGLGVIA** | **CIGEK**LDERE | AGITEKVINA | QTK**HFADNIK** | **DWSKVVLAYE** |
| **151** | **PVWAIGTGKT** | **ASPAQAQDVH** | **DK**LRQWVK**AN** | **VSEAVANSVR** | **IIYGGSVTGG** |
| **201** | **TCK**ELGGMKD |  |  |  |  |

**SPOT # 473**

**(FOUND BY BLAST TO HAVE 100% HOMOLOGY WITH FRUCTOSE BISPHOSPHATE ALDOLASE Q4RV19)**

**Protein View: Q4RVI9_TETNG**

**Chromosome 15 SCAF14992, whole genome shotgun sequence.- Tetraodon nigroviridis (Green puffer).**

Top of Form

| **Database:** | MSDB |
| --- | --- |
| **Score:** | 98 |
| **Nominal mass (M_r_):** | 39742 |
| **Calculated pI:** | 8.45 |
| **Taxonomy:** | [Tetraodon nigroviridis](http://www.ncbi.nlm.nih.gov/Taxonomy/Browser/wwwtax.cgi?lvl=0&id=99883) |

Sequence similarity is available as [an NCBI BLAST search of Q4RVI9_TETNG against nr](http://www.ncbi.nlm.nih.gov/blast/Blast.cgi?ALIGNMENTS=50;ALIGNMENT_VIEW=Pairwise;AUTO_FORMAT=Semiauto;CDD_SEARCH=on;CLIENT=web;COMPOSITION_BASED_STATISTICS=on;DATABASE=nr;DESCRIPTIONS=100;ENTREZ_QUERY=%28none%29;EXPECT=10;FILTER=L;FORMAT_BLOCK_ON_RESPAGE=None;FORMAT_OBJECT=Alignment;FORMAT_TYPE=HTML;GAPCOSTS=11%201;I_THRESH=0.001;LAYOUT=TwoWindows;MATRIX_NAME=BLOSUM62;NCBI_GI=on;PAGE=Proteins;PROGRAM=blastp;QUERY=MPHAYPFLTPEQKKELSDIAQRIVATGKGILAADESTGSVAKRFQSINAENTEENRRLYRQLLFTADDRIKPCIGGVILFHETMYQKTDDGKPFPDYLKERGMVVGIKVDKGVVPLAGTNGETTTQVDGLYERCAQYKKDGADFAKWRCVLKITPTTPSKLAILENANVLARYASICQMHGIVPIVEPEILPDGDHDLKRCQYITEKVLAAVYKALSDHHVYLEGTLLKPNMVTAGHSCSHKYSNQEIAMATVTALRRTVPPAVPGITFLSGGQSEEEASINLNAMNQCPLHRPWALTFSYGRALQASALKSWGGKKENGKACQEEFIKRALACQGKYASSGTSTAGGESLFVANHAY;SERVICE=plain;SET_DEFAULTS.x=9;SET_DEFAULTS.y=5;SHOW_OVERVIEW=on;WORD_SIZE=3;END_OF_HTTPGET=Yes).

**Search parameters**

| **MS data file:** | H:\MBA1-G6\5_BA5_01_1637.mgf |
| --- | --- |
| **Enzyme:** | Trypsin: cuts C-term side of KR unless next residue is P. |
| **Fixed modifications:** | [Carbamidomethyl (C)](http://fun-gen1.ibls.gla.ac.uk/mascot/cgi/client.pl?modification;file=..%2Fdata%2F20110930%2FF112336.dat;mod_name=Carbamidomethyl%20%28C%29) |
| **Variable modifications:** | [Oxidation (M)](http://fun-gen1.ibls.gla.ac.uk/mascot/cgi/client.pl?modification;file=..%2Fdata%2F20110930%2FF112336.dat;mod_name=Oxidation%20%28M%29) |

**Protein sequence coverage: 3%**

Matched peptides shown in ***bold red***.

| **1** | MPHAYPFLTP | EQKKELSDIA | QRIVATGK**GI** | **LAADESTGSV** | **AK**RFQSINAE |
| --- | --- | --- | --- | --- | --- |
| **51** | NTEENRRLYR | QLLFTADDRI | KPCIGGVILF | HETMYQKTDD | GKPFPDYLKE |
| **101** | RGMVVGIKVD | KGVVPLAGTN | GETTTQVDGL | YERCAQYKKD | GADFAKWRCV |
| **151** | LKITPTTPSK | LAILENANVL | ARYASICQMH | GIVPIVEPEI | LPDGDHDLKR |
| **201** | CQYITEKVLA | AVYKALSDHH | VYLEGTLLKP | NMVTAGHSCS | HKYSNQEIAM |
| **251** | ATVTALRRTV | PPAVPGITFL | SGGQSEEEAS | INLNAMNQCP | LHRPWALTFS |
| **301** | YGRALQASAL | KSWGGKKENG | KACQEEFIKR | ALACQGKYAS | SGTSTAGGES |
| **351** | LFVANHAY |  |  |  |  |

Bottom of Form

**SPOT # 150**

**Protein View: P79825_ONCMY**

**Hemopexin-like protein (Fragment).- Oncorhynchus mykiss (Rainbow trout) (Salmo gairdneri).**

Top of Form

| **Database:** | MSDB |
| --- | --- |
| **Score:** | 64 |
| **Nominal mass (M_r_):** | 51106 |
| **Calculated pI:** | 5.61 |
| **Taxonomy:** | [Oncorhynchus mykiss](http://www.ncbi.nlm.nih.gov/Taxonomy/Browser/wwwtax.cgi?lvl=0&id=8022) |

This protein sequence matches the following other entries:

- CAA92147 from [Oncorhynchus mykiss](http://www.ncbi.nlm.nih.gov/Taxonomy/Browser/wwwtax.cgi?lvl=0&id=8022)

Sequence similarity is available as [an NCBI BLAST search of P79825_ONCMY against nr](http://www.ncbi.nlm.nih.gov/blast/Blast.cgi?ALIGNMENTS=50;ALIGNMENT_VIEW=Pairwise;AUTO_FORMAT=Semiauto;CDD_SEARCH=on;CLIENT=web;COMPOSITION_BASED_STATISTICS=on;DATABASE=nr;DESCRIPTIONS=100;ENTREZ_QUERY=%28none%29;EXPECT=10;FILTER=L;FORMAT_BLOCK_ON_RESPAGE=None;FORMAT_OBJECT=Alignment;FORMAT_TYPE=HTML;GAPCOSTS=11%201;I_THRESH=0.001;LAYOUT=TwoWindows;MATRIX_NAME=BLOSUM62;NCBI_GI=on;PAGE=Proteins;PROGRAM=blastp;QUERY=TMKPLSQTLCLCLVLALSHAHHHAGHQGGEDEGHEGHDHGHHEGLLLDRCQGIEMDAVAVTEEGIPYFFKGGHVFKGFHGKAELSNESFAELDDHHHLGHVDAAFLMHFPDKPTEHDHIFFMLDTKVFSYYKHQLETGFPKDISEVFPGIPDHLDAAVVCPAPDCEEDAVIFFKGDEIYHYNVKTKKVEEKKFEGMPNCTSAFRFMEHYYCFHGHQFSKFDPKTGEVHGRYPKEARDYFMKCSKFGDTTDHIERERCSRVHLDAITSDDAGNIYAFRGHHFLEQDAGNDTWAADTIESDFKELHSEVDATFSYENHLYMVKDDKVYIYKVGDSHTHLDGSPKPLKEVLGVEGPIDAAFVCQDHHIAHVIKGQTVYDVDLKASPPVPVKEGSFTLFNKVDAAMCGPEGVKLFKGNHYFHFQSVKVMLMAKAIPEEHKTALELFGCDH;SERVICE=plain;SET_DEFAULTS.x=9;SET_DEFAULTS.y=5;SHOW_OVERVIEW=on;WORD_SIZE=3;END_OF_HTTPGET=Yes).

**Search parameters**

| **MS data file:** | H:\MBA1-G6\6_BA6_01_1638.mgf |
| --- | --- |
| **Enzyme:** | Trypsin: cuts C-term side of KR unless next residue is P. |
| **Fixed modifications:** | [Carbamidomethyl (C)](http://fun-gen1.ibls.gla.ac.uk/mascot/cgi/client.pl?modification;file=..%2Fdata%2F20110930%2FF112337.dat;mod_name=Carbamidomethyl%20%28C%29) |
| **Variable modifications:** | [Oxidation (M)](http://fun-gen1.ibls.gla.ac.uk/mascot/cgi/client.pl?modification;file=..%2Fdata%2F20110930%2FF112337.dat;mod_name=Oxidation%20%28M%29) |

**Protein sequence coverage: 9%**

Matched peptides shown in ***bold red***.

| **1** | TMKPLSQTLC | LCLVLALSHA | HHHAGHQGGE | DEGHEGHDHG | HHEGLLLDRC |
| --- | --- | --- | --- | --- | --- |
| **51** | QGIEMDAVAV | TEEGIPYFFK | GGHVFKGFHG | KAELSNESFA | ELDDHHHLGH |
| **101** | VDAAFLMHFP | DKPTEHDHIF | FMLDTKVFSY | YKHQLETGFP | KDISEVFPGI |
| **151** | PDHLDAAVVC | PAPDCEEDAV | IFFKGDEIYH | YNVKTKKVEE | KKFEGMPNCT |
| **201** | SAFR**FMEHYY** | **CFHGHQFSK**F | DPKTGEVHGR | YPKEARDYFM | KCSK**FGDTTD** |
| **251** | **HIER**ERCSR**V** | **HLDAITSDDA** | **GNIYAFR**GHH | FLEQDAGNDT | WAADTIESDF |
| **301** | KELHSEVDAT | FSYENHLYMV | KDDKVYIYKV | GDSHTHLDGS | PKPLKEVLGV |
| **351** | EGPIDAAFVC | QDHHIAHVIK | GQTVYDVDLK | ASPPVPVKEG | SFTLFNKVDA |
| **401** | AMCGPEGVKL | FKGNHYFHFQ | SVKVMLMAKA | IPEEHKTALE | LFGCDH |

**SPOT # 326**

**Alpha-2 enolase-1 (Fragment).- Salmo trutta (Brown trout).**

Top of Form

| **Database:** | MSDB |
| --- | --- |
| **Score:** | 560 |
| **Nominal mass (M_r_):** | 39667 |
| **Calculated pI:** | 5.78 |
| **Taxonomy:** | [Salmo trutta](http://www.ncbi.nlm.nih.gov/Taxonomy/Browser/wwwtax.cgi?lvl=0&id=8032) |

This protein sequence matches the following other entries:

- AAG16311 from [Salmo trutta](http://www.ncbi.nlm.nih.gov/Taxonomy/Browser/wwwtax.cgi?lvl=0&id=8032)

Sequence similarity is available as [an NCBI BLAST search of Q9DDG6_SALTR against nr](http://www.ncbi.nlm.nih.gov/blast/Blast.cgi?ALIGNMENTS=50;ALIGNMENT_VIEW=Pairwise;AUTO_FORMAT=Semiauto;CDD_SEARCH=on;CLIENT=web;COMPOSITION_BASED_STATISTICS=on;DATABASE=nr;DESCRIPTIONS=100;ENTREZ_QUERY=%28none%29;EXPECT=10;FILTER=L;FORMAT_BLOCK_ON_RESPAGE=None;FORMAT_OBJECT=Alignment;FORMAT_TYPE=HTML;GAPCOSTS=11%201;I_THRESH=0.001;LAYOUT=TwoWindows;MATRIX_NAME=BLOSUM62;NCBI_GI=on;PAGE=Proteins;PROGRAM=blastp;QUERY=TKKGLFRAAVPSGASTGIYEALELRDNDKTRYLGKGVKRAVKHINEFLAPALCNQNVNVLEQEKVDKLMLDMDGTENKSKFGANAILGVSLAVCKAGAAEKGVPLYRHIADLAGNPNXILPCPAFNVINGGSHAGNKLAMQEFMILPIGASNFHEAMRIGAEVYHNLKNVIKAKYGKDATNVGDEGGFAPNILENNEALELLKSAIEKAGYPDKIIIGMDVAASEFYKAGKYDLDFKSPDDPARYITXDQLGDLYKSFIKGYPVQSIEDPFDQDDWAAWSKFTAAVDIQVVGDDLTVTNPKRIQQAVEKKACNCLLLKVNQIGSVTESIKACKLAQSNGWGVMVSHRSGETEDTFIADLVVGL;SERVICE=plain;SET_DEFAULTS.x=9;SET_DEFAULTS.y=5;SHOW_OVERVIEW=on;WORD_SIZE=3;END_OF_HTTPGET=Yes).

**Search parameters**

| **MS data file:** | H:\MBA1-G6\7_BA7_01_1639.mgf |
| --- | --- |
| **Enzyme:** | Trypsin: cuts C-term side of KR unless next residue is P. |
| **Fixed modifications:** | [Carbamidomethyl (C)](http://fun-gen1.ibls.gla.ac.uk/mascot/cgi/client.pl?modification;file=..%2Fdata%2F20110930%2FF112338.dat;mod_name=Carbamidomethyl%20%28C%29) |
| **Variable modifications:** | [Oxidation (M)](http://fun-gen1.ibls.gla.ac.uk/mascot/cgi/client.pl?modification;file=..%2Fdata%2F20110930%2FF112338.dat;mod_name=Oxidation%20%28M%29) |

**Protein sequence coverage: 42%**

Matched peptides shown in ***bold red***.

| **1** | TKKGLFRAAV | PSGASTGIYE | ALELRDNDKT | RYLGKGVKRA | VKHINEFLAP |
| --- | --- | --- | --- | --- | --- |
| **51** | ALCNQNVNVL | EQEKVDKLML | DMDGTENKSK | **FGANAILGVS** | **LAVCK**AGAAE |
| **101** | KGVPLYRHIA | DLAGNPNXIL | PCPAFNVING | GSHAGNK**LAM** | **QEFMILPIGA** |
| **151** | **SNFHEAMRIG** | **AEVYHNLK**NV | IKAKYGK**DAT** | **NVGDEGGFAP** | **NILENNEALE** |
| **201** | **LLK**SAIEKAG | YPDK**IIIGMD** | **VAASEFYK**AG | KYDLDFKSPD | DPAR**YITXDQ** |
| **251** | **LGDLYK**SFIK | GYPVQSIEDP | FDQDDWAAWS | K**FTAAVDIQV** | **VGDDLTVTNP** |
| **301** | **K**RIQQAVEK**K** | **ACNCLLLKVN** | **QIGSVTESIK** | ACK**LAQSNGW** | **GVMVSHR**SGE |
| **351** | TEDTFIADLV | VGL |  |  |  |

Bottom of Form

Bottom of Form

Bottom of Form

**SPOT # 391**

**Protein View: Q98SS7_GADMO**

**Creatine kinase (Fragment).- Gadus morhua (Atlantic cod).**

Top of Form

| **Database:** | MSDB |
| --- | --- |
| **Score:** | 252 |
| **Nominal mass (M_r_):** | 29122 |
| **Calculated pI:** | 8.89 |
| **Taxonomy:** | [Gadus morhua](http://www.ncbi.nlm.nih.gov/Taxonomy/Browser/wwwtax.cgi?lvl=0&id=8049) |

This protein sequence matches the following other entries:

- AAK17970 from [Gadus morhua](http://www.ncbi.nlm.nih.gov/Taxonomy/Browser/wwwtax.cgi?lvl=0&id=8049)

Sequence similarity is available as [an NCBI BLAST search of Q98SS7_GADMO against nr](http://www.ncbi.nlm.nih.gov/blast/Blast.cgi?ALIGNMENTS=50;ALIGNMENT_VIEW=Pairwise;AUTO_FORMAT=Semiauto;CDD_SEARCH=on;CLIENT=web;COMPOSITION_BASED_STATISTICS=on;DATABASE=nr;DESCRIPTIONS=100;ENTREZ_QUERY=%28none%29;EXPECT=10;FILTER=L;FORMAT_BLOCK_ON_RESPAGE=None;FORMAT_OBJECT=Alignment;FORMAT_TYPE=HTML;GAPCOSTS=11%201;I_THRESH=0.001;LAYOUT=TwoWindows;MATRIX_NAME=BLOSUM62;NCBI_GI=on;PAGE=Proteins;PROGRAM=blastp;QUERY=AYVLSSRVRTGRSIKGFTLPPHNSRGERRTIEKLSIEALATLSGEFKGKYYPLNGMTDKEQDQLINDHFLFDKPVSPLLTCAGMARDWPDARGIWHNDAKTFLVWVNEEDHLRVISMQQGGNMKEVFRRFCTGLLKIEETFKKHNHGFMWNEHLGYVLTCPSNLGTGLRGGVHVKLPKLSTHAKFEEILTRLRLQKRGTGGVDTASVGGVFDISNADRLGSSEVDQVQMVVDGVKLMVAMEKKPEKSESIDDMIPAQK;SERVICE=plain;SET_DEFAULTS.x=9;SET_DEFAULTS.y=5;SHOW_OVERVIEW=on;WORD_SIZE=3;END_OF_HTTPGET=Yes).

**Search parameters**

| **MS data file:** | H:\MBA1-G6\8_BA8_01_1640.mgf |
| --- | --- |
| **Enzyme:** | Trypsin: cuts C-term side of KR unless next residue is P. |
| **Fixed modifications:** | [Carbamidomethyl (C)](http://fun-gen1.ibls.gla.ac.uk/mascot/cgi/client.pl?modification;file=..%2Fdata%2F20110930%2FF112339.dat;mod_name=Carbamidomethyl%20%28C%29) |
| **Variable modifications:** | [Oxidation (M)](http://fun-gen1.ibls.gla.ac.uk/mascot/cgi/client.pl?modification;file=..%2Fdata%2F20110930%2FF112339.dat;mod_name=Oxidation%20%28M%29) |

**Protein sequence coverage: 17%**

Matched peptides shown in ***bold red***.

| **1** | AYVLSSRVRT | GRSIKGFTLP | PHNSRGERRT | IEKLSIEALA | TLSGEFKGKY |
| --- | --- | --- | --- | --- | --- |
| **51** | YPLNGMTDKE | QDQLINDHFL | FDKPVSPLLT | CAGMARDWPD | ARGIWHNDAK |
| **101** | TFLVWVNEED | HLRVISMQQG | GNMKEVFRRF | CTGLLKIEET | FKKHNHGFMW |
| **151** | NEHLGYVLTC | PSNLGTGLRG | GVHVKLPKLS | THAK**FEEILT** | **R**LRLQK**RGTG** |
| **201** | **GVDTASVGGV** | **FDISNADRLG** | **SSEVDQVQMV** | **VDGVK**LMVAM | EKKPEKSESI |
| **251** | DDMIPAQK |  |  |  |  |

**SPOT # 386**

**Protein View: Q804Y1_ICTPU**

**Aldolase (Fragment).- Ictalurus punctatus (Channel catfish).**

Top of Form

| **Database:** | MSDB |
| --- | --- |
| **Score:** | 108 |
| **Nominal mass (M_r_):** | 17424 |
| **Calculated pI:** | 8.73 |
| **Taxonomy:** | [Ictalurus punctatus](http://www.ncbi.nlm.nih.gov/Taxonomy/Browser/wwwtax.cgi?lvl=0&id=7998) |

This protein sequence matches the following other entries:

- AAO25766 from [Ictalurus punctatus](http://www.ncbi.nlm.nih.gov/Taxonomy/Browser/wwwtax.cgi?lvl=0&id=7998)

Sequence similarity is available as [an NCBI BLAST search of Q804Y1_ICTPU against nr](http://www.ncbi.nlm.nih.gov/blast/Blast.cgi?ALIGNMENTS=50;ALIGNMENT_VIEW=Pairwise;AUTO_FORMAT=Semiauto;CDD_SEARCH=on;CLIENT=web;COMPOSITION_BASED_STATISTICS=on;DATABASE=nr;DESCRIPTIONS=100;ENTREZ_QUERY=%28none%29;EXPECT=10;FILTER=L;FORMAT_BLOCK_ON_RESPAGE=None;FORMAT_OBJECT=Alignment;FORMAT_TYPE=HTML;GAPCOSTS=11%201;I_THRESH=0.001;LAYOUT=TwoWindows;MATRIX_NAME=BLOSUM62;NCBI_GI=on;PAGE=Proteins;PROGRAM=blastp;QUERY=MPHAYPFLSPEQKKELSDIALRIVAPGKGILAADESTGSVAKRFQSINAENTEENRRLYRQLLFTADDRVKPCIGGVIFFHETLYQKTDDGKLFPQLIKERGMVVGIKVDKGVVPLAGTNGETTTQGLDGLYERCAQYKKDGADFAKWRCVLKIT;SERVICE=plain;SET_DEFAULTS.x=9;SET_DEFAULTS.y=5;SHOW_OVERVIEW=on;WORD_SIZE=3;END_OF_HTTPGET=Yes).

**Search parameters**

| **MS data file:** | H:\MBA1-G6\9_BA9_01_1641.mgf |
| --- | --- |
| **Enzyme:** | Trypsin: cuts C-term side of KR unless next residue is P. |
| **Fixed modifications:** | [Carbamidomethyl (C)](http://fun-gen1.ibls.gla.ac.uk/mascot/cgi/client.pl?modification;file=..%2Fdata%2F20110930%2FF112340.dat;mod_name=Carbamidomethyl%20%28C%29) |
| **Variable modifications:** | [Oxidation (M)](http://fun-gen1.ibls.gla.ac.uk/mascot/cgi/client.pl?modification;file=..%2Fdata%2F20110930%2FF112340.dat;mod_name=Oxidation%20%28M%29) |

**Protein sequence coverage: 23%**

Matched peptides shown in ***bold red***.

| **1** | MPHAYPFLSP | EQKKELSDIA | LRIVAPGK**GI** | **LAADESTGSV** | **AK**RFQSINAE |
| --- | --- | --- | --- | --- | --- |
| **51** | NTEENRRLYR | QLLFTADDRV | KPCIGGVIFF | HETLYQKTDD | GKLFPQLIKE |
| **101** | RGMVVGIKVD | K**GVVPLAGTN** | **GETTTQGLDG** | **LYER**CAQYKK | DGADFAKWRC |
| **151** | VLKIT |  |  |  |  |

**SPOT # 393**

**Protein View: Q90ZF1_ONCMY**

**Glyceraldehyde 3-phosphate dehydrogenase (EC 1.2.1.12).- Oncorhynchus mykiss (Rainbow trout) (Salmo gairdneri).**

Top of Form

| **Database:** | MSDB |
| --- | --- |
| **Score:** | 102 |
| **Nominal mass (M_r_):** | 36055 |
| **Calculated pI:** | 8.63 |
| **Taxonomy:** | [Oncorhynchus mykiss](http://www.ncbi.nlm.nih.gov/Taxonomy/Browser/wwwtax.cgi?lvl=0&id=8022) |

This protein sequence matches the following other entries:

- BAB62189 from [Oncorhynchus mykiss](http://www.ncbi.nlm.nih.gov/Taxonomy/Browser/wwwtax.cgi?lvl=0&id=8022)

Sequence similarity is available as [an NCBI BLAST search of Q90ZF1_ONCMY against nr](http://www.ncbi.nlm.nih.gov/blast/Blast.cgi?ALIGNMENTS=50;ALIGNMENT_VIEW=Pairwise;AUTO_FORMAT=Semiauto;CDD_SEARCH=on;CLIENT=web;COMPOSITION_BASED_STATISTICS=on;DATABASE=nr;DESCRIPTIONS=100;ENTREZ_QUERY=%28none%29;EXPECT=10;FILTER=L;FORMAT_BLOCK_ON_RESPAGE=None;FORMAT_OBJECT=Alignment;FORMAT_TYPE=HTML;GAPCOSTS=11%201;I_THRESH=0.001;LAYOUT=TwoWindows;MATRIX_NAME=BLOSUM62;NCBI_GI=on;PAGE=Proteins;PROGRAM=blastp;QUERY=MVKVGVNGFGRIGRLVTRAAFHSKKGVEIVAINDPFIDLDYMVYMFKYDSTHGRFHGEVKAEGGKLVIDGHKITVFHERDPANIKWGDAGATYVVESTGVFTTIEKASTHLKGGAKRVVISAPSADAPMFVMGVNHEKYENSLKVVSNASCTTNCLAPLAKVIHDNYHIIEGLMSTVHAVTATQKTVDGPSGKLWRDGRGASQNIIPASTGAAKAVGKVIPELNGKITGMAFRVPTPNVSVVDLTVRLEKPASYDAIKKVVKAAADGPMKGILGYTEQQVVSSDFNGDTHSSIFDAGAGIALNDHFVKLVTWYDNEFGYSNRVIDLMAHMATKE;SERVICE=plain;SET_DEFAULTS.x=9;SET_DEFAULTS.y=5;SHOW_OVERVIEW=on;WORD_SIZE=3;END_OF_HTTPGET=Yes).

**Search parameters**

| **MS data file:** | H:\MBA1-G6\10_BA10_01_1642.mgf |
| --- | --- |
| **Enzyme:** | Trypsin: cuts C-term side of KR unless next residue is P. |
| **Fixed modifications:** | [Carbamidomethyl (C)](http://fun-gen1.ibls.gla.ac.uk/mascot/cgi/client.pl?modification;file=..%2Fdata%2F20110930%2FF112341.dat;mod_name=Carbamidomethyl%20%28C%29) |
| **Variable modifications:** | [Oxidation (M)](http://fun-gen1.ibls.gla.ac.uk/mascot/cgi/client.pl?modification;file=..%2Fdata%2F20110930%2FF112341.dat;mod_name=Oxidation%20%28M%29) |

**Protein sequence coverage: 36%**

Matched peptides shown in ***bold red***.

| **1** | MVKVGVNGFG | RIGRLVTRAA | FHSKKGVEIV | AINDPFIDLD | YMVYMFKYDS |
| --- | --- | --- | --- | --- | --- |
| **51** | THGRFHGEVK | AEGGKLVIDG | HK**ITVFHERD** | **PANIK**WGDAG | ATYVVESTGV |
| **101** | FTTIEKASTH | LKGGAK**RVVI** | **SAPSADAPMF** | **VMGVNHEK**YE | NSLK**VVSNAS** |
| **151** | **CTTNCLAPLA** | **KVIHDNYHII** | **EGLMSTVHAV** | **TATQK**TVDGP | SGKLWRDGR**G** |
| **201** | **ASQNIIPAST** | **GAAK**AVGKVI | PELNGK**ITGM** | **AFR**VPTPNVS | VVDLTVR**LEK** |
| **251** | **PASYDAIKK**V | VKAAADGPMK | GILGYTEQQV | VSSDFNGDTH | SSIFDAGAGI |
| **301** | ALNDHFVKLV | TWYDNEFGYS | NR**VIDLMAHM** | **ATK**E |  |

Bottom of Form

**SPOT # 623**

**Protein View: Q70I40_ONCMY**

**Triosephosphate isomerase (Fragment).- Oncorhynchus mykiss (Rainbow trout) (Salmo gairdneri).**

Top of Form

| **Database:** | MSDB |
| --- | --- |
| **Score:** | 305 |
| **Nominal mass (M_r_):** | 22759 |
| **Calculated pI:** | 6.59 |
| **Taxonomy:** | [Oncorhynchus mykiss](http://www.ncbi.nlm.nih.gov/Taxonomy/Browser/wwwtax.cgi?lvl=0&id=8022) |

This protein sequence matches the following other entries:

- CAE45565 from [Oncorhynchus mykiss](http://www.ncbi.nlm.nih.gov/Taxonomy/Browser/wwwtax.cgi?lvl=0&id=8022)

Sequence similarity is available as [an NCBI BLAST search of Q70I40_ONCMY against nr](http://www.ncbi.nlm.nih.gov/blast/Blast.cgi?ALIGNMENTS=50;ALIGNMENT_VIEW=Pairwise;AUTO_FORMAT=Semiauto;CDD_SEARCH=on;CLIENT=web;COMPOSITION_BASED_STATISTICS=on;DATABASE=nr;DESCRIPTIONS=100;ENTREZ_QUERY=%28none%29;EXPECT=10;FILTER=L;FORMAT_BLOCK_ON_RESPAGE=None;FORMAT_OBJECT=Alignment;FORMAT_TYPE=HTML;GAPCOSTS=11%201;I_THRESH=0.001;LAYOUT=TwoWindows;MATRIX_NAME=BLOSUM62;NCBI_GI=on;PAGE=Proteins;PROGRAM=blastp;QUERY=GDKASLGELIKTLNSAKLDPNTEVVCGAPSIYLEFARAKLDPKIGVAAQNCYKVKGGAFTGEISPAMIKDVGVHWVILGHSERRWVFGETDELIGQKCAHALENGLGVIACIGEKLDEREAGITEKVINAQTKHFADNIKDWSKVVLAYEPVWAIGTGKTASPAQAQDVHDKLRQWVKANVSEAVANSVRIIYGGSVTGGTCKELGGMKD;SERVICE=plain;SET_DEFAULTS.x=9;SET_DEFAULTS.y=5;SHOW_OVERVIEW=on;WORD_SIZE=3;END_OF_HTTPGET=Yes).

**Search parameters**

| **MS data file:** | H:\MBA1-G6\11_BA11_01_1643.mgf |
| --- | --- |
| **Enzyme:** | Trypsin: cuts C-term side of KR unless next residue is P. |
| **Fixed modifications:** | [Carbamidomethyl (C)](http://fun-gen1.ibls.gla.ac.uk/mascot/cgi/client.pl?modification;file=..%2Fdata%2F20110930%2FF112342.dat;mod_name=Carbamidomethyl%20%28C%29) |
| **Variable modifications:** | [Oxidation (M)](http://fun-gen1.ibls.gla.ac.uk/mascot/cgi/client.pl?modification;file=..%2Fdata%2F20110930%2FF112342.dat;mod_name=Oxidation%20%28M%29) |

**Protein sequence coverage: 49%**

Matched peptides shown in ***bold red***.

| **1** | GDK**ASLGELI** | **K**TLNSAK**LDP** | **NTEVVCGAPS** | **IYLEFAR**AKL | DPKIGVAAQN |
| --- | --- | --- | --- | --- | --- |
| **51** | CYKVK**GGAFT** | **GEISPAMIK**D | VGVHWVILGH | SERRWVFGET | DELIGQKCAH |
| **101** | ALENGLGVIA | CIGEKLDERE | AGITEKVINA | QTK**HFADNIK** | DWSK**VVLAYE** |
| **151** | **PVWAIGTGKT** | **ASPAQAQDVH** | **DKLR**QWVK**AN** | **VSEAVANSVR** | **IIYGGSVTGG** |
| **201** | **TCK**ELGGMKD |  |  |  |  |

**Protein View: AAG18369**

**Immunoglobulin light chain precursor.- Salmo salar (Atlantic salmon).**

Top of Form

| **Database:** | MSDB |
| --- | --- |
| **Score:** | 109 |
| **Nominal mass (M_r_):** | 26519 |
| **Calculated pI:** | 6.29 |
| **Taxonomy:** | [Salmo salar](http://www.ncbi.nlm.nih.gov/Taxonomy/Browser/wwwtax.cgi?lvl=0&id=8030) |

Sequence similarity is available as [an NCBI BLAST search of AAG18369 against nr](http://www.ncbi.nlm.nih.gov/blast/Blast.cgi?ALIGNMENTS=50;ALIGNMENT_VIEW=Pairwise;AUTO_FORMAT=Semiauto;CDD_SEARCH=on;CLIENT=web;COMPOSITION_BASED_STATISTICS=on;DATABASE=nr;DESCRIPTIONS=100;ENTREZ_QUERY=%28none%29;EXPECT=10;FILTER=L;FORMAT_BLOCK_ON_RESPAGE=None;FORMAT_OBJECT=Alignment;FORMAT_TYPE=HTML;GAPCOSTS=11%201;I_THRESH=0.001;LAYOUT=TwoWindows;MATRIX_NAME=BLOSUM62;NCBI_GI=on;PAGE=Proteins;PROGRAM=blastp;QUERY=MTFIMSFVWILMSLIHESRGQVTVTQTPAVKAVLTGQTVPLNCKTSSDVYQAGTSSPRLAWYQQKPGEAPKLLIYYATTLQSGTPSRFSGSGTHSDFTLTISGVQAEDAGDYYCQSFHYPNSKYVYTFGSATRLDVGSNSAPTLTVLPPSSEELSSTTTATLMCLANKGFPSDWTMSWKVDGNSKKQEASPGVLEKDGLYSWSSTLTLTAQEWTKAGEVTCEAQQISQTPVTKTLRRADCSG;SERVICE=plain;SET_DEFAULTS.x=9;SET_DEFAULTS.y=5;SHOW_OVERVIEW=on;WORD_SIZE=3;END_OF_HTTPGET=Yes).

**Search parameters**

| **MS data file:** | H:\MBA1-G6\11_BA11_01_1643.mgf |
| --- | --- |
| **Enzyme:** | Trypsin: cuts C-term side of KR unless next residue is P. |
| **Fixed modifications:** | [Carbamidomethyl (C)](http://fun-gen1.ibls.gla.ac.uk/mascot/cgi/client.pl?modification;file=..%2Fdata%2F20110930%2FF112342.dat;mod_name=Carbamidomethyl%20%28C%29) |
| **Variable modifications:** | [Oxidation (M)](http://fun-gen1.ibls.gla.ac.uk/mascot/cgi/client.pl?modification;file=..%2Fdata%2F20110930%2FF112342.dat;mod_name=Oxidation%20%28M%29) |

**Protein sequence coverage: 19%**

Matched peptides shown in ***bold red***.

| **1** | MTFIMSFVWI | LMSLIHESRG | QVTVTQTPAV | KAVLTGQTVP | LNCK**TSSDVY** |
| --- | --- | --- | --- | --- | --- |
| **51** | **QAGTSSPR**LA | WYQQKPGEAP | K**LLIYYATTL** | **QSGTPSR**FSG | SGTHSDFTLT |
| **101** | ISGVQAEDAG | DYYCQSFHYP | NSKYVYTFGS | ATRLDVGSNS | APTLTVLPPS |
| **151** | SEELSSTTTA | TLMCLANKGF | PSDWTMSWKV | DGNSKKQEAS | PGVLEKDGLY |
| **201** | SWSSTLTLTA | QEWTK**AGEVT** | **CEAQQISQTP** | **VTK**TLRRADC | SG |

Bottom of Form

**SPOT # 500**

**Protein View: Q8JH72_BRARE**

**Aldolase A.- Brachydanio rerio (Zebrafish) (Danio rerio).**

Top of Form

| **Database:** | MSDB |
| --- | --- |
| **Score:** | 110 |
| **Nominal mass (M_r_):** | 40214 |
| **Calculated pI:** | 8.27 |
| **Taxonomy:** | [Danio rerio](http://www.ncbi.nlm.nih.gov/Taxonomy/Browser/wwwtax.cgi?lvl=0&id=7955) |

This protein sequence matches the following other entries:

- AAN04476 from [Danio rerio](http://www.ncbi.nlm.nih.gov/Taxonomy/Browser/wwwtax.cgi?lvl=0&id=7955)

Sequence similarity is available as [an NCBI BLAST search of Q8JH72_BRARE against nr](http://www.ncbi.nlm.nih.gov/blast/Blast.cgi?ALIGNMENTS=50;ALIGNMENT_VIEW=Pairwise;AUTO_FORMAT=Semiauto;CDD_SEARCH=on;CLIENT=web;COMPOSITION_BASED_STATISTICS=on;DATABASE=nr;DESCRIPTIONS=100;ENTREZ_QUERY=%28none%29;EXPECT=10;FILTER=L;FORMAT_BLOCK_ON_RESPAGE=None;FORMAT_OBJECT=Alignment;FORMAT_TYPE=HTML;GAPCOSTS=11%201;I_THRESH=0.001;LAYOUT=TwoWindows;MATRIX_NAME=BLOSUM62;NCBI_GI=on;PAGE=Proteins;PROGRAM=blastp;QUERY=MPHAYPFLTPEQKKELSDIAQRIVAPGKGILAADESTGSVAKRFQSINAENTEENRRLYRQLLFTADDRVKPCIGGVILFHETLYQKTDDGKVFSDYLKERGMVVGIKVDKGVVPLAGTNGETTTQGLDGLYERCAQYKKDGADFAKWRCVLKITPTTPSRLAIIENANVLARYASICQMHGLVPIVEPEILPDGDHDLKRCQYVTEKVLAAVYKALSDHHVYLEGTLLKPNMVTAGHACSQNNTPQEIAMATVTALRRTVPPAVPGVTFLSGGQSEEEATLNLNAMNQCPLHRPWALTFSYGRALQASALKAWGGKKENGKACQEEFIKRALNNSQACVGKYVSSGDKGAAAGESLFVANHAY;SERVICE=plain;SET_DEFAULTS.x=9;SET_DEFAULTS.y=5;SHOW_OVERVIEW=on;WORD_SIZE=3;END_OF_HTTPGET=Yes).

**Search parameters**

| **MS data file:** | H:\MBA1-G6\12_BA12_01_1644.mgf |
| --- | --- |
| **Enzyme:** | Trypsin: cuts C-term side of KR unless next residue is P. |
| **Fixed modifications:** | [Carbamidomethyl (C)](http://fun-gen1.ibls.gla.ac.uk/mascot/cgi/client.pl?modification;file=..%2Fdata%2F20110930%2FF112343.dat;mod_name=Carbamidomethyl%20%28C%29) |
| **Variable modifications:** | [Oxidation (M)](http://fun-gen1.ibls.gla.ac.uk/mascot/cgi/client.pl?modification;file=..%2Fdata%2F20110930%2FF112343.dat;mod_name=Oxidation%20%28M%29) |

**Protein sequence coverage: 17%**

Matched peptides shown in ***bold red***.

| **1** | MPHAYPFLTP | EQKKELSDIA | QRIVAPGK**GI** | **LAADESTGSV** | **AK**RFQSINAE |
| --- | --- | --- | --- | --- | --- |
| **51** | NTEENRRLYR | QLLFTADDRV | KPCIGGVILF | HETLYQKTDD | GKVFSDYLKE |
| **101** | RGMVVGIKVD | K**GVVPLAGTN** | **GETTTQGLDG** | **LYER**CAQYKK | DGADFAKWRC |
| **151** | VLKITPTTPS | RLAIIENANV | LAR**YASICQM** | **HGLVPIVEPE** | **ILPDGDHDLK** |
| **201** | **R**CQYVTEKVL | AAVYKALSDH | HVYLEGTLLK | PNMVTAGHAC | SQNNTPQEIA |
| **251** | MATVTALRRT | VPPAVPGVTF | LSGGQSEEEA | TLNLNAMNQC | PLHRPWALTF |
| **301** | SYGRALQASA | LKAWGGKKEN | GKACQEEFIK | RALNNSQACV | GKYVSSGDKG |
| **351** | AAAGESLFVA | NHAY |  |  |  |

**SPOT # 548**

**Protein View: ABONS1**

**serum albumin 1 precursor - Atlantic salmon**

Top of Form

| **Database:** | MSDB |
| --- | --- |
| **Score:** | 122 |
| **Nominal mass (M_r_):** | 69216 |
| **Calculated pI:** | 5.44 |
| **Taxonomy:** | [Salmo salar](http://www.ncbi.nlm.nih.gov/Taxonomy/Browser/wwwtax.cgi?lvl=0&id=8030) |

This protein sequence matches the following other entries:

- CAA36643 from [Salmo salar](http://www.ncbi.nlm.nih.gov/Taxonomy/Browser/wwwtax.cgi?lvl=0&id=8030)
- ALBU1_SALSA from [Salmo salar](http://www.ncbi.nlm.nih.gov/Taxonomy/Browser/wwwtax.cgi?lvl=0&id=8030)

Sequence similarity is available as [an NCBI BLAST search of ABONS1 against nr](http://www.ncbi.nlm.nih.gov/blast/Blast.cgi?ALIGNMENTS=50;ALIGNMENT_VIEW=Pairwise;AUTO_FORMAT=Semiauto;CDD_SEARCH=on;CLIENT=web;COMPOSITION_BASED_STATISTICS=on;DATABASE=nr;DESCRIPTIONS=100;ENTREZ_QUERY=%28none%29;EXPECT=10;FILTER=L;FORMAT_BLOCK_ON_RESPAGE=None;FORMAT_OBJECT=Alignment;FORMAT_TYPE=HTML;GAPCOSTS=11%201;I_THRESH=0.001;LAYOUT=TwoWindows;MATRIX_NAME=BLOSUM62;NCBI_GI=on;PAGE=Proteins;PROGRAM=blastp;QUERY=MQWLSVCSLLVLLSVLSRSQAQNQICTIFTEAKEDGFKSLILVGLAQNLPDSTLGDLVPLIAEALAMGVKCCSDTPPEDCERDVADLFQSAVCSSETLVEKNDLKMCCEKTAAERTHCFVDHKAKIPRDLSLKAELPAADQCEDFKKDHKAFVGRFIFKFSKSNPMLPPHVVLAIAKGYGEVLTTCCGEAEAQTCFDTKKATFQHAVMKRVAELRSLCIVHKKYGDRVVKAKKLVQYSQKMPQASFQEMGGMVDKIVATVAPCCSGDMVTCMKERKTLVDEVCADESVLSRAAGLSACCKEDAVHRGSCVEAMKPDPKPDGLSEHYDIHADIAAVCQTFTKTPDVAMGKLVYEISVRHPESSQQVILRFAKEAEQALLQCCDMEDHAECVKTALAGSDIDKKITDETDYYKKMCAAEAAVSDDSFEKSMMVYYTRIMPQASFDQLHMVSETVHDVLHACCKDEQGHFVLPCAEEKLTDAIDATCDDYDPSSINPHIAHCCNQSYSMRRHCILAIQPDTEFTPPELDASSFHMGPELCTKDSKDLLLSGKKLLYGVVRHKTTITEDHLKTISTKYHTMKEKCCAAEDQAACFTEEAPKLVSESAELVKV;SERVICE=plain;SET_DEFAULTS.x=9;SET_DEFAULTS.y=5;SHOW_OVERVIEW=on;WORD_SIZE=3;END_OF_HTTPGET=Yes).

**Search parameters**

| **MS data file:** | H:\MBA1-G6\13_BB1_01_1645.mgf |
| --- | --- |
| **Enzyme:** | Trypsin: cuts C-term side of KR unless next residue is P. |
| **Fixed modifications:** | [Carbamidomethyl (C)](http://fun-gen1.ibls.gla.ac.uk/mascot/cgi/client.pl?modification;file=..%2Fdata%2F20110930%2FF112344.dat;mod_name=Carbamidomethyl%20%28C%29) |
| **Variable modifications:** | [Oxidation (M)](http://fun-gen1.ibls.gla.ac.uk/mascot/cgi/client.pl?modification;file=..%2Fdata%2F20110930%2FF112344.dat;mod_name=Oxidation%20%28M%29) |

**Protein sequence coverage: 7%**

Matched peptides shown in ***bold red***.

| **1** | MQWLSVCSLL | VLLSVLSRSQ | AQNQICTIFT | EAKEDGFKSL | ILVGLAQNLP |
| --- | --- | --- | --- | --- | --- |
| **51** | DSTLGDLVPL | IAEALAMGVK | CCSDTPPEDC | ER**DVADLFQS** | **AVCSSETLVE** |
| **101** | **K**NDLKMCCEK | TAAERTHCFV | DHKAKIPRDL | SLK**AELPAAD** | **QCEDFKK**DHK |
| **151** | AFVGRFIFKF | SKSNPMLPPH | VVLAIAKGYG | EVLTTCCGEA | EAQTCFDTK**K** |
| **201** | **ATFQHAVMK**R | VAELRSLCIV | HKKYGDRVVK | AKKLVQYSQK | MPQASFQEMG |
| **251** | GMVDKIVATV | APCCSGDMVT | CMKERKTLVD | EVCADESVLS | RAAGLSACCK |
| **301** | EDAVHRGSCV | EAMKPDPKPD | GLSEHYDIHA | DIAAVCQTFT | KTPDVAMGKL |
| **351** | VYEISVRHPE | SSQQVILRFA | KEAEQALLQC | CDMEDHAECV | KTALAGSDID |
| **401** | KKITDETDYY | KKMCAAEAAV | SDDSFEKSMM | VYYTRIMPQA | SFDQLHMVSE |
| **451** | TVHDVLHACC | KDEQGHFVLP | CAEEKLTDAI | DATCDDYDPS | SINPHIAHCC |
| **501** | NQSYSMRRHC | ILAIQPDTEF | TPPELDASSF | HMGPELCTKD | SKDLLLSGKK |
| **551** | LLYGVVRHKT | TITEDHLKTI | STKYHTMKEK | CCAAEDQAAC | FTEEAPKLVS |
| **601** | ESAELVKV |  |  |  |  |

**SPOT # 493**

**Protein View: Q90Z48_ONCMY**

**Glyceraldehyde phosphate dehydrogenase.- Oncorhynchus mykiss (Rainbow trout) (Salmo gairdneri).**

Top of Form

| **Database:** | MSDB |
| --- | --- |
| **Score:** | 147 |
| **Nominal mass (M_r_):** | 36422 |
| **Calculated pI:** | 7.23 |
| **Taxonomy:** | [Oncorhynchus mykiss](http://www.ncbi.nlm.nih.gov/Taxonomy/Browser/wwwtax.cgi?lvl=0&id=8022) |

This protein sequence matches the following other entries:

- AAK49985 from [Oncorhynchus mykiss](http://www.ncbi.nlm.nih.gov/Taxonomy/Browser/wwwtax.cgi?lvl=0&id=8022)

Sequence similarity is available as [an NCBI BLAST search of Q90Z48_ONCMY against nr](http://www.ncbi.nlm.nih.gov/blast/Blast.cgi?ALIGNMENTS=50;ALIGNMENT_VIEW=Pairwise;AUTO_FORMAT=Semiauto;CDD_SEARCH=on;CLIENT=web;COMPOSITION_BASED_STATISTICS=on;DATABASE=nr;DESCRIPTIONS=100;ENTREZ_QUERY=%28none%29;EXPECT=10;FILTER=L;FORMAT_BLOCK_ON_RESPAGE=None;FORMAT_OBJECT=Alignment;FORMAT_TYPE=HTML;GAPCOSTS=11%201;I_THRESH=0.001;LAYOUT=TwoWindows;MATRIX_NAME=BLOSUM62;NCBI_GI=on;PAGE=Proteins;PROGRAM=blastp;QUERY=MVKVGVNGFGRIGRLVTRAAFHSKKGVEIVAINDPFIDLDYMVYMFKYDSTHGRFHGEVKAEGGKLVIDGHKITVFHERDPANIKWGDAGATYVVESTGVFTTIEKASTHLKGGAKRVVISAPSADAPMFVMXVNHEKYENSLKVVSNASCXTNCLAPLAKVIHDNYHIIEGLMXTVXAVTATQKTEDGPSGKLWRDGRGASQNIIPASTGXRKAVGKVIPELNGXDHCMAFRVPTPNVSVVDLTVRLEKPASYDAIKKVVNXAADGPMKGILGYTEQQVVSSDFNGDTHSSIFDAGAGIALNDHFVKLVTWYDNEFGYSNRVIDLMAHMATKE;SERVICE=plain;SET_DEFAULTS.x=9;SET_DEFAULTS.y=5;SHOW_OVERVIEW=on;WORD_SIZE=3;END_OF_HTTPGET=Yes).

**Search parameters**

| **MS data file:** | H:\MBA1-G6\14_BB2_01_1646.mgf |
| --- | --- |
| **Enzyme:** | Trypsin: cuts C-term side of KR unless next residue is P. |
| **Fixed modifications:** | [Carbamidomethyl (C)](http://fun-gen1.ibls.gla.ac.uk/mascot/cgi/client.pl?modification;file=..%2Fdata%2F20110930%2FF112345.dat;mod_name=Carbamidomethyl%20%28C%29) |
| **Variable modifications:** | [Oxidation (M)](http://fun-gen1.ibls.gla.ac.uk/mascot/cgi/client.pl?modification;file=..%2Fdata%2F20110930%2FF112345.dat;mod_name=Oxidation%20%28M%29) |

**Protein sequence coverage: 37%**

Matched peptides shown in ***bold red***.

| **1** | MVKVGVNGFG | RIGRLVTRAA | FHSKKGVEIV | AINDPFIDLD | YMVYMFKYDS |
| --- | --- | --- | --- | --- | --- |
| **51** | THGRFHGEVK | AEGGKLVIDG | HK**ITVFHERD** | **PANIKWGDAG** | **ATYVVESTGV** |
| **101** | **FTTIEK**ASTH | LKGGAK**RVVI** | **SAPSADAPMF** | **VMXVNHEK**YE | NSLK**VVSNAS** |
| **151** | **CXTNCLAPLA** | **K**VIHDNYHII | EGLMXTVXAV | TATQKTEDGP | SGKLWRDGR**G** |
| **201** | **ASQNIIPAST** | **GXR**KAVGKVI | PELNGXDHCM | AFRVPTPNVS | VVDLTVR**LEK** |
| **251** | **PASYDAIKK**V | VNXAADGPMK | GILGYTEQQV | VSSDFNGDTH | SSIFDAGAGI |
| **301** | ALNDHFVK**LV** | **TWYDNEFGYS** | **NRVIDLMAHM** | **ATKE** |  |

**SPOT #477**

**Protein View: Q8JH72_BRARE**

**Aldolase A.- Brachydanio rerio (Zebrafish) (Danio rerio).**

Top of Form

| **Database:** | MSDB |
| --- | --- |
| **Score:** | 130 |
| **Nominal mass (M_r_):** | 40214 |
| **Calculated pI:** | 8.27 |
| **Taxonomy:** | [Danio rerio](http://www.ncbi.nlm.nih.gov/Taxonomy/Browser/wwwtax.cgi?lvl=0&id=7955) |

This protein sequence matches the following other entries:

- AAN04476 from [Danio rerio](http://www.ncbi.nlm.nih.gov/Taxonomy/Browser/wwwtax.cgi?lvl=0&id=7955)

Sequence similarity is available as [an NCBI BLAST search of Q8JH72_BRARE against nr](http://www.ncbi.nlm.nih.gov/blast/Blast.cgi?ALIGNMENTS=50;ALIGNMENT_VIEW=Pairwise;AUTO_FORMAT=Semiauto;CDD_SEARCH=on;CLIENT=web;COMPOSITION_BASED_STATISTICS=on;DATABASE=nr;DESCRIPTIONS=100;ENTREZ_QUERY=%28none%29;EXPECT=10;FILTER=L;FORMAT_BLOCK_ON_RESPAGE=None;FORMAT_OBJECT=Alignment;FORMAT_TYPE=HTML;GAPCOSTS=11%201;I_THRESH=0.001;LAYOUT=TwoWindows;MATRIX_NAME=BLOSUM62;NCBI_GI=on;PAGE=Proteins;PROGRAM=blastp;QUERY=MPHAYPFLTPEQKKELSDIAQRIVAPGKGILAADESTGSVAKRFQSINAENTEENRRLYRQLLFTADDRVKPCIGGVILFHETLYQKTDDGKVFSDYLKERGMVVGIKVDKGVVPLAGTNGETTTQGLDGLYERCAQYKKDGADFAKWRCVLKITPTTPSRLAIIENANVLARYASICQMHGLVPIVEPEILPDGDHDLKRCQYVTEKVLAAVYKALSDHHVYLEGTLLKPNMVTAGHACSQNNTPQEIAMATVTALRRTVPPAVPGVTFLSGGQSEEEATLNLNAMNQCPLHRPWALTFSYGRALQASALKAWGGKKENGKACQEEFIKRALNNSQACVGKYVSSGDKGAAAGESLFVANHAY;SERVICE=plain;SET_DEFAULTS.x=9;SET_DEFAULTS.y=5;SHOW_OVERVIEW=on;WORD_SIZE=3;END_OF_HTTPGET=Yes).

**Search parameters**

| **MS data file:** | H:\MBA1-G6\15_BB3_01_1647.mgf |
| --- | --- |
| **Enzyme:** | Trypsin: cuts C-term side of KR unless next residue is P. |
| **Fixed modifications:** | [Carbamidomethyl (C)](http://fun-gen1.ibls.gla.ac.uk/mascot/cgi/client.pl?modification;file=..%2Fdata%2F20110930%2FF112346.dat;mod_name=Carbamidomethyl%20%28C%29) |
| **Variable modifications:** | [Oxidation (M)](http://fun-gen1.ibls.gla.ac.uk/mascot/cgi/client.pl?modification;file=..%2Fdata%2F20110930%2FF112346.dat;mod_name=Oxidation%20%28M%29) |

**Protein sequence coverage: 18%**

Matched peptides shown in ***bold red***.

| **1** | MPHAYPFLTP | EQKKELSDIA | QRIVAPGK**GI** | **LAADESTGSV** | **AKR**FQSINAE |
| --- | --- | --- | --- | --- | --- |
| **51** | NTEENRRLYR | QLLFTADDRV | KPCIGGVILF | HETLYQKTDD | GKVFSDYLKE |
| **101** | RGMVVGIKVD | K**GVVPLAGTN** | **GETTTQGLDG** | **LYER**CAQYKK | DGADFAKWRC |
| **151** | VLKITPTTPS | RLAIIENANV | LAR**YASICQM** | **HGLVPIVEPE** | **ILPDGDHDLK** |
| **201** | **R**CQYVTEKVL | AAVYKALSDH | HVYLEGTLLK | PNMVTAGHAC | SQNNTPQEIA |
| **251** | MATVTALRRT | VPPAVPGVTF | LSGGQSEEEA | TLNLNAMNQC | PLHRPWALTF |
| **301** | SYGRALQASA | LKAWGGKKEN | GKACQEEFIK | RALNNSQACV | GKYVSSGDKG |
| **351** | AAAGESLFVA | NHAY |  |  |  |

**SPOT # 687**

**Protein View: TRF2_SALSA**

**Serotransferrin II precursor (Siderophilin II) (STF II).- Salmo salar (Atlantic salmon).**

Top of Form

| **Database:** | MSDB |
| --- | --- |
| **Score:** | 199 |
| **Nominal mass (M_r_):** | 76497 |
| **Calculated pI:** | 7.08 |
| **Taxonomy:** | [Salmo salar](http://www.ncbi.nlm.nih.gov/Taxonomy/Browser/wwwtax.cgi?lvl=0&id=8030) |

Sequence similarity is available as [an NCBI BLAST search of TRF2_SALSA against nr](http://www.ncbi.nlm.nih.gov/blast/Blast.cgi?ALIGNMENTS=50;ALIGNMENT_VIEW=Pairwise;AUTO_FORMAT=Semiauto;CDD_SEARCH=on;CLIENT=web;COMPOSITION_BASED_STATISTICS=on;DATABASE=nr;DESCRIPTIONS=100;ENTREZ_QUERY=%28none%29;EXPECT=10;FILTER=L;FORMAT_BLOCK_ON_RESPAGE=None;FORMAT_OBJECT=Alignment;FORMAT_TYPE=HTML;GAPCOSTS=11%201;I_THRESH=0.001;LAYOUT=TwoWindows;MATRIX_NAME=BLOSUM62;NCBI_GI=on;PAGE=Proteins;PROGRAM=blastp;QUERY=MKLLLLSALLGCLATAYAAPAEGIVKWCVKSEQELRKCHDLAAKVAEFSCVRKDGSFECIQAIKGGEADAITLDGGDIYTAGLTNYGLQPIIAEDYGEDSDTCYYAVAVAKKGTAFGFKTLRGKKSCHTGLGKSAGWNIPIGTLVTESQIRWAGIEDRPVESAVSDFFNASCAPGATMGSKLCQLCKGDCSRSHKEPYYDYAGAFQCLKDGAGDVAFIKPLAVPAAEKASYELLCKDGTRASIDSYKTCHLARVPAHAVVSRKDPELANRIYNKLVAVKDFNLFSSDGYAAKNLMFKDSAQKLVQLPTTTDSFLYLGAEYMSTIRSLKKSQATGASSRAIKWCAVGHAEKGKCDTWTINSFADGESKISCQDAPTVEECIKKIMRKEADAIAVDGGEVYTAGKCGLVPVMVEQYDADLCSAPGEASSYYAVAVAKKGSGLTWKTLKGKRSCHTGLGRTAGWNIPMGLIHQETNDCDFTKYFSKGCAPGSEVGSPFCAQCKGSGKARGGDEDRCKARSEEQYYGYTGAFRCLVEDAGDVAFIKHTIVPESTDGNGPDWAKDLKSSDFELLCQDGTTQPVTKFSECHLAKVPAHAVITRPETRGDVVSILLELQAKFGSSGSDSSFRMFQSSVEKNLLFKDSTKCLQEIPKGTKYQDFLGKEYMIAMQSLRKCSDSTSDLEKACTFHSCQQKE;SERVICE=plain;SET_DEFAULTS.x=9;SET_DEFAULTS.y=5;SHOW_OVERVIEW=on;WORD_SIZE=3;END_OF_HTTPGET=Yes).

**Search parameters**

| **MS data file:** | H:\MBA1-G6\16_BB4_01_1648.mgf |
| --- | --- |
| **Enzyme:** | Trypsin: cuts C-term side of KR unless next residue is P. |
| **Fixed modifications:** | [Carbamidomethyl (C)](http://fun-gen1.ibls.gla.ac.uk/mascot/cgi/client.pl?modification;file=..%2Fdata%2F20110930%2FF112347.dat;mod_name=Carbamidomethyl%20%28C%29) |
| **Variable modifications:** | [Oxidation (M)](http://fun-gen1.ibls.gla.ac.uk/mascot/cgi/client.pl?modification;file=..%2Fdata%2F20110930%2FF112347.dat;mod_name=Oxidation%20%28M%29) |

**Protein sequence coverage: 12%**

Matched peptides shown in ***bold red***.

| **1** | MKLLLLSALL | GCLATAYAAP | AEGIVKWCVK | SEQELRKCHD | LAAKVAEFSC |
| --- | --- | --- | --- | --- | --- |
| **51** | VRKDGSFECI | QAIKGGEADA | ITLDGGDIYT | AGLTNYGLQP | IIAEDYGEDS |
| **101** | DTCYYAVAVA | KKGTAFGFKT | LRGKKSCHTG | LGKSAGWNIP | IGTLVTESQI |
| **151** | RWAGIEDRPV | ESAVSDFFNA | SCAPGATMGS | KLCQLCKGDC | SRSHKEPYYD |
| **201** | YAGAFQCLK**D** | **GAGDVAFIKP** | **LAVPAAEK**AS | YELLCKDGTR | ASIDSYKTCH |
| **251** | LARVPAHAVV | SRKDPELANR | IYNKLVAVK**D** | **FNLFSSDGYA** | **AK**NLMFKDSA |
| **301** | QKLVQLPTTT | DSFLYLGAEY | MSTIRSLKKS | QATGASSRAI | KWCAVGHAEK |
| **351** | GK**CDTWTINS** | **FADGESK**ISC | QDAPTVEECI | KKIMR**KEADA** | **IAVDGGEVYT** |
| **401** | **AGK**CGLVPVM | VEQYDADLCS | APGEASSYYA | VAVAKKGSGL | TWKTLKGKRS |
| **451** | CHTGLGRTAG | WNIPMGLIHQ | ETNDCDFTKY | FSK**GCAPGSE** | **VGSPFCAQCK** |
| **501** | **GSGK**ARGGDE | DRCKARSEEQ | YYGYTGAFRC | LVEDAGDVAF | IKHTIVPEST |
| **551** | DGNGPDWAKD | LKSSDFELLC | QDGTTQPVTK | FSECHLAKVP | AHAVITRPET |
| **601** | RGDVVSILLE | LQAKFGSSGS | DSSFRMFQSS | VEKNLLFKDS | TKCLQEIPKG |
| **651** | TKYQDFLGKE | YMIAMQSLRK | CSDSTSDLEK | ACTFHSCQQK | E |

**SPOT # 479**

**Protein View: Q90Z48_ONCMY**

**Glyceraldehyde phosphate dehydrogenase.- Oncorhynchus mykiss (Rainbow trout) (Salmo gairdneri).**

Top of Form

| **Database:** | MSDB |
| --- | --- |
| **Score:** | 190 |
| **Nominal mass (M_r_):** | 36422 |
| **Calculated pI:** | 7.23 |
| **Taxonomy:** | [Oncorhynchus mykiss](http://www.ncbi.nlm.nih.gov/Taxonomy/Browser/wwwtax.cgi?lvl=0&id=8022) |

This protein sequence matches the following other entries:

- AAK49985 from [Oncorhynchus mykiss](http://www.ncbi.nlm.nih.gov/Taxonomy/Browser/wwwtax.cgi?lvl=0&id=8022)

Sequence similarity is available as [an NCBI BLAST search of Q90Z48_ONCMY against nr](http://www.ncbi.nlm.nih.gov/blast/Blast.cgi?ALIGNMENTS=50;ALIGNMENT_VIEW=Pairwise;AUTO_FORMAT=Semiauto;CDD_SEARCH=on;CLIENT=web;COMPOSITION_BASED_STATISTICS=on;DATABASE=nr;DESCRIPTIONS=100;ENTREZ_QUERY=%28none%29;EXPECT=10;FILTER=L;FORMAT_BLOCK_ON_RESPAGE=None;FORMAT_OBJECT=Alignment;FORMAT_TYPE=HTML;GAPCOSTS=11%201;I_THRESH=0.001;LAYOUT=TwoWindows;MATRIX_NAME=BLOSUM62;NCBI_GI=on;PAGE=Proteins;PROGRAM=blastp;QUERY=MVKVGVNGFGRIGRLVTRAAFHSKKGVEIVAINDPFIDLDYMVYMFKYDSTHGRFHGEVKAEGGKLVIDGHKITVFHERDPANIKWGDAGATYVVESTGVFTTIEKASTHLKGGAKRVVISAPSADAPMFVMXVNHEKYENSLKVVSNASCXTNCLAPLAKVIHDNYHIIEGLMXTVXAVTATQKTEDGPSGKLWRDGRGASQNIIPASTGXRKAVGKVIPELNGXDHCMAFRVPTPNVSVVDLTVRLEKPASYDAIKKVVNXAADGPMKGILGYTEQQVVSSDFNGDTHSSIFDAGAGIALNDHFVKLVTWYDNEFGYSNRVIDLMAHMATKE;SERVICE=plain;SET_DEFAULTS.x=9;SET_DEFAULTS.y=5;SHOW_OVERVIEW=on;WORD_SIZE=3;END_OF_HTTPGET=Yes).

**Search parameters**

| **MS data file:** | H:\MBA1-G6\17_BB5_01_1649.mgf |
| --- | --- |
| **Enzyme:** | Trypsin: cuts C-term side of KR unless next residue is P. |
| **Fixed modifications:** | [Carbamidomethyl (C)](http://fun-gen1.ibls.gla.ac.uk/mascot/cgi/client.pl?modification;file=..%2Fdata%2F20110930%2FF112270.dat;mod_name=Carbamidomethyl%20%28C%29) |
| **Variable modifications:** | [Oxidation (M)](http://fun-gen1.ibls.gla.ac.uk/mascot/cgi/client.pl?modification;file=..%2Fdata%2F20110930%2FF112270.dat;mod_name=Oxidation%20%28M%29) |

**Protein sequence coverage: 34%**

Matched peptides shown in ***bold red***.

| **1** | MVKVGVNGFG | RIGRLVTRAA | FHSK**KGVEIV** | **AINDPFIDLD** | **YMVYMFK**YDS |
| --- | --- | --- | --- | --- | --- |
| **51** | THGRFHGEVK | AEGGKLVIDG | HK**ITVFHERD** | **PANIK**WGDAG | ATYVVESTGV |
| **101** | FTTIEKASTH | LKGGAK**RVVI** | **SAPSADAPMF** | **VMXVNHEK**YE | NSLK**VVSNAS** |
| **151** | **CXTNCLAPLA** | **K**VIHDNYHII | EGLMXTVXAV | TATQKTEDGP | SGKLWRDGR**G** |
| **201** | **ASQNIIPAST** | **GXR**KAVGKVI | PELNGXDHCM | AFR**VPTPNVS** | **VVDLTVRLEK** |
| **251** | **PASYDAIKK**V | VNXAADGPMK | GILGYTEQQV | VSSDFNGDTH | SSIFDAGAGI |
| **301** | ALNDHFVKLV | TWYDNEFGYS | NRVIDLMAHM | ATKE |  |

**SPOT # 440**

**Protein View: Q9DDG6_SALTR**

**Alpha-2 enolase-1 (Fragment).- Salmo trutta (Brown trout).**

Top of Form

| **Database:** | MSDB |
| --- | --- |
| **Score:** | 164 |
| **Nominal mass (M_r_):** | 39667 |
| **Calculated pI:** | 5.78 |
| **Taxonomy:** | [Salmo trutta](http://www.ncbi.nlm.nih.gov/Taxonomy/Browser/wwwtax.cgi?lvl=0&id=8032) |

This protein sequence matches the following other entries:

- AAG16311 from [Salmo trutta](http://www.ncbi.nlm.nih.gov/Taxonomy/Browser/wwwtax.cgi?lvl=0&id=8032)

Sequence similarity is available as [an NCBI BLAST search of Q9DDG6_SALTR against nr](http://www.ncbi.nlm.nih.gov/blast/Blast.cgi?ALIGNMENTS=50;ALIGNMENT_VIEW=Pairwise;AUTO_FORMAT=Semiauto;CDD_SEARCH=on;CLIENT=web;COMPOSITION_BASED_STATISTICS=on;DATABASE=nr;DESCRIPTIONS=100;ENTREZ_QUERY=%28none%29;EXPECT=10;FILTER=L;FORMAT_BLOCK_ON_RESPAGE=None;FORMAT_OBJECT=Alignment;FORMAT_TYPE=HTML;GAPCOSTS=11%201;I_THRESH=0.001;LAYOUT=TwoWindows;MATRIX_NAME=BLOSUM62;NCBI_GI=on;PAGE=Proteins;PROGRAM=blastp;QUERY=TKKGLFRAAVPSGASTGIYEALELRDNDKTRYLGKGVKRAVKHINEFLAPALCNQNVNVLEQEKVDKLMLDMDGTENKSKFGANAILGVSLAVCKAGAAEKGVPLYRHIADLAGNPNXILPCPAFNVINGGSHAGNKLAMQEFMILPIGASNFHEAMRIGAEVYHNLKNVIKAKYGKDATNVGDEGGFAPNILENNEALELLKSAIEKAGYPDKIIIGMDVAASEFYKAGKYDLDFKSPDDPARYITXDQLGDLYKSFIKGYPVQSIEDPFDQDDWAAWSKFTAAVDIQVVGDDLTVTNPKRIQQAVEKKACNCLLLKVNQIGSVTESIKACKLAQSNGWGVMVSHRSGETEDTFIADLVVGL;SERVICE=plain;SET_DEFAULTS.x=9;SET_DEFAULTS.y=5;SHOW_OVERVIEW=on;WORD_SIZE=3;END_OF_HTTPGET=Yes).

**Search parameters**

| **MS data file:** | H:\MBA1-G6\18_BB6_01_1650.mgf |
| --- | --- |
| **Enzyme:** | Trypsin: cuts C-term side of KR unless next residue is P. |
| **Fixed modifications:** | [Carbamidomethyl (C)](http://fun-gen1.ibls.gla.ac.uk/mascot/cgi/client.pl?modification;file=..%2Fdata%2F20110930%2FF112271.dat;mod_name=Carbamidomethyl%20%28C%29) |
| **Variable modifications:** | [Oxidation (M)](http://fun-gen1.ibls.gla.ac.uk/mascot/cgi/client.pl?modification;file=..%2Fdata%2F20110930%2FF112271.dat;mod_name=Oxidation%20%28M%29) |

**Protein sequence coverage: 9%**

Matched peptides shown in ***bold red***.

| **1** | TKKGLFRAAV | PSGASTGIYE | ALELRDNDKT | RYLGKGVKRA | VKHINEFLAP |
| --- | --- | --- | --- | --- | --- |
| **51** | ALCNQNVNVL | EQEKVDKLML | DMDGTENKSK | FGANAILGVS | LAVCKAGAAE |
| **101** | KGVPLYRHIA | DLAGNPNXIL | PCPAFNVING | GSHAGNKLAM | QEFMILPIGA |
| **151** | SNFHEAMR**IG** | **AEVYHNLK**NV | IKAKYGKDAT | NVGDEGGFAP | NILENNEALE |
| **201** | LLKSAIEKAG | YPDK**IIIGMD** | **VAASEFYK**AG | KYDLDFKSPD | DPAR**YITXDQ** |
| **251** | **LGDLYK**SFIK | GYPVQSIEDP | FDQDDWAAWS | KFTAAVDIQV | VGDDLTVTNP |
| **301** | KRIQQAVEKK | ACNCLLLKVN | QIGSVTESIK | ACKLAQSNGW | GVMVSHRSGE |
| **351** | TEDTFIADLV | VGL |  |  |  |

Bottom of Form

**SPOT # 602**

**No significant matches**

**SPOT # 668**

**Protein View: JH0472**

**apolipoprotein A-I precursor - Atlantic salmon**

Top of Form

| **Database:** | MSDB |
| --- | --- |
| **Score:** | 325 |
| **Nominal mass (M_r_):** | 29557 |
| **Calculated pI:** | 8.48 |
| **Taxonomy:** | [Salmo salar](http://www.ncbi.nlm.nih.gov/Taxonomy/Browser/wwwtax.cgi?lvl=0&id=8030) |

Sequence similarity is available as [an NCBI BLAST search of JH0472 against nr](http://www.ncbi.nlm.nih.gov/blast/Blast.cgi?ALIGNMENTS=50;ALIGNMENT_VIEW=Pairwise;AUTO_FORMAT=Semiauto;CDD_SEARCH=on;CLIENT=web;COMPOSITION_BASED_STATISTICS=on;DATABASE=nr;DESCRIPTIONS=100;ENTREZ_QUERY=%28none%29;EXPECT=10;FILTER=L;FORMAT_BLOCK_ON_RESPAGE=None;FORMAT_OBJECT=Alignment;FORMAT_TYPE=HTML;GAPCOSTS=11%201;I_THRESH=0.001;LAYOUT=TwoWindows;MATRIX_NAME=BLOSUM62;NCBI_GI=on;PAGE=Proteins;PROGRAM=blastp;QUERY=MKFLVLALTILLAAGTQAFPMQADAPSQLEHVKAALNMYIAQVKLTAQRSIDLLDDTEYKEYKMQLSQSLDNLQQFADSTSKSWPPTPRSSAPSCDATATVRAEVMKDVEDVRTQLEPKRAELREVLNKHIDEYRKKLEPLIKEHIELRRTEMDAFRAKIEPVVEEMRAKVAVNVEETKTKLMPIVEIVRAKLTERLEELRTLAAPYAEEYKEQMFKAVGEVREKVAPLSEDFKARWAPPPRRPSKSSWLSTRPSARP;SERVICE=plain;SET_DEFAULTS.x=9;SET_DEFAULTS.y=5;SHOW_OVERVIEW=on;WORD_SIZE=3;END_OF_HTTPGET=Yes).

**Search parameters**

| **MS data file:** | H:\MBA1-G6\20_BB8_01_1652.mgf |
| --- | --- |
| **Enzyme:** | Trypsin: cuts C-term side of KR unless next residue is P. |
| **Fixed modifications:** | [Carbamidomethyl (C)](http://fun-gen1.ibls.gla.ac.uk/mascot/cgi/client.pl?modification;file=..%2Fdata%2F20110930%2FF112273.dat;mod_name=Carbamidomethyl%20%28C%29) |
| **Variable modifications:** | [Oxidation (M)](http://fun-gen1.ibls.gla.ac.uk/mascot/cgi/client.pl?modification;file=..%2Fdata%2F20110930%2FF112273.dat;mod_name=Oxidation%20%28M%29) |

**Protein sequence coverage: 34%**

Matched peptides shown in ***bold red***.

| **1** | MKFLVLALTI | LLAAGTQAFP | MQADAPSQLE | HVK**AALNMYI** | **AQVK**LTAQR**S** |
| --- | --- | --- | --- | --- | --- |
| **51** | **IDLLDDTEYK** | **EYKMQLSQSL** | **DNLQQFADST** | **SK**SWPPTPRS | SAPSCDATAT |
| **101** | VR**AEVMKDVE** | **DVR**TQLEPKR | AELREVLNKH | IDEYRK**KLEP** | **LIK**EHIELRR |
| **151** | TEMDAFR**AKI** | **EPVVEEMR**AK | VAVNVEETKT | KLMPIVEIVR | AKLTERLEEL |
| **201** | R**TLAAPYAEE** | **YKEQMFK**AVG | EVREKVAPLS | EDFKARWAPP | PRRPSKSSWL |
| **251** | STRPSARP |  |  |  |  |

**SPOT # 342**

**Protein View: ABONS2**

**serum albumin 2 precursor - Atlantic salmon**

Top of Form

| **Database:** | MSDB |
| --- | --- |
| **Score:** | 666 |
| **Nominal mass (M_r_):** | 69124 |
| **Calculated pI:** | 5.44 |
| **Taxonomy:** | [Salmo salar](http://www.ncbi.nlm.nih.gov/Taxonomy/Browser/wwwtax.cgi?lvl=0&id=8030) |

This protein sequence matches the following other entries:

- CAA43187 from [Salmo salar](http://www.ncbi.nlm.nih.gov/Taxonomy/Browser/wwwtax.cgi?lvl=0&id=8030)
- ALBU2_SALSA from [Salmo salar](http://www.ncbi.nlm.nih.gov/Taxonomy/Browser/wwwtax.cgi?lvl=0&id=8030)

Sequence similarity is available as [an NCBI BLAST search of ABONS2 against nr](http://www.ncbi.nlm.nih.gov/blast/Blast.cgi?ALIGNMENTS=50;ALIGNMENT_VIEW=Pairwise;AUTO_FORMAT=Semiauto;CDD_SEARCH=on;CLIENT=web;COMPOSITION_BASED_STATISTICS=on;DATABASE=nr;DESCRIPTIONS=100;ENTREZ_QUERY=%28none%29;EXPECT=10;FILTER=L;FORMAT_BLOCK_ON_RESPAGE=None;FORMAT_OBJECT=Alignment;FORMAT_TYPE=HTML;GAPCOSTS=11%201;I_THRESH=0.001;LAYOUT=TwoWindows;MATRIX_NAME=BLOSUM62;NCBI_GI=on;PAGE=Proteins;PROGRAM=blastp;QUERY=MQWLSVCSLLVLLSVLSRSQAQNQICTIFTEAKEDGFKSLILVGLAQNLPDSTLGDLVPLIAEALAMGVKCCSDTPPEDCERDVADLFQSAVCSSETLVEKNDLKMCCEKTAAERTHCFVDHKAKIPRDLSLKAELPAADQCEDFKKDHKAFVGRFIFKFSKSNPMLPPHVVLAIAKGYGEVLTTCCGEAEAQTCFDTKKATFQHAIAKRVAELKSLCIVHKKYGDRVVKAKKLVQYSQKMPQASFQEMAGMVDKIVATVAPCCSGDMVTCMKERKTLVDEVCADESVLSRAAGLSACCKEDAVHRGSCVEAMKPDPKPDGLSEHYDVHADIAAVCQTFTKTPDVAMGKLVYEISVRHPESSQQVILRFAKEAEQALLQCCDMEDHAECVKTALAGSDIDKKITDETDYYKKMCAAEAAVSDDNFEKSMMVYYTRIMPQASFDQLHMVSETVHDVLHACCKDEPGHFVLPCAEEKLTDAIDATCDDYDPSSINPHIAHCCNQSYSMRRHCILAIQPDTEFTPPELDASSFHMGPELCTKDSKDLLLSGKKLLYGVVRHKTTITEDHLKTISTKYHTMKDKCCAAEDQAACFTEEAPKLVSESAELVKV;SERVICE=plain;SET_DEFAULTS.x=9;SET_DEFAULTS.y=5;SHOW_OVERVIEW=on;WORD_SIZE=3;END_OF_HTTPGET=Yes).

**Search parameters**

| **MS data file:** | H:\MBA1-G6\21_BB9_01_1653.mgf |
| --- | --- |
| **Enzyme:** | Trypsin: cuts C-term side of KR unless next residue is P. |
| **Fixed modifications:** | [Carbamidomethyl (C)](http://fun-gen1.ibls.gla.ac.uk/mascot/cgi/client.pl?modification;file=..%2Fdata%2F20110930%2FF112274.dat;mod_name=Carbamidomethyl%20%28C%29) |
| **Variable modifications:** | [Oxidation (M)](http://fun-gen1.ibls.gla.ac.uk/mascot/cgi/client.pl?modification;file=..%2Fdata%2F20110930%2FF112274.dat;mod_name=Oxidation%20%28M%29) |

**Protein sequence coverage: 31%**

Matched peptides shown in ***bold red***.

| **1** | MQWLSVCSLL | VLLSVLSRSQ | AQNQICTIFT | EAKEDGFKSL | ILVGLAQNLP |
| --- | --- | --- | --- | --- | --- |
| **51** | DSTLGDLVPL | IAEALAMGVK | CCSDTPPEDC | ERDVADLFQS | AVCSSETLVE |
| **101** | KNDLKMCCEK | TAAERTHCFV | DHKAKIPRDL | SLKAELPAAD | QCEDFKKDHK |
| **151** | AFVGRFIFKF | SKSNPMLPPH | VVLAIAKGYG | EVLTTCCGEA | EAQTCFDTKK |
| **201** | ATFQHAIAKR | VAELK**SLCIV** | **HK**KYGDRVVK | AKK**LVQYSQK** | **MPQASFQEMA** |
| **251** | **GMVDKIVATV** | **APCCSGDMVT** | **CMK**ER**KTLVD** | **EVCADESVLS** | **R**AAGLSACCK |
| **301** | EDAVHRGSCV | EAMKPDPKPD | GLSEHYDVHA | DIAAVCQTFT | KTPDVAMGKL |
| **351** | VYEISVR**HPE** | **SSQQVILR**FA | K**EAEQALLQC** | **CDMEDHAECV** | **KTALAGSDID** |
| **401** | **KKITDETDYY** | **KKMCAAEAAV** | **SDDNFEK**SMM | VYYTRIMPQA | SFDQLHMVSE |
| **451** | TVHDVLHACC | K**DEPGHFVLP** | **CAEEK**LTDAI | DATCDDYDPS | SINPHIAHCC |
| **501** | NQSYSMRRHC | ILAIQPDTEF | TPPELDASSF | HMGPELCTKD | SKDLLLSGKK |
| **551** | **LLYGVVRHKT** | **TITEDHLK**TI | STKYHTMK**DK** | **CCAAEDQAAC** | **FTEEAPKLVS** |
| **601** | **ESAELVKV** |  |  |  |  |

**SPOT # 656**

**Protein View: TRF2_SALSA**

**Serotransferrin II precursor (Siderophilin II) (STF II).- Salmo salar (Atlantic salmon).**

Top of Form

| **Database:** | MSDB |
| --- | --- |
| **Score:** | 251 |
| **Nominal mass (M_r_):** | 76497 |
| **Calculated pI:** | 7.08 |
| **Taxonomy:** | [Salmo salar](http://www.ncbi.nlm.nih.gov/Taxonomy/Browser/wwwtax.cgi?lvl=0&id=8030) |

Sequence similarity is available as [an NCBI BLAST search of TRF2_SALSA against nr](http://www.ncbi.nlm.nih.gov/blast/Blast.cgi?ALIGNMENTS=50;ALIGNMENT_VIEW=Pairwise;AUTO_FORMAT=Semiauto;CDD_SEARCH=on;CLIENT=web;COMPOSITION_BASED_STATISTICS=on;DATABASE=nr;DESCRIPTIONS=100;ENTREZ_QUERY=%28none%29;EXPECT=10;FILTER=L;FORMAT_BLOCK_ON_RESPAGE=None;FORMAT_OBJECT=Alignment;FORMAT_TYPE=HTML;GAPCOSTS=11%201;I_THRESH=0.001;LAYOUT=TwoWindows;MATRIX_NAME=BLOSUM62;NCBI_GI=on;PAGE=Proteins;PROGRAM=blastp;QUERY=MKLLLLSALLGCLATAYAAPAEGIVKWCVKSEQELRKCHDLAAKVAEFSCVRKDGSFECIQAIKGGEADAITLDGGDIYTAGLTNYGLQPIIAEDYGEDSDTCYYAVAVAKKGTAFGFKTLRGKKSCHTGLGKSAGWNIPIGTLVTESQIRWAGIEDRPVESAVSDFFNASCAPGATMGSKLCQLCKGDCSRSHKEPYYDYAGAFQCLKDGAGDVAFIKPLAVPAAEKASYELLCKDGTRASIDSYKTCHLARVPAHAVVSRKDPELANRIYNKLVAVKDFNLFSSDGYAAKNLMFKDSAQKLVQLPTTTDSFLYLGAEYMSTIRSLKKSQATGASSRAIKWCAVGHAEKGKCDTWTINSFADGESKISCQDAPTVEECIKKIMRKEADAIAVDGGEVYTAGKCGLVPVMVEQYDADLCSAPGEASSYYAVAVAKKGSGLTWKTLKGKRSCHTGLGRTAGWNIPMGLIHQETNDCDFTKYFSKGCAPGSEVGSPFCAQCKGSGKARGGDEDRCKARSEEQYYGYTGAFRCLVEDAGDVAFIKHTIVPESTDGNGPDWAKDLKSSDFELLCQDGTTQPVTKFSECHLAKVPAHAVITRPETRGDVVSILLELQAKFGSSGSDSSFRMFQSSVEKNLLFKDSTKCLQEIPKGTKYQDFLGKEYMIAMQSLRKCSDSTSDLEKACTFHSCQQKE;SERVICE=plain;SET_DEFAULTS.x=9;SET_DEFAULTS.y=5;SHOW_OVERVIEW=on;WORD_SIZE=3;END_OF_HTTPGET=Yes).**Search parameters**

| **MS data file:** | H:\MBA1-G6\22_BB10_01_1654.mgf |
| --- | --- |
| **Enzyme:** | Trypsin: cuts C-term side of KR unless next residue is P. |
| **Fixed modifications:** | [Carbamidomethyl (C)](http://fun-gen1.ibls.gla.ac.uk/mascot/cgi/client.pl?modification;file=..%2Fdata%2F20110930%2FF112275.dat;mod_name=Carbamidomethyl%20%28C%29) |
| **Variable modifications:** | [Oxidation (M)](http://fun-gen1.ibls.gla.ac.uk/mascot/cgi/client.pl?modification;file=..%2Fdata%2F20110930%2FF112275.dat;mod_name=Oxidation%20%28M%29) |

**Protein sequence coverage: 10%**

Matched peptides shown in ***bold red***.

| **1** | MKLLLLSALL | GCLATAYAAP | AEGIVKWCVK | SEQELRKCHD | LAAKVAEFSC |
| --- | --- | --- | --- | --- | --- |
| **51** | VRKDGSFECI | QAIKGGEADA | ITLDGGDIYT | AGLTNYGLQP | IIAEDYGEDS |
| **101** | DTCYYAVAVA | KKGTAFGFKT | LRGKKSCHTG | LGKSAGWNIP | IGTLVTESQI |
| **151** | RWAGIEDRPV | ESAVSDFFNA | SCAPGATMGS | KLCQLCKGDC | SRSHKEPYYD |
| **201** | YAGAFQCLK**D** | **GAGDVAFIKP** | **LAVPAAEK**AS | YELLCKDGTR | ASIDSYKTCH |
| **251** | LARVPAHAVV | SRKDPELANR | IYNKLVAVK**D** | **FNLFSSDGYA** | **AK**NLMFKDSA |
| **301** | QK**LVQLPTTT** | **DSFLYLGAEY** | **MSTIR**SLKKS | QATGASSRAI | KWCAVGHAEK |
| **351** | GKCDTWTINS | FADGESKISC | QDAPTVEECI | KKIMR**KEADA** | **IAVDGGEVYT** |
| **401** | **AGK**CGLVPVM | VEQYDADLCS | APGEASSYYA | VAVAKKGSGL | TWKTLKGKRS |
| **451** | CHTGLGRTAG | WNIPMGLIHQ | ETNDCDFTKY | FSKGCAPGSE | VGSPFCAQCK |
| **501** | GSGKARGGDE | DRCKARSEEQ | YYGYTGAFRC | LVEDAGDVAF | IKHTIVPEST |
| **551** | DGNGPDWAKD | LKSSDFELLC | QDGTTQPVTK | FSECHLAKVP | AHAVITRPET |
| **601** | RGDVVSILLE | LQAKFGSSGS | DSSFRMFQSS | VEKNLLFKDS | TKCLQEIPKG |
| **651** | TKYQDFLGKE | YMIAMQSLRK | CSDSTSDLEK | ACTFHSCQQK | E |

**SPOT # 669**

**Protein View: JH0472**

**apolipoprotein A-I precursor - Atlantic salmon**

Top of Form

| **Database:** | MSDB |
| --- | --- |
| **Score:** | 221 |
| **Nominal mass (M_r_):** | 29557 |
| **Calculated pI:** | 8.48 |
| **Taxonomy:** | [Salmo salar](http://www.ncbi.nlm.nih.gov/Taxonomy/Browser/wwwtax.cgi?lvl=0&id=8030) |

Sequence similarity is available as [an NCBI BLAST search of JH0472 against nr](http://www.ncbi.nlm.nih.gov/blast/Blast.cgi?ALIGNMENTS=50;ALIGNMENT_VIEW=Pairwise;AUTO_FORMAT=Semiauto;CDD_SEARCH=on;CLIENT=web;COMPOSITION_BASED_STATISTICS=on;DATABASE=nr;DESCRIPTIONS=100;ENTREZ_QUERY=%28none%29;EXPECT=10;FILTER=L;FORMAT_BLOCK_ON_RESPAGE=None;FORMAT_OBJECT=Alignment;FORMAT_TYPE=HTML;GAPCOSTS=11%201;I_THRESH=0.001;LAYOUT=TwoWindows;MATRIX_NAME=BLOSUM62;NCBI_GI=on;PAGE=Proteins;PROGRAM=blastp;QUERY=MKFLVLALTILLAAGTQAFPMQADAPSQLEHVKAALNMYIAQVKLTAQRSIDLLDDTEYKEYKMQLSQSLDNLQQFADSTSKSWPPTPRSSAPSCDATATVRAEVMKDVEDVRTQLEPKRAELREVLNKHIDEYRKKLEPLIKEHIELRRTEMDAFRAKIEPVVEEMRAKVAVNVEETKTKLMPIVEIVRAKLTERLEELRTLAAPYAEEYKEQMFKAVGEVREKVAPLSEDFKARWAPPPRRPSKSSWLSTRPSARP;SERVICE=plain;SET_DEFAULTS.x=9;SET_DEFAULTS.y=5;SHOW_OVERVIEW=on;WORD_SIZE=3;END_OF_HTTPGET=Yes).

**Search parameters**

| **MS data file:** | H:\MBA1-G6\23_BB11_01_1655.mgf |
| --- | --- |
| **Enzyme:** | Trypsin: cuts C-term side of KR unless next residue is P. |
| **Fixed modifications:** | [Carbamidomethyl (C)](http://fun-gen1.ibls.gla.ac.uk/mascot/cgi/client.pl?modification;file=..%2Fdata%2F20110930%2FF112276.dat;mod_name=Carbamidomethyl%20%28C%29) |
| **Variable modifications:** | [Oxidation (M)](http://fun-gen1.ibls.gla.ac.uk/mascot/cgi/client.pl?modification;file=..%2Fdata%2F20110930%2FF112276.dat;mod_name=Oxidation%20%28M%29) |

**Protein sequence coverage: 32%**

Matched peptides shown in ***bold red***.

| **1** | MKFLVLALTI | LLAAGTQAFP | MQADAPSQLE | HVK**AALNMYI** | **AQVK**LTAQR**S** |
| --- | --- | --- | --- | --- | --- |
| **51** | **IDLLDDTEYK** | **EYKMQLSQSL** | **DNLQQFADST** | **SK**SWPPTPRS | SAPSCDATAT |
| **101** | VRAEVMKDVE | DVRTQLEPKR | AELREVLNKH | IDEYRK**KLEP** | **LIK**EHIELRR |
| **151** | **TEMDAFRAKI** | **EPVVEEMR**AK | VAVNVEETKT | KLMPIVEIVR | AKLTERLEEL |
| **201** | R**TLAAPYAEE** | **YKEQMFK**AVG | EVREKVAPLS | EDFKARWAPP | PRRPSKSSWL |
| **251** | STRPSARP |  |  |  |  |

**SPOT # 545**

**Protein View: TRF2_SALSA**

**Serotransferrin II precursor (Siderophilin II) (STF II).- Salmo salar (Atlantic salmon).**

Top of Form

| **Database:** | MSDB |
| --- | --- |
| **Score:** | 64 |
| **Nominal mass (M_r_):** | 76497 |
| **Calculated pI:** | 7.08 |
| **Taxonomy:** | [Salmo salar](http://www.ncbi.nlm.nih.gov/Taxonomy/Browser/wwwtax.cgi?lvl=0&id=8030) |

Sequence similarity is available as [an NCBI BLAST search of TRF2_SALSA against nr](http://www.ncbi.nlm.nih.gov/blast/Blast.cgi?ALIGNMENTS=50;ALIGNMENT_VIEW=Pairwise;AUTO_FORMAT=Semiauto;CDD_SEARCH=on;CLIENT=web;COMPOSITION_BASED_STATISTICS=on;DATABASE=nr;DESCRIPTIONS=100;ENTREZ_QUERY=%28none%29;EXPECT=10;FILTER=L;FORMAT_BLOCK_ON_RESPAGE=None;FORMAT_OBJECT=Alignment;FORMAT_TYPE=HTML;GAPCOSTS=11%201;I_THRESH=0.001;LAYOUT=TwoWindows;MATRIX_NAME=BLOSUM62;NCBI_GI=on;PAGE=Proteins;PROGRAM=blastp;QUERY=MKLLLLSALLGCLATAYAAPAEGIVKWCVKSEQELRKCHDLAAKVAEFSCVRKDGSFECIQAIKGGEADAITLDGGDIYTAGLTNYGLQPIIAEDYGEDSDTCYYAVAVAKKGTAFGFKTLRGKKSCHTGLGKSAGWNIPIGTLVTESQIRWAGIEDRPVESAVSDFFNASCAPGATMGSKLCQLCKGDCSRSHKEPYYDYAGAFQCLKDGAGDVAFIKPLAVPAAEKASYELLCKDGTRASIDSYKTCHLARVPAHAVVSRKDPELANRIYNKLVAVKDFNLFSSDGYAAKNLMFKDSAQKLVQLPTTTDSFLYLGAEYMSTIRSLKKSQATGASSRAIKWCAVGHAEKGKCDTWTINSFADGESKISCQDAPTVEECIKKIMRKEADAIAVDGGEVYTAGKCGLVPVMVEQYDADLCSAPGEASSYYAVAVAKKGSGLTWKTLKGKRSCHTGLGRTAGWNIPMGLIHQETNDCDFTKYFSKGCAPGSEVGSPFCAQCKGSGKARGGDEDRCKARSEEQYYGYTGAFRCLVEDAGDVAFIKHTIVPESTDGNGPDWAKDLKSSDFELLCQDGTTQPVTKFSECHLAKVPAHAVITRPETRGDVVSILLELQAKFGSSGSDSSFRMFQSSVEKNLLFKDSTKCLQEIPKGTKYQDFLGKEYMIAMQSLRKCSDSTSDLEKACTFHSCQQKE;SERVICE=plain;SET_DEFAULTS.x=9;SET_DEFAULTS.y=5;SHOW_OVERVIEW=on;WORD_SIZE=3;END_OF_HTTPGET=Yes).**Search parameters**

| **MS data file:** | H:\MBA1-G6\24_BB12_01_1656.mgf |
| --- | --- |
| **Enzyme:** | Trypsin: cuts C-term side of KR unless next residue is P. |
| **Fixed modifications:** | [Carbamidomethyl (C)](http://fun-gen1.ibls.gla.ac.uk/mascot/cgi/client.pl?modification;file=..%2Fdata%2F20110930%2FF112277.dat;mod_name=Carbamidomethyl%20%28C%29) |
| **Variable modifications:** | [Oxidation (M)](http://fun-gen1.ibls.gla.ac.uk/mascot/cgi/client.pl?modification;file=..%2Fdata%2F20110930%2FF112277.dat;mod_name=Oxidation%20%28M%29) |

**Protein sequence coverage: 5%**

Matched peptides shown in ***bold red***.

| **1** | MKLLLLSALL | GCLATAYAAP | AEGIVKWCVK | SEQELRKCHD | LAAKVAEFSC |
| --- | --- | --- | --- | --- | --- |
| **51** | VRKDGSFECI | QAIKGGEADA | ITLDGGDIYT | AGLTNYGLQP | IIAEDYGEDS |
| **101** | DTCYYAVAVA | KKGTAFGFKT | LRGKKSCHTG | LGKSAGWNIP | IGTLVTESQI |
| **151** | RWAGIEDRPV | ESAVSDFFNA | SCAPGATMGS | KLCQLCKGDC | SRSHKEPYYD |
| **201** | YAGAFQCLKD | GAGDVAFIKP | LAVPAAEKAS | YELLCKDGTR | ASIDSYKTCH |
| **251** | LARVPAHAVV | SRKDPELANR | IYNKLVAVKD | FNLFSSDGYA | AKNLMFKDSA |
| **301** | QKLVQLPTTT | DSFLYLGAEY | MSTIRSLKKS | QATGASSRAI | KWCAVGHAEK |
| **351** | GKCDTWTINS | FADGESKISC | QDAPTVEECI | KKIMRKEADA | IAVDGGEVYT |
| **401** | AGKCGLVPVM | VEQYDADLCS | APGEASSYYA | VAVAKKGSGL | TWKTLKGKRS |
| **451** | CHTGLGRTAG | WNIPMGLIHQ | ETNDCDFTKY | FSKGCAPGSE | VGSPFCAQCK |
| **501** | GSGKARGGDE | DRCKAR**SEEQ** | **YYGYTGAFR**C | LVEDAGDVAF | IKHTIVPEST |
| **551** | DGNGPDWAKD | LKSSDFELLC | QDGTTQPVTK | FSECHLAK**VP** | **AHAVITRPET** |
| **601** | **RGDVVSILLE** | **LQAK**FGSSGS | DSSFRMFQSS | VEKNLLFKDS | TKCLQEIPKG |
| **651** | TKYQDFLGKE | YMIAMQSLRK | CSDSTSDLEK | ACTFHSCQQK | E |

**SPOT # 328**

**Protein View: Q9DDG6_SALTR**

**Alpha-2 enolase-1 (Fragment).- Salmo trutta (Brown trout).**

Top of Form

| **Database:** | MSDB |
| --- | --- |
| **Score:** | 292 |
| **Nominal mass (M_r_):** | 39667 |
| **Calculated pI:** | 5.78 |
| **Taxonomy:** | [Salmo trutta](http://www.ncbi.nlm.nih.gov/Taxonomy/Browser/wwwtax.cgi?lvl=0&id=8032) |

This protein sequence matches the following other entries:

- AAG16311 from [Salmo trutta](http://www.ncbi.nlm.nih.gov/Taxonomy/Browser/wwwtax.cgi?lvl=0&id=8032)

Sequence similarity is available as [an NCBI BLAST search of Q9DDG6_SALTR against nr](http://www.ncbi.nlm.nih.gov/blast/Blast.cgi?ALIGNMENTS=50;ALIGNMENT_VIEW=Pairwise;AUTO_FORMAT=Semiauto;CDD_SEARCH=on;CLIENT=web;COMPOSITION_BASED_STATISTICS=on;DATABASE=nr;DESCRIPTIONS=100;ENTREZ_QUERY=%28none%29;EXPECT=10;FILTER=L;FORMAT_BLOCK_ON_RESPAGE=None;FORMAT_OBJECT=Alignment;FORMAT_TYPE=HTML;GAPCOSTS=11%201;I_THRESH=0.001;LAYOUT=TwoWindows;MATRIX_NAME=BLOSUM62;NCBI_GI=on;PAGE=Proteins;PROGRAM=blastp;QUERY=TKKGLFRAAVPSGASTGIYEALELRDNDKTRYLGKGVKRAVKHINEFLAPALCNQNVNVLEQEKVDKLMLDMDGTENKSKFGANAILGVSLAVCKAGAAEKGVPLYRHIADLAGNPNXILPCPAFNVINGGSHAGNKLAMQEFMILPIGASNFHEAMRIGAEVYHNLKNVIKAKYGKDATNVGDEGGFAPNILENNEALELLKSAIEKAGYPDKIIIGMDVAASEFYKAGKYDLDFKSPDDPARYITXDQLGDLYKSFIKGYPVQSIEDPFDQDDWAAWSKFTAAVDIQVVGDDLTVTNPKRIQQAVEKKACNCLLLKVNQIGSVTESIKACKLAQSNGWGVMVSHRSGETEDTFIADLVVGL;SERVICE=plain;SET_DEFAULTS.x=9;SET_DEFAULTS.y=5;SHOW_OVERVIEW=on;WORD_SIZE=3;END_OF_HTTPGET=Yes).

**Search parameters**

| **MS data file:** | H:\MBA1-G6\25_BC1_01_1657.mgf |
| --- | --- |
| **Enzyme:** | Trypsin: cuts C-term side of KR unless next residue is P. |
| **Fixed modifications:** | [Carbamidomethyl (C)](http://fun-gen1.ibls.gla.ac.uk/mascot/cgi/client.pl?modification;file=..%2Fdata%2F20110930%2FF112278.dat;mod_name=Carbamidomethyl%20%28C%29) |
| **Variable modifications:** | [Oxidation (M)](http://fun-gen1.ibls.gla.ac.uk/mascot/cgi/client.pl?modification;file=..%2Fdata%2F20110930%2FF112278.dat;mod_name=Oxidation%20%28M%29) |

**Protein sequence coverage: 39%**

Matched peptides shown in ***bold red***.

| **1** | TKKGLFRAAV | PSGASTGIYE | ALELRDNDKT | RYLGKGVKRA | VKHINEFLAP |
| --- | --- | --- | --- | --- | --- |
| **51** | ALCNQNVNVL | EQEKVDKLML | DMDGTENKSK | **FGANAILGVS** | **LAVCK**AGAAE |
| **101** | KGVPLYRHIA | DLAGNPNXIL | PCPAFNVING | GSHAGNK**LAM** | **QEFMILPIGA** |
| **151** | **SNFHEAMRIG** | **AEVYHNLK**NV | IKAKYGK**DAT** | **NVGDEGGFAP** | **NILENNEALE** |
| **201** | **LLK**SAIEKAG | YPDK**IIIGMD** | **VAASEFYK**AG | KYDLDFKSPD | DPAR**YITXDQ** |
| **251** | **LGDLYK**SFIK | GYPVQSIEDP | FDQDDWAAWS | K**FTAAVDIQV** | **VGDDLTVTNP** |
| **301** | **K**RIQQAVEKK | ACNCLLLK**VN** | **QIGSVTESIK** | ACK**LAQSNGW** | **GVMVSHR**SGE |
| **351** | TEDTFIADLV | VGL |  |  |  |

**SPOT # 388**

**rotein View: Q804Y1_ICTPU**

**Aldolase (Fragment).- Ictalurus punctatus (Channel catfish).**

Top of Form

| **Database:** | MSDB |
| --- | --- |
| **Score:** | 94 |
| **Nominal mass (M_r_):** | 17424 |
| **Calculated pI:** | 8.73 |
| **Taxonomy:** | [Ictalurus punctatus](http://www.ncbi.nlm.nih.gov/Taxonomy/Browser/wwwtax.cgi?lvl=0&id=7998) |

This protein sequence matches the following other entries:

- AAO25766 from [Ictalurus punctatus](http://www.ncbi.nlm.nih.gov/Taxonomy/Browser/wwwtax.cgi?lvl=0&id=7998)

Sequence similarity is available as [an NCBI BLAST search of Q804Y1_ICTPU against nr](http://www.ncbi.nlm.nih.gov/blast/Blast.cgi?ALIGNMENTS=50;ALIGNMENT_VIEW=Pairwise;AUTO_FORMAT=Semiauto;CDD_SEARCH=on;CLIENT=web;COMPOSITION_BASED_STATISTICS=on;DATABASE=nr;DESCRIPTIONS=100;ENTREZ_QUERY=%28none%29;EXPECT=10;FILTER=L;FORMAT_BLOCK_ON_RESPAGE=None;FORMAT_OBJECT=Alignment;FORMAT_TYPE=HTML;GAPCOSTS=11%201;I_THRESH=0.001;LAYOUT=TwoWindows;MATRIX_NAME=BLOSUM62;NCBI_GI=on;PAGE=Proteins;PROGRAM=blastp;QUERY=MPHAYPFLSPEQKKELSDIALRIVAPGKGILAADESTGSVAKRFQSINAENTEENRRLYRQLLFTADDRVKPCIGGVIFFHETLYQKTDDGKLFPQLIKERGMVVGIKVDKGVVPLAGTNGETTTQGLDGLYERCAQYKKDGADFAKWRCVLKIT;SERVICE=plain;SET_DEFAULTS.x=9;SET_DEFAULTS.y=5;SHOW_OVERVIEW=on;WORD_SIZE=3;END_OF_HTTPGET=Yes).

**Search parameters**

| **MS data file:** | H:\MBA1-G6\26_BC2_01_1658.mgf |
| --- | --- |
| **Enzyme:** | Trypsin: cuts C-term side of KR unless next residue is P. |
| **Fixed modifications:** | [Carbamidomethyl (C)](http://fun-gen1.ibls.gla.ac.uk/mascot/cgi/client.pl?modification;file=..%2Fdata%2F20110930%2FF112279.dat;mod_name=Carbamidomethyl%20%28C%29) |
| **Variable modifications:** | [Oxidation (M)](http://fun-gen1.ibls.gla.ac.uk/mascot/cgi/client.pl?modification;file=..%2Fdata%2F20110930%2FF112279.dat;mod_name=Oxidation%20%28M%29) |

**Protein sequence coverage: 9%**

Matched peptides shown in ***bold red***.

| **1** | MPHAYPFLSP | EQKKELSDIA | LRIVAPGK**GI** | **LAADESTGSV** | **AK**RFQSINAE |
| --- | --- | --- | --- | --- | --- |
| **51** | NTEENRRLYR | QLLFTADDRV | KPCIGGVIFF | HETLYQKTDD | GKLFPQLIKE |
| **101** | RGMVVGIKVD | KGVVPLAGTN | GETTTQGLDG | LYERCAQYKK | DGADFAKWRC |
| **151** | VLKIT |  |  |  |  |

Bottom of Form

**SPOT # 249**

**Protein View: TRF2_SALSA**

**Serotransferrin II precursor (Siderophilin II) (STF II).- Salmo salar (Atlantic salmon).**

Top of Form

| **Database:** | MSDB |
| --- | --- |
| **Score:** | 101 |
| **Nominal mass (M_r_):** | 76497 |
| **Calculated pI:** | 7.08 |
| **Taxonomy:** | [Salmo salar](http://www.ncbi.nlm.nih.gov/Taxonomy/Browser/wwwtax.cgi?lvl=0&id=8030) |

Sequence similarity is available as [an NCBI BLAST search of TRF2_SALSA against nr](http://www.ncbi.nlm.nih.gov/blast/Blast.cgi?ALIGNMENTS=50;ALIGNMENT_VIEW=Pairwise;AUTO_FORMAT=Semiauto;CDD_SEARCH=on;CLIENT=web;COMPOSITION_BASED_STATISTICS=on;DATABASE=nr;DESCRIPTIONS=100;ENTREZ_QUERY=%28none%29;EXPECT=10;FILTER=L;FORMAT_BLOCK_ON_RESPAGE=None;FORMAT_OBJECT=Alignment;FORMAT_TYPE=HTML;GAPCOSTS=11%201;I_THRESH=0.001;LAYOUT=TwoWindows;MATRIX_NAME=BLOSUM62;NCBI_GI=on;PAGE=Proteins;PROGRAM=blastp;QUERY=MKLLLLSALLGCLATAYAAPAEGIVKWCVKSEQELRKCHDLAAKVAEFSCVRKDGSFECIQAIKGGEADAITLDGGDIYTAGLTNYGLQPIIAEDYGEDSDTCYYAVAVAKKGTAFGFKTLRGKKSCHTGLGKSAGWNIPIGTLVTESQIRWAGIEDRPVESAVSDFFNASCAPGATMGSKLCQLCKGDCSRSHKEPYYDYAGAFQCLKDGAGDVAFIKPLAVPAAEKASYELLCKDGTRASIDSYKTCHLARVPAHAVVSRKDPELANRIYNKLVAVKDFNLFSSDGYAAKNLMFKDSAQKLVQLPTTTDSFLYLGAEYMSTIRSLKKSQATGASSRAIKWCAVGHAEKGKCDTWTINSFADGESKISCQDAPTVEECIKKIMRKEADAIAVDGGEVYTAGKCGLVPVMVEQYDADLCSAPGEASSYYAVAVAKKGSGLTWKTLKGKRSCHTGLGRTAGWNIPMGLIHQETNDCDFTKYFSKGCAPGSEVGSPFCAQCKGSGKARGGDEDRCKARSEEQYYGYTGAFRCLVEDAGDVAFIKHTIVPESTDGNGPDWAKDLKSSDFELLCQDGTTQPVTKFSECHLAKVPAHAVITRPETRGDVVSILLELQAKFGSSGSDSSFRMFQSSVEKNLLFKDSTKCLQEIPKGTKYQDFLGKEYMIAMQSLRKCSDSTSDLEKACTFHSCQQKE;SERVICE=plain;SET_DEFAULTS.x=9;SET_DEFAULTS.y=5;SHOW_OVERVIEW=on;WORD_SIZE=3;END_OF_HTTPGET=Yes).

**Search parameters**

| **MS data file:** | H:\MBA1-G6\27_BC3_01_1659.mgf |
| --- | --- |
| **Enzyme:** | Trypsin: cuts C-term side of KR unless next residue is P. |
| **Fixed modifications:** | [Carbamidomethyl (C)](http://fun-gen1.ibls.gla.ac.uk/mascot/cgi/client.pl?modification;file=..%2Fdata%2F20110930%2FF112280.dat;mod_name=Carbamidomethyl%20%28C%29) |
| **Variable modifications:** | [Oxidation (M)](http://fun-gen1.ibls.gla.ac.uk/mascot/cgi/client.pl?modification;file=..%2Fdata%2F20110930%2FF112280.dat;mod_name=Oxidation%20%28M%29) |

**Protein sequence coverage: 7%**

Matched peptides shown in ***bold red***.

| **1** | MKLLLLSALL | GCLATAYAAP | AEGIVKWCVK | SEQELRKCHD | LAAKVAEFSC |
| --- | --- | --- | --- | --- | --- |
| **51** | VRKDGSFECI | QAIKGGEADA | ITLDGGDIYT | AGLTNYGLQP | IIAEDYGEDS |
| **101** | DTCYYAVAVA | KKGTAFGFKT | LRGKKSCHTG | LGKSAGWNIP | IGTLVTESQI |
| **151** | RWAGIEDRPV | ESAVSDFFNA | SCAPGATMGS | KLCQLCKGDC | SRSHKEPYYD |
| **201** | YAGAFQCLK**D** | **GAGDVAFIKP** | **LAVPAAEK**AS | YELLCKDGTR | ASIDSYKTCH |
| **251** | LARVPAHAVV | SRKDPELANR | IYNKLVAVK**D** | **FNLFSSDGYA** | **AK**NLMFKDSA |
| **301** | QKLVQLPTTT | DSFLYLGAEY | MSTIRSLKKS | QATGASSRAI | KWCAVGHAEK |
| **351** | GKCDTWTINS | FADGESKISC | QDAPTVEECI | KKIMRK**EADA** | **IAVDGGEVYT** |
| **401** | **AGK**CGLVPVM | VEQYDADLCS | APGEASSYYA | VAVAKKGSGL | TWKTLKGKRS |
| **451** | CHTGLGRTAG | WNIPMGLIHQ | ETNDCDFTKY | FSKGCAPGSE | VGSPFCAQCK |
| **501** | GSGKARGGDE | DRCKARSEEQ | YYGYTGAFRC | LVEDAGDVAF | IKHTIVPEST |
| **551** | DGNGPDWAKD | LKSSDFELLC | QDGTTQPVTK | FSECHLAKVP | AHAVITRPET |
| **601** | RGDVVSILLE | LQAKFGSSGS | DSSFRMFQSS | VEKNLLFKDS | TKCLQEIPKG |
| **651** | TKYQDFLGKE | YMIAMQSLRK | CSDSTSDLEK | ACTFHSCQQK | E |

Bottom of Form

**SPOT # 584**

**Protein View: Q7T3D9_BRARE**

**Malate dehydrogenase 1a, NAD (Soluble).- Brachydanio rerio (Zebrafish) (Danio rerio).**

Top of Form

| **Database:** | MSDB |
| --- | --- |
| **Score:** | 70 |
| **Nominal mass (M_r_):** | 33738 |
| **Calculated pI:** | 8.13 |
| **Taxonomy:** | [Danio rerio](http://www.ncbi.nlm.nih.gov/Taxonomy/Browser/wwwtax.cgi?lvl=0&id=7955) |

This protein sequence matches the following other entries:

- AAH53158 from [Danio rerio](http://www.ncbi.nlm.nih.gov/Taxonomy/Browser/wwwtax.cgi?lvl=0&id=7955)

Sequence similarity is available as [an NCBI BLAST search of Q7T3D9_BRARE against nr](http://www.ncbi.nlm.nih.gov/blast/Blast.cgi?ALIGNMENTS=50;ALIGNMENT_VIEW=Pairwise;AUTO_FORMAT=Semiauto;CDD_SEARCH=on;CLIENT=web;COMPOSITION_BASED_STATISTICS=on;DATABASE=nr;DESCRIPTIONS=100;ENTREZ_QUERY=%28none%29;EXPECT=10;FILTER=L;FORMAT_BLOCK_ON_RESPAGE=None;FORMAT_OBJECT=Alignment;FORMAT_TYPE=HTML;GAPCOSTS=11%201;I_THRESH=0.001;LAYOUT=TwoWindows;MATRIX_NAME=BLOSUM62;NCBI_GI=on;PAGE=Proteins;PROGRAM=blastp;QUERY=MCSARTSQSSWCSWITPMLPVLDGVVMELQDCALPLLREVIPTDKVEVGFKDLDAAILVGSMPRKEGMERKDLLKANVAIFKTQGEALEKYAKKTVKVLVVGNPANTNCLIASKSAPSIPKENFSCLTRLDHNRARSQVAMRVGVPSDSVKNVTIWGNHSSTQYPDVHHAIVTRNGKEIAAFDAVNDESWLKGDFISTVQQRGAAVIKARKLSSAMSAAKAICDHMRDIWFGTPDGEWVSMGIYSSGNSYGVPDDLMYSFPVKIKNKSWKVVDGLSINDFSRGKMDATAAELVEERDTALTFLSA;SERVICE=plain;SET_DEFAULTS.x=9;SET_DEFAULTS.y=5;SHOW_OVERVIEW=on;WORD_SIZE=3;END_OF_HTTPGET=Yes).

**Search parameters**

| **MS data file:** | H:\MBA1-G6\28_BC4_01_1660.mgf |
| --- | --- |
| **Enzyme:** | Trypsin: cuts C-term side of KR unless next residue is P. |
| **Fixed modifications:** | [Carbamidomethyl (C)](http://fun-gen1.ibls.gla.ac.uk/mascot/cgi/client.pl?modification;file=..%2Fdata%2F20110930%2FF112281.dat;mod_name=Carbamidomethyl%20%28C%29) |
| **Variable modifications:** | [Oxidation (M)](http://fun-gen1.ibls.gla.ac.uk/mascot/cgi/client.pl?modification;file=..%2Fdata%2F20110930%2FF112281.dat;mod_name=Oxidation%20%28M%29) |

**Protein sequence coverage: 4%**

Matched peptides shown in ***bold red***.

| **1** | MCSARTSQSS | WCSWITPMLP | VLDGVVMELQ | DCALPLLREV | IPTDKVEVGF |
| --- | --- | --- | --- | --- | --- |
| **51** | KDLDAAILVG | SMPRKEGMER | KDLLKANVAI | FKTQGEALEK | YAKKTVKVLV |
| **101** | VGNPANTNCL | IASKSAPSIP | KENFSCLTRL | DHNRARSQVA | MRVGVPSDSV |
| **151** | KNVTIWGNHS | STQYPDVHHA | IVTRNGKEIA | AFDAVNDESW | LKGDFISTVQ |
| **201** | QRGAAVIKAR | KLSSAMSAAK | AICDHMRDIW | FGTPDGEWVS | MGIYSSGNSY |
| **251** | GVPDDLMYSF | PVKIKNKSWK | VVDGLSINDF | SR**GKMDATAA** | **ELVEER**DTAL |
|  |  |  |  |  |  |
| **301** | TFLSA |  |  |  |  |

Bottom of Form

**SPOT # 299**

**Protein View: Q7ZZM5_SPAAU**

**Enolase (Fragment).- Sparus aurata (Gilthead sea bream).**

Top of Form

| **Database:** | MSDB |
| --- | --- |
| **Score:** | 308 |
| **Nominal mass (M_r_):** | 28752 |
| **Calculated pI:** | 8.15 |
| **Taxonomy:** | [Sparus aurata](http://www.ncbi.nlm.nih.gov/Taxonomy/Browser/wwwtax.cgi?lvl=0&id=8175) |

This protein sequence matches the following other entries:

- AAO92646 from [Sparus aurata](http://www.ncbi.nlm.nih.gov/Taxonomy/Browser/wwwtax.cgi?lvl=0&id=8175)

Sequence similarity is available as [an NCBI BLAST search of Q7ZZM5_SPAAU against nr](http://www.ncbi.nlm.nih.gov/blast/Blast.cgi?ALIGNMENTS=50;ALIGNMENT_VIEW=Pairwise;AUTO_FORMAT=Semiauto;CDD_SEARCH=on;CLIENT=web;COMPOSITION_BASED_STATISTICS=on;DATABASE=nr;DESCRIPTIONS=100;ENTREZ_QUERY=%28none%29;EXPECT=10;FILTER=L;FORMAT_BLOCK_ON_RESPAGE=None;FORMAT_OBJECT=Alignment;FORMAT_TYPE=HTML;GAPCOSTS=11%201;I_THRESH=0.001;LAYOUT=TwoWindows;MATRIX_NAME=BLOSUM62;NCBI_GI=on;PAGE=Proteins;PROGRAM=blastp;QUERY=SNFHEAMRIGAEVYHNLKNVIKAKYGKDATNVGDEGGFAPNILENNEALELLKTAIEKAGYPDKIIIGMDVAASEFYKAGKYDLDFKSPDDPARYIPGDQLGDLYKSFIKGISSPVHRGSLRQDDWAAWSKFPAAVDIQVVGDDLTVTNPKRIQQAVEKKACNCLLLKVNQIGSVTESIKACKLAQSNGWGVMVSHRSGETEDTFIADLVVGLCTGQIKTGAPCRSERLAKYNQLMRIEEELGAKAKFAGKDYRRPKIN;SERVICE=plain;SET_DEFAULTS.x=9;SET_DEFAULTS.y=5;SHOW_OVERVIEW=on;WORD_SIZE=3;END_OF_HTTPGET=Yes).

**Search parameters**

| **MS data file:** | H:\MBA1-G6\29_BC5_01_1661.mgf |
| --- | --- |
| **Enzyme:** | Trypsin: cuts C-term side of KR unless next residue is P. |
| **Fixed modifications:** | [Carbamidomethyl (C)](http://fun-gen1.ibls.gla.ac.uk/mascot/cgi/client.pl?modification;file=..%2Fdata%2F20110930%2FF112282.dat;mod_name=Carbamidomethyl%20%28C%29) |
| **Variable modifications:** | [Oxidation (M)](http://fun-gen1.ibls.gla.ac.uk/mascot/cgi/client.pl?modification;file=..%2Fdata%2F20110930%2FF112282.dat;mod_name=Oxidation%20%28M%29) |

**Protein sequence coverage: 25%**

Matched peptides shown in ***bold red***.

| **1** | SNFHEAMR**IG** | **AEVYHNLK**NV | IKAKYGKDAT | NVGDEGGFAP | NILENNEALE |
| --- | --- | --- | --- | --- | --- |
| **51** | LLKTAIEKAG | YPDK**IIIGMD** | **VAASEFYK**AG | KYDLDFKSPD | DPARYIPGDQ |
| **101** | LGDLYKSFIK | GISSPVHRGS | LRQDDWAAWS | KFPAAVDIQV | VGDDLTVTNP |
| **151** | KRIQQAVEKK | ACNCLLLK**VN** | **QIGSVTESIK** | ACKLAQSNGW | GVMVSHR**SGE** |
| **201** | **TEDTFIADLV** | **VGLCTGQIK**T | GAPCRSERLA | KYNQLMR**IEE** | **ELGAK**AKFAG |
| **251** | KDYRRPKIN |  |  |  |  |

**SPOT # 586**

**Protein View: JH0472**

**apolipoprotein A-I precursor - Atlantic salmon**

Top of Form

| **Database:** | MSDB |
| --- | --- |
| **Score:** | 262 |
| **Nominal mass (M_r_):** | 29557 |
| **Calculated pI:** | 8.48 |
| **Taxonomy:** | [Salmo salar](http://www.ncbi.nlm.nih.gov/Taxonomy/Browser/wwwtax.cgi?lvl=0&id=8030) |

Sequence similarity is available as [an NCBI BLAST search of JH0472 against nr](http://www.ncbi.nlm.nih.gov/blast/Blast.cgi?ALIGNMENTS=50;ALIGNMENT_VIEW=Pairwise;AUTO_FORMAT=Semiauto;CDD_SEARCH=on;CLIENT=web;COMPOSITION_BASED_STATISTICS=on;DATABASE=nr;DESCRIPTIONS=100;ENTREZ_QUERY=%28none%29;EXPECT=10;FILTER=L;FORMAT_BLOCK_ON_RESPAGE=None;FORMAT_OBJECT=Alignment;FORMAT_TYPE=HTML;GAPCOSTS=11%201;I_THRESH=0.001;LAYOUT=TwoWindows;MATRIX_NAME=BLOSUM62;NCBI_GI=on;PAGE=Proteins;PROGRAM=blastp;QUERY=MKFLVLALTILLAAGTQAFPMQADAPSQLEHVKAALNMYIAQVKLTAQRSIDLLDDTEYKEYKMQLSQSLDNLQQFADSTSKSWPPTPRSSAPSCDATATVRAEVMKDVEDVRTQLEPKRAELREVLNKHIDEYRKKLEPLIKEHIELRRTEMDAFRAKIEPVVEEMRAKVAVNVEETKTKLMPIVEIVRAKLTERLEELRTLAAPYAEEYKEQMFKAVGEVREKVAPLSEDFKARWAPPPRRPSKSSWLSTRPSARP;SERVICE=plain;SET_DEFAULTS.x=9;SET_DEFAULTS.y=5;SHOW_OVERVIEW=on;WORD_SIZE=3;END_OF_HTTPGET=Yes).

**Search parameters**

| **MS data file:** | H:\MBA1-G6\30_BC6_01_1662.mgf |
| --- | --- |
| **Enzyme:** | Trypsin: cuts C-term side of KR unless next residue is P. |
| **Fixed modifications:** | [Carbamidomethyl (C)](http://fun-gen1.ibls.gla.ac.uk/mascot/cgi/client.pl?modification;file=..%2Fdata%2F20110930%2FF112283.dat;mod_name=Carbamidomethyl%20%28C%29) |
| **Variable modifications:** | [Oxidation (M)](http://fun-gen1.ibls.gla.ac.uk/mascot/cgi/client.pl?modification;file=..%2Fdata%2F20110930%2FF112283.dat;mod_name=Oxidation%20%28M%29) |

**Protein sequence coverage: 27%**

Matched peptides shown in ***bold red***.

| **1** | MKFLVLALTI | LLAAGTQAFP | MQADAPSQLE | HVK**AALNMYI** | **AQVK**LTAQR**S** |
| --- | --- | --- | --- | --- | --- |
| **51** | **IDLLDDTEYK** | **EYKMQLSQSL** | **DNLQQFADST** | **SK**SWPPTPRS | SAPSCDATAT |
| **101** | VRAEVMKDVE | DVRTQLEPKR | AELREVLNKH | IDEYRKKLEP | LIKEHIELRR |
| **151** | TEMDAFR**AKI** | **EPVVEEMR**AK | VAVNVEETKT | KLMPIVEIVR | AKLTERLEEL |
| **201** | R**TLAAPYAEE** | **YKEQMFK**AVG | EVREKVAPLS | EDFKARWAPP | PRRPSKSSWL |
| **251** | STRPSARP |  |  |  |  |

**SPOT # 98**

**apolipoprotein A-I precursor - Atlantic salmon**

Top of Form

| **Database:** | MSDB |
| --- | --- |
| **Score:** | 113 |
| **Nominal mass (M_r_):** | 29557 |
| **Calculated pI:** | 8.48 |
| **Taxonomy:** | [Salmo salar](http://www.ncbi.nlm.nih.gov/Taxonomy/Browser/wwwtax.cgi?lvl=0&id=8030) |

Sequence similarity is available as [an NCBI BLAST search of JH0472 against nr](http://www.ncbi.nlm.nih.gov/blast/Blast.cgi?ALIGNMENTS=50;ALIGNMENT_VIEW=Pairwise;AUTO_FORMAT=Semiauto;CDD_SEARCH=on;CLIENT=web;COMPOSITION_BASED_STATISTICS=on;DATABASE=nr;DESCRIPTIONS=100;ENTREZ_QUERY=%28none%29;EXPECT=10;FILTER=L;FORMAT_BLOCK_ON_RESPAGE=None;FORMAT_OBJECT=Alignment;FORMAT_TYPE=HTML;GAPCOSTS=11%201;I_THRESH=0.001;LAYOUT=TwoWindows;MATRIX_NAME=BLOSUM62;NCBI_GI=on;PAGE=Proteins;PROGRAM=blastp;QUERY=MKFLVLALTILLAAGTQAFPMQADAPSQLEHVKAALNMYIAQVKLTAQRSIDLLDDTEYKEYKMQLSQSLDNLQQFADSTSKSWPPTPRSSAPSCDATATVRAEVMKDVEDVRTQLEPKRAELREVLNKHIDEYRKKLEPLIKEHIELRRTEMDAFRAKIEPVVEEMRAKVAVNVEETKTKLMPIVEIVRAKLTERLEELRTLAAPYAEEYKEQMFKAVGEVREKVAPLSEDFKARWAPPPRRPSKSSWLSTRPSARP;SERVICE=plain;SET_DEFAULTS.x=9;SET_DEFAULTS.y=5;SHOW_OVERVIEW=on;WORD_SIZE=3;END_OF_HTTPGET=Yes).

**Search parameters**

| **MS data file:** | H:\MBA1-G6\31_BC7_01_1663.mgf |
| --- | --- |
| **Enzyme:** | Trypsin: cuts C-term side of KR unless next residue is P. |
| **Fixed modifications:** | [Carbamidomethyl (C)](http://fun-gen1.ibls.gla.ac.uk/mascot/cgi/client.pl?modification;file=..%2Fdata%2F20110930%2FF112284.dat;mod_name=Carbamidomethyl%20%28C%29) |
| **Variable modifications:** | [Oxidation (M)](http://fun-gen1.ibls.gla.ac.uk/mascot/cgi/client.pl?modification;file=..%2Fdata%2F20110930%2FF112284.dat;mod_name=Oxidation%20%28M%29) |

**Protein sequence coverage: 23%**

Matched peptides shown in ***bold red***.

| **1** | MKFLVLALTI | LLAAGTQAFP | MQADAPSQLE | HVKAALNMYI | AQVKLTAQR**S** |
| --- | --- | --- | --- | --- | --- |
| **51** | **IDLLDDTEYK** | **EYKMQLSQSL** | **DNLQQFADST** | **SK**SWPPTPRS | SAPSCDATAT |
| **101** | VRAEVMKDVE | DVRTQLEPKR | AELREVLNKH | IDEYRKKLEP | LIKEHIELRR |
| **151** | TEMDAFR**AKI** | **EPVVEEMR**AK | VAVNVEETKT | KLMPIVEIVR | AKLTERLEEL |
| **201** | R**TLAAPYAEE** | **YKEQMFK**AVG | EVREKVAPLS | EDFKARWAPP | PRRPSKSSWL |
| **251** | STRPSARP |  |  |  |  |

**SPOT # 313**

**Protein View: Q8QGU8_FUGRU**

**Pyruvate kinase.- Fugu rubripes (Japanese pufferfish) (Takifugu rubripes).**

Top of Form

| **Database:** | MSDB |
| --- | --- |
| **Score:** | 155 |
| **Nominal mass (M_r_):** | 58572 |
| **Calculated pI:** | 7.96 |
| **Taxonomy:** | [Takifugu rubripes](http://www.ncbi.nlm.nih.gov/Taxonomy/Browser/wwwtax.cgi?lvl=0&id=31033) |

This protein sequence matches the following other entries:

- BAB91009 from [Takifugu rubripes](http://www.ncbi.nlm.nih.gov/Taxonomy/Browser/wwwtax.cgi?lvl=0&id=31033)

Sequence similarity is available as [an NCBI BLAST search of Q8QGU8_FUGRU against nr](http://www.ncbi.nlm.nih.gov/blast/Blast.cgi?ALIGNMENTS=50;ALIGNMENT_VIEW=Pairwise;AUTO_FORMAT=Semiauto;CDD_SEARCH=on;CLIENT=web;COMPOSITION_BASED_STATISTICS=on;DATABASE=nr;DESCRIPTIONS=100;ENTREZ_QUERY=%28none%29;EXPECT=10;FILTER=L;FORMAT_BLOCK_ON_RESPAGE=None;FORMAT_OBJECT=Alignment;FORMAT_TYPE=HTML;GAPCOSTS=11%201;I_THRESH=0.001;LAYOUT=TwoWindows;MATRIX_NAME=BLOSUM62;NCBI_GI=on;PAGE=Proteins;PROGRAM=blastp;QUERY=MSKSKDMSSSAIHTQQLHAAIADTFIEHMCLLDIDSEPAVSRNTGIVCTIGPASRSVEMAKEMIKSGMNVARMNFSHGTHEYHAETIKNVREATESFGPGSVNYRPVAIALDTKGPEIRTGLIKGSGTAEVELKKGETIKITLDDQYMEKCDEKILWLDYKNITKVVQVGSHVYVDDGLITLTVKEVGSDYLMCTIGNGGTLGSKKGVNLPGAAVDLPAVSEKDVKDLQFGVEQGVDMVFASFIRKAADVHAVRKVLGEKGKDIKIISKLENHEGVRRFDEILEASDGIMVARGDLGIEIPTEKVFLAQKMMTGKCNRVGKPIICATQMLESMTKKPRPTRAEASDVANAVLDGNDCIMLSGETAKGDYPLEAVHTQHMIAREAEAAMFHRQMFEELRRITHLTRDPTETIAIGAVEASFKCCASAIIVLTKTGRSAHMLSRYRPRAPIIAVTRCGQTARQAHLYRGIYPVLYTKPANDVWAEDVDLRVNFALEVGKHRKFLKSGDVALVVTGWRPGPGYTNTVRVVLVP;SERVICE=plain;SET_DEFAULTS.x=9;SET_DEFAULTS.y=5;SHOW_OVERVIEW=on;WORD_SIZE=3;END_OF_HTTPGET=Yes).

**Search parameters**

| **MS data file:** | H:\MBA1-G6\32_BC8_01_1664.mgf |
| --- | --- |
| **Enzyme:** | Trypsin: cuts C-term side of KR unless next residue is P. |
| **Fixed modifications:** | [Carbamidomethyl (C)](http://fun-gen1.ibls.gla.ac.uk/mascot/cgi/client.pl?modification;file=..%2Fdata%2F20110930%2FF112285.dat;mod_name=Carbamidomethyl%20%28C%29) |
| **Variable modifications:** | [Oxidation (M)](http://fun-gen1.ibls.gla.ac.uk/mascot/cgi/client.pl?modification;file=..%2Fdata%2F20110930%2FF112285.dat;mod_name=Oxidation%20%28M%29) |

**Protein sequence coverage: 11%**

Matched peptides shown in ***bold red***.

| **1** | MSKSKDMSSS | AIHTQQLHAA | IADTFIEHMC | LLDIDSEPAV | SRNTGIVCTI |
| --- | --- | --- | --- | --- | --- |
| **51** | GPASRSVEMA | KEMIKSGMNV | AR**MNFSHGTH** | **EYHAETIK**NV | REATESFGPG |
| **101** | SVNYRPVAIA | LDTKGPEIRT | GLIKGSGTAE | VELKKGETIK | ITLDDQYMEK |
| **151** | CDEKILWLDY | KNITKVVQVG | SHVYVDDGLI | TLTVKEVGSD | YLMCTIGNGG |
| **201** | TLGSK**KGVNL** | **PGAAVDLPAV** | **SEK**DVKDLQF | GVEQGVDMVF | ASFIRKAADV |
| **251** | HAVRKVLGEK | GKDIKIISKL | ENHEGVR**RFD** | **EILEASDGIM** | **VARGDLGIEI** |
| **301** | **PTEK**VFLAQK | MMTGKCNRVG | KPIICATQML | ESMTKKPRPT | RAEASDVANA |
| **351** | VLDGNDCIML | SGETAKGDYP | LEAVHTQHMI | AREAEAAMFH | RQMFEELRRI |
| **401** | THLTRDPTET | IAIGAVEASF | KCCASAIIVL | TKTGRSAHML | SRYRPRAPII |
| **451** | AVTRCGQTAR | QAHLYRGIYP | VLYTKPANDV | WAEDVDLRVN | FALEVGKHRK |
| **501** | FLKSGDVALV | VTGWRPGPGY | TNTVRVVLVP |  |  |

Bottom of Form

**SPOT # 463**

**Protein View: Q90ZF1_ONCMY**

**Glyceraldehyde 3-phosphate dehydrogenase (EC 1.2.1.12).- Oncorhynchus mykiss (Rainbow trout) (Salmo gairdneri).**

Top of Form

| **Database:** | MSDB |
| --- | --- |
| **Score:** | 247 |
| **Nominal mass (M_r_):** | 36055 |
| **Calculated pI:** | 8.63 |
| **Taxonomy:** | [Oncorhynchus mykiss](http://www.ncbi.nlm.nih.gov/Taxonomy/Browser/wwwtax.cgi?lvl=0&id=8022) |

This protein sequence matches the following other entries:

- BAB62189 from [Oncorhynchus mykiss](http://www.ncbi.nlm.nih.gov/Taxonomy/Browser/wwwtax.cgi?lvl=0&id=8022)

Sequence similarity is available as [an NCBI BLAST search of Q90ZF1_ONCMY against nr](http://www.ncbi.nlm.nih.gov/blast/Blast.cgi?ALIGNMENTS=50;ALIGNMENT_VIEW=Pairwise;AUTO_FORMAT=Semiauto;CDD_SEARCH=on;CLIENT=web;COMPOSITION_BASED_STATISTICS=on;DATABASE=nr;DESCRIPTIONS=100;ENTREZ_QUERY=%28none%29;EXPECT=10;FILTER=L;FORMAT_BLOCK_ON_RESPAGE=None;FORMAT_OBJECT=Alignment;FORMAT_TYPE=HTML;GAPCOSTS=11%201;I_THRESH=0.001;LAYOUT=TwoWindows;MATRIX_NAME=BLOSUM62;NCBI_GI=on;PAGE=Proteins;PROGRAM=blastp;QUERY=MVKVGVNGFGRIGRLVTRAAFHSKKGVEIVAINDPFIDLDYMVYMFKYDSTHGRFHGEVKAEGGKLVIDGHKITVFHERDPANIKWGDAGATYVVESTGVFTTIEKASTHLKGGAKRVVISAPSADAPMFVMGVNHEKYENSLKVVSNASCTTNCLAPLAKVIHDNYHIIEGLMSTVHAVTATQKTVDGPSGKLWRDGRGASQNIIPASTGAAKAVGKVIPELNGKITGMAFRVPTPNVSVVDLTVRLEKPASYDAIKKVVKAAADGPMKGILGYTEQQVVSSDFNGDTHSSIFDAGAGIALNDHFVKLVTWYDNEFGYSNRVIDLMAHMATKE;SERVICE=plain;SET_DEFAULTS.x=9;SET_DEFAULTS.y=5;SHOW_OVERVIEW=on;WORD_SIZE=3;END_OF_HTTPGET=Yes).

**Search parameters**

| **MS data file:** | H:\MBA1-G6\33_BC9_01_1665.mgf |
| --- | --- |
| **Enzyme:** | Trypsin: cuts C-term side of KR unless next residue is P. |
| **Fixed modifications:** | [Carbamidomethyl (C)](http://fun-gen1.ibls.gla.ac.uk/mascot/cgi/client.pl?modification;file=..%2Fdata%2F20110930%2FF112286.dat;mod_name=Carbamidomethyl%20%28C%29) |
| **Variable modifications:** | [Oxidation (M)](http://fun-gen1.ibls.gla.ac.uk/mascot/cgi/client.pl?modification;file=..%2Fdata%2F20110930%2FF112286.dat;mod_name=Oxidation%20%28M%29) |

**Protein sequence coverage: 41%**

Matched peptides shown in ***bold red***.

| **1** | MVKVGVNGFG | RIGRLVTRAA | FHSKK**GVEIV** | **AINDPFIDLD** | **YMVYMFK**YDS |
| --- | --- | --- | --- | --- | --- |
| **51** | THGRFHGEVK | AEGGKLVIDG | HK**ITVFHERD** | **PANIK**WGDAG | ATYVVESTGV |
| **101** | FTTIEKASTH | LKGGAK**RVVI** | **SAPSADAPMF** | **VMGVNHEK**YE | NSLK**VVSNAS** |
| **151** | **CTTNCLAPLA** | **KVIHDNYHII** | **EGLMSTVHAV** | **TATQK**TVDGP | SGKLWRDGR**G** |
| **201** | **ASQNIIPAST** | **GAAK**AVGKVI | PELNGKITGM | AFR**VPTPNVS** | **VVDLTVRLEK** |
| **251** | **PASYDAIKK**V | VKAAADGPMK | GILGYTEQQV | VSSDFNGDTH | SSIFDAGAGI |
| **301** | ALNDHFVKLV | TWYDNEFGYS | NRVIDLMAHM | ATKE |  |

**SPOT # 556**

**Protein View: TRF2_SALSA**

**Serotransferrin II precursor (Siderophilin II) (STF II).- Salmo salar (Atlantic salmon).**

Top of Form

| **Database:** | MSDB |
| --- | --- |
| **Score:** | 404 |
| **Nominal mass (M_r_):** | 76497 |
| **Calculated pI:** | 7.08 |
| **Taxonomy:** | [Salmo salar](http://www.ncbi.nlm.nih.gov/Taxonomy/Browser/wwwtax.cgi?lvl=0&id=8030) |

Sequence similarity is available as [an NCBI BLAST search of TRF2_SALSA against nr](http://www.ncbi.nlm.nih.gov/blast/Blast.cgi?ALIGNMENTS=50;ALIGNMENT_VIEW=Pairwise;AUTO_FORMAT=Semiauto;CDD_SEARCH=on;CLIENT=web;COMPOSITION_BASED_STATISTICS=on;DATABASE=nr;DESCRIPTIONS=100;ENTREZ_QUERY=%28none%29;EXPECT=10;FILTER=L;FORMAT_BLOCK_ON_RESPAGE=None;FORMAT_OBJECT=Alignment;FORMAT_TYPE=HTML;GAPCOSTS=11%201;I_THRESH=0.001;LAYOUT=TwoWindows;MATRIX_NAME=BLOSUM62;NCBI_GI=on;PAGE=Proteins;PROGRAM=blastp;QUERY=MKLLLLSALLGCLATAYAAPAEGIVKWCVKSEQELRKCHDLAAKVAEFSCVRKDGSFECIQAIKGGEADAITLDGGDIYTAGLTNYGLQPIIAEDYGEDSDTCYYAVAVAKKGTAFGFKTLRGKKSCHTGLGKSAGWNIPIGTLVTESQIRWAGIEDRPVESAVSDFFNASCAPGATMGSKLCQLCKGDCSRSHKEPYYDYAGAFQCLKDGAGDVAFIKPLAVPAAEKASYELLCKDGTRASIDSYKTCHLARVPAHAVVSRKDPELANRIYNKLVAVKDFNLFSSDGYAAKNLMFKDSAQKLVQLPTTTDSFLYLGAEYMSTIRSLKKSQATGASSRAIKWCAVGHAEKGKCDTWTINSFADGESKISCQDAPTVEECIKKIMRKEADAIAVDGGEVYTAGKCGLVPVMVEQYDADLCSAPGEASSYYAVAVAKKGSGLTWKTLKGKRSCHTGLGRTAGWNIPMGLIHQETNDCDFTKYFSKGCAPGSEVGSPFCAQCKGSGKARGGDEDRCKARSEEQYYGYTGAFRCLVEDAGDVAFIKHTIVPESTDGNGPDWAKDLKSSDFELLCQDGTTQPVTKFSECHLAKVPAHAVITRPETRGDVVSILLELQAKFGSSGSDSSFRMFQSSVEKNLLFKDSTKCLQEIPKGTKYQDFLGKEYMIAMQSLRKCSDSTSDLEKACTFHSCQQKE;SERVICE=plain;SET_DEFAULTS.x=9;SET_DEFAULTS.y=5;SHOW_OVERVIEW=on;WORD_SIZE=3;END_OF_HTTPGET=Yes).

**Search parameters**

| **MS data file:** | H:\MBA1-G6\34_BC10_01_1666.mgf |
| --- | --- |
| **Enzyme:** | Trypsin: cuts C-term side of KR unless next residue is P. |
| **Fixed modifications:** | [Carbamidomethyl (C)](http://fun-gen1.ibls.gla.ac.uk/mascot/cgi/client.pl?modification;file=..%2Fdata%2F20110930%2FF112287.dat;mod_name=Carbamidomethyl%20%28C%29) |
| **Variable modifications:** | [Oxidation (M)](http://fun-gen1.ibls.gla.ac.uk/mascot/cgi/client.pl?modification;file=..%2Fdata%2F20110930%2FF112287.dat;mod_name=Oxidation%20%28M%29) |

**Protein sequence coverage: 16%**

Matched peptides shown in ***bold red***.

| **1** | MKLLLLSALL | GCLATAYAAP | AEGIVKWCVK | SEQELRKCHD | LAAKVAEFSC |
| --- | --- | --- | --- | --- | --- |
| **51** | VRKDGSFECI | QAIKGGEADA | ITLDGGDIYT | AGLTNYGLQP | IIAEDYGEDS |
| **101** | DTCYYAVAVA | KKGTAFGFKT | LRGKKSCHTG | LGKSAGWNIP | IGTLVTESQI |
| **151** | RWAGIEDRPV | ESAVSDFFNA | SCAPGATMGS | KLCQLCKGDC | SRSHKEPYYD |
| **201** | YAGAFQCLKD | GAGDVAFIKP | LAVPAAEKAS | YELLCKDGTR | ASIDSYKTCH |
| **251** | LARVPAHAVV | SRKDPELANR | IYNKLVAVKD | FNLFSSDGYA | AKNLMFKDSA |
| **301** | QKLVQLPTTT | DSFLYLGAEY | MSTIRSLKKS | QATGASSRAI | KWCAVGHAEK |
| **351** | GKCDTWTINS | FADGESKISC | QDAPTVEECI | KKIMRKEADA | IAVDGGEVYT |
| **401** | AGKCGLVPVM | VEQYDADLCS | APGEASSYYA | VAVAKKGSGL | TWKTLKGKRS |
| **451** | CHTGLGRTAG | WNIPMGLIHQ | ETNDCDFTKY | FSK**GCAPGSE** | **VGSPFCAQCK** |
| **501** | GSGKARGGDE | DRCKAR**SEEQ** | **YYGYTGAFRC** | **LVEDAGDVAF** | **IK**HTIVPEST |
| **551** | DGNGPDWAKD | LK**SSDFELLC** | **QDGTTQPVTK** | FSECHLAK**VP** | **AHAVITRPET** |
| **601** | **RGDVVSILLE** | **LQAKFGSSGS** | **DSSFR**MFQSS | VEKNLLFKDS | TK**CLQEIPK**G |
| **651** | TKYQDFLGK**E** | **YMIAMQSLR**K | CSDSTSDLEK | ACTFHSCQQK | E |

**Protein View: AAG18369**

**Immunoglobulin light chain precursor.- Salmo salar (Atlantic salmon).**

Top of Form

| **Database:** | MSDB |
| --- | --- |
| **Score:** | 150 |
| **Nominal mass (M_r_):** | 26519 |
| **Calculated pI:** | 6.29 |
| **Taxonomy:** | [Salmo salar](http://www.ncbi.nlm.nih.gov/Taxonomy/Browser/wwwtax.cgi?lvl=0&id=8030) |

Sequence similarity is available as [an NCBI BLAST search of AAG18369 against nr](http://www.ncbi.nlm.nih.gov/blast/Blast.cgi?ALIGNMENTS=50;ALIGNMENT_VIEW=Pairwise;AUTO_FORMAT=Semiauto;CDD_SEARCH=on;CLIENT=web;COMPOSITION_BASED_STATISTICS=on;DATABASE=nr;DESCRIPTIONS=100;ENTREZ_QUERY=%28none%29;EXPECT=10;FILTER=L;FORMAT_BLOCK_ON_RESPAGE=None;FORMAT_OBJECT=Alignment;FORMAT_TYPE=HTML;GAPCOSTS=11%201;I_THRESH=0.001;LAYOUT=TwoWindows;MATRIX_NAME=BLOSUM62;NCBI_GI=on;PAGE=Proteins;PROGRAM=blastp;QUERY=MTFIMSFVWILMSLIHESRGQVTVTQTPAVKAVLTGQTVPLNCKTSSDVYQAGTSSPRLAWYQQKPGEAPKLLIYYATTLQSGTPSRFSGSGTHSDFTLTISGVQAEDAGDYYCQSFHYPNSKYVYTFGSATRLDVGSNSAPTLTVLPPSSEELSSTTTATLMCLANKGFPSDWTMSWKVDGNSKKQEASPGVLEKDGLYSWSSTLTLTAQEWTKAGEVTCEAQQISQTPVTKTLRRADCSG;SERVICE=plain;SET_DEFAULTS.x=9;SET_DEFAULTS.y=5;SHOW_OVERVIEW=on;WORD_SIZE=3;END_OF_HTTPGET=Yes).

**Search parameters**

| **MS data file:** | H:\MBA1-G6\34_BC10_01_1666.mgf |
| --- | --- |
| **Enzyme:** | Trypsin: cuts C-term side of KR unless next residue is P. |
| **Fixed modifications:** | [Carbamidomethyl (C)](http://fun-gen1.ibls.gla.ac.uk/mascot/cgi/client.pl?modification;file=..%2Fdata%2F20110930%2FF112287.dat;mod_name=Carbamidomethyl%20%28C%29) |
| **Variable modifications:** | [Oxidation (M)](http://fun-gen1.ibls.gla.ac.uk/mascot/cgi/client.pl?modification;file=..%2Fdata%2F20110930%2FF112287.dat;mod_name=Oxidation%20%28M%29) |

**Protein sequence coverage: 33%**

Matched peptides shown in ***bold red***.

| **1** | MTFIMSFVWI | LMSLIHESRG | QVTVTQTPAV | K**AVLTGQTVP** | **LNCKTSSDVY** |
| --- | --- | --- | --- | --- | --- |
| **51** | **QAGTSSPR**LA | WYQQKPGEAP | K**LLIYYATTL** | **QSGTPSR**FSG | SGTHSDFTLT |
| **101** | ISGVQAEDAG | DYYCQSFHYP | NSK**YVYTFGS** | **ATR**LDVGSNS | APTLTVLPPS |
| **151** | SEELSSTTTA | TLMCLANKGF | PSDWTMSWKV | DGNSK**KQEAS** | **PGVLEK**DGLY |
| **201** | SWSSTLTLTA | QEWTK**AGEVT** | **CEAQQISQTP** | **VTK**TLRRADC | SG |

**SPOT # 565**

**Protein View: P79825_ONCMY**

**Hemopexin-like protein (Fragment).- Oncorhynchus mykiss (Rainbow trout) (Salmo gairdneri).**

Top of Form

| **Database:** | MSDB |
| --- | --- |
| **Score:** | 51 |
| **Nominal mass (M_r_):** | 51106 |
| **Calculated pI:** | 5.61 |
| **Taxonomy:** | [Oncorhynchus mykiss](http://www.ncbi.nlm.nih.gov/Taxonomy/Browser/wwwtax.cgi?lvl=0&id=8022) |

This protein sequence matches the following other entries:

- CAA92147 from [Oncorhynchus mykiss](http://www.ncbi.nlm.nih.gov/Taxonomy/Browser/wwwtax.cgi?lvl=0&id=8022)

Sequence similarity is available as [an NCBI BLAST search of P79825_ONCMY against nr](http://www.ncbi.nlm.nih.gov/blast/Blast.cgi?ALIGNMENTS=50;ALIGNMENT_VIEW=Pairwise;AUTO_FORMAT=Semiauto;CDD_SEARCH=on;CLIENT=web;COMPOSITION_BASED_STATISTICS=on;DATABASE=nr;DESCRIPTIONS=100;ENTREZ_QUERY=%28none%29;EXPECT=10;FILTER=L;FORMAT_BLOCK_ON_RESPAGE=None;FORMAT_OBJECT=Alignment;FORMAT_TYPE=HTML;GAPCOSTS=11%201;I_THRESH=0.001;LAYOUT=TwoWindows;MATRIX_NAME=BLOSUM62;NCBI_GI=on;PAGE=Proteins;PROGRAM=blastp;QUERY=TMKPLSQTLCLCLVLALSHAHHHAGHQGGEDEGHEGHDHGHHEGLLLDRCQGIEMDAVAVTEEGIPYFFKGGHVFKGFHGKAELSNESFAELDDHHHLGHVDAAFLMHFPDKPTEHDHIFFMLDTKVFSYYKHQLETGFPKDISEVFPGIPDHLDAAVVCPAPDCEEDAVIFFKGDEIYHYNVKTKKVEEKKFEGMPNCTSAFRFMEHYYCFHGHQFSKFDPKTGEVHGRYPKEARDYFMKCSKFGDTTDHIERERCSRVHLDAITSDDAGNIYAFRGHHFLEQDAGNDTWAADTIESDFKELHSEVDATFSYENHLYMVKDDKVYIYKVGDSHTHLDGSPKPLKEVLGVEGPIDAAFVCQDHHIAHVIKGQTVYDVDLKASPPVPVKEGSFTLFNKVDAAMCGPEGVKLFKGNHYFHFQSVKVMLMAKAIPEEHKTALELFGCDH;SERVICE=plain;SET_DEFAULTS.x=9;SET_DEFAULTS.y=5;SHOW_OVERVIEW=on;WORD_SIZE=3;END_OF_HTTPGET=Yes).

**Search parameters**

| **MS data file:** | H:\MBA1-G6\35_BC11_01_1667.mgf |
| --- | --- |
| **Enzyme:** | Trypsin: cuts C-term side of KR unless next residue is P. |
| **Fixed modifications:** | [Carbamidomethyl (C)](http://fun-gen1.ibls.gla.ac.uk/mascot/cgi/client.pl?modification;file=..%2Fdata%2F20110930%2FF112288.dat;mod_name=Carbamidomethyl%20%28C%29) |
| **Variable modifications:** | [Oxidation (M)](http://fun-gen1.ibls.gla.ac.uk/mascot/cgi/client.pl?modification;file=..%2Fdata%2F20110930%2FF112288.dat;mod_name=Oxidation%20%28M%29) |

**Protein sequence coverage: 8%**

Matched peptides shown in ***bold red***.

| **1** | TMKPLSQTLC | LCLVLALSHA | HHHAGHQGGE | DEGHEGHDHG | HHEGLLLDRC |
| --- | --- | --- | --- | --- | --- |
| **51** | QGIEMDAVAV | TEEGIPYFFK | GGHVFKGFHG | KAELSNESFA | ELDDHHHLGH |
| **101** | VDAAFLMHFP | DKPTEHDHIF | FMLDTKVFSY | YKHQLETGFP | KDISEVFPGI |
| **151** | PDHLDAAVVC | PAPDCEEDAV | IFFKGDEIYH | YNVKTKKVEE | KKFEGMPNCT |
| **201** | SAFRFMEHYY | CFHGHQFSKF | DPKTGEVHGR | YPKEARDYFM | KCSK**FGDTTD** |
| **251** | **HIER**ERCSR**V** | **HLDAITSDDA** | **GNIYAFR**GHH | FLEQDAGNDT | WAADTIESDF |
| **301** | KELHSEVDAT | FSYENHLYMV | KDDKVYIYKV | GDSHTHLDGS | PKPLKEVLGV |
| **351** | EGPIDAAFVC | QDHHIAHVIK | **GQTVYDVDLK** | ASPPVPVKEG | SFTLFNKVDA |
| **401** | AMCGPEGVKL | FKGNHYFHFQ | SVKVMLMAKA | IPEEHKTALE | LFGCDH |

**SPOT # 568**

**Protein View: Q8AYG2_SALSA**

**Transferrin (Fragment).- Salmo salar (Atlantic salmon).**

Top of Form

| **Database:** | MSDB |
| --- | --- |
| **Score:** | 285 |
| **Nominal mass (M_r_):** | 8634 |
| **Calculated pI:** | 5.22 |
| **Taxonomy:** | [Salmo salar](http://www.ncbi.nlm.nih.gov/Taxonomy/Browser/wwwtax.cgi?lvl=0&id=8030) |

This protein sequence matches the following other entries:

- AAN17021 from [Salmo salar](http://www.ncbi.nlm.nih.gov/Taxonomy/Browser/wwwtax.cgi?lvl=0&id=8030)

Sequence similarity is available as [an NCBI BLAST search of Q8AYG2_SALSA against nr](http://www.ncbi.nlm.nih.gov/blast/Blast.cgi?ALIGNMENTS=50;ALIGNMENT_VIEW=Pairwise;AUTO_FORMAT=Semiauto;CDD_SEARCH=on;CLIENT=web;COMPOSITION_BASED_STATISTICS=on;DATABASE=nr;DESCRIPTIONS=100;ENTREZ_QUERY=%28none%29;EXPECT=10;FILTER=L;FORMAT_BLOCK_ON_RESPAGE=None;FORMAT_OBJECT=Alignment;FORMAT_TYPE=HTML;GAPCOSTS=11%201;I_THRESH=0.001;LAYOUT=TwoWindows;MATRIX_NAME=BLOSUM62;NCBI_GI=on;PAGE=Proteins;PROGRAM=blastp;QUERY=NDCDFTKYFSXGCAPGSEVGSPFCAQCKGSGKAXGXDEXRCKARSEEQYYGYTGAFRCLVEXAGDVAFIKHTIVPES;SERVICE=plain;SET_DEFAULTS.x=9;SET_DEFAULTS.y=5;SHOW_OVERVIEW=on;WORD_SIZE=3;END_OF_HTTPGET=Yes).

**Search parameters**

| **MS data file:** | H:\MBA1-G6\36_BC12_01_1668.mgf |
| --- | --- |
| **Enzyme:** | Trypsin: cuts C-term side of KR unless next residue is P. |
| **Fixed modifications:** | [Carbamidomethyl (C)](http://fun-gen1.ibls.gla.ac.uk/mascot/cgi/client.pl?modification;file=..%2Fdata%2F20110930%2FF112289.dat;mod_name=Carbamidomethyl%20%28C%29) |
| **Variable modifications:** | [Oxidation (M)](http://fun-gen1.ibls.gla.ac.uk/mascot/cgi/client.pl?modification;file=..%2Fdata%2F20110930%2FF112289.dat;mod_name=Oxidation%20%28M%29) |

**Protein sequence coverage: 36%**

Matched peptides shown in ***bold red***.

| **1** | NDCDFTKYFS | XGCAPGSEVG | SPFCAQCKGS | GKAXGXDEXR | CK**ARSEEQYY** |
| --- | --- | --- | --- | --- | --- |
| **51** | **GYTGAFRCLV** | **EXAGDVAFIK** | HTIVPES |  |  |

Bottom of Form

**SPOT # 394**

**Protein View: Q6P043_BRARE**

**Hypothetical protein aldoab.- Brachydanio rerio (Zebrafish) (Danio rerio).**

Top of Form

| **Database:** | MSDB |
| --- | --- |
| **Score:** | 231 |
| **Nominal mass (M_r_):** | 39956 |
| **Calculated pI:** | 8.48 |
| **Taxonomy:** | [Danio rerio](http://www.ncbi.nlm.nih.gov/Taxonomy/Browser/wwwtax.cgi?lvl=0&id=7955) |

This protein sequence matches the following other entries:

- AAH65847 from [Danio rerio](http://www.ncbi.nlm.nih.gov/Taxonomy/Browser/wwwtax.cgi?lvl=0&id=7955)

Sequence similarity is available as [an NCBI BLAST search of Q6P043_BRARE against nr](http://www.ncbi.nlm.nih.gov/blast/Blast.cgi?ALIGNMENTS=50;ALIGNMENT_VIEW=Pairwise;AUTO_FORMAT=Semiauto;CDD_SEARCH=on;CLIENT=web;COMPOSITION_BASED_STATISTICS=on;DATABASE=nr;DESCRIPTIONS=100;ENTREZ_QUERY=%28none%29;EXPECT=10;FILTER=L;FORMAT_BLOCK_ON_RESPAGE=None;FORMAT_OBJECT=Alignment;FORMAT_TYPE=HTML;GAPCOSTS=11%201;I_THRESH=0.001;LAYOUT=TwoWindows;MATRIX_NAME=BLOSUM62;NCBI_GI=on;PAGE=Proteins;PROGRAM=blastp;QUERY=MPHAYPFLSPDQKKELSDIAQRIVAPGKGILAADESTGSVAKRFQSINAENTEENRRLYRQLLFTADDRIKPCIGGVILFHETLYQKTDDGKLFSQLIKERGMVVGIKVDKGVVPLAGTNGETTTQGLDGLYERCAQYKKDGADFAKWRCVLKITPTTPSNLAIIENANVLARYASICQMHGIVPIVEPEILPDGDHDLKRCQYVTEKVLAAVYKALSDHHVYLEGTLLKPNMVTAGHSCSHKYSPQEIAMATVTALRRTVPPAVPGITFLSGGQSEEEASINLSTINQCPLSKPWALTFSYGRALQASALKAWGGKKENGKACQEEFVKRAVNNSAAAVGKYVSKGDTGAAAGESLFVANHAY;SERVICE=plain;SET_DEFAULTS.x=9;SET_DEFAULTS.y=5;SHOW_OVERVIEW=on;WORD_SIZE=3;END_OF_HTTPGET=Yes).

**Search parameters**

| **MS data file:** | H:\MBA1-G6\38_BD2_01_1670.mgf |
| --- | --- |
| **Enzyme:** | Trypsin: cuts C-term side of KR unless next residue is P. |
| **Fixed modifications:** | [Carbamidomethyl (C)](http://fun-gen1.ibls.gla.ac.uk/mascot/cgi/client.pl?modification;file=..%2Fdata%2F20110930%2FF112291.dat;mod_name=Carbamidomethyl%20%28C%29) |
| **Variable modifications:** | [Oxidation (M)](http://fun-gen1.ibls.gla.ac.uk/mascot/cgi/client.pl?modification;file=..%2Fdata%2F20110930%2FF112291.dat;mod_name=Oxidation%20%28M%29) |

**Protein sequence coverage: 20%**

Matched peptides shown in ***bold red***.

| **1** | MPHAYPFLSP | DQKKELSDIA | QRIVAPGK**GI** | **LAADESTGSV** | **AK**RFQSINAE |
| --- | --- | --- | --- | --- | --- |
| **51** | NTEENRRLYR | QLLFTADDRI | KPCIGGVILF | HETLYQKTDD | GKLFSQLIKE |
| **101** | RGMVVGIK**VD** | **KGVVPLAGTN** | **GETTTQGLDG** | **LYER**CAQYKK | DGADFAKWRC |
| **151** | VLKITPTTPS | NLAIIENANV | LAR**YASICQM** | **HGIVPIVEPE** | **ILPDGDHDLK** |
| **201** | **R**CQYVTEKVL | AAVYKALSDH | HVYLEGTLLK | PNMVTAGHSC | SHKYSPQEIA |
| **251** | MATVTALRRT | VPPAVPGITF | LSGGQSEEEA | SINLSTINQC | PLSKPWALTF |
| **301** | SYGR**ALQASA** | **LK**AWGGKKEN | GKACQEEFVK | RAVNNSAAAV | GKYVSKGDTG |
| **351** | AAAGESLFVA | NHAY |  |  |  |

Bottom of Form

**SPOT #499**

**Protein View: Q7ZVP5_BRARE**

**Proteasome (Prosome, macropain) 26S subunit, non-ATPase, 1.- Brachydanio rerio (Zebrafish) (Danio rerio).**

Top of Form

| **Database:** | MSDB |
| --- | --- |
| **Score:** | 35 |
| **Nominal mass (M_r_):** | 100524 |
| **Calculated pI:** | 9.20 |
| **Taxonomy:** | [Danio rerio](http://www.ncbi.nlm.nih.gov/Taxonomy/Browser/wwwtax.cgi?lvl=0&id=7955) |

This protein sequence matches the following other entries:

- AAH45463 from [Danio rerio](http://www.ncbi.nlm.nih.gov/Taxonomy/Browser/wwwtax.cgi?lvl=0&id=7955)

Sequence similarity is available as [an NCBI BLAST search of Q7ZVP5_BRARE against nr](http://www.ncbi.nlm.nih.gov/blast/Blast.cgi?ALIGNMENTS=50;ALIGNMENT_VIEW=Pairwise;AUTO_FORMAT=Semiauto;CDD_SEARCH=on;CLIENT=web;COMPOSITION_BASED_STATISTICS=on;DATABASE=nr;DESCRIPTIONS=100;ENTREZ_QUERY=%28none%29;EXPECT=10;FILTER=L;FORMAT_BLOCK_ON_RESPAGE=None;FORMAT_OBJECT=Alignment;FORMAT_TYPE=HTML;GAPCOSTS=11%201;I_THRESH=0.001;LAYOUT=TwoWindows;MATRIX_NAME=BLOSUM62;NCBI_GI=on;PAGE=Proteins;PROGRAM=blastp;QUERY=MITSAAGIISLLDEDEPQLKEFALHKLNSVVNDFWAEISESVGKIEVLYEDETFRSREFAALVASKVFYHLGAFDESLSYALGAGDLFNVNDDSEYVETIIAKCIDHYTKQRVENAELPEEEEKKDIDPRLEGIVNKMFQRCLGDHKYKQAIGIALETRRLDIFEKTILESNDIGGLLAYSLKICMSLMQNKKFRNEVLRVLVKLYMNLEKPDFINVCQCLIFLDDPQAVSDILEKLVKEDNLLMAYQICFDLYESASQQFLSSVIQNLRTVGTPIPAVPGSTNTGTVPTPDKDSDSMETEDKAGSSPAGKTADAKDEPKDQNSKMIKILSGEMAIELHLQFLIRNNNTDLMILKNTKDAVRNSVCHTATVIANSFMHTGTTSDQFLRENLEWLARATNWAKFTATASLGVIHKGHEKEALQLMATYLPKDTSPGSAYQEGGGLYALGLIHANHGGDIIDYLLSQLKNASNDIVRHGGALGLGLAALGTARQDVYDLLKSNLYQDDAVTGEAAGLALGLVMLGSKSAQAIEDMVGYAQETQHEKILRGLAVGIAMVMYGRMEEADALIESLCRDKDPILRRSGMYTVAMAYCGSGNNKAIRRLLHVAVSDVNDDVRRAAVESIGFIMFRTPEQCPSVVSLLSESYNPHVRYGAAMALGICCAGTGNKEAIHLLEPMTNDPVNYVRQGALIASALIMIQQTEVTCPKVNQFRQLYSKVINDKHDDVMAKFGAILAQGVLDAGGRNVTISLQSRTGHTHMPSVVGLLVFTQFWFWFPLSHFLSLAFTPTAIIGLNKDLKMPKVQYRSNCKPSTLPTLHPWKCQRRRKRRRCPPPSCPSLLKPRRRRRRRKRRRRKRWKWKHRRRLRKRRKTRRRRKKRKRRKSQSQTSR;SERVICE=plain;SET_DEFAULTS.x=9;SET_DEFAULTS.y=5;SHOW_OVERVIEW=on;WORD_SIZE=3;END_OF_HTTPGET=Yes).

**Search parameters**

| **MS data file:** | H:\MBA1-G6\39_BD3_01_1671.mgf |
| --- | --- |
| **Enzyme:** | Trypsin: cuts C-term side of KR unless next residue is P. |
| **Fixed modifications:** | [Carbamidomethyl (C)](http://fun-gen1.ibls.gla.ac.uk/mascot/cgi/client.pl?modification;file=..%2Fdata%2F20110930%2FF112292.dat;mod_name=Carbamidomethyl%20%28C%29) |
| **Variable modifications:** | [Oxidation (M)](http://fun-gen1.ibls.gla.ac.uk/mascot/cgi/client.pl?modification;file=..%2Fdata%2F20110930%2FF112292.dat;mod_name=Oxidation%20%28M%29) |

**Protein sequence coverage: 1%**

Matched peptides shown in ***bold red***.

| **1** | MITSAAGIIS | LLDEDEPQLK | EFALHKLNSV | VNDFWAEISE | SVGKIEVLYE |
| --- | --- | --- | --- | --- | --- |
| **51** | DETFRSREFA | ALVASKVFYH | LGAFDESLSY | ALGAGDLFNV | NDDSEYVETI |
| **101** | IAKCIDHYTK | QRVENAELPE | EEEKKDIDPR | LEGIVNKMFQ | RCLGDHKYKQ |
| **151** | AIGIALETRR | LDIFEKTILE | SNDIGGLLAY | SLKICMSLMQ | NKKFRNEVLR |
| **201** | VLVKLYMNLE | KPDFINVCQC | LIFLDDPQAV | SDILEKLVKE | DNLLMAYQIC |
| **251** | FDLYESASQQ | FLSSVIQNLR | TVGTPIPAVP | GSTNTGTVPT | PDKDSDSMET |
| **301** | EDKAGSSPAG | KTADAKDEPK | DQNSKMIKIL | SGEMAIELHL | QFLIR**NNNTD** |
| **351** | **LMILK**NTKDA | VRNSVCHTAT | VIANSFMHTG | TTSDQFLREN | LEWLARATNW |
| **401** | AKFTATASLG | VIHKGHEKEA | LQLMATYLPK | DTSPGSAYQE | GGGLYALGLI |
| **451** | HANHGGDIID | YLLSQLKNAS | NDIVRHGGAL | GLGLAALGTA | RQDVYDLLKS |
| **501** | NLYQDDAVTG | EAAGLALGLV | MLGSKSAQAI | EDMVGYAQET | QHEKILRGLA |
| **551** | VGIAMVMYGR | MEEADALIES | LCRDKDPILR | RSGMYTVAMA | YCGSGNNKAI |
| **601** | RRLLHVAVSD | VNDDVRRAAV | ESIGFIMFRT | PEQCPSVVSL | LSESYNPHVR |
| **651** | YGAAMALGIC | CAGTGNKEAI | HLLEPMTNDP | VNYVRQGALI | ASALIMIQQT |
| **701** | EVTCPKVNQF | RQLYSKVIND | KHDDVMAKFG | AILAQGVLDA | GGRNVTISLQ |
| **751** | SRTGHTHMPS | VVGLLVFTQF | WFWFPLSHFL | SLAFTPTAII | GLNKDLKMPK |
| **801** | VQYRSNCKPS | TLPTLHPWKC | QRRRKRRRCP | PPSCPSLLKP | RRRRRRRKRR |
| **851** | RRKRWKWKHR | RRLRKRRKTR | RRRKKRKRRK | SQSQTSR |  |

**SPOT #642**

**Protein View: JH0472**

**apolipoprotein A-I precursor - Atlantic salmon**

Top of Form

| **Database:** | MSDB |
| --- | --- |
| **Score:** | 435 |
| **Nominal mass (M_r_):** | 29557 |
| **Calculated pI:** | 8.48 |
| **Taxonomy:** | [Salmo salar](http://www.ncbi.nlm.nih.gov/Taxonomy/Browser/wwwtax.cgi?lvl=0&id=8030) |

Sequence similarity is available as [an NCBI BLAST search of JH0472 against nr](http://www.ncbi.nlm.nih.gov/blast/Blast.cgi?ALIGNMENTS=50;ALIGNMENT_VIEW=Pairwise;AUTO_FORMAT=Semiauto;CDD_SEARCH=on;CLIENT=web;COMPOSITION_BASED_STATISTICS=on;DATABASE=nr;DESCRIPTIONS=100;ENTREZ_QUERY=%28none%29;EXPECT=10;FILTER=L;FORMAT_BLOCK_ON_RESPAGE=None;FORMAT_OBJECT=Alignment;FORMAT_TYPE=HTML;GAPCOSTS=11%201;I_THRESH=0.001;LAYOUT=TwoWindows;MATRIX_NAME=BLOSUM62;NCBI_GI=on;PAGE=Proteins;PROGRAM=blastp;QUERY=MKFLVLALTILLAAGTQAFPMQADAPSQLEHVKAALNMYIAQVKLTAQRSIDLLDDTEYKEYKMQLSQSLDNLQQFADSTSKSWPPTPRSSAPSCDATATVRAEVMKDVEDVRTQLEPKRAELREVLNKHIDEYRKKLEPLIKEHIELRRTEMDAFRAKIEPVVEEMRAKVAVNVEETKTKLMPIVEIVRAKLTERLEELRTLAAPYAEEYKEQMFKAVGEVREKVAPLSEDFKARWAPPPRRPSKSSWLSTRPSARP;SERVICE=plain;SET_DEFAULTS.x=9;SET_DEFAULTS.y=5;SHOW_OVERVIEW=on;WORD_SIZE=3;END_OF_HTTPGET=Yes).

**Search parameters**

| **MS data file:** | H:\MBA1-G6\40_BD4_01_1672.mgf |
| --- | --- |
| **Enzyme:** | Trypsin: cuts C-term side of KR unless next residue is P. |
| **Fixed modifications:** | [Carbamidomethyl (C)](http://fun-gen1.ibls.gla.ac.uk/mascot/cgi/client.pl?modification;file=..%2Fdata%2F20110930%2FF112293.dat;mod_name=Carbamidomethyl%20%28C%29) |
| **Variable modifications:** | [Oxidation (M)](http://fun-gen1.ibls.gla.ac.uk/mascot/cgi/client.pl?modification;file=..%2Fdata%2F20110930%2FF112293.dat;mod_name=Oxidation%20%28M%29) |

**Protein sequence coverage: 37%**

Matched peptides shown in ***bold red***.

| **1** | MKFLVLALTI | LLAAGTQAFP | MQADAPSQLE | HVK**AALNMYI** | **AQVK**LTAQR**S** |
| --- | --- | --- | --- | --- | --- |
| **51** | **IDLLDDTEYK** | EYK**MQLSQSL** | **DNLQQFADST** | **SK**SWPPTPRS | SAPSCDATAT |
| **101** | VRAEVMKDVE | DVRTQLEPKR | AELREVLNKH | IDEYRKKLEP | LIKEHIELRR |
| **151** | **TEMDAFRAKI** | **EPVVEEMR**AK | **VAVNVEETKT** | **K**LMPIVEIVR | AKLTERLEEL |
| **201** | R**TLAAPYAEE** | **YKEQMFK**AVG | EVR**EKVAPLS** | **EDFK**ARWAPP | PRRPSKSSWL |
| **251** | STRPSARP |  |  |  |  |

Bottom of Form

**SPOT # 32**

**C1 inhibitor precursor.- Oncorhynchus mykiss (Rainbow trout) (Salmo gairdneri).**

Top of Form

| **Database:** | MSDB |
| --- | --- |
| **Score:** | 137 |
| **Nominal mass (M_r_):** | 69065 |
| **Calculated pI:** | 5.62 |
| **Taxonomy:** | [Oncorhynchus mykiss](http://www.ncbi.nlm.nih.gov/Taxonomy/Browser/wwwtax.cgi?lvl=0&id=8022) |

This protein sequence matches the following other entries:

- CAD58653 from [Oncorhynchus mykiss](http://www.ncbi.nlm.nih.gov/Taxonomy/Browser/wwwtax.cgi?lvl=0&id=8022)

Sequence similarity is available as [an NCBI BLAST search of Q70W32_ONCMY against nr](http://www.ncbi.nlm.nih.gov/blast/Blast.cgi?ALIGNMENTS=50;ALIGNMENT_VIEW=Pairwise;AUTO_FORMAT=Semiauto;CDD_SEARCH=on;CLIENT=web;COMPOSITION_BASED_STATISTICS=on;DATABASE=nr;DESCRIPTIONS=100;ENTREZ_QUERY=%28none%29;EXPECT=10;FILTER=L;FORMAT_BLOCK_ON_RESPAGE=None;FORMAT_OBJECT=Alignment;FORMAT_TYPE=HTML;GAPCOSTS=11%201;I_THRESH=0.001;LAYOUT=TwoWindows;MATRIX_NAME=BLOSUM62;NCBI_GI=on;PAGE=Proteins;PROGRAM=blastp;QUERY=MGISVLSCVSLLLVFELFSSSVGVLQSVPGSTLVFPCLPGQNQKSFAGAKITWKYNDYLVPDYPESSKELKASKDGFYLEISPVSVANEGEYECVIKQNDMEWQKVHIVQVDVSSSYILKVIEGSTVNLPCDRPPSSKDPVHWYRYDKGMTNGKRKQLNPAETGEMVEGDRLEWLYGPLEKDMTITLNEVKMEDAGMYYCETAEQGRDSSNFNTIELIVEAAPTVLPYSCVGFMTPWESRQDETSRPWEAMLGESLNEFSMKLYAHLSQSQPMKNLLFSPISISGVLTHLLLGARGKTRRDMETALCLSHDFFCVHSEMKKLKLKLQDTLKMASQIYYNPNMKLSESFTNQSMQFYDADPVKLTNTSEVNVEMINSWVAKQTNNKIKELVDSVPAHTELLLLNTVYFNGQWKMKFDAKSKTFPFVKLNGDTVKVPVLYSAKYKLAVQYVPAVKAQVAMFPLSGASSLFILLPPTTKLTDLQLVEGKMTDRAVSQMVEQMNQVSPQATEVTLPKIKLDIRTEMNTLLRKIGLSELFDSANLCGLYPDNELLLTEARHRAFLSLTEEGVEAGAATSLSFSRSFSSFSALRPFLMILWSDQAKVPLFVGRVTEP;SERVICE=plain;SET_DEFAULTS.x=9;SET_DEFAULTS.y=5;SHOW_OVERVIEW=on;WORD_SIZE=3;END_OF_HTTPGET=Yes).

**Search parameters**

| **MS data file:** | H:\MBA1-G6\41_BD5_01_1673.mgf |
| --- | --- |
| **Enzyme:** | Trypsin: cuts C-term side of KR unless next residue is P. |
| **Fixed modifications:** | [Carbamidomethyl (C)](http://fun-gen1.ibls.gla.ac.uk/mascot/cgi/client.pl?modification;file=..%2Fdata%2F20110930%2FF112294.dat;mod_name=Carbamidomethyl%20%28C%29) |
| **Variable modifications:** | [Oxidation (M)](http://fun-gen1.ibls.gla.ac.uk/mascot/cgi/client.pl?modification;file=..%2Fdata%2F20110930%2FF112294.dat;mod_name=Oxidation%20%28M%29) |

**Protein sequence coverage: 14%**

Matched peptides shown in ***bold red***.

| **1** | MGISVLSCVS | LLLVFELFSS | SVGVLQSVPG | STLVFPCLPG | QNQKSFAGAK |
| --- | --- | --- | --- | --- | --- |
| **51** | ITWKYNDYLV | PDYPESSKEL | KASKDGFYLE | ISPVSVANEG | EYECVIKQND |
| **101** | MEWQKVHIVQ | VDVSSSYILK | **VIEGSTVNLP** | **CDRPPSSK**DP | VHWYRYDKGM |
| **151** | TNGKRKQLNP | AETGEMVEGD | RLEWLYGPLE | KDMTITLNEV | K**MEDAGMYYC** |
| **201** | **ETAEQGR**DSS | NFNTIELIVE | AAPTVLPYSC | VGFMTPWESR | QDETSRPWEA |
| **251** | MLGESLNEFS | MK**LYAHLSQS** | **QPMK**NLLFSP | ISISGVLTHL | LLGARGKTRR |
| **301** | DMETALCLSH | DFFCVHSEMK | KLKLKLQDTL | K**MASQIYYNP** | **NMK**LSESFTN |
| **351** | QSMQFYDADP | VKLTNTSEVN | VEMINSWVAK | QTNNKIKELV | DSVPAHTELL |
| **401** | LLNTVYFNGQ | WKMKFDAKSK | TFPFVKLNGD | TVK**VPVLYSA** | **K**YKLAVQYVP |
| **451** | AVKAQVAMFP | LSGASSLFIL | LPPTTKLTDL | QLVEGKMTDR | **AVSQMVEQMN** |
| **501** | **QVSPQATEVT** | **LPK**IKLDIRT | EMNTLLRKIG | LSELFDSANL | CGLYPDNELL |
| **551** | LTEARHRAFL | SLTEEGVEAG | AATSLSFSRS | FSSFSALRPF | LMILWSDQAK |
| **601** | VPLFVGRVTE | P |  |  |  |

Bottom of Form

**SPOT # 509**

**Protein View: TRF2_SALSA**

**Serotransferrin II precursor (Siderophilin II) (STF II).- Salmo salar (Atlantic salmon).**

Top of Form

| **Database:** | MSDB |
| --- | --- |
| **Score:** | 250 |
| **Nominal mass (M_r_):** | 76497 |
| **Calculated pI:** | 7.08 |
| **Taxonomy:** | [Salmo salar](http://www.ncbi.nlm.nih.gov/Taxonomy/Browser/wwwtax.cgi?lvl=0&id=8030) |

Sequence similarity is available as [an NCBI BLAST search of TRF2_SALSA against nr](http://www.ncbi.nlm.nih.gov/blast/Blast.cgi?ALIGNMENTS=50;ALIGNMENT_VIEW=Pairwise;AUTO_FORMAT=Semiauto;CDD_SEARCH=on;CLIENT=web;COMPOSITION_BASED_STATISTICS=on;DATABASE=nr;DESCRIPTIONS=100;ENTREZ_QUERY=%28none%29;EXPECT=10;FILTER=L;FORMAT_BLOCK_ON_RESPAGE=None;FORMAT_OBJECT=Alignment;FORMAT_TYPE=HTML;GAPCOSTS=11%201;I_THRESH=0.001;LAYOUT=TwoWindows;MATRIX_NAME=BLOSUM62;NCBI_GI=on;PAGE=Proteins;PROGRAM=blastp;QUERY=MKLLLLSALLGCLATAYAAPAEGIVKWCVKSEQELRKCHDLAAKVAEFSCVRKDGSFECIQAIKGGEADAITLDGGDIYTAGLTNYGLQPIIAEDYGEDSDTCYYAVAVAKKGTAFGFKTLRGKKSCHTGLGKSAGWNIPIGTLVTESQIRWAGIEDRPVESAVSDFFNASCAPGATMGSKLCQLCKGDCSRSHKEPYYDYAGAFQCLKDGAGDVAFIKPLAVPAAEKASYELLCKDGTRASIDSYKTCHLARVPAHAVVSRKDPELANRIYNKLVAVKDFNLFSSDGYAAKNLMFKDSAQKLVQLPTTTDSFLYLGAEYMSTIRSLKKSQATGASSRAIKWCAVGHAEKGKCDTWTINSFADGESKISCQDAPTVEECIKKIMRKEADAIAVDGGEVYTAGKCGLVPVMVEQYDADLCSAPGEASSYYAVAVAKKGSGLTWKTLKGKRSCHTGLGRTAGWNIPMGLIHQETNDCDFTKYFSKGCAPGSEVGSPFCAQCKGSGKARGGDEDRCKARSEEQYYGYTGAFRCLVEDAGDVAFIKHTIVPESTDGNGPDWAKDLKSSDFELLCQDGTTQPVTKFSECHLAKVPAHAVITRPETRGDVVSILLELQAKFGSSGSDSSFRMFQSSVEKNLLFKDSTKCLQEIPKGTKYQDFLGKEYMIAMQSLRKCSDSTSDLEKACTFHSCQQKE;SERVICE=plain;SET_DEFAULTS.x=9;SET_DEFAULTS.y=5;SHOW_OVERVIEW=on;WORD_SIZE=3;END_OF_HTTPGET=Yes).

**Search parameters**

| **MS data file:** | H:\MBA1-G6\42_BD6_01_1674.mgf |
| --- | --- |
| **Enzyme:** | Trypsin: cuts C-term side of KR unless next residue is P. |
| **Fixed modifications:** | [Carbamidomethyl (C)](http://fun-gen1.ibls.gla.ac.uk/mascot/cgi/client.pl?modification;file=..%2Fdata%2F20110930%2FF112295.dat;mod_name=Carbamidomethyl%20%28C%29) |
| **Variable modifications:** | [Oxidation (M)](http://fun-gen1.ibls.gla.ac.uk/mascot/cgi/client.pl?modification;file=..%2Fdata%2F20110930%2FF112295.dat;mod_name=Oxidation%20%28M%29) |

**Protein sequence coverage: 18%**

Matched peptides shown in ***bold red***.

| **1** | MKLLLLSALL | GCLATAYAAP | AEGIVKWCVK | SEQELRKCHD | LAAKVAEFSC |
| --- | --- | --- | --- | --- | --- |
| **51** | VRKDGSFECI | QAIKGGEADA | ITLDGGDIYT | AGLTNYGLQP | IIAEDYGEDS |
| **101** | DTCYYAVAVA | K**KGTAFGFK**T | LRGKKSCHTG | LGKSAGWNIP | IGTLVTESQI |
| **151** | RWAGIEDRPV | ESAVSDFFNA | SCAPGATMGS | KLCQLCKGDC | SR**SHKEPYYD** |
| **201** | **YAGAFQCLKD** | **GAGDVAFIKP** | **LAVPAAEK**AS | YELLCKDGTR | ASIDSYKTCH |
| **251** | LARVPAHAVV | SRKDPELANR | IYNKLVAVK**D** | **FNLFSSDGYA** | **AK**NLMFKDSA |
| **301** | QKLVQLPTTT | DSFLYLGAEY | MSTIRSLKKS | QATGASSRAI | KWCAVGHAEK |
| **351** | GKCDTWTINS | FADGESKISC | QDAPTVEECI | KKIMRKEADA | IAVDGGEVYT |
| **401** | AGKCGLVPVM | VEQYDADLCS | APGEASSYYA | VAVAKKGSGL | TWKTLKGKRS |
| **451** | CHTGLGRTAG | WNIPMGLIHQ | ETNDCDFTKY | FSKGCAPGSE | VGSPFCAQCK |
| **501** | GSGKARGGDE | DRCKAR**SEEQ** | **YYGYTGAFRC** | **LVEDAGDVAF** | **IK**HTIVPEST |
| **551** | DGNGPDWAKD | LKSSDFELLC | QDGTTQPVTK | FSECHLAK**VP** | **AHAVITRPET** |
| **601** | **RGDVVSILLE** | **LQAK**FGSSGS | DSSFRMFQSS | VEKNLLFKDS | TK**CLQEIPK**G |
| **651** | TKYQDFLGK**E** | **YMIAMQSLR**K | CSDSTSDLEK | ACTFHSCQQK | E |

**SPOT # 472**

**Protein View: ABONS2**

**serum albumin 2 precursor - Atlantic salmon**

Top of Form

| **Database:** | MSDB |
| --- | --- |
| **Score:** | 323 |
| **Nominal mass (M_r_):** | 69124 |
| **Calculated pI:** | 5.44 |
| **Taxonomy:** | [Salmo salar](http://www.ncbi.nlm.nih.gov/Taxonomy/Browser/wwwtax.cgi?lvl=0&id=8030) |

This protein sequence matches the following other entries:

- CAA43187 from [Salmo salar](http://www.ncbi.nlm.nih.gov/Taxonomy/Browser/wwwtax.cgi?lvl=0&id=8030)
- ALBU2_SALSA from [Salmo salar](http://www.ncbi.nlm.nih.gov/Taxonomy/Browser/wwwtax.cgi?lvl=0&id=8030)

Sequence similarity is available as [an NCBI BLAST search of ABONS2 against nr](http://www.ncbi.nlm.nih.gov/blast/Blast.cgi?ALIGNMENTS=50;ALIGNMENT_VIEW=Pairwise;AUTO_FORMAT=Semiauto;CDD_SEARCH=on;CLIENT=web;COMPOSITION_BASED_STATISTICS=on;DATABASE=nr;DESCRIPTIONS=100;ENTREZ_QUERY=%28none%29;EXPECT=10;FILTER=L;FORMAT_BLOCK_ON_RESPAGE=None;FORMAT_OBJECT=Alignment;FORMAT_TYPE=HTML;GAPCOSTS=11%201;I_THRESH=0.001;LAYOUT=TwoWindows;MATRIX_NAME=BLOSUM62;NCBI_GI=on;PAGE=Proteins;PROGRAM=blastp;QUERY=MQWLSVCSLLVLLSVLSRSQAQNQICTIFTEAKEDGFKSLILVGLAQNLPDSTLGDLVPLIAEALAMGVKCCSDTPPEDCERDVADLFQSAVCSSETLVEKNDLKMCCEKTAAERTHCFVDHKAKIPRDLSLKAELPAADQCEDFKKDHKAFVGRFIFKFSKSNPMLPPHVVLAIAKGYGEVLTTCCGEAEAQTCFDTKKATFQHAIAKRVAELKSLCIVHKKYGDRVVKAKKLVQYSQKMPQASFQEMAGMVDKIVATVAPCCSGDMVTCMKERKTLVDEVCADESVLSRAAGLSACCKEDAVHRGSCVEAMKPDPKPDGLSEHYDVHADIAAVCQTFTKTPDVAMGKLVYEISVRHPESSQQVILRFAKEAEQALLQCCDMEDHAECVKTALAGSDIDKKITDETDYYKKMCAAEAAVSDDNFEKSMMVYYTRIMPQASFDQLHMVSETVHDVLHACCKDEPGHFVLPCAEEKLTDAIDATCDDYDPSSINPHIAHCCNQSYSMRRHCILAIQPDTEFTPPELDASSFHMGPELCTKDSKDLLLSGKKLLYGVVRHKTTITEDHLKTISTKYHTMKDKCCAAEDQAACFTEEAPKLVSESAELVKV;SERVICE=plain;SET_DEFAULTS.x=9;SET_DEFAULTS.y=5;SHOW_OVERVIEW=on;WORD_SIZE=3;END_OF_HTTPGET=Yes).

**Search parameters**

| **MS data file:** | H:\MBA1-G6\43_BD7_01_1675.mgf |
| --- | --- |
| **Enzyme:** | Trypsin: cuts C-term side of KR unless next residue is P. |
| **Fixed modifications:** | [Carbamidomethyl (C)](http://fun-gen1.ibls.gla.ac.uk/mascot/cgi/client.pl?modification;file=..%2Fdata%2F20110930%2FF112296.dat;mod_name=Carbamidomethyl%20%28C%29) |
| **Variable modifications:** | [Oxidation (M)](http://fun-gen1.ibls.gla.ac.uk/mascot/cgi/client.pl?modification;file=..%2Fdata%2F20110930%2FF112296.dat;mod_name=Oxidation%20%28M%29) |

**Protein sequence coverage: 19%**

Matched peptides shown in ***bold red***.

| **1** | MQWLSVCSLL | VLLSVLSRSQ | AQNQICTIFT | EAKEDGFKSL | ILVGLAQNLP |
| --- | --- | --- | --- | --- | --- |
| **51** | DSTLGDLVPL | IAEALAMGVK | CCSDTPPEDC | ERDVADLFQS | AVCSSETLVE |
| **101** | KNDLKMCCEK | TAAERTHCFV | DHKAKIPRDL | SLKAELPAAD | QCEDFKKDHK |
| **151** | AFVGRFIFKF | SKSNPMLPPH | VVLAIAKGYG | EVLTTCCGEA | EAQTCFDTKK |
| **201** | ATFQHAIAKR | VAELKSLCIV | HKKYGDRVVK | AKKLVQYSQK | **MPQASFQEMA** |
| **251** | **GMVDKIVATV** | **APCCSGDMVT** | **CMK**ERKTLVD | EVCADESVLS | RAAGLSACCK |
| **301** | EDAVHRGSCV | EAMKPDPKPD | GLSEHYDVHA | DIAAVCQTFT | KTPDVAMGKL |
| **351** | VYEISVR**HPE** | **SSQQVILR**FA | KEAEQALLQC | CDMEDHAECV | KTALAGSDID |
| **401** | KK**ITDETDYY** | **KKMCAAEAAV** | **SDDNFEK**SMM | VYYTRIMPQA | SFDQLHMVSE |
| **451** | TVHDVLHACC | K**DEPGHFVLP** | **CAEEK**LTDAI | DATCDDYDPS | SINPHIAHCC |
| **501** | NQSYSMRRHC | ILAIQPDTEF | TPPELDASSF | HMGPELCTKD | SKDLLLSGKK |
| **551** | **LLYGVVR**HKT | TITEDHLKTI | STKYHTMKDK | **CCAAEDQAAC** | **FTEEAPKLVS** |
| **601** | **ESAELVKV** |  |  |  |  |

**SPOT # 442**Bottom of Form

**Protein View: Q98SS7_GADMO**

**Creatine kinase (Fragment).- Gadus morhua (Atlantic cod).**

Top of Form

| **Database:** | MSDB |
| --- | --- |
| **Score:** | 218 |
| **Nominal mass (M_r_):** | 29122 |
| **Calculated pI:** | 8.89 |
| **Taxonomy:** | [Gadus morhua](http://www.ncbi.nlm.nih.gov/Taxonomy/Browser/wwwtax.cgi?lvl=0&id=8049) |

This protein sequence matches the following other entries:

- AAK17970 from [Gadus morhua](http://www.ncbi.nlm.nih.gov/Taxonomy/Browser/wwwtax.cgi?lvl=0&id=8049)

Sequence similarity is available as [an NCBI BLAST search of Q98SS7_GADMO against nr](http://www.ncbi.nlm.nih.gov/blast/Blast.cgi?ALIGNMENTS=50;ALIGNMENT_VIEW=Pairwise;AUTO_FORMAT=Semiauto;CDD_SEARCH=on;CLIENT=web;COMPOSITION_BASED_STATISTICS=on;DATABASE=nr;DESCRIPTIONS=100;ENTREZ_QUERY=%28none%29;EXPECT=10;FILTER=L;FORMAT_BLOCK_ON_RESPAGE=None;FORMAT_OBJECT=Alignment;FORMAT_TYPE=HTML;GAPCOSTS=11%201;I_THRESH=0.001;LAYOUT=TwoWindows;MATRIX_NAME=BLOSUM62;NCBI_GI=on;PAGE=Proteins;PROGRAM=blastp;QUERY=AYVLSSRVRTGRSIKGFTLPPHNSRGERRTIEKLSIEALATLSGEFKGKYYPLNGMTDKEQDQLINDHFLFDKPVSPLLTCAGMARDWPDARGIWHNDAKTFLVWVNEEDHLRVISMQQGGNMKEVFRRFCTGLLKIEETFKKHNHGFMWNEHLGYVLTCPSNLGTGLRGGVHVKLPKLSTHAKFEEILTRLRLQKRGTGGVDTASVGGVFDISNADRLGSSEVDQVQMVVDGVKLMVAMEKKPEKSESIDDMIPAQK;SERVICE=plain;SET_DEFAULTS.x=9;SET_DEFAULTS.y=5;SHOW_OVERVIEW=on;WORD_SIZE=3;END_OF_HTTPGET=Yes).

**Search parameters**

| **MS data file:** | H:\MBA1-G6\44_BD8_01_1676.mgf |
| --- | --- |
| **Enzyme:** | Trypsin: cuts C-term side of KR unless next residue is P. |
| **Fixed modifications:** | [Carbamidomethyl (C)](http://fun-gen1.ibls.gla.ac.uk/mascot/cgi/client.pl?modification;file=..%2Fdata%2F20110930%2FF112297.dat;mod_name=Carbamidomethyl%20%28C%29) |
| **Variable modifications:** | [Oxidation (M)](http://fun-gen1.ibls.gla.ac.uk/mascot/cgi/client.pl?modification;file=..%2Fdata%2F20110930%2FF112297.dat;mod_name=Oxidation%20%28M%29) |

**Protein sequence coverage: 17%**

Matched peptides shown in ***bold red***.

| **1** | AYVLSSRVRT | GRSIKGFTLP | PHNSRGERRT | IEKLSIEALA | TLSGEFKGKY |
| --- | --- | --- | --- | --- | --- |
| **51** | YPLNGMTDKE | QDQLINDHFL | FDKPVSPLLT | CAGMARDWPD | ARGIWHNDAK |
| **101** | TFLVWVNEED | HLRVISMQQG | GNMKEVFRRF | CTGLLKIEET | FKKHNHGFMW |
| **151** | NEHLGYVLTC | PSNLGTGLRG | GVHVKLPKLS | THAK**FEEILT** | **R**LRLQKR**GTG** |
| **201** | **GVDTASVGGV** | **FDISNADRLG** | **SSEVDQVQMV** | **VDGVK**LMVAM | EKKPEKSESI |
| **251** | DDMIPAQK |  |  |  |  |

**SPOT # 608**

**Protein View: Q70I40_ONCMY**

**Triosephosphate isomerase (Fragment).- Oncorhynchus mykiss (Rainbow trout) (Salmo gairdneri).**

Top of Form

| **Database:** | MSDB |
| --- | --- |
| **Score:** | 170 |
| **Nominal mass (M_r_):** | 22759 |
| **Calculated pI:** | 6.59 |
| **Taxonomy:** | [Oncorhynchus mykiss](http://www.ncbi.nlm.nih.gov/Taxonomy/Browser/wwwtax.cgi?lvl=0&id=8022) |

This protein sequence matches the following other entries:

- CAE45565 from [Oncorhynchus mykiss](http://www.ncbi.nlm.nih.gov/Taxonomy/Browser/wwwtax.cgi?lvl=0&id=8022)

Sequence similarity is available as [an NCBI BLAST search of Q70I40_ONCMY against nr](http://www.ncbi.nlm.nih.gov/blast/Blast.cgi?ALIGNMENTS=50;ALIGNMENT_VIEW=Pairwise;AUTO_FORMAT=Semiauto;CDD_SEARCH=on;CLIENT=web;COMPOSITION_BASED_STATISTICS=on;DATABASE=nr;DESCRIPTIONS=100;ENTREZ_QUERY=%28none%29;EXPECT=10;FILTER=L;FORMAT_BLOCK_ON_RESPAGE=None;FORMAT_OBJECT=Alignment;FORMAT_TYPE=HTML;GAPCOSTS=11%201;I_THRESH=0.001;LAYOUT=TwoWindows;MATRIX_NAME=BLOSUM62;NCBI_GI=on;PAGE=Proteins;PROGRAM=blastp;QUERY=GDKASLGELIKTLNSAKLDPNTEVVCGAPSIYLEFARAKLDPKIGVAAQNCYKVKGGAFTGEISPAMIKDVGVHWVILGHSERRWVFGETDELIGQKCAHALENGLGVIACIGEKLDEREAGITEKVINAQTKHFADNIKDWSKVVLAYEPVWAIGTGKTASPAQAQDVHDKLRQWVKANVSEAVANSVRIIYGGSVTGGTCKELGGMKD;SERVICE=plain;SET_DEFAULTS.x=9;SET_DEFAULTS.y=5;SHOW_OVERVIEW=on;WORD_SIZE=3;END_OF_HTTPGET=Yes).

**Search parameters**

| **MS data file:** | H:\MBA1-G6\45_BD9_01_1677.mgf |
| --- | --- |
| **Enzyme:** | Trypsin: cuts C-term side of KR unless next residue is P. |
| **Fixed modifications:** | [Carbamidomethyl (C)](http://fun-gen1.ibls.gla.ac.uk/mascot/cgi/client.pl?modification;file=..%2Fdata%2F20110930%2FF112298.dat;mod_name=Carbamidomethyl%20%28C%29) |
| **Variable modifications:** | [Oxidation (M)](http://fun-gen1.ibls.gla.ac.uk/mascot/cgi/client.pl?modification;file=..%2Fdata%2F20110930%2FF112298.dat;mod_name=Oxidation%20%28M%29) |

**Protein sequence coverage: 22%**

Matched peptides shown in ***bold red***.

| **1** | GDK**ASLGELI** | **K**TLNSAKLDP | NTEVVCGAPS | IYLEFARAKL | DPKIGVAAQN |
| --- | --- | --- | --- | --- | --- |
| **51** | CYKVKGGAFT | GEISPAMIKD | VGVHWVILGH | SERRWVFGET | DELIGQKCAH |
| **101** | ALENGLGVIA | CIGEKLDERE | AGITEKVINA | QTKHFADNIK | DWSKVVLAYE |
| **151** | PVWAIGTGK**T** | **ASPAQAQDVH** | **DKLR**QWVK**AN** | **VSEAVANSVR** | **IIYGGSVTGG** |
| **201** | **TCK**ELGGMKD |  |  |  |  |

**SPOT # 444**

**Protein View: CO3_ONCMY**

**Complement C3-1 [Contains: Complement C3 beta chain; Complement C3 alpha chain; C3a anaphylatoxin; Complement C3b alpha' chain; Complement C3c fragment; Complement C3dg fragment; Complement C3g fragment; Complement C3d fragment; Complement C3f fragm**

Top of Form

| **Database:** | MSDB |
| --- | --- |
| **Score:** | 156 |
| **Nominal mass (M_r_):** | 183586 |
| **Calculated pI:** | 6.12 |
| **Taxonomy:** | [Oncorhynchus mykiss](http://www.ncbi.nlm.nih.gov/Taxonomy/Browser/wwwtax.cgi?lvl=0&id=8022) |

Sequence similarity is available as [an NCBI BLAST search of CO3_ONCMY against nr](http://www.ncbi.nlm.nih.gov/blast/Blast.cgi?ALIGNMENTS=50;ALIGNMENT_VIEW=Pairwise;AUTO_FORMAT=Semiauto;CDD_SEARCH=on;CLIENT=web;COMPOSITION_BASED_STATISTICS=on;DATABASE=nr;DESCRIPTIONS=100;ENTREZ_QUERY=%28none%29;EXPECT=10;FILTER=L;FORMAT_BLOCK_ON_RESPAGE=None;FORMAT_OBJECT=Alignment;FORMAT_TYPE=HTML;GAPCOSTS=11%201;I_THRESH=0.001;LAYOUT=TwoWindows;MATRIX_NAME=BLOSUM62;NCBI_GI=on;PAGE=Proteins;PROGRAM=blastp;QUERY=CO3_ONCMY;SERVICE=plain;SET_DEFAULTS.x=21;SET_DEFAULTS.y=7;SHOW_OVERVIEW=on;WORD_SIZE=3;END_OF_HTTPGET=Yes).

**Search parameters**

| **MS data file:** | H:\MBA1-G6\46_BD10_01_1678.mgf |
| --- | --- |
| **Enzyme:** | Trypsin: cuts C-term side of KR unless next residue is P. |
| **Fixed modifications:** | [Carbamidomethyl (C)](http://fun-gen1.ibls.gla.ac.uk/mascot/cgi/client.pl?modification;file=..%2Fdata%2F20110930%2FF112299.dat;mod_name=Carbamidomethyl%20%28C%29) |
| **Variable modifications:** | [Oxidation (M)](http://fun-gen1.ibls.gla.ac.uk/mascot/cgi/client.pl?modification;file=..%2Fdata%2F20110930%2FF112299.dat;mod_name=Oxidation%20%28M%29) |

**Protein sequence coverage: 6%**

Matched peptides shown in ***bold red***.

| **1** | AALQVLSAPN | LLRVGSNENI | FVESQDHVGG | PLNVKIMVKN | HPTQSKELAS |
| --- | --- | --- | --- | --- | --- |
| **51** | KSVVLDQANN | FQAMTQLVIQ | RGPLVDDPKQ | KQYVVLQAQF | PDRLLEKVVL |
| **101** | VSFQSGYIFI | QTDKTIYTPA | STVHYRVFSM | TPGLEPLTRE | IFEDQEVAKN |
| **151** | KEIAVSVEIM | TPENITIFRE | IVNPDKGVKS | GQFKLPDIVS | FGTWHVVTRF |
| **201** | QSTPQKTFSS | EFEVKEYVLP | SFEVSLTPAK | AFFYVDDNDL | TVDITARYLY |
| **251** | GKEVTGTGYV | VFGVITTESE | KKSFPASLQR | VEIKDGKGVA | CLKKEHITQT |
| **301** | FPKIHDLVKQ | SIFVSVSVLT | EGGGEMVEAE | KRGIQIVTSP | YSILFKRTPK |
| **351** | YFKPGMPFDV | SVYITNPDNS | PAIGVEVEVT | PDHAKGVTRA | NGFAKIPLNT |
| **401** | VASATELVIT | VKTKDPGDPR | QQTGGGTMKA | LPYRTSTKNF | LHVGVDSNEL |
| **451** | KIGDPIKIDL | NLGPTTIPNH | DLTYMFLSRG | QLVKVGRFKR | QGNALVTLSV |
| **501** | PVSKELLPSF | RIVAYYHVGA | ADLVADSVWV | DIKVSCMGSL | KVTSTRPKAS |
| **551** | YEPRRAFSLT | ITGDPGAKVG | LVAVDKGVYV | LNSKHRLTQT | KIWDTIEKHD |
| **601** | TGCTAGGGAD | NMGVFYDAGL | VFETNTAKGT | GIRTDPSCPV | SSRRRRAVTI |
| **651** | SDVITSMASK | YHGLAKECCV | DGMRDNTMGY | TCDRRAQYIS | DGDVCVQAFL |
| **701** | VCCTEMASKK | IESKQDALLL | SRSEEDDDDD | AYMRSEDIVS | RSQFPESWMW |
| **751** | EDTNLPECPA | QNKHCESTSV | IRNNFLKDSI | TTWQITAISL | SKTHGICVAD |
| **801** | PFEMIVLKEF | FIDLKLPYSA | VRNEQLEVKA | ILHNYSEDPI | IVR**VELMENG** |
| **851** | **EVCSSASK**KG | KYRQEVNMDP | MSTR**VVPYVI** | **IPMK**LGLHSI | EVKASVKNSG |
| **901** | SNDGVKRDLR | VVAEGVLVKK | ETNVLLNPVK | HGGEQTSHIP | SGVPRNQVPN |
| **951** | SDADTLISVT | AGEQTSVLVE | QAISGDSLGS | LIVQPVGCGE | QNMIYMTLPV |
| **1001** | IATHYLDNTK | KWEDIGLDKR | NTAIKYINIG | YQRQLAYRKE | DGSYAAWVSR |
| **1051** | QSSTWLTAYV | VKVFAMSSTL | ISVQENVLCT | AVKWLILNTQ | QPDGIFNEFA |
| **1101** | PVIHAEMTGN | VRGSDNDASM | TAFVLIAMQE | ASSVCEQSVN | SLPGSMAKAV |
| **1151** | AYLEKRLPHL | TNPYAVAMTS | YALANAGKLN | KETLLKFASP | QLDHWPVPGG |
| **1201** | YQYTLEATSY | ALLALVKVKA | FEEAGPIVRW | LNKQKKVGGG | YGSTQSTIMV |
| **1251** | FQAVAEYWSH | VKDLKDFDLN | INLEVAGRAS | VTKWSINNKN | QFHTRTDKVN |
| **1301** | SIDKDLTVK**A** | **SGNGEATLSV** | **VTLYYALPEE** | **K**DSDCESFDL | SVTLTKMDKT |
| **1351** | SHEDAKESFM | LTIEVLYKNS | ERDATMSILD | IGLLTGFIVD | TDDLNQLSKG |
| **1401** | RERYIEKFEM | DKVLSERGSL | ILYLDKVSHK | LEDRISFKIH | RVQEVGVLQP |
| **1451** | AAVSVYEYYN | QKRCVK**FYHP** | **QR**EGGTLSR**L** | **CLGDVCTCAE** | **ESCSMQK**KGE |
| **1501** | PDVQRIDK**AC** | **GAGLDYVYK**A | TVVDSKLTTH | TDTYTVKIDL | VIKPGTDEGV |
| **1551** | EGKNRDFMGL | AYCREALGLM | QGK**TYMIMGK** | SEDLHRVEDK | GLLQYKYVLG |
| **1601** | EQTWIEYWPS | QQECTSRDYR | **EVCLGIDEFI** | **NQITTFGCPV** |  |

**Protein View: Q70SU8_SALSA**

**Cystein proteinase inhibitor protein precursor.- Salmo salar (Atlantic salmon).**

Top of Form

| **Database:** | MSDB |
| --- | --- |
| **Score:** | 36 |
| **Nominal mass (M_r_):** | 39471 |
| **Calculated pI:** | 5.73 |
| **Taxonomy:** | [Salmo salar](http://www.ncbi.nlm.nih.gov/Taxonomy/Browser/wwwtax.cgi?lvl=0&id=8030) |

This protein sequence matches the following other entries:

- CAD80246 from [Salmo salar](http://www.ncbi.nlm.nih.gov/Taxonomy/Browser/wwwtax.cgi?lvl=0&id=8030)

Sequence similarity is available as [an NCBI BLAST search of Q70SU8_SALSA against nr](http://www.ncbi.nlm.nih.gov/blast/Blast.cgi?ALIGNMENTS=50;ALIGNMENT_VIEW=Pairwise;AUTO_FORMAT=Semiauto;CDD_SEARCH=on;CLIENT=web;COMPOSITION_BASED_STATISTICS=on;DATABASE=nr;DESCRIPTIONS=100;ENTREZ_QUERY=%28none%29;EXPECT=10;FILTER=L;FORMAT_BLOCK_ON_RESPAGE=None;FORMAT_OBJECT=Alignment;FORMAT_TYPE=HTML;GAPCOSTS=11%201;I_THRESH=0.001;LAYOUT=TwoWindows;MATRIX_NAME=BLOSUM62;NCBI_GI=on;PAGE=Proteins;PROGRAM=blastp;QUERY=MKSLVLLLLVAVTVSSVVSKPLPEDSEAEVHKEFETWKVKYGKSYPSTEEEAKRKEMWLATRKKVMEHNTRAGNGLESYTMAVNHLADLTTEEVPKGLLPMPRPEEEEVDKEFEMWKTHNGKTYNSTEEEAKRKEIWLATRARVMEHNKRAENGSESFTMGINYFSDMTFEEIPKARLMVVFPTRDGGEEAEVDKEFETWKVQHGKNYGSTEEEAKRKGIWLATRTRVMEHNKRAETGSESFTMGMNHLSDKTTAEVTGRRLQDGEEAEVHKEFETWKVKYGKTYPSTVEEAKRKEIWLATRKMVMEHNKRAENGLESFTMGVNHFADLTAEEVPRGLFPME;SERVICE=plain;SET_DEFAULTS.x=9;SET_DEFAULTS.y=5;SHOW_OVERVIEW=on;WORD_SIZE=3;END_OF_HTTPGET=Yes).

**Search parameters**

| **MS data file:** | H:\MBA1-G6\46_BD10_01_1678.mgf |
| --- | --- |
| **Enzyme:** | Trypsin: cuts C-term side of KR unless next residue is P. |
| **Fixed modifications:** | [Carbamidomethyl (C)](http://fun-gen1.ibls.gla.ac.uk/mascot/cgi/client.pl?modification;file=..%2Fdata%2F20110930%2FF112299.dat;mod_name=Carbamidomethyl%20%28C%29) |
| **Variable modifications:** | [Oxidation (M)](http://fun-gen1.ibls.gla.ac.uk/mascot/cgi/client.pl?modification;file=..%2Fdata%2F20110930%2FF112299.dat;mod_name=Oxidation%20%28M%29) |

**Protein sequence coverage: 12%**

Matched peptides shown in ***bold red***.

| **1** | MKSLVLLLLV | AVTVSSVVSK | PLPEDSEAEV | HKEFETWKVK | YGKSYPSTEE |
| --- | --- | --- | --- | --- | --- |
| **51** | EAKRKEMWLA | TRKKVMEHNT | R**AGNGLESYT** | **MAVNHLADLT** | **TEEVPK**GLLP |
| **101** | MPRPEEEEVD | KEFEMWKTHN | GKTYNSTEEE | AKRKEIWLAT | RARVMEHNKR |
| **151** | AENGSESFTM | GINYFSDMTF | EEIPKARLMV | VFPTRDGGEE | AEVDKEFETW |
| **201** | KVQHGKNYGS | TEEEAKRKGI | WLATRTRVME | HNK**RAETGSE** | **SFTMGMNHLS** |
| **251** | **DK**TTAEVTGR | RLQDGEEAEV | HKEFETWKVK | YGKTYPSTVE | EAKRKEIWLA |
| **301** | TRKMVMEHNK | RAENGLESFT | MGVNHFADLT | AEEVPRGLFP | ME |

**SPOT # 357**

**Protein View: ABONS2**

**serum albumin 2 precursor - Atlantic salmon**

Top of Form

| **Database:** | MSDB |
| --- | --- |
| **Score:** | 302 |
| **Nominal mass (M_r_):** | 69124 |
| **Calculated pI:** | 5.44 |
| **Taxonomy:** | [Salmo salar](http://www.ncbi.nlm.nih.gov/Taxonomy/Browser/wwwtax.cgi?lvl=0&id=8030) |

This protein sequence matches the following other entries:

- CAA43187 from [Salmo salar](http://www.ncbi.nlm.nih.gov/Taxonomy/Browser/wwwtax.cgi?lvl=0&id=8030)
- ALBU2_SALSA from [Salmo salar](http://www.ncbi.nlm.nih.gov/Taxonomy/Browser/wwwtax.cgi?lvl=0&id=8030)

Sequence similarity is available as [an NCBI BLAST search of ABONS2 against nr](http://www.ncbi.nlm.nih.gov/blast/Blast.cgi?ALIGNMENTS=50;ALIGNMENT_VIEW=Pairwise;AUTO_FORMAT=Semiauto;CDD_SEARCH=on;CLIENT=web;COMPOSITION_BASED_STATISTICS=on;DATABASE=nr;DESCRIPTIONS=100;ENTREZ_QUERY=%28none%29;EXPECT=10;FILTER=L;FORMAT_BLOCK_ON_RESPAGE=None;FORMAT_OBJECT=Alignment;FORMAT_TYPE=HTML;GAPCOSTS=11%201;I_THRESH=0.001;LAYOUT=TwoWindows;MATRIX_NAME=BLOSUM62;NCBI_GI=on;PAGE=Proteins;PROGRAM=blastp;QUERY=MQWLSVCSLLVLLSVLSRSQAQNQICTIFTEAKEDGFKSLILVGLAQNLPDSTLGDLVPLIAEALAMGVKCCSDTPPEDCERDVADLFQSAVCSSETLVEKNDLKMCCEKTAAERTHCFVDHKAKIPRDLSLKAELPAADQCEDFKKDHKAFVGRFIFKFSKSNPMLPPHVVLAIAKGYGEVLTTCCGEAEAQTCFDTKKATFQHAIAKRVAELKSLCIVHKKYGDRVVKAKKLVQYSQKMPQASFQEMAGMVDKIVATVAPCCSGDMVTCMKERKTLVDEVCADESVLSRAAGLSACCKEDAVHRGSCVEAMKPDPKPDGLSEHYDVHADIAAVCQTFTKTPDVAMGKLVYEISVRHPESSQQVILRFAKEAEQALLQCCDMEDHAECVKTALAGSDIDKKITDETDYYKKMCAAEAAVSDDNFEKSMMVYYTRIMPQASFDQLHMVSETVHDVLHACCKDEPGHFVLPCAEEKLTDAIDATCDDYDPSSINPHIAHCCNQSYSMRRHCILAIQPDTEFTPPELDASSFHMGPELCTKDSKDLLLSGKKLLYGVVRHKTTITEDHLKTISTKYHTMKDKCCAAEDQAACFTEEAPKLVSESAELVKV;SERVICE=plain;SET_DEFAULTS.x=9;SET_DEFAULTS.y=5;SHOW_OVERVIEW=on;WORD_SIZE=3;END_OF_HTTPGET=Yes).

**Search parameters**

| **MS data file:** | H:\MBA1-G6\47_BD11_01_1679.mgf |
| --- | --- |
| **Enzyme:** | Trypsin: cuts C-term side of KR unless next residue is P. |
| **Fixed modifications:** | [Carbamidomethyl (C)](http://fun-gen1.ibls.gla.ac.uk/mascot/cgi/client.pl?modification;file=..%2Fdata%2F20110930%2FF112300.dat;mod_name=Carbamidomethyl%20%28C%29) |
| **Variable modifications:** | [Oxidation (M)](http://fun-gen1.ibls.gla.ac.uk/mascot/cgi/client.pl?modification;file=..%2Fdata%2F20110930%2FF112300.dat;mod_name=Oxidation%20%28M%29) |

**Protein sequence coverage: 18%**

Matched peptides shown in ***bold red***.

| **1** | MQWLSVCSLL | VLLSVLSRSQ | AQNQICTIFT | EAKEDGFKSL | ILVGLAQNLP |
| --- | --- | --- | --- | --- | --- |
| **51** | DSTLGDLVPL | IAEALAMGVK | CCSDTPPEDC | ERDVADLFQS | AVCSSETLVE |
| **101** | KNDLKMCCEK | TAAERTHCFV | DHKAKIPRDL | SLKAELPAAD | QCEDFKKDHK |
| **151** | AFVGRFIFKF | SKSNPMLPPH | VVLAIAKGYG | EVLTTCCGEA | EAQTCFDTKK |
| **201** | ATFQHAIAKR | VAELK**SLCIV** | **HK**KYGDRVVK | AKKLVQYSQK | **MPQASFQEMA** |
| **251** | **GMVDK**IVATV | APCCSGDMVT | CMKER**KTLVD** | **EVCADESVLS** | **R**AAGLSACCK |
| **301** | EDAVHRGSCV | EAMKPDPKPD | GLSEHYDVHA | DIAAVCQTFT | KTPDVAMGKL |
| **351** | VYEISVR**HPE** | **SSQQVILR**FA | KEAEQALLQC | CDMEDHAECV | K**TALAGSDID** |
| **401** | **KKITDETDYY** | **KKMCAAEAAV** | **SDDNFEK**SMM | VYYTRIMPQA | SFDQLHMVSE |
| **451** | TVHDVLHACC | KDEPGHFVLP | CAEEKLTDAI | DATCDDYDPS | SINPHIAHCC |
| **501** | NQSYSMRRHC | ILAIQPDTEF | TPPELDASSF | HMGPELCTKD | SK**DLLLSGK**K |
| **551** | **LLYGVVR**HKT | TITEDHLKTI | STKYHTMKDK | CCAAEDQAAC | FTEEAPK**LVS** |
| **601** | **ESAELVKV** |  |  |  |  |

Bottom of Form

**Protein View: Q9DEC8_ONCMY**

**Complement factor B/C2-B.- Oncorhynchus mykiss (Rainbow trout) (Salmo gairdneri).**

Top of Form

| **Database:** | MSDB |
| --- | --- |
| **Score:** | 49 |
| **Nominal mass (M_r_):** | 88834 |
| **Calculated pI:** | 7.52 |
| **Taxonomy:** | [Oncorhynchus mykiss](http://www.ncbi.nlm.nih.gov/Taxonomy/Browser/wwwtax.cgi?lvl=0&id=8022) |

This protein sequence matches the following other entries:

- BAB19788 from [Oncorhynchus mykiss](http://www.ncbi.nlm.nih.gov/Taxonomy/Browser/wwwtax.cgi?lvl=0&id=8022)

Sequence similarity is available as [an NCBI BLAST search of Q9DEC8_ONCMY against nr](http://www.ncbi.nlm.nih.gov/blast/Blast.cgi?ALIGNMENTS=50;ALIGNMENT_VIEW=Pairwise;AUTO_FORMAT=Semiauto;CDD_SEARCH=on;CLIENT=web;COMPOSITION_BASED_STATISTICS=on;DATABASE=nr;DESCRIPTIONS=100;ENTREZ_QUERY=%28none%29;EXPECT=10;FILTER=L;FORMAT_BLOCK_ON_RESPAGE=None;FORMAT_OBJECT=Alignment;FORMAT_TYPE=HTML;GAPCOSTS=11%201;I_THRESH=0.001;LAYOUT=TwoWindows;MATRIX_NAME=BLOSUM62;NCBI_GI=on;PAGE=Proteins;PROGRAM=blastp;QUERY=MHAVTLLLCAAFSFSSVKEVWMQGEDYGDYEDEQPQNCSIAEKIRGGNVSYSQAGTEGSVLTYHCKAGHYPYPVSQRVCSADGEWSSMRLADGRRVSRASCKEIMCPGQLQLDHGEFWPREQWLKPGESQSFSCHSGFSLSGSAQRNCTSLGDWTGTIPVCDSQADDCKNPGVPPGALRSEGRFRKGEKVQYRCQMGLDLLGSAERVCLESREWSGSETRCQTQYAFDSPSAVAEAMGGSLSGVMDVLSPEFIKKNKGVTFGRTLRVADGRMNVFILMDTSGSISKTHFNLARGAIADLIRKLDSYEIELNFQVISYASQAKDIVDIVSSNDEHVRSNVRSVLKRLAAFDHRSHGNKTGTNLYAALYRVYERMAILRERNETRFSETQNVILIETDGYSNTGGSPLAVLAKIRSLLGYSTSALDHTEDKLLDVYVFGVGDNVNKNELNLIASRKRHEKHLFILKDYKQLGEVFNSMISDKSVTMCGVAQEDVSENQEVFGPTKKAYTRPWHVNVITTGVKSETCQGSIVTQNWILTAAHCFSAIRFEGKVDQEKVTVKHGYGGNKAAKVLLVISHPQYNVNGLSHKKVKEFYDYDIALVKVETIKLSWTARPICLPCTKPANRAMKMSPNSTCERHEKALLPMEETRAYFINKGATVKDAKRKQTHIHTGSKRPDCIKQAENTLLTPHDATLNEYVPDRFLCSGGSADHIDAVTCKGDSGGALFLLNRLRYFQVGVVSWGTKNVCEPQDSSDRPPPDARDFHISVFHLLPWLKQHLGTELEFLPMDD;SERVICE=plain;SET_DEFAULTS.x=9;SET_DEFAULTS.y=5;SHOW_OVERVIEW=on;WORD_SIZE=3;END_OF_HTTPGET=Yes).

**Search parameters**

| **MS data file:** | H:\MBA1-G6\47_BD11_01_1679.mgf |
| --- | --- |
| **Enzyme:** | Trypsin: cuts C-term side of KR unless next residue is P. |
| **Fixed modifications:** | [Carbamidomethyl (C)](http://fun-gen1.ibls.gla.ac.uk/mascot/cgi/client.pl?modification;file=..%2Fdata%2F20110930%2FF112300.dat;mod_name=Carbamidomethyl%20%28C%29) |
| **Variable modifications:** | [Oxidation (M)](http://fun-gen1.ibls.gla.ac.uk/mascot/cgi/client.pl?modification;file=..%2Fdata%2F20110930%2FF112300.dat;mod_name=Oxidation%20%28M%29) |

**Protein sequence coverage: 3%**

Matched peptides shown in ***bold red***.

| **1** | MHAVTLLLCA | AFSFSSVKEV | WMQGEDYGDY | EDEQPQNCSI | AEKIRGGNVS |
| --- | --- | --- | --- | --- | --- |
| **51** | YSQAGTEGSV | LTYHCK**AGHY** | **PYPVSQR**VCS | ADGEWSSMRL | ADGRRVSRAS |
| **101** | CKEIMCPGQL | QLDHGEFWPR | EQWLKPGESQ | SFSCHSGFSL | SGSAQRNCTS |
| **151** | LGDWTGTIPV | CDSQADDCKN | PGVPPGALRS | EGRFRKGEKV | QYRCQMGLDL |
| **201** | LGSAERVCLE | SREWSGSETR | CQTQYAFDSP | SAVAEAMGGS | LSGVMDVLSP |
| **251** | EFIKKNKGVT | FGRTLRVADG | R**MNVFILMDT** | **SGSISK**THFN | LARGAIADLI |
| **301** | RKLDSYEIEL | NFQVISYASQ | AKDIVDIVSS | NDEHVRSNVR | SVLKRLAAFD |
| **351** | HRSHGNKTGT | NLYAALYRVY | ERMAILRERN | ETRFSETQNV | ILIETDGYSN |
| **401** | TGGSPLAVLA | KIRSLLGYST | SALDHTEDKL | LDVYVFGVGD | NVNKNELNLI |
| **451** | ASRKRHEKHL | FILKDYKQLG | EVFNSMISDK | SVTMCGVAQE | DVSENQEVFG |
| **501** | PTKKAYTRPW | HVNVITTGVK | SETCQGSIVT | QNWILTAAHC | FSAIRFEGKV |
| **551** | DQEKVTVKHG | YGGNKAAKVL | LVISHPQYNV | NGLSHKKVKE | FYDYDIALVK |
| **601** | VETIKLSWTA | RPICLPCTKP | ANRAMKMSPN | STCERHEKAL | LPMEETRAYF |
| **651** | INKGATVKDA | KRKQTHIHTG | SKRPDCIKQA | ENTLLTPHDA | TLNEYVPDRF |
| **701** | LCSGGSADHI | DAVTCKGDSG | GALFLLNRLR | YFQVGVVSWG | TKNVCEPQDS |
| **751** | SDRPPPDARD | FHISVFHLLP | WLKQHLGTEL | EFLPMDD |  |

**SPOT #529**

**Protein View: Q9DDG6_SALTR**

**Alpha-2 enolase-1 (Fragment).- Salmo trutta (Brown trout).**

Top of Form

| **Database:** | MSDB |
| --- | --- |
| **Score:** | 80 |
| **Nominal mass (M_r_):** | 39667 |
| **Calculated pI:** | 5.78 |
| **Taxonomy:** | [Salmo trutta](http://www.ncbi.nlm.nih.gov/Taxonomy/Browser/wwwtax.cgi?lvl=0&id=8032) |

This protein sequence matches the following other entries:

- AAG16311 from [Salmo trutta](http://www.ncbi.nlm.nih.gov/Taxonomy/Browser/wwwtax.cgi?lvl=0&id=8032)

Sequence similarity is available as [an NCBI BLAST search of Q9DDG6_SALTR against nr](http://www.ncbi.nlm.nih.gov/blast/Blast.cgi?ALIGNMENTS=50;ALIGNMENT_VIEW=Pairwise;AUTO_FORMAT=Semiauto;CDD_SEARCH=on;CLIENT=web;COMPOSITION_BASED_STATISTICS=on;DATABASE=nr;DESCRIPTIONS=100;ENTREZ_QUERY=%28none%29;EXPECT=10;FILTER=L;FORMAT_BLOCK_ON_RESPAGE=None;FORMAT_OBJECT=Alignment;FORMAT_TYPE=HTML;GAPCOSTS=11%201;I_THRESH=0.001;LAYOUT=TwoWindows;MATRIX_NAME=BLOSUM62;NCBI_GI=on;PAGE=Proteins;PROGRAM=blastp;QUERY=TKKGLFRAAVPSGASTGIYEALELRDNDKTRYLGKGVKRAVKHINEFLAPALCNQNVNVLEQEKVDKLMLDMDGTENKSKFGANAILGVSLAVCKAGAAEKGVPLYRHIADLAGNPNXILPCPAFNVINGGSHAGNKLAMQEFMILPIGASNFHEAMRIGAEVYHNLKNVIKAKYGKDATNVGDEGGFAPNILENNEALELLKSAIEKAGYPDKIIIGMDVAASEFYKAGKYDLDFKSPDDPARYITXDQLGDLYKSFIKGYPVQSIEDPFDQDDWAAWSKFTAAVDIQVVGDDLTVTNPKRIQQAVEKKACNCLLLKVNQIGSVTESIKACKLAQSNGWGVMVSHRSGETEDTFIADLVVGL;SERVICE=plain;SET_DEFAULTS.x=9;SET_DEFAULTS.y=5;SHOW_OVERVIEW=on;WORD_SIZE=3;END_OF_HTTPGET=Yes).

**Search parameters**

| **MS data file:** | H:\MBA1-G6\48_BD12_01_1680.mgf |
| --- | --- |
| **Enzyme:** | Trypsin: cuts C-term side of KR unless next residue is P. |
| **Fixed modifications:** | [Carbamidomethyl (C)](http://fun-gen1.ibls.gla.ac.uk/mascot/cgi/client.pl?modification;file=..%2Fdata%2F20110930%2FF112301.dat;mod_name=Carbamidomethyl%20%28C%29) |
| **Variable modifications:** | [Oxidation (M)](http://fun-gen1.ibls.gla.ac.uk/mascot/cgi/client.pl?modification;file=..%2Fdata%2F20110930%2FF112301.dat;mod_name=Oxidation%20%28M%29) |

**Protein sequence coverage: 11%**

Matched peptides shown in ***bold red***.

| **1** | TKKGLFRAAV | PSGASTGIYE | ALELRDNDKT | RYLGKGVKRA | VKHINEFLAP |
| --- | --- | --- | --- | --- | --- |
| **51** | ALCNQNVNVL | EQEKVDKLML | DMDGTENKSK | FGANAILGVS | LAVCKAGAAE |
| **101** | KGVPLYRHIA | DLAGNPNXIL | PCPAFNVING | GSHAGNK**LAM** | **QEFMILPIGA** |
| **151** | **SNFHEAMRIG** | **AEVYHNLK**NV | IKAKYGKDAT | NVGDEGGFAP | NILENNEALE |
| **201** | LLKSAIEKAG | YPDKIIIGMD | VAASEFYKAG | KYDLDFKSPD | DPAR**YITXDQ** |
| **251** | **LGDLYK**SFIK | GYPVQSIEDP | FDQDDWAAWS | KFTAAVDIQV | VGDDLTVTNP |
| **301** | KRIQQAVEKK | ACNCLLLKVN | QIGSVTESIK | ACKLAQSNGW | GVMVSHRSGE |
| **351** | TEDTFIADLV | VGL |  |  |  |

**SPOT #368**

**Protein View: ABONS2**

**serum albumin 2 precursor - Atlantic salmon**

Top of Form

| **Database:** | MSDB |
| --- | --- |
| **Score:** | 318 |
| **Nominal mass (M_r_):** | 69124 |
| **Calculated pI:** | 5.44 |
| **Taxonomy:** | [Salmo salar](http://www.ncbi.nlm.nih.gov/Taxonomy/Browser/wwwtax.cgi?lvl=0&id=8030) |

This protein sequence matches the following other entries:

- CAA43187 from [Salmo salar](http://www.ncbi.nlm.nih.gov/Taxonomy/Browser/wwwtax.cgi?lvl=0&id=8030)
- ALBU2_SALSA from [Salmo salar](http://www.ncbi.nlm.nih.gov/Taxonomy/Browser/wwwtax.cgi?lvl=0&id=8030)

Sequence similarity is available as [an NCBI BLAST search of ABONS2 against nr](http://www.ncbi.nlm.nih.gov/blast/Blast.cgi?ALIGNMENTS=50;ALIGNMENT_VIEW=Pairwise;AUTO_FORMAT=Semiauto;CDD_SEARCH=on;CLIENT=web;COMPOSITION_BASED_STATISTICS=on;DATABASE=nr;DESCRIPTIONS=100;ENTREZ_QUERY=%28none%29;EXPECT=10;FILTER=L;FORMAT_BLOCK_ON_RESPAGE=None;FORMAT_OBJECT=Alignment;FORMAT_TYPE=HTML;GAPCOSTS=11%201;I_THRESH=0.001;LAYOUT=TwoWindows;MATRIX_NAME=BLOSUM62;NCBI_GI=on;PAGE=Proteins;PROGRAM=blastp;QUERY=MQWLSVCSLLVLLSVLSRSQAQNQICTIFTEAKEDGFKSLILVGLAQNLPDSTLGDLVPLIAEALAMGVKCCSDTPPEDCERDVADLFQSAVCSSETLVEKNDLKMCCEKTAAERTHCFVDHKAKIPRDLSLKAELPAADQCEDFKKDHKAFVGRFIFKFSKSNPMLPPHVVLAIAKGYGEVLTTCCGEAEAQTCFDTKKATFQHAIAKRVAELKSLCIVHKKYGDRVVKAKKLVQYSQKMPQASFQEMAGMVDKIVATVAPCCSGDMVTCMKERKTLVDEVCADESVLSRAAGLSACCKEDAVHRGSCVEAMKPDPKPDGLSEHYDVHADIAAVCQTFTKTPDVAMGKLVYEISVRHPESSQQVILRFAKEAEQALLQCCDMEDHAECVKTALAGSDIDKKITDETDYYKKMCAAEAAVSDDNFEKSMMVYYTRIMPQASFDQLHMVSETVHDVLHACCKDEPGHFVLPCAEEKLTDAIDATCDDYDPSSINPHIAHCCNQSYSMRRHCILAIQPDTEFTPPELDASSFHMGPELCTKDSKDLLLSGKKLLYGVVRHKTTITEDHLKTISTKYHTMKDKCCAAEDQAACFTEEAPKLVSESAELVKV;SERVICE=plain;SET_DEFAULTS.x=9;SET_DEFAULTS.y=5;SHOW_OVERVIEW=on;WORD_SIZE=3;END_OF_HTTPGET=Yes).

**Search parameters**

| **MS data file:** | H:\MBA1-G6\49_BE1_01_1681.mgf |
| --- | --- |
| **Enzyme:** | Trypsin: cuts C-term side of KR unless next residue is P. |
| **Fixed modifications:** | [Carbamidomethyl (C)](http://fun-gen1.ibls.gla.ac.uk/mascot/cgi/client.pl?modification;file=..%2Fdata%2F20110930%2FF112302.dat;mod_name=Carbamidomethyl%20%28C%29) |
| **Variable modifications:** | [Oxidation (M)](http://fun-gen1.ibls.gla.ac.uk/mascot/cgi/client.pl?modification;file=..%2Fdata%2F20110930%2FF112302.dat;mod_name=Oxidation%20%28M%29) |

**Protein sequence coverage: 19%**

Matched peptides shown in ***bold red***.

| **1** | MQWLSVCSLL | VLLSVLSRSQ | AQNQICTIFT | EAKEDGFKSL | ILVGLAQNLP |
| --- | --- | --- | --- | --- | --- |
| **51** | DSTLGDLVPL | IAEALAMGVK | CCSDTPPEDC | ERDVADLFQS | AVCSSETLVE |
| **101** | KNDLKMCCEK | TAAERTHCFV | DHKAKIPRDL | SLKAELPAAD | QCEDFKKDHK |
| **151** | AFVGRFIFKF | SKSNPMLPPH | VVLAIAKGYG | EVLTTCCGEA | EAQTCFDTKK |
| **201** | ATFQHAIAKR | VAELKSLCIV | HKKYGDRVVK | AKKLVQYSQK | **MPQASFQEMA** |
| **251** | **GMVDKIVATV** | **APCCSGDMVT** | **CMK**ER**KTLVD** | **EVCADESVLS** | **R**AAGLSACCK |
| **301** | EDAVHRGSCV | EAMKPDPKPD | GLSEHYDVHA | DIAAVCQTFT | KTPDVAMGKL |
| **351** | VYEISVR**HPE** | **SSQQVILR**FA | KEAEQALLQC | CDMEDHAECV | K**TALAGSDID** |
| **401** | **KKITDETDYY** | **KKMCAAEAAV** | **SDDNFEK**SMM | VYYTRIMPQA | SFDQLHMVSE |
| **451** | TVHDVLHACC | K**DEPGHFVLP** | **CAEEK**LTDAI | DATCDDYDPS | SINPHIAHCC |
| **501** | NQSYSMRRHC | ILAIQPDTEF | TPPELDASSF | HMGPELCTKD | SKDLLLSGKK |
| **551** | **LLYGVVR**HKT | TITEDHLKTI | STKYHTMKDK | CCAAEDQAAC | FTEEAPKLVS |
| **601** | ESAELVKV |  |  |  |  |

Bottom of Form

**Protein View: ABONS1**

**serum albumin 1 precursor - Atlantic salmon**

Top of Form

| **Database:** | MSDB |
| --- | --- |
| **Score:** | 295 |
| **Nominal mass (M_r_):** | 69216 |
| **Calculated pI:** | 5.44 |
| **Taxonomy:** | [Salmo salar](http://www.ncbi.nlm.nih.gov/Taxonomy/Browser/wwwtax.cgi?lvl=0&id=8030) |

This protein sequence matches the following other entries:

- CAA36643 from [Salmo salar](http://www.ncbi.nlm.nih.gov/Taxonomy/Browser/wwwtax.cgi?lvl=0&id=8030)
- ALBU1_SALSA from [Salmo salar](http://www.ncbi.nlm.nih.gov/Taxonomy/Browser/wwwtax.cgi?lvl=0&id=8030)

Sequence similarity is available as [an NCBI BLAST search of ABONS1 against nr](http://www.ncbi.nlm.nih.gov/blast/Blast.cgi?ALIGNMENTS=50;ALIGNMENT_VIEW=Pairwise;AUTO_FORMAT=Semiauto;CDD_SEARCH=on;CLIENT=web;COMPOSITION_BASED_STATISTICS=on;DATABASE=nr;DESCRIPTIONS=100;ENTREZ_QUERY=%28none%29;EXPECT=10;FILTER=L;FORMAT_BLOCK_ON_RESPAGE=None;FORMAT_OBJECT=Alignment;FORMAT_TYPE=HTML;GAPCOSTS=11%201;I_THRESH=0.001;LAYOUT=TwoWindows;MATRIX_NAME=BLOSUM62;NCBI_GI=on;PAGE=Proteins;PROGRAM=blastp;QUERY=MQWLSVCSLLVLLSVLSRSQAQNQICTIFTEAKEDGFKSLILVGLAQNLPDSTLGDLVPLIAEALAMGVKCCSDTPPEDCERDVADLFQSAVCSSETLVEKNDLKMCCEKTAAERTHCFVDHKAKIPRDLSLKAELPAADQCEDFKKDHKAFVGRFIFKFSKSNPMLPPHVVLAIAKGYGEVLTTCCGEAEAQTCFDTKKATFQHAVMKRVAELRSLCIVHKKYGDRVVKAKKLVQYSQKMPQASFQEMGGMVDKIVATVAPCCSGDMVTCMKERKTLVDEVCADESVLSRAAGLSACCKEDAVHRGSCVEAMKPDPKPDGLSEHYDIHADIAAVCQTFTKTPDVAMGKLVYEISVRHPESSQQVILRFAKEAEQALLQCCDMEDHAECVKTALAGSDIDKKITDETDYYKKMCAAEAAVSDDSFEKSMMVYYTRIMPQASFDQLHMVSETVHDVLHACCKDEQGHFVLPCAEEKLTDAIDATCDDYDPSSINPHIAHCCNQSYSMRRHCILAIQPDTEFTPPELDASSFHMGPELCTKDSKDLLLSGKKLLYGVVRHKTTITEDHLKTISTKYHTMKEKCCAAEDQAACFTEEAPKLVSESAELVKV;SERVICE=plain;SET_DEFAULTS.x=9;SET_DEFAULTS.y=5;SHOW_OVERVIEW=on;WORD_SIZE=3;END_OF_HTTPGET=Yes).

**Search parameters**

| **MS data file:** | H:\MBA1-G6\49_BE1_01_1681.mgf |
| --- | --- |
| **Enzyme:** | Trypsin: cuts C-term side of KR unless next residue is P. |
| **Fixed modifications:** | [Carbamidomethyl (C)](http://fun-gen1.ibls.gla.ac.uk/mascot/cgi/client.pl?modification;file=..%2Fdata%2F20110930%2FF112302.dat;mod_name=Carbamidomethyl%20%28C%29) |
| **Variable modifications:** | [Oxidation (M)](http://fun-gen1.ibls.gla.ac.uk/mascot/cgi/client.pl?modification;file=..%2Fdata%2F20110930%2FF112302.dat;mod_name=Oxidation%20%28M%29) |

**Protein sequence coverage: 16%**

Matched peptides shown in ***bold red***.

| **1** | MQWLSVCSLL | VLLSVLSRSQ | AQNQICTIFT | EAKEDGFKSL | ILVGLAQNLP |
| --- | --- | --- | --- | --- | --- |
| **51** | DSTLGDLVPL | IAEALAMGVK | CCSDTPPEDC | ERDVADLFQS | AVCSSETLVE |
| **101** | KNDLKMCCEK | TAAERTHCFV | DHKAKIPRDL | SLKAELPAAD | QCEDFKKDHK |
| **151** | AFVGRFIFKF | SKSNPMLPPH | VVLAIAKGYG | EVLTTCCGEA | EAQTCFDTKK |
| **201** | ATFQHAVMKR | VAELRSLCIV | HKKYGDRVVK | AKKLVQYSQK | **MPQASFQEMG** |
| **251** | **GMVDKIVATV** | **APCCSGDMVT** | **CMK**ER**KTLVD** | **EVCADESVLS** | **R**AAGLSACCK |
| **301** | EDAVHRGSCV | EAMKPDPKPD | GLSEHYDIHA | DIAAVCQTFT | KTPDVAMGKL |
| **351** | VYEISVR**HPE** | **SSQQVILR**FA | KEAEQALLQC | CDMEDHAECV | K**TALAGSDID** |
| **401** | **KKITDETDYY** | **KK**MCAAEAAV | SDDSFEKSMM | VYYTRIMPQA | SFDQLHMVSE |
| **451** | TVHDVLHACC | K**DEQGHFVLP** | **CAEEK**LTDAI | DATCDDYDPS | SINPHIAHCC |
| **501** | NQSYSMRRHC | ILAIQPDTEF | TPPELDASSF | HMGPELCTKD | SKDLLLSGKK |
| **551** | **LLYGVVR**HKT | TITEDHLKTI | STKYHTMKEK | CCAAEDQAAC | FTEEAPKLVS |
| **601** | ESAELVKV |  |  |  |  |

**SPOT #450**

**Protein View: TRF2_SALSA**

**Serotransferrin II precursor (Siderophilin II) (STF II).- Salmo salar (Atlantic salmon).**

Top of Form

| **Database:** | MSDB |
| --- | --- |
| **Score:** | 444 |
| **Nominal mass (M_r_):** | 76497 |
| **Calculated pI:** | 7.08 |
| **Taxonomy:** | [Salmo salar](http://www.ncbi.nlm.nih.gov/Taxonomy/Browser/wwwtax.cgi?lvl=0&id=8030) |

Sequence similarity is available as [an NCBI BLAST search of TRF2_SALSA against nr](http://www.ncbi.nlm.nih.gov/blast/Blast.cgi?ALIGNMENTS=50;ALIGNMENT_VIEW=Pairwise;AUTO_FORMAT=Semiauto;CDD_SEARCH=on;CLIENT=web;COMPOSITION_BASED_STATISTICS=on;DATABASE=nr;DESCRIPTIONS=100;ENTREZ_QUERY=%28none%29;EXPECT=10;FILTER=L;FORMAT_BLOCK_ON_RESPAGE=None;FORMAT_OBJECT=Alignment;FORMAT_TYPE=HTML;GAPCOSTS=11%201;I_THRESH=0.001;LAYOUT=TwoWindows;MATRIX_NAME=BLOSUM62;NCBI_GI=on;PAGE=Proteins;PROGRAM=blastp;QUERY=MKLLLLSALLGCLATAYAAPAEGIVKWCVKSEQELRKCHDLAAKVAEFSCVRKDGSFECIQAIKGGEADAITLDGGDIYTAGLTNYGLQPIIAEDYGEDSDTCYYAVAVAKKGTAFGFKTLRGKKSCHTGLGKSAGWNIPIGTLVTESQIRWAGIEDRPVESAVSDFFNASCAPGATMGSKLCQLCKGDCSRSHKEPYYDYAGAFQCLKDGAGDVAFIKPLAVPAAEKASYELLCKDGTRASIDSYKTCHLARVPAHAVVSRKDPELANRIYNKLVAVKDFNLFSSDGYAAKNLMFKDSAQKLVQLPTTTDSFLYLGAEYMSTIRSLKKSQATGASSRAIKWCAVGHAEKGKCDTWTINSFADGESKISCQDAPTVEECIKKIMRKEADAIAVDGGEVYTAGKCGLVPVMVEQYDADLCSAPGEASSYYAVAVAKKGSGLTWKTLKGKRSCHTGLGRTAGWNIPMGLIHQETNDCDFTKYFSKGCAPGSEVGSPFCAQCKGSGKARGGDEDRCKARSEEQYYGYTGAFRCLVEDAGDVAFIKHTIVPESTDGNGPDWAKDLKSSDFELLCQDGTTQPVTKFSECHLAKVPAHAVITRPETRGDVVSILLELQAKFGSSGSDSSFRMFQSSVEKNLLFKDSTKCLQEIPKGTKYQDFLGKEYMIAMQSLRKCSDSTSDLEKACTFHSCQQKE;SERVICE=plain;SET_DEFAULTS.x=9;SET_DEFAULTS.y=5;SHOW_OVERVIEW=on;WORD_SIZE=3;END_OF_HTTPGET=Yes).

**Search parameters**

| **MS data file:** | H:\MBA1-G6\37_BD1_01_1669.mgf |
| --- | --- |
| **Enzyme:** | Trypsin: cuts C-term side of KR unless next residue is P. |
| **Fixed modifications:** | [Carbamidomethyl (C)](http://fun-gen1.ibls.gla.ac.uk/mascot/cgi/client.pl?modification;file=..%2Fdata%2F20110930%2FF112290.dat;mod_name=Carbamidomethyl%20%28C%29) |
| **Variable modifications:** | [Oxidation (M)](http://fun-gen1.ibls.gla.ac.uk/mascot/cgi/client.pl?modification;file=..%2Fdata%2F20110930%2FF112290.dat;mod_name=Oxidation%20%28M%29) |

**Protein sequence coverage: 23%**

Matched peptides shown in ***bold red***.

| **1** | MKLLLLSALL | GCLATAYAAP | AEGIVKWCVK | SEQELRKCHD | LAAKVAEFSC |
| --- | --- | --- | --- | --- | --- |
| **51** | VRK**DGSFECI** | **QAIK**GGEADA | ITLDGGDIYT | AGLTNYGLQP | IIAEDYGEDS |
| **101** | DTCYYAVAVA | KKGTAFGFKT | LRGKKSCHTG | LGKSAGWNIP | IGTLVTESQI |
| **151** | RWAGIEDRPV | ESAVSDFFNA | SCAPGATMGS | KLCQLCKGDC | SR**SHKEPYYD** |
| **201** | **YAGAFQCLKD** | **GAGDVAFIKP** | **LAVPAAEK**AS | YELLCKDGTR | ASIDSYKTCH |
| **251** | LARVPAHAVV | SRKDPELANR | IYNKLVAVK**D** | **FNLFSSDGYA** | **AK**NLMFKDSA |
| **301** | QKLVQLPTTT | DSFLYLGAEY | MSTIRSLKKS | QATGASSRAI | KWCAVGHAEK |
| **351** | GKCDTWTINS | FADGESKISC | QDAPTVEECI | KKIMRK**EADA** | **IAVDGGEVYT** |
| **401** | **AGK**CGLVPVM | VEQYDADLCS | APGEASSYYA | VAVAKKGSGL | TWKTLKGKRS |
| **451** | CHTGLGRTAG | WNIPMGLIHQ | ETNDCDFTKY | FSKGCAPGSE | VGSPFCAQCK |
| **501** | GSGKARGGDE | DRCKAR**SEEQ** | **YYGYTGAFRC** | **LVEDAGDVAF** | **IK**HTIVPEST |
| **551** | DGNGPDWAKD | LK**SSDFELLC** | **QDGTTQPVTK** | FSECHLAK**VP** | **AHAVITRPET** |
| **601** | **RGDVVSILLE** | **LQAKFGSSGS** | **DSSFR**MFQSS | VEKNLLFKDS | TK**CLQEIPK**G |
| **651** | TKYQDFLGKE | YMIAMQSLRK | CSDSTSDLEK | ACTFHSCQQK | E |

**SPOT #395**

**Protein View: TRF2_SALSA**

**Serotransferrin II precursor (Siderophilin II) (STF II).- Salmo salar (Atlantic salmon).**

Top of Form

| **Database:** | MSDB |
| --- | --- |
| **Score:** | 210 |
| **Nominal mass (M_r_):** | 76497 |
| **Calculated pI:** | 7.08 |
| **Taxonomy:** | [Salmo salar](http://www.ncbi.nlm.nih.gov/Taxonomy/Browser/wwwtax.cgi?lvl=0&id=8030) |

Sequence similarity is available as [an NCBI BLAST search of TRF2_SALSA against nr](http://www.ncbi.nlm.nih.gov/blast/Blast.cgi?ALIGNMENTS=50;ALIGNMENT_VIEW=Pairwise;AUTO_FORMAT=Semiauto;CDD_SEARCH=on;CLIENT=web;COMPOSITION_BASED_STATISTICS=on;DATABASE=nr;DESCRIPTIONS=100;ENTREZ_QUERY=%28none%29;EXPECT=10;FILTER=L;FORMAT_BLOCK_ON_RESPAGE=None;FORMAT_OBJECT=Alignment;FORMAT_TYPE=HTML;GAPCOSTS=11%201;I_THRESH=0.001;LAYOUT=TwoWindows;MATRIX_NAME=BLOSUM62;NCBI_GI=on;PAGE=Proteins;PROGRAM=blastp;QUERY=MKLLLLSALLGCLATAYAAPAEGIVKWCVKSEQELRKCHDLAAKVAEFSCVRKDGSFECIQAIKGGEADAITLDGGDIYTAGLTNYGLQPIIAEDYGEDSDTCYYAVAVAKKGTAFGFKTLRGKKSCHTGLGKSAGWNIPIGTLVTESQIRWAGIEDRPVESAVSDFFNASCAPGATMGSKLCQLCKGDCSRSHKEPYYDYAGAFQCLKDGAGDVAFIKPLAVPAAEKASYELLCKDGTRASIDSYKTCHLARVPAHAVVSRKDPELANRIYNKLVAVKDFNLFSSDGYAAKNLMFKDSAQKLVQLPTTTDSFLYLGAEYMSTIRSLKKSQATGASSRAIKWCAVGHAEKGKCDTWTINSFADGESKISCQDAPTVEECIKKIMRKEADAIAVDGGEVYTAGKCGLVPVMVEQYDADLCSAPGEASSYYAVAVAKKGSGLTWKTLKGKRSCHTGLGRTAGWNIPMGLIHQETNDCDFTKYFSKGCAPGSEVGSPFCAQCKGSGKARGGDEDRCKARSEEQYYGYTGAFRCLVEDAGDVAFIKHTIVPESTDGNGPDWAKDLKSSDFELLCQDGTTQPVTKFSECHLAKVPAHAVITRPETRGDVVSILLELQAKFGSSGSDSSFRMFQSSVEKNLLFKDSTKCLQEIPKGTKYQDFLGKEYMIAMQSLRKCSDSTSDLEKACTFHSCQQKE;SERVICE=plain;SET_DEFAULTS.x=9;SET_DEFAULTS.y=5;SHOW_OVERVIEW=on;WORD_SIZE=3;END_OF_HTTPGET=Yes).

**Search parameters**

| **MS data file:** | H:\MBA1-G6\50_BE2_01_1682.mgf |
| --- | --- |
| **Enzyme:** | Trypsin: cuts C-term side of KR unless next residue is P. |
| **Fixed modifications:** | [Carbamidomethyl (C)](http://fun-gen1.ibls.gla.ac.uk/mascot/cgi/client.pl?modification;file=..%2Fdata%2F20110930%2FF112303.dat;mod_name=Carbamidomethyl%20%28C%29) |
| **Variable modifications:** | [Oxidation (M)](http://fun-gen1.ibls.gla.ac.uk/mascot/cgi/client.pl?modification;file=..%2Fdata%2F20110930%2FF112303.dat;mod_name=Oxidation%20%28M%29) |

**Protein sequence coverage: 9%**

Matched peptides shown in ***bold red***.

| **1** | MKLLLLSALL | GCLATAYAAP | AEGIVKWCVK | SEQELRKCHD | LAAKVAEFSC |
| --- | --- | --- | --- | --- | --- |
| **51** | VRKDGSFECI | QAIKGGEADA | ITLDGGDIYT | AGLTNYGLQP | IIAEDYGEDS |
| **101** | DTCYYAVAVA | KKGTAFGFKT | LRGKKSCHTG | LGKSAGWNIP | IGTLVTESQI |
| **151** | RWAGIEDRPV | ESAVSDFFNA | SCAPGATMGS | KLCQLCKGDC | SRSHKEPYYD |
| **201** | YAGAFQCLKD | GAGDVAFIKP | LAVPAAEKAS | YELLCKDGTR | ASIDSYKTCH |
| **251** | LARVPAHAVV | SRKDPELANR | IYNKLVAVKD | FNLFSSDGYA | AKNLMFKDSA |
| **301** | QKLVQLPTTT | DSFLYLGAEY | MSTIRSLKKS | QATGASSRAI | KWCAVGHAEK |
| **351** | GKCDTWTINS | FADGESKISC | QDAPTVEECI | KKIMRK**EADA** | **IAVDGGEVYT** |
| **401** | **AGK**CGLVPVM | VEQYDADLCS | APGEASSYYA | VAVAKKGSGL | TWKTLKGKRS |
| **451** | CHTGLGRTAG | WNIPMGLIHQ | ETNDCDFTKY | FSKGCAPGSE | VGSPFCAQCK |
| **501** | GSGKARGGDE | DRCKAR**SEEQ** | **YYGYTGAFRC** | **LVEDAGDVAF** | **IK**HTIVPEST |
| **551** | DGNGPDWAKD | LKSSDFELLC | QDGTTQPVTK | FSECHLAK**VP** | **AHAVITRPET** |
| **601** | **R**GDVVSILLE | LQAKFGSSGS | DSSFRMFQSS | VEKNLLFKDS | TKCLQEIPKG |
| **651** | TKYQDFLGK**E** | **YMIAMQSLR**K | CSDSTSDLEK | ACTFHSCQQK | E |

**SPOT #146**

**Protein View: Q4QZ25_ONCMY**

**Complement component C9.- Oncorhynchus mykiss (Rainbow trout) (Salmo gairdneri).**

Top of Form

| **Database:** | MSDB |
| --- | --- |
| **Score:** | 226 |
| **Nominal mass (M_r_):** | 68118 |
| **Calculated pI:** | 5.46 |
| **Taxonomy:** | [Oncorhynchus mykiss](http://www.ncbi.nlm.nih.gov/Taxonomy/Browser/wwwtax.cgi?lvl=0&id=8022) |

This protein sequence matches the following other entries:

- CAJ01692 from [Oncorhynchus mykiss](http://www.ncbi.nlm.nih.gov/Taxonomy/Browser/wwwtax.cgi?lvl=0&id=8022)

Sequence similarity is available as [an NCBI BLAST search of Q4QZ25_ONCMY against nr](http://www.ncbi.nlm.nih.gov/blast/Blast.cgi?ALIGNMENTS=50;ALIGNMENT_VIEW=Pairwise;AUTO_FORMAT=Semiauto;CDD_SEARCH=on;CLIENT=web;COMPOSITION_BASED_STATISTICS=on;DATABASE=nr;DESCRIPTIONS=100;ENTREZ_QUERY=%28none%29;EXPECT=10;FILTER=L;FORMAT_BLOCK_ON_RESPAGE=None;FORMAT_OBJECT=Alignment;FORMAT_TYPE=HTML;GAPCOSTS=11%201;I_THRESH=0.001;LAYOUT=TwoWindows;MATRIX_NAME=BLOSUM62;NCBI_GI=on;PAGE=Proteins;PROGRAM=blastp;QUERY=MAEVAAALLLGFCAVAVSVSVSGDENRTGKRDTREVKVAPPDPVDCVWSRWSEWTPCNSCTKIRHRSRSVEVFGQFGGKPCQGQPIGEQQACTSDAVCEQALPSECSSTEFTCESGACIKLRLSCNGDYDCEDGSDEDCEPVRKPCGTKLYDTNEQGRTAGYGINILGMEPRINPFNNDYFNGMCNKVKNINNNEYNRLPWNVGLLNYETIAEETVSKEIYEDTYTLLRELMTETKLTVSAGLNLKFTPTEKSMAKSNTTVSGGVGLDAEYDRTQMIKEVSEYTTIKNKSFMRVNGRLQLSTYRMRSRDLQVAGEFLEHVKSLPLEYEKGQYFSFLEDYGTHYTRNGKSGGEYQLVYVLNQDTIKDKKLTERKLQDCIKVGISANFDTNIGIGGDAHIRPGHCKDTVNKNTAEKEGKALVDKVITLVRGGTLEAAVAMRTQITKEGLMDVTTYQNWARTVGDAPALLSSEPEPIQTLIPLSMPDANTRRLNMQRATQEYEAEYSVCKCKPCHNGGSLALLDGKCLCLCLPQFEGLACQDAKADNNKNTKTPVESVPQEGNWSCWAAWSGCSGGKRIRTRSCNTQGLSDATCRGDIVTEDYC;SERVICE=plain;SET_DEFAULTS.x=9;SET_DEFAULTS.y=5;SHOW_OVERVIEW=on;WORD_SIZE=3;END_OF_HTTPGET=Yes).

**Search parameters**

| **MS data file:** | H:\MBA1-G6\51_BE3_01_1683.mgf |
| --- | --- |
| **Enzyme:** | Trypsin: cuts C-term side of KR unless next residue is P. |
| **Fixed modifications:** | [Carbamidomethyl (C)](http://fun-gen1.ibls.gla.ac.uk/mascot/cgi/client.pl?modification;file=..%2Fdata%2F20110930%2FF112304.dat;mod_name=Carbamidomethyl%20%28C%29) |
| **Variable modifications:** | [Oxidation (M)](http://fun-gen1.ibls.gla.ac.uk/mascot/cgi/client.pl?modification;file=..%2Fdata%2F20110930%2FF112304.dat;mod_name=Oxidation%20%28M%29) |

**Protein sequence coverage: 17%**

Matched peptides shown in ***bold red***.

| **1** | MAEVAAALLL | GFCAVAVSVS | VSGDENRTGK | RDTREVKVAP | PDPVDCVWSR |
| --- | --- | --- | --- | --- | --- |
| **51** | WSEWTPCNSC | TKIRHRSRSV | EVFGQFGGKP | CQGQPIGEQQ | ACTSDAVCEQ |
| **101** | ALPSECSSTE | FTCESGACIK | LRLSCNGDYD | CEDGSDEDCE | PVRKPCGTKL |
| **151** | YDTNEQGR**TA** | **GYGINILGME** | **PR**INPFNNDY | FNGMCNKVKN | INNNEYNRLP |
| **201** | WNVGLLNYET | IAEETVSK**EI** | **YEDTYTLLR**E | LMTETKLTVS | AGLNLKFTPT |
| **251** | EKSMAKSNTT | VSGGVGLDAE | YDRTQMIKEV | SEYTTIKNKS | FMRVNGRLQL |
| **301** | STYRMRSR**DL** | **QVAGEFLEHV** | **K**SLPLEYEKG | QYFSFLEDYG | THYTRNGKSG |
| **351** | GEYQLVYVLN | QDTIKDKKLT | ER**KLQDCIK**V | GISANFDTNI | GIGGDAHIRP |
| **401** | GHCKDTVNKN | TAEKEGKALV | DKVITLVRGG | TLEAAVAMRT | QITKEGLMDV |
| **451** | TTYQNWAR**TV** | **GDAPALLSSE** | **PEPIQTLIPL** | **SMPDANTR**RL | NMQR**ATQEYE** |
| **501** | **AEYSVCK**CKP | CHNGGSLALL | DGK**CLCLCLP** | **QFEGLACQDA** | **K**ADNNKNTKT |
| **551** | PVESVPQEGN | WSCWAAWSGC | SGGKRIRTRS | CNTQGLSDAT | CRGDIVTEDY |
| **601** | C |  |  |  |  |

Bottom of Form

**SPOT #575**

**Protein View: JH0472**

**apolipoprotein A-I precursor - Atlantic salmon**

Top of Form

| **Database:** | MSDB |
| --- | --- |
| **Score:** | 188 |
| **Nominal mass (M_r_):** | 29557 |
| **Calculated pI:** | 8.48 |
| **Taxonomy:** | [Salmo salar](http://www.ncbi.nlm.nih.gov/Taxonomy/Browser/wwwtax.cgi?lvl=0&id=8030) |

Sequence similarity is available as [an NCBI BLAST search of JH0472 against nr](http://www.ncbi.nlm.nih.gov/blast/Blast.cgi?ALIGNMENTS=50;ALIGNMENT_VIEW=Pairwise;AUTO_FORMAT=Semiauto;CDD_SEARCH=on;CLIENT=web;COMPOSITION_BASED_STATISTICS=on;DATABASE=nr;DESCRIPTIONS=100;ENTREZ_QUERY=%28none%29;EXPECT=10;FILTER=L;FORMAT_BLOCK_ON_RESPAGE=None;FORMAT_OBJECT=Alignment;FORMAT_TYPE=HTML;GAPCOSTS=11%201;I_THRESH=0.001;LAYOUT=TwoWindows;MATRIX_NAME=BLOSUM62;NCBI_GI=on;PAGE=Proteins;PROGRAM=blastp;QUERY=MKFLVLALTILLAAGTQAFPMQADAPSQLEHVKAALNMYIAQVKLTAQRSIDLLDDTEYKEYKMQLSQSLDNLQQFADSTSKSWPPTPRSSAPSCDATATVRAEVMKDVEDVRTQLEPKRAELREVLNKHIDEYRKKLEPLIKEHIELRRTEMDAFRAKIEPVVEEMRAKVAVNVEETKTKLMPIVEIVRAKLTERLEELRTLAAPYAEEYKEQMFKAVGEVREKVAPLSEDFKARWAPPPRRPSKSSWLSTRPSARP;SERVICE=plain;SET_DEFAULTS.x=9;SET_DEFAULTS.y=5;SHOW_OVERVIEW=on;WORD_SIZE=3;END_OF_HTTPGET=Yes).

**Search parameters**

| **MS data file:** | H:\MBA1-G6\52_BE4_01_1684.mgf |
| --- | --- |
| **Enzyme:** | Trypsin: cuts C-term side of KR unless next residue is P. |
| **Fixed modifications:** | [Carbamidomethyl (C)](http://fun-gen1.ibls.gla.ac.uk/mascot/cgi/client.pl?modification;file=..%2Fdata%2F20110930%2FF112305.dat;mod_name=Carbamidomethyl%20%28C%29) |
| **Variable modifications:** | [Oxidation (M)](http://fun-gen1.ibls.gla.ac.uk/mascot/cgi/client.pl?modification;file=..%2Fdata%2F20110930%2FF112305.dat;mod_name=Oxidation%20%28M%29) |

**Protein sequence coverage: 28%**

Matched peptides shown in ***bold red***.

| **1** | MKFLVLALTI | LLAAGTQAFP | MQADAPSQLE | HVK**AALNMYI** | **AQVK**LTAQR**S** |
| --- | --- | --- | --- | --- | --- |
| **51** | **IDLLDDTEYK** | **EYKMQLSQSL** | **DNLQQFADST** | **SK**SWPPTPRS | SAPSCDATAT |
| **101** | VRAEVMKDVE | DVRTQLEPKR | AELREVLNKH | IDEYRK**KLEP** | **LIK**EHIELRR |
| **151** | TEMDAFR**AKI** | **EPVVEEMR**AK | VAVNVEETKT | KLMPIVEIVR | AKLTERLEEL |
| **201** | R**TLAAPYAEE** | **YK**EQMFKAVG | EVREKVAPLS | EDFKARWAPP | PRRPSKSSWL |
| **251** | STRPSARP |  |  |  |  |

Bottom of Form

**Protein View: AAG18369**

**Immunoglobulin light chain precursor.- Salmo salar (Atlantic salmon).**

Top of Form

| **Database:** | MSDB |
| --- | --- |
| **Score:** | 76 |
| **Nominal mass (M_r_):** | 26519 |
| **Calculated pI:** | 6.29 |
| **Taxonomy:** | [Salmo salar](http://www.ncbi.nlm.nih.gov/Taxonomy/Browser/wwwtax.cgi?lvl=0&id=8030) |

Sequence similarity is available as [an NCBI BLAST search of AAG18369 against nr](http://www.ncbi.nlm.nih.gov/blast/Blast.cgi?ALIGNMENTS=50;ALIGNMENT_VIEW=Pairwise;AUTO_FORMAT=Semiauto;CDD_SEARCH=on;CLIENT=web;COMPOSITION_BASED_STATISTICS=on;DATABASE=nr;DESCRIPTIONS=100;ENTREZ_QUERY=%28none%29;EXPECT=10;FILTER=L;FORMAT_BLOCK_ON_RESPAGE=None;FORMAT_OBJECT=Alignment;FORMAT_TYPE=HTML;GAPCOSTS=11%201;I_THRESH=0.001;LAYOUT=TwoWindows;MATRIX_NAME=BLOSUM62;NCBI_GI=on;PAGE=Proteins;PROGRAM=blastp;QUERY=MTFIMSFVWILMSLIHESRGQVTVTQTPAVKAVLTGQTVPLNCKTSSDVYQAGTSSPRLAWYQQKPGEAPKLLIYYATTLQSGTPSRFSGSGTHSDFTLTISGVQAEDAGDYYCQSFHYPNSKYVYTFGSATRLDVGSNSAPTLTVLPPSSEELSSTTTATLMCLANKGFPSDWTMSWKVDGNSKKQEASPGVLEKDGLYSWSSTLTLTAQEWTKAGEVTCEAQQISQTPVTKTLRRADCSG;SERVICE=plain;SET_DEFAULTS.x=9;SET_DEFAULTS.y=5;SHOW_OVERVIEW=on;WORD_SIZE=3;END_OF_HTTPGET=Yes).

**Search parameters**

| **MS data file:** | H:\MBA1-G6\52_BE4_01_1684.mgf |
| --- | --- |
| **Enzyme:** | Trypsin: cuts C-term side of KR unless next residue is P. |
| **Fixed modifications:** | [Carbamidomethyl (C)](http://fun-gen1.ibls.gla.ac.uk/mascot/cgi/client.pl?modification;file=..%2Fdata%2F20110930%2FF112305.dat;mod_name=Carbamidomethyl%20%28C%29) |
| **Variable modifications:** | [Oxidation (M)](http://fun-gen1.ibls.gla.ac.uk/mascot/cgi/client.pl?modification;file=..%2Fdata%2F20110930%2FF112305.dat;mod_name=Oxidation%20%28M%29) |

**Protein sequence coverage: 24%**

Matched peptides shown in ***bold red***.

| **1** | MTFIMSFVWI | LMSLIHESRG | QVTVTQTPAV | KAVLTGQTVP | LNCK**TSSDVY** |
| --- | --- | --- | --- | --- | --- |
| **51** | **QAGTSSPR**LA | WYQQKPGEAP | K**LLIYYATTL** | **QSGTPSR**FSG | SGTHSDFTLT |
| **101** | ISGVQAEDAG | DYYCQSFHYP | NSKYVYTFGS | ATRLDVGSNS | APTLTVLPPS |
| **151** | SEELSSTTTA | TLMCLANKGF | PSDWTMSWKV | DGNSK**KQEAS** | **PGVLEK**DGLY |
| **201** | SWSSTLTLTA | QEWTK**AGEVT** | **CEAQQISQTP** | **VTK**TLRRADC | SG |

**SPOT # 626**

**Protein View: AAG18369**

**Immunoglobulin light chain precursor.- Salmo salar (Atlantic salmon).**

Top of Form

| **Database:** | MSDB |
| --- | --- |
| **Score:** | 190 |
| **Nominal mass (M_r_):** | 26519 |
| **Calculated pI:** | 6.29 |
| **Taxonomy:** | [Salmo salar](http://www.ncbi.nlm.nih.gov/Taxonomy/Browser/wwwtax.cgi?lvl=0&id=8030) |

Sequence similarity is available as [an NCBI BLAST search of AAG18369 against nr](http://www.ncbi.nlm.nih.gov/blast/Blast.cgi?ALIGNMENTS=50;ALIGNMENT_VIEW=Pairwise;AUTO_FORMAT=Semiauto;CDD_SEARCH=on;CLIENT=web;COMPOSITION_BASED_STATISTICS=on;DATABASE=nr;DESCRIPTIONS=100;ENTREZ_QUERY=%28none%29;EXPECT=10;FILTER=L;FORMAT_BLOCK_ON_RESPAGE=None;FORMAT_OBJECT=Alignment;FORMAT_TYPE=HTML;GAPCOSTS=11%201;I_THRESH=0.001;LAYOUT=TwoWindows;MATRIX_NAME=BLOSUM62;NCBI_GI=on;PAGE=Proteins;PROGRAM=blastp;QUERY=MTFIMSFVWILMSLIHESRGQVTVTQTPAVKAVLTGQTVPLNCKTSSDVYQAGTSSPRLAWYQQKPGEAPKLLIYYATTLQSGTPSRFSGSGTHSDFTLTISGVQAEDAGDYYCQSFHYPNSKYVYTFGSATRLDVGSNSAPTLTVLPPSSEELSSTTTATLMCLANKGFPSDWTMSWKVDGNSKKQEASPGVLEKDGLYSWSSTLTLTAQEWTKAGEVTCEAQQISQTPVTKTLRRADCSG;SERVICE=plain;SET_DEFAULTS.x=9;SET_DEFAULTS.y=5;SHOW_OVERVIEW=on;WORD_SIZE=3;END_OF_HTTPGET=Yes).

**Search parameters**

| **MS data file:** | H:\MBA1-G6\53_BE5_01_1685.mgf |
| --- | --- |
| **Enzyme:** | Trypsin: cuts C-term side of KR unless next residue is P. |
| **Fixed modifications:** | [Carbamidomethyl (C)](http://fun-gen1.ibls.gla.ac.uk/mascot/cgi/client.pl?modification;file=..%2Fdata%2F20110930%2FF112306.dat;mod_name=Carbamidomethyl%20%28C%29) |
| **Variable modifications:** | [Oxidation (M)](http://fun-gen1.ibls.gla.ac.uk/mascot/cgi/client.pl?modification;file=..%2Fdata%2F20110930%2FF112306.dat;mod_name=Oxidation%20%28M%29) |

**Protein sequence coverage: 24%**

Matched peptides shown in ***bold red***.

| **1** | MTFIMSFVWI | LMSLIHESRG | QVTVTQTPAV | KAVLTGQTVP | LNCK**TSSDVY** |
| --- | --- | --- | --- | --- | --- |
| **51** | **QAGTSSPR**LA | WYQQKPGEAP | K**LLIYYATTL** | **QSGTPSR**FSG | SGTHSDFTLT |
| **101** | ISGVQAEDAG | DYYCQSFHYP | NSKYVYTFGS | ATRLDVGSNS | APTLTVLPPS |
| **151** | SEELSSTTTA | TLMCLANKGF | PSDWTMSWKV | DGNSK**KQEAS** | **PGVLEK**DGLY |
| **201** | SWSSTLTLTA | QEWTK**AGEVT** | **CEAQQISQTP** | **VTK**TLRRADC | SG |

Bottom of Form

**SPOT # 741**

**Protein View: Q7ZZQ7_OREMO**

**Nucleoside diphosphate kinase.- Oreochromis mossambicus (Mozambique tilapia) (Tilapia mossambica).**

Top of Form

| **Database:** | MSDB |
| --- | --- |
| **Score:** | 58 |
| **Nominal mass (M_r_):** | 17328 |
| **Calculated pI:** | 5.93 |
| **Taxonomy:** | [Oreochromis mossambicus](http://www.ncbi.nlm.nih.gov/Taxonomy/Browser/wwwtax.cgi?lvl=0&id=8127) |

This protein sequence matches the following other entries:

- AAP13059 from [Oreochromis mossambicus](http://www.ncbi.nlm.nih.gov/Taxonomy/Browser/wwwtax.cgi?lvl=0&id=8127)

Sequence similarity is available as [an NCBI BLAST search of Q7ZZQ7_OREMO against nr](http://www.ncbi.nlm.nih.gov/blast/Blast.cgi?ALIGNMENTS=50;ALIGNMENT_VIEW=Pairwise;AUTO_FORMAT=Semiauto;CDD_SEARCH=on;CLIENT=web;COMPOSITION_BASED_STATISTICS=on;DATABASE=nr;DESCRIPTIONS=100;ENTREZ_QUERY=%28none%29;EXPECT=10;FILTER=L;FORMAT_BLOCK_ON_RESPAGE=None;FORMAT_OBJECT=Alignment;FORMAT_TYPE=HTML;GAPCOSTS=11%201;I_THRESH=0.001;LAYOUT=TwoWindows;MATRIX_NAME=BLOSUM62;NCBI_GI=on;PAGE=Proteins;PROGRAM=blastp;QUERY=MAELKERTFIAIKPDGVQRGIIGEVIKRFEMKGFKLVGMKMLHASEDLLMEHYVDLKDRPFFPTLINYMRSGPVVAMVWEDKGVVKTGRVMLGETNPADSKPGTIRGDFCIDVSKNIIHGSDSVESANKEISLWFKDDELVNYSSCAFSWLY;SERVICE=plain;SET_DEFAULTS.x=9;SET_DEFAULTS.y=5;SHOW_OVERVIEW=on;WORD_SIZE=3;END_OF_HTTPGET=Yes).

**Search parameters**

| **MS data file:** | H:\MBA1-G6\54_BE6_01_1686.mgf |
| --- | --- |
| **Enzyme:** | Trypsin: cuts C-term side of KR unless next residue is P. |
| **Fixed modifications:** | [Carbamidomethyl (C)](http://fun-gen1.ibls.gla.ac.uk/mascot/cgi/client.pl?modification;file=..%2Fdata%2F20110930%2FF112307.dat;mod_name=Carbamidomethyl%20%28C%29) |
| **Variable modifications:** | [Oxidation (M)](http://fun-gen1.ibls.gla.ac.uk/mascot/cgi/client.pl?modification;file=..%2Fdata%2F20110930%2FF112307.dat;mod_name=Oxidation%20%28M%29) |

**Protein sequence coverage: 22%**

Matched peptides shown in ***bold red***.

| **1** | MAELKER**TFI** | **AIKPDGVQRG** | **IIGEVIK**RFE | MKGFKLVGMK | MLHASEDLLM |
| --- | --- | --- | --- | --- | --- |
| **51** | EHYVDLKDRP | FFPTLINYMR | SGPVVAMVWE | DKGVVKTGRV | MLGETNPADS |
| **101** | KPGTIRGDFC | IDVSK**NIIHG** | **SDSVESANK**E | ISLWFKDDEL | VNYSSCAFSW |
| **151** | LY |  |  |  |  |

**SPOT # 201**

**Protein View: Q9PTA8_SALSA**

**Antithrombin precursor.- Salmo salar (Atlantic salmon).**

Top of Form

| **Database:** | MSDB |
| --- | --- |
| **Score:** | 156 |
| **Nominal mass (M_r_):** | 51781 |
| **Calculated pI:** | 7.55 |
| **Taxonomy:** | [Salmo salar](http://www.ncbi.nlm.nih.gov/Taxonomy/Browser/wwwtax.cgi?lvl=0&id=8030) |

This protein sequence matches the following other entries:

- CAB64714 from [Salmo salar](http://www.ncbi.nlm.nih.gov/Taxonomy/Browser/wwwtax.cgi?lvl=0&id=8030)

Sequence similarity is available as [an NCBI BLAST search of Q9PTA8_SALSA against nr](http://www.ncbi.nlm.nih.gov/blast/Blast.cgi?ALIGNMENTS=50;ALIGNMENT_VIEW=Pairwise;AUTO_FORMAT=Semiauto;CDD_SEARCH=on;CLIENT=web;COMPOSITION_BASED_STATISTICS=on;DATABASE=nr;DESCRIPTIONS=100;ENTREZ_QUERY=%28none%29;EXPECT=10;FILTER=L;FORMAT_BLOCK_ON_RESPAGE=None;FORMAT_OBJECT=Alignment;FORMAT_TYPE=HTML;GAPCOSTS=11%201;I_THRESH=0.001;LAYOUT=TwoWindows;MATRIX_NAME=BLOSUM62;NCBI_GI=on;PAGE=Proteins;PROGRAM=blastp;QUERY=MRLPQFVWTLGLLLPLLSTSQAFKDICNAKPKDVPLEPRCIYRSPEDEAPTGDAIPEKVPENTNPRVWELSKANSRFALSLFKQLAQGKPSESNIFMSPISISSAFAMTKLGACNNTLKQIMNVFEFDTIKEKTSDQVHFFFAKLNCRLYRKKDKTTELISANRLFGEKSLAFNEIYQNISELVYGAKLMPLNFKEKPELSRVTINDWIANKTENRIQNTLPKDSLNSNTVLVLVNTIYFKGQWKSKFDKKNVFKADFYVSKSKTCPVSMMYQETKFHYGRFTEDKVQVLELPYRGDDITMVLILPLKDTPLSEVEENLDLKKLTGWLHNMRETSVSVHLPRFRIEDSFSLKEKLQAMGLEDLFSPKDASLPGILEDEANYLYISDAFHKAFLEVNEEGSEAAAATAVMAVGRSINSNREMFVANKPFLLLIRESTINTMVFTGRVADPCDP;SERVICE=plain;SET_DEFAULTS.x=9;SET_DEFAULTS.y=5;SHOW_OVERVIEW=on;WORD_SIZE=3;END_OF_HTTPGET=Yes).

**Search parameters**

| **MS data file:** | H:\MBA1-G6\55_BE7_01_1687.mgf |
| --- | --- |
| **Enzyme:** | Trypsin: cuts C-term side of KR unless next residue is P. |
| **Fixed modifications:** | [Carbamidomethyl (C)](http://fun-gen1.ibls.gla.ac.uk/mascot/cgi/client.pl?modification;file=..%2Fdata%2F20110930%2FF112308.dat;mod_name=Carbamidomethyl%20%28C%29) |
| **Variable modifications:** | [Oxidation (M)](http://fun-gen1.ibls.gla.ac.uk/mascot/cgi/client.pl?modification;file=..%2Fdata%2F20110930%2FF112308.dat;mod_name=Oxidation%20%28M%29) |

**Protein sequence coverage: 21%**

Matched peptides shown in ***bold red***.

| **1** | MRLPQFVWTL | GLLLPLLSTS | QAFKDICNAK | PKDVPLEPRC | IYRSPEDEAP |
| --- | --- | --- | --- | --- | --- |
| **51** | TGDAIPEKVP | ENTNPRVWEL | SKANSR**FALS** | **LFK**QLAQGKP | SESNIFMSPI |
| **101** | SISSAFAMTK | LGACNNTLKQ | IMNVFEFDTI | KEK**TSDQVHF** | **FFAK**LNCRLY |
| **151** | RKKDKTTELI | SANRLFGEKS | LAFNEIYQNI | SELVYGAKLM | PLNFKEKPEL |
| **201** | SRVTINDWIA | NKTENRIQNT | LPKDSLNSNT | VLVLVNTIYF | KGQWKSKFDK |
| **251** | KNVFKADFYV | SKSK**TCPVSM** | **MYQETK**FHYG | R**FTEDKVQVL** | **ELPYRGDDIT** |
| **301** | **MVLILPLK**DT | PLSEVEENLD | LKKLTGWLHN | MRETSVSVHL | PRFRIEDSFS |
| **351** | LKEK**LQAMGL** | **EDLFSPK**DAS | LPGILEDEAN | YLYISDAFHK | AFLEVNEEGS |
| **401** | EAAAATAVMA | VGRSINSNR**E** | **MFVANKPFLL** | **LIRESTINTM** | **VFTGR**VADPC |
| **451** | DP |  |  |  |  |

Bottom of Form

**Protein View: Q4QZ25_ONCMY**

**Complement component C9.- Oncorhynchus mykiss (Rainbow trout) (Salmo gairdneri).**

Top of Form

| **Database:** | MSDB |
| --- | --- |
| **Score:** | 196 |
| **Nominal mass (M_r_):** | 68118 |
| **Calculated pI:** | 5.46 |
| **Taxonomy:** | [Oncorhynchus mykiss](http://www.ncbi.nlm.nih.gov/Taxonomy/Browser/wwwtax.cgi?lvl=0&id=8022) |

This protein sequence matches the following other entries:

- CAJ01692 from [Oncorhynchus mykiss](http://www.ncbi.nlm.nih.gov/Taxonomy/Browser/wwwtax.cgi?lvl=0&id=8022)

Sequence similarity is available as [an NCBI BLAST search of Q4QZ25_ONCMY against nr](http://www.ncbi.nlm.nih.gov/blast/Blast.cgi?ALIGNMENTS=50;ALIGNMENT_VIEW=Pairwise;AUTO_FORMAT=Semiauto;CDD_SEARCH=on;CLIENT=web;COMPOSITION_BASED_STATISTICS=on;DATABASE=nr;DESCRIPTIONS=100;ENTREZ_QUERY=%28none%29;EXPECT=10;FILTER=L;FORMAT_BLOCK_ON_RESPAGE=None;FORMAT_OBJECT=Alignment;FORMAT_TYPE=HTML;GAPCOSTS=11%201;I_THRESH=0.001;LAYOUT=TwoWindows;MATRIX_NAME=BLOSUM62;NCBI_GI=on;PAGE=Proteins;PROGRAM=blastp;QUERY=MAEVAAALLLGFCAVAVSVSVSGDENRTGKRDTREVKVAPPDPVDCVWSRWSEWTPCNSCTKIRHRSRSVEVFGQFGGKPCQGQPIGEQQACTSDAVCEQALPSECSSTEFTCESGACIKLRLSCNGDYDCEDGSDEDCEPVRKPCGTKLYDTNEQGRTAGYGINILGMEPRINPFNNDYFNGMCNKVKNINNNEYNRLPWNVGLLNYETIAEETVSKEIYEDTYTLLRELMTETKLTVSAGLNLKFTPTEKSMAKSNTTVSGGVGLDAEYDRTQMIKEVSEYTTIKNKSFMRVNGRLQLSTYRMRSRDLQVAGEFLEHVKSLPLEYEKGQYFSFLEDYGTHYTRNGKSGGEYQLVYVLNQDTIKDKKLTERKLQDCIKVGISANFDTNIGIGGDAHIRPGHCKDTVNKNTAEKEGKALVDKVITLVRGGTLEAAVAMRTQITKEGLMDVTTYQNWARTVGDAPALLSSEPEPIQTLIPLSMPDANTRRLNMQRATQEYEAEYSVCKCKPCHNGGSLALLDGKCLCLCLPQFEGLACQDAKADNNKNTKTPVESVPQEGNWSCWAAWSGCSGGKRIRTRSCNTQGLSDATCRGDIVTEDYC;SERVICE=plain;SET_DEFAULTS.x=9;SET_DEFAULTS.y=5;SHOW_OVERVIEW=on;WORD_SIZE=3;END_OF_HTTPGET=Yes).

**Search parameters**

| **MS data file:** | H:\MBA1-G6\55_BE7_01_1687.mgf |
| --- | --- |
| **Enzyme:** | Trypsin: cuts C-term side of KR unless next residue is P. |
| **Fixed modifications:** | [Carbamidomethyl (C)](http://fun-gen1.ibls.gla.ac.uk/mascot/cgi/client.pl?modification;file=..%2Fdata%2F20110930%2FF112308.dat;mod_name=Carbamidomethyl%20%28C%29) |
| **Variable modifications:** | [Oxidation (M)](http://fun-gen1.ibls.gla.ac.uk/mascot/cgi/client.pl?modification;file=..%2Fdata%2F20110930%2FF112308.dat;mod_name=Oxidation%20%28M%29) |

**Protein sequence coverage: 14%**

Matched peptides shown in ***bold red***.

| **1** | MAEVAAALLL | GFCAVAVSVS | VSGDENRTGK | RDTREVKVAP | PDPVDCVWSR |
| --- | --- | --- | --- | --- | --- |
| **51** | WSEWTPCNSC | TKIRHRSRSV | EVFGQFGGKP | CQGQPIGEQQ | ACTSDAVCEQ |
| **101** | ALPSECSSTE | FTCESGACIK | LRLSCNGDYD | CEDGSDEDCE | PVRKPCGTKL |
| **151** | YDTNEQGR**TA** | **GYGINILGME** | **PR**INPFNNDY | FNGMCNKVKN | INNNEYNRLP |
| **201** | WNVGLLNYET | IAEETVSK**EI** | **YEDTYTLLR**E | LMTETKLTVS | AGLNLKFTPT |
| **251** | EKSMAK**SNTT** | **VSGGVGLDAE** | **YDRTQMIK**EV | SEYTTIKNKS | FMRVNGRLQL |
| **301** | STYRMRSR**DL** | **QVAGEFLEHV** | **K**SLPLEYEK**G** | **QYFSFLEDYG** | **THYTR**NGKSG |
| **351** | GEYQLVYVLN | QDTIKDKKLT | ERKLQDCIKV | GISANFDTNI | GIGGDAHIRP |
| **401** | GHCKDTVNKN | TAEKEGKALV | DKVITLVRGG | TLEAAVAMRT | QITKEGLMDV |
| **451** | TTYQNWARTV | GDAPALLSSE | PEPIQTLIPL | SMPDANTRRL | NMQR**ATQEYE** |
| **501** | **AEYSVCK**CKP | CHNGGSLALL | DGKCLCLCLP | QFEGLACQDA | KADNNKNTKT |
| **551** | PVESVPQEGN | WSCWAAWSGC | SGGKRIRTRS | CNTQGLSDAT | CRGDIVTEDY |
| **601** | C |  |  |  |  |

**SPOT # 624**

**Protein View: Q70I40_ONCMY**

**Triosephosphate isomerase (Fragment).- Oncorhynchus mykiss (Rainbow trout) (Salmo gairdneri).**

Top of Form

| **Database:** | MSDB |
| --- | --- |
| **Score:** | 194 |
| **Nominal mass (M_r_):** | 22759 |
| **Calculated pI:** | 6.59 |
| **Taxonomy:** | [Oncorhynchus mykiss](http://www.ncbi.nlm.nih.gov/Taxonomy/Browser/wwwtax.cgi?lvl=0&id=8022) |

This protein sequence matches the following other entries:

- CAE45565 from [Oncorhynchus mykiss](http://www.ncbi.nlm.nih.gov/Taxonomy/Browser/wwwtax.cgi?lvl=0&id=8022)

Sequence similarity is available as [an NCBI BLAST search of Q70I40_ONCMY against nr](http://www.ncbi.nlm.nih.gov/blast/Blast.cgi?ALIGNMENTS=50;ALIGNMENT_VIEW=Pairwise;AUTO_FORMAT=Semiauto;CDD_SEARCH=on;CLIENT=web;COMPOSITION_BASED_STATISTICS=on;DATABASE=nr;DESCRIPTIONS=100;ENTREZ_QUERY=%28none%29;EXPECT=10;FILTER=L;FORMAT_BLOCK_ON_RESPAGE=None;FORMAT_OBJECT=Alignment;FORMAT_TYPE=HTML;GAPCOSTS=11%201;I_THRESH=0.001;LAYOUT=TwoWindows;MATRIX_NAME=BLOSUM62;NCBI_GI=on;PAGE=Proteins;PROGRAM=blastp;QUERY=GDKASLGELIKTLNSAKLDPNTEVVCGAPSIYLEFARAKLDPKIGVAAQNCYKVKGGAFTGEISPAMIKDVGVHWVILGHSERRWVFGETDELIGQKCAHALENGLGVIACIGEKLDEREAGITEKVINAQTKHFADNIKDWSKVVLAYEPVWAIGTGKTASPAQAQDVHDKLRQWVKANVSEAVANSVRIIYGGSVTGGTCKELGGMKD;SERVICE=plain;SET_DEFAULTS.x=9;SET_DEFAULTS.y=5;SHOW_OVERVIEW=on;WORD_SIZE=3;END_OF_HTTPGET=Yes).

**Search parameters**

| **MS data file:** | H:\MBA1-G6\56_BE8_01_1688.mgf |
| --- | --- |
| **Enzyme:** | Trypsin: cuts C-term side of KR unless next residue is P. |
| **Fixed modifications:** | [Carbamidomethyl (C)](http://fun-gen1.ibls.gla.ac.uk/mascot/cgi/client.pl?modification;file=..%2Fdata%2F20110930%2FF112309.dat;mod_name=Carbamidomethyl%20%28C%29) |
| **Variable modifications:** | [Oxidation (M)](http://fun-gen1.ibls.gla.ac.uk/mascot/cgi/client.pl?modification;file=..%2Fdata%2F20110930%2FF112309.dat;mod_name=Oxidation%20%28M%29) |

**Protein sequence coverage: 50%**

Matched peptides shown in ***bold red***.

| **1** | GDK**ASLGELI** | **K**TLNSAK**LDP** | **NTEVVCGAPS** | **IYLEFAR**AKL | DPKIGVAAQN |
| --- | --- | --- | --- | --- | --- |
| **51** | CYKVK**GGAFT** | **GEISPAMIK**D | VGVHWVILGH | SERRWVFGET | DELIGQK**CAH** |
| **101** | **ALENGLGVIA** | **CIGEK**LDERE | AGITEKVINA | QTK**HFADNIK** | DWSK**VVLAYE** |
| **151** | **PVWAIGTGKT** | **ASPAQAQDVH** | **DK**LRQWVK**AN** | **VSEAVANSVR** | IIYGGSVTGG |
| **201** | TCKELGGMKD |  |  |  |  |

**SPOT #74**

**NO SIGNIFICANT RESULTS**

**SPOT #738**

**Protein View: Q9DFC0_ICTPU**

**Putative oncoprotein nm23.- Ictalurus punctatus (Channel catfish).**

Top of Form

| **Database:** | MSDB |
| --- | --- |
| **Score:** | 58 |
| **Nominal mass (M_r_):** | 17315 |
| **Calculated pI:** | 8.52 |
| **Taxonomy:** | [Ictalurus punctatus](http://www.ncbi.nlm.nih.gov/Taxonomy/Browser/wwwtax.cgi?lvl=0&id=7998) |

This protein sequence matches the following other entries:

- AAG14350 from [Ictalurus punctatus](http://www.ncbi.nlm.nih.gov/Taxonomy/Browser/wwwtax.cgi?lvl=0&id=7998)

Sequence similarity is available as [an NCBI BLAST search of Q9DFC0_ICTPU against nr](http://www.ncbi.nlm.nih.gov/blast/Blast.cgi?ALIGNMENTS=50;ALIGNMENT_VIEW=Pairwise;AUTO_FORMAT=Semiauto;CDD_SEARCH=on;CLIENT=web;COMPOSITION_BASED_STATISTICS=on;DATABASE=nr;DESCRIPTIONS=100;ENTREZ_QUERY=%28none%29;EXPECT=10;FILTER=L;FORMAT_BLOCK_ON_RESPAGE=None;FORMAT_OBJECT=Alignment;FORMAT_TYPE=HTML;GAPCOSTS=11%201;I_THRESH=0.001;LAYOUT=TwoWindows;MATRIX_NAME=BLOSUM62;NCBI_GI=on;PAGE=Proteins;PROGRAM=blastp;QUERY=MAAKAERTFIAIKPDGVQRGLIGDIIKRFEQKGFRLVALKFLQASEDLLKQHYIDLKDRPFYPGLVKYMSSGPVVAMVWEGLNVVKTGRVMLGETNPADSKPGTIRGDFCIEVGRNIIHGSDSVESANKEISLWFKPEELVSFKSCAFNWIYE;SERVICE=plain;SET_DEFAULTS.x=9;SET_DEFAULTS.y=5;SHOW_OVERVIEW=on;WORD_SIZE=3;END_OF_HTTPGET=Yes).

**Search parameters**

| **MS data file:** | H:\MBA1-G6\58_BE10_01_1690.mgf |
| --- | --- |
| **Enzyme:** | Trypsin: cuts C-term side of KR unless next residue is P. |
| **Fixed modifications:** | [Carbamidomethyl (C)](http://fun-gen1.ibls.gla.ac.uk/mascot/cgi/client.pl?modification;file=..%2Fdata%2F20110930%2FF112311.dat;mod_name=Carbamidomethyl%20%28C%29) |
| **Variable modifications:** | [Oxidation (M)](http://fun-gen1.ibls.gla.ac.uk/mascot/cgi/client.pl?modification;file=..%2Fdata%2F20110930%2FF112311.dat;mod_name=Oxidation%20%28M%29) |

**Protein sequence coverage: 22%**

Matched peptides shown in ***bold red***.

| **1** | MAAKAER**TFI** | **AIKPDGVQRG** | **LIGDIIK**RFE | QKGFRLVALK | FLQASEDLLK |
| --- | --- | --- | --- | --- | --- |
| **51** | QHYIDLKDRP | FYPGLVKYMS | SGPVVAMVWE | GLNVVKTGRV | MLGETNPADS |
| **101** | KPGTIRGDFC | IEVGR**NIIHG** | **SDSVESANK**E | ISLWFKPEEL | VSFKSCAFNW |
| **151** | IYE |  |  |  |  |

**SPOT # 45**

**Complement factor H precursor.- Oncorhynchus mykiss (Rainbow trout) (Salmo gairdneri).**

Top of Form

| **Database:** | MSDB |
| --- | --- |
| **Score:** | 79 |
| **Nominal mass (M_r_):** | 96036 |
| **Calculated pI:** | 6.47 |
| **Taxonomy:** | [Oncorhynchus mykiss](http://www.ncbi.nlm.nih.gov/Taxonomy/Browser/wwwtax.cgi?lvl=0&id=8022) |

This protein sequence matches the following other entries:

- CAF25505 from [Oncorhynchus mykiss](http://www.ncbi.nlm.nih.gov/Taxonomy/Browser/wwwtax.cgi?lvl=0&id=8022)

Sequence similarity is available as [an NCBI BLAST search of Q2L4Q6_ONCMY against nr](http://www.ncbi.nlm.nih.gov/blast/Blast.cgi?ALIGNMENTS=50;ALIGNMENT_VIEW=Pairwise;AUTO_FORMAT=Semiauto;CDD_SEARCH=on;CLIENT=web;COMPOSITION_BASED_STATISTICS=on;DATABASE=nr;DESCRIPTIONS=100;ENTREZ_QUERY=%28none%29;EXPECT=10;FILTER=L;FORMAT_BLOCK_ON_RESPAGE=None;FORMAT_OBJECT=Alignment;FORMAT_TYPE=HTML;GAPCOSTS=11%201;I_THRESH=0.001;LAYOUT=TwoWindows;MATRIX_NAME=BLOSUM62;NCBI_GI=on;PAGE=Proteins;PROGRAM=blastp;QUERY=MRLITLGFVFALWLCTLSFVKGQVCFRKGVSEIPEAKNVDISNLEDTEIDKTVRLPCAIGYVGFVRLKCGSKGWSKDGGRKCEPKSCGHPGDTPNGDFHLSILDDFVFGAQVLYQCRKGYQMVTRSRHRTCVEQGWDSALPICEALKCPVIQANDNVVVIGNSEDATYGNVIQFECQSNHMVLNGSSEMVCNDKGEWSSTVPTCKVIKCYAPDIANGAVSGPPKEDYDEDDTLRYSCNTKYVKSQERVPKCTKMVNSANWSPTPACEEVRCKLSLPPTRGTSYRPADRNLFLPDERVTVTCASGFWNSISRQTENTMTCKEDGKWSPSTTDCERITCGDPRDPLVSSPYYWQRGQFRGTQRYNCRTGFKTTNPRGVATCTSDGSWTPKPLCEEITCDKPDILNAVIKDPKTRYKINDLLTYECKMNYELLDSTTRPTATCTTNGWTKTLGCKEIEGACIKPNVMNGFIVQSNERNVDPRNSKIYSSCNEGFKPSTGGWWGEATCTEGTWSGILECIDQSQCGRIPVIPNTIKVPHSEVYDNEQTVTIDCKVGYTSETKTIKCKDGEWQTPLPACRLQGVPCDPPPKVENAIVKIPYQNKYREGFEVNYECRKSFRIEGHKKLTCENGSWTTPPPTCKQYCGKPEGAGKQIQILDQELERYENGNQIDYTCINPYKGPGGTATCNNGEWHMPIECKASCPDPPPITNGDFTKEKRDVEGVITEVSYQCSRQFTLSFTGNIRCLDGKWQSPPKCLRPCEISTFDAEYNLQNLPEKDNIAHGEKKTLHCKEGYYHQYKRFRPNIEEIEMKCDDGELQYGEHMPICRYRYS;SERVICE=plain;SET_DEFAULTS.x=9;SET_DEFAULTS.y=5;SHOW_OVERVIEW=on;WORD_SIZE=3;END_OF_HTTPGET=Yes).

**Search parameters**

| **MS data file:** | H:\MBA1-G6\59_BE11_01_1691.mgf |
| --- | --- |
| **Enzyme:** | Trypsin: cuts C-term side of KR unless next residue is P. |
| **Fixed modifications:** | [Carbamidomethyl (C)](http://fun-gen1.ibls.gla.ac.uk/mascot/cgi/client.pl?modification;file=..%2Fdata%2F20110930%2FF112312.dat;mod_name=Carbamidomethyl%20%28C%29) |
| **Variable modifications:** | [Oxidation (M)](http://fun-gen1.ibls.gla.ac.uk/mascot/cgi/client.pl?modification;file=..%2Fdata%2F20110930%2FF112312.dat;mod_name=Oxidation%20%28M%29) |

**Protein sequence coverage: 3%**

Matched peptides shown in ***bold red***.

| **1** | MRLITLGFVF | ALWLCTLSFV | KGQVCFRKGV | SEIPEAK**NVD** | **ISNLEDTEID** |
| --- | --- | --- | --- | --- | --- |
| **51** | **K**TVR**LPCAIG** | **YVGFVR**LKCG | SKGWSKDGGR | KCEPKSCGHP | GDTPNGDFHL |
| **101** | SILDDFVFGA | QVLYQCRKGY | QMVTRSRHRT | CVEQGWDSAL | PICEALKCPV |
| **151** | IQANDNVVVI | GNSEDATYGN | VIQFECQSNH | MVLNGSSEMV | CNDKGEWSST |
| **201** | VPTCKVIKCY | APDIANGAVS | GPPKEDYDED | DTLRYSCNTK | YVKSQERVPK |
| **251** | CTKMVNSANW | SPTPACEEVR | CKLSLPPTRG | TSYRPADRNL | FLPDERVTVT |
| **301** | CASGFWNSIS | RQTENTMTCK | EDGKWSPSTT | DCERITCGDP | RDPLVSSPYY |
| **351** | WQRGQFRGTQ | RYNCRTGFKT | TNPRGVATCT | SDGSWTPKPL | CEEITCDKPD |
| **401** | ILNAVIKDPK | TRYKINDLLT | YECKMNYELL | DSTTRPTATC | TTNGWTKTLG |
| **451** | CKEIEGACIK | PNVMNGFIVQ | SNERNVDPRN | SKIYSSCNEG | FKPSTGGWWG |
| **501** | EATCTEGTWS | GILECIDQSQ | CGRIPVIPNT | IKVPHSEVYD | NEQTVTIDCK |
| **551** | VGYTSETKTI | KCKDGEWQTP | LPACRLQGVP | CDPPPKVENA | IVKIPYQNKY |
| **601** | REGFEVNYEC | RKSFRIEGHK | KLTCENGSWT | TPPPTCKQYC | GKPEGAGKQI |
| **651** | QILDQELERY | ENGNQIDYTC | INPYKGPGGT | ATCNNGEWHM | PIECKASCPD |
| **701** | PPPITNGDFT | KEKRDVEGVI | TEVSYQCSRQ | FTLSFTGNIR | CLDGKWQSPP |
| **751** | KCLRPCEIST | FDAEYNLQNL | PEKDNIAHGE | KKTLHCKEGY | YHQYKRFRPN |
| **801** | IEEIEMKCDD | GELQYGEHMP | ICRYRYS |  |  |

**SPOT # 47**

**Protein View: Q9DDG6_SALTR**

**Alpha-2 enolase-1 (Fragment).- Salmo trutta (Brown trout).**

Top of Form

| **Database:** | MSDB |
| --- | --- |
| **Score:** | 111 |
| **Nominal mass (M_r_):** | 39667 |
| **Calculated pI:** | 5.78 |
| **Taxonomy:** | [Salmo trutta](http://www.ncbi.nlm.nih.gov/Taxonomy/Browser/wwwtax.cgi?lvl=0&id=8032) |

This protein sequence matches the following other entries:

- AAG16311 from [Salmo trutta](http://www.ncbi.nlm.nih.gov/Taxonomy/Browser/wwwtax.cgi?lvl=0&id=8032)

Sequence similarity is available as [an NCBI BLAST search of Q9DDG6_SALTR against nr](http://www.ncbi.nlm.nih.gov/blast/Blast.cgi?ALIGNMENTS=50;ALIGNMENT_VIEW=Pairwise;AUTO_FORMAT=Semiauto;CDD_SEARCH=on;CLIENT=web;COMPOSITION_BASED_STATISTICS=on;DATABASE=nr;DESCRIPTIONS=100;ENTREZ_QUERY=%28none%29;EXPECT=10;FILTER=L;FORMAT_BLOCK_ON_RESPAGE=None;FORMAT_OBJECT=Alignment;FORMAT_TYPE=HTML;GAPCOSTS=11%201;I_THRESH=0.001;LAYOUT=TwoWindows;MATRIX_NAME=BLOSUM62;NCBI_GI=on;PAGE=Proteins;PROGRAM=blastp;QUERY=TKKGLFRAAVPSGASTGIYEALELRDNDKTRYLGKGVKRAVKHINEFLAPALCNQNVNVLEQEKVDKLMLDMDGTENKSKFGANAILGVSLAVCKAGAAEKGVPLYRHIADLAGNPNXILPCPAFNVINGGSHAGNKLAMQEFMILPIGASNFHEAMRIGAEVYHNLKNVIKAKYGKDATNVGDEGGFAPNILENNEALELLKSAIEKAGYPDKIIIGMDVAASEFYKAGKYDLDFKSPDDPARYITXDQLGDLYKSFIKGYPVQSIEDPFDQDDWAAWSKFTAAVDIQVVGDDLTVTNPKRIQQAVEKKACNCLLLKVNQIGSVTESIKACKLAQSNGWGVMVSHRSGETEDTFIADLVVGL;SERVICE=plain;SET_DEFAULTS.x=9;SET_DEFAULTS.y=5;SHOW_OVERVIEW=on;WORD_SIZE=3;END_OF_HTTPGET=Yes).

**Search parameters**

| **MS data file:** | H:\MBA1-G6\60_BE12_01_1692.mgf |
| --- | --- |
| **Enzyme:** | Trypsin: cuts C-term side of KR unless next residue is P. |
| **Fixed modifications:** | [Carbamidomethyl (C)](http://fun-gen1.ibls.gla.ac.uk/mascot/cgi/client.pl?modification;file=..%2Fdata%2F20110930%2FF112313.dat;mod_name=Carbamidomethyl%20%28C%29) |
| **Variable modifications:** | [Oxidation (M)](http://fun-gen1.ibls.gla.ac.uk/mascot/cgi/client.pl?modification;file=..%2Fdata%2F20110930%2FF112313.dat;mod_name=Oxidation%20%28M%29) |

**Protein sequence coverage: 9%**

Matched peptides shown in ***bold red***.

| **1** | TKKGLFRAAV | PSGASTGIYE | ALELRDNDKT | RYLGKGVKRA | VKHINEFLAP |
| --- | --- | --- | --- | --- | --- |
| **51** | ALCNQNVNVL | EQEKVDKLML | DMDGTENKSK | FGANAILGVS | LAVCKAGAAE |
| **101** | KGVPLYRHIA | DLAGNPNXIL | PCPAFNVING | GSHAGNKLAM | QEFMILPIGA |
| **151** | SNFHEAMR**IG** | **AEVYHNLK**NV | IKAKYGKDAT | NVGDEGGFAP | NILENNEALE |
| **201** | LLKSAIEKAG | YPDK**IIIGMD** | **VAASEFYK**AG | KYDLDFKSPD | DPAR**YITXDQ** |
| **251** | **LGDLYK**SFIK | GYPVQSIEDP | FDQDDWAAWS | KFTAAVDIQV | VGDDLTVTNP |
| **301** | KRIQQAVEKK | ACNCLLLKVN | QIGSVTESIK | ACKLAQSNGW | GVMVSHRSGE |
| **351** | TEDTFIADLV | VGL |  |  |  |

**Protein View: Q2L4Q6_ONCMY**

**Complement factor H precursor.- Oncorhynchus mykiss (Rainbow trout) (Salmo gairdneri).**

Top of Form

| **Database:** | MSDB |
| --- | --- |
| **Score:** | 94 |
| **Nominal mass (M_r_):** | 96036 |
| **Calculated pI:** | 6.47 |
| **Taxonomy:** | [Oncorhynchus mykiss](http://www.ncbi.nlm.nih.gov/Taxonomy/Browser/wwwtax.cgi?lvl=0&id=8022) |

This protein sequence matches the following other entries:

- CAF25505 from [Oncorhynchus mykiss](http://www.ncbi.nlm.nih.gov/Taxonomy/Browser/wwwtax.cgi?lvl=0&id=8022)

Sequence similarity is available as [an NCBI BLAST search of Q2L4Q6_ONCMY against nr](http://www.ncbi.nlm.nih.gov/blast/Blast.cgi?ALIGNMENTS=50;ALIGNMENT_VIEW=Pairwise;AUTO_FORMAT=Semiauto;CDD_SEARCH=on;CLIENT=web;COMPOSITION_BASED_STATISTICS=on;DATABASE=nr;DESCRIPTIONS=100;ENTREZ_QUERY=%28none%29;EXPECT=10;FILTER=L;FORMAT_BLOCK_ON_RESPAGE=None;FORMAT_OBJECT=Alignment;FORMAT_TYPE=HTML;GAPCOSTS=11%201;I_THRESH=0.001;LAYOUT=TwoWindows;MATRIX_NAME=BLOSUM62;NCBI_GI=on;PAGE=Proteins;PROGRAM=blastp;QUERY=MRLITLGFVFALWLCTLSFVKGQVCFRKGVSEIPEAKNVDISNLEDTEIDKTVRLPCAIGYVGFVRLKCGSKGWSKDGGRKCEPKSCGHPGDTPNGDFHLSILDDFVFGAQVLYQCRKGYQMVTRSRHRTCVEQGWDSALPICEALKCPVIQANDNVVVIGNSEDATYGNVIQFECQSNHMVLNGSSEMVCNDKGEWSSTVPTCKVIKCYAPDIANGAVSGPPKEDYDEDDTLRYSCNTKYVKSQERVPKCTKMVNSANWSPTPACEEVRCKLSLPPTRGTSYRPADRNLFLPDERVTVTCASGFWNSISRQTENTMTCKEDGKWSPSTTDCERITCGDPRDPLVSSPYYWQRGQFRGTQRYNCRTGFKTTNPRGVATCTSDGSWTPKPLCEEITCDKPDILNAVIKDPKTRYKINDLLTYECKMNYELLDSTTRPTATCTTNGWTKTLGCKEIEGACIKPNVMNGFIVQSNERNVDPRNSKIYSSCNEGFKPSTGGWWGEATCTEGTWSGILECIDQSQCGRIPVIPNTIKVPHSEVYDNEQTVTIDCKVGYTSETKTIKCKDGEWQTPLPACRLQGVPCDPPPKVENAIVKIPYQNKYREGFEVNYECRKSFRIEGHKKLTCENGSWTTPPPTCKQYCGKPEGAGKQIQILDQELERYENGNQIDYTCINPYKGPGGTATCNNGEWHMPIECKASCPDPPPITNGDFTKEKRDVEGVITEVSYQCSRQFTLSFTGNIRCLDGKWQSPPKCLRPCEISTFDAEYNLQNLPEKDNIAHGEKKTLHCKEGYYHQYKRFRPNIEEIEMKCDDGELQYGEHMPICRYRYS;SERVICE=plain;SET_DEFAULTS.x=9;SET_DEFAULTS.y=5;SHOW_OVERVIEW=on;WORD_SIZE=3;END_OF_HTTPGET=Yes).

**Search parameters**

| **MS data file:** | H:\MBA1-G6\60_BE12_01_1692.mgf |
| --- | --- |
| **Enzyme:** | Trypsin: cuts C-term side of KR unless next residue is P. |
| **Fixed modifications:** | [Carbamidomethyl (C)](http://fun-gen1.ibls.gla.ac.uk/mascot/cgi/client.pl?modification;file=..%2Fdata%2F20110930%2FF112313.dat;mod_name=Carbamidomethyl%20%28C%29) |
| **Variable modifications:** | [Oxidation (M)](http://fun-gen1.ibls.gla.ac.uk/mascot/cgi/client.pl?modification;file=..%2Fdata%2F20110930%2FF112313.dat;mod_name=Oxidation%20%28M%29) |

**Protein sequence coverage: 3%**

Matched peptides shown in ***bold red***.

| **1** | MRLITLGFVF | ALWLCTLSFV | KGQVCFRKGV | SEIPEAK**NVD** | **ISNLEDTEID** |
| --- | --- | --- | --- | --- | --- |
| **51** | **K**TVR**LPCAIG** | **YVGFVR**LKCG | SKGWSKDGGR | KCEPKSCGHP | GDTPNGDFHL |
| **101** | SILDDFVFGA | QVLYQCRKGY | QMVTRSRHRT | CVEQGWDSAL | PICEALKCPV |
| **151** | IQANDNVVVI | GNSEDATYGN | VIQFECQSNH | MVLNGSSEMV | CNDKGEWSST |
| **201** | VPTCKVIKCY | APDIANGAVS | GPPKEDYDED | DTLRYSCNTK | YVKSQERVPK |
| **251** | CTKMVNSANW | SPTPACEEVR | CKLSLPPTRG | TSYRPADRNL | FLPDERVTVT |
| **301** | CASGFWNSIS | RQTENTMTCK | EDGKWSPSTT | DCERITCGDP | RDPLVSSPYY |
| **351** | WQRGQFRGTQ | RYNCRTGFKT | TNPRGVATCT | SDGSWTPKPL | CEEITCDKPD |
| **401** | ILNAVIKDPK | TRYKINDLLT | YECKMNYELL | DSTTRPTATC | TTNGWTKTLG |
| **451** | CKEIEGACIK | PNVMNGFIVQ | SNERNVDPRN | SKIYSSCNEG | FKPSTGGWWG |
| **501** | EATCTEGTWS | GILECIDQSQ | CGRIPVIPNT | IKVPHSEVYD | NEQTVTIDCK |
| **551** | VGYTSETKTI | KCKDGEWQTP | LPACRLQGVP | CDPPPKVENA | IVKIPYQNKY |
| **601** | REGFEVNYEC | RKSFRIEGHK | KLTCENGSWT | TPPPTCKQYC | GKPEGAGKQI |
| **651** | QILDQELERY | ENGNQIDYTC | INPYKGPGGT | ATCNNGEWHM | PIECKASCPD |
| **701** | PPPITNGDFT | KEKRDVEGVI | TEVSYQCSRQ | FTLSFTGNIR | CLDGKWQSPP |
| **751** | KCLRPCEIST | FDAEYNLQNL | PEKDNIAHGE | KKTLHCKEGY | YHQYKRFRPN |
| **801** | IEEIEMKCDD | GELQYGEHMP | ICRYRYS |  |  |

**SPOT # 627**

**Protein View: Q70I40_ONCMY**

**Triosephosphate isomerase (Fragment).- Oncorhynchus mykiss (Rainbow trout) (Salmo gairdneri).**

Top of Form

| **Database:** | MSDB |
| --- | --- |
| **Score:** | 131 |
| **Nominal mass (M_r_):** | 22759 |
| **Calculated pI:** | 6.59 |
| **Taxonomy:** | [Oncorhynchus mykiss](http://www.ncbi.nlm.nih.gov/Taxonomy/Browser/wwwtax.cgi?lvl=0&id=8022) |

This protein sequence matches the following other entries:

- CAE45565 from [Oncorhynchus mykiss](http://www.ncbi.nlm.nih.gov/Taxonomy/Browser/wwwtax.cgi?lvl=0&id=8022)

Sequence similarity is available as [an NCBI BLAST search of Q70I40_ONCMY against nr](http://www.ncbi.nlm.nih.gov/blast/Blast.cgi?ALIGNMENTS=50;ALIGNMENT_VIEW=Pairwise;AUTO_FORMAT=Semiauto;CDD_SEARCH=on;CLIENT=web;COMPOSITION_BASED_STATISTICS=on;DATABASE=nr;DESCRIPTIONS=100;ENTREZ_QUERY=%28none%29;EXPECT=10;FILTER=L;FORMAT_BLOCK_ON_RESPAGE=None;FORMAT_OBJECT=Alignment;FORMAT_TYPE=HTML;GAPCOSTS=11%201;I_THRESH=0.001;LAYOUT=TwoWindows;MATRIX_NAME=BLOSUM62;NCBI_GI=on;PAGE=Proteins;PROGRAM=blastp;QUERY=GDKASLGELIKTLNSAKLDPNTEVVCGAPSIYLEFARAKLDPKIGVAAQNCYKVKGGAFTGEISPAMIKDVGVHWVILGHSERRWVFGETDELIGQKCAHALENGLGVIACIGEKLDEREAGITEKVINAQTKHFADNIKDWSKVVLAYEPVWAIGTGKTASPAQAQDVHDKLRQWVKANVSEAVANSVRIIYGGSVTGGTCKELGGMKD;SERVICE=plain;SET_DEFAULTS.x=9;SET_DEFAULTS.y=5;SHOW_OVERVIEW=on;WORD_SIZE=3;END_OF_HTTPGET=Yes).

**Search parameters**

| **MS data file:** | H:\MBA1-G6\61_BF1_01_1693.mgf |
| --- | --- |
| **Enzyme:** | Trypsin: cuts C-term side of KR unless next residue is P. |
| **Fixed modifications:** | [Carbamidomethyl (C)](http://fun-gen1.ibls.gla.ac.uk/mascot/cgi/client.pl?modification;file=..%2Fdata%2F20110930%2FF112314.dat;mod_name=Carbamidomethyl%20%28C%29) |
| **Variable modifications:** | [Oxidation (M)](http://fun-gen1.ibls.gla.ac.uk/mascot/cgi/client.pl?modification;file=..%2Fdata%2F20110930%2FF112314.dat;mod_name=Oxidation%20%28M%29) |

**Protein sequence coverage: 22%**

Matched peptides shown in ***bold red***.

| **1** | GDK**ASLGELI** | **K**TLNSAKLDP | NTEVVCGAPS | IYLEFARAKL | DPKIGVAAQN |
| --- | --- | --- | --- | --- | --- |
| **51** | CYKVK**GGAFT** | **GEISPAMIK**D | VGVHWVILGH | SERRWVFGET | DELIGQKCAH |
| **101** | ALENGLGVIA | CIGEKLDERE | AGITEKVINA | QTKHFADNIK | DWSKVVLAYE |
| **151** | PVWAIGTGKT | ASPAQAQDVH | DKLRQWVK**AN** | **VSEAVANSVR** | **IIYGGSVTGG** |
| **201** | **TCK**ELGGMKD |  |  |  |  |

**Protein View: AAN40739**

**Immunoglobulin light chain constant region (Fragment).- Salmo salar (Atlantic salmon).**

Top of Form

| **Database:** | MSDB |
| --- | --- |
| **Score:** | 87 |
| **Nominal mass (M_r_):** | 10117 |
| **Calculated pI:** | 5.73 |
| **Taxonomy:** | [Salmo salar](http://www.ncbi.nlm.nih.gov/Taxonomy/Browser/wwwtax.cgi?lvl=0&id=8030) |

Sequence similarity is available as [an NCBI BLAST search of AAN40739 against nr](http://www.ncbi.nlm.nih.gov/blast/Blast.cgi?ALIGNMENTS=50;ALIGNMENT_VIEW=Pairwise;AUTO_FORMAT=Semiauto;CDD_SEARCH=on;CLIENT=web;COMPOSITION_BASED_STATISTICS=on;DATABASE=nr;DESCRIPTIONS=100;ENTREZ_QUERY=%28none%29;EXPECT=10;FILTER=L;FORMAT_BLOCK_ON_RESPAGE=None;FORMAT_OBJECT=Alignment;FORMAT_TYPE=HTML;GAPCOSTS=11%201;I_THRESH=0.001;LAYOUT=TwoWindows;MATRIX_NAME=BLOSUM62;NCBI_GI=on;PAGE=Proteins;PROGRAM=blastp;QUERY=QQGKATLMCLANKGFPSDWKLGWKVDGSSSSSSTWEVTGSPGVLEKDGHYSWSSTLTLPVDQWKKVGSVVCEATQGSQSPLSETLRRDQCSD;SERVICE=plain;SET_DEFAULTS.x=9;SET_DEFAULTS.y=5;SHOW_OVERVIEW=on;WORD_SIZE=3;END_OF_HTTPGET=Yes).

**Search parameters**

| **MS data file:** | H:\MBA1-G6\61_BF1_01_1693.mgf |
| --- | --- |
| **Enzyme:** | Trypsin: cuts C-term side of KR unless next residue is P. |
| **Fixed modifications:** | [Carbamidomethyl (C)](http://fun-gen1.ibls.gla.ac.uk/mascot/cgi/client.pl?modification;file=..%2Fdata%2F20110930%2FF112314.dat;mod_name=Carbamidomethyl%20%28C%29) |
| **Variable modifications:** | [Oxidation (M)](http://fun-gen1.ibls.gla.ac.uk/mascot/cgi/client.pl?modification;file=..%2Fdata%2F20110930%2FF112314.dat;mod_name=Oxidation%20%28M%29) |

**Protein sequence coverage: 25%**

Matched peptides shown in ***bold red***.

| **1** | QQGKATLMCL | ANKGFPSDWK | LGWKVDGSSS | SSSTWEVTGS | PGVLEKDGHY |
| --- | --- | --- | --- | --- | --- |
| **51** | SWSSTLTLPV | DQWK**KVGSVV** | **CEATQGSQSP** | **LSETLRR**DQC | SD |

**SPOT # 43**

**Protein View: TRF2_SALSA**

**Serotransferrin II precursor (Siderophilin II) (STF II).- Salmo salar (Atlantic salmon).**

Top of Form

| **Database:** | MSDB |
| --- | --- |
| **Score:** | 527 |
| **Nominal mass (M_r_):** | 76497 |
| **Calculated pI:** | 7.08 |
| **Taxonomy:** | [Salmo salar](http://www.ncbi.nlm.nih.gov/Taxonomy/Browser/wwwtax.cgi?lvl=0&id=8030) |

Sequence similarity is available as [an NCBI BLAST search of TRF2_SALSA against nr](http://www.ncbi.nlm.nih.gov/blast/Blast.cgi?ALIGNMENTS=50;ALIGNMENT_VIEW=Pairwise;AUTO_FORMAT=Semiauto;CDD_SEARCH=on;CLIENT=web;COMPOSITION_BASED_STATISTICS=on;DATABASE=nr;DESCRIPTIONS=100;ENTREZ_QUERY=%28none%29;EXPECT=10;FILTER=L;FORMAT_BLOCK_ON_RESPAGE=None;FORMAT_OBJECT=Alignment;FORMAT_TYPE=HTML;GAPCOSTS=11%201;I_THRESH=0.001;LAYOUT=TwoWindows;MATRIX_NAME=BLOSUM62;NCBI_GI=on;PAGE=Proteins;PROGRAM=blastp;QUERY=MKLLLLSALLGCLATAYAAPAEGIVKWCVKSEQELRKCHDLAAKVAEFSCVRKDGSFECIQAIKGGEADAITLDGGDIYTAGLTNYGLQPIIAEDYGEDSDTCYYAVAVAKKGTAFGFKTLRGKKSCHTGLGKSAGWNIPIGTLVTESQIRWAGIEDRPVESAVSDFFNASCAPGATMGSKLCQLCKGDCSRSHKEPYYDYAGAFQCLKDGAGDVAFIKPLAVPAAEKASYELLCKDGTRASIDSYKTCHLARVPAHAVVSRKDPELANRIYNKLVAVKDFNLFSSDGYAAKNLMFKDSAQKLVQLPTTTDSFLYLGAEYMSTIRSLKKSQATGASSRAIKWCAVGHAEKGKCDTWTINSFADGESKISCQDAPTVEECIKKIMRKEADAIAVDGGEVYTAGKCGLVPVMVEQYDADLCSAPGEASSYYAVAVAKKGSGLTWKTLKGKRSCHTGLGRTAGWNIPMGLIHQETNDCDFTKYFSKGCAPGSEVGSPFCAQCKGSGKARGGDEDRCKARSEEQYYGYTGAFRCLVEDAGDVAFIKHTIVPESTDGNGPDWAKDLKSSDFELLCQDGTTQPVTKFSECHLAKVPAHAVITRPETRGDVVSILLELQAKFGSSGSDSSFRMFQSSVEKNLLFKDSTKCLQEIPKGTKYQDFLGKEYMIAMQSLRKCSDSTSDLEKACTFHSCQQKE;SERVICE=plain;SET_DEFAULTS.x=9;SET_DEFAULTS.y=5;SHOW_OVERVIEW=on;WORD_SIZE=3;END_OF_HTTPGET=Yes).

**Search parameters**

| **MS data file:** | H:\MBA1-G6\62_BF2_01_1694.mgf |
| --- | --- |
| **Enzyme:** | Trypsin: cuts C-term side of KR unless next residue is P. |
| **Fixed modifications:** | [Carbamidomethyl (C)](http://fun-gen1.ibls.gla.ac.uk/mascot/cgi/client.pl?modification;file=..%2Fdata%2F20110930%2FF112315.dat;mod_name=Carbamidomethyl%20%28C%29) |
| **Variable modifications:** | [Oxidation (M)](http://fun-gen1.ibls.gla.ac.uk/mascot/cgi/client.pl?modification;file=..%2Fdata%2F20110930%2FF112315.dat;mod_name=Oxidation%20%28M%29) |

**Protein sequence coverage: 19%**

Matched peptides shown in ***bold red***.

| **1** | MKLLLLSALL | GCLATAYAAP | AEGIVKWCVK | SEQELRKCHD | LAAKVAEFSC |
| --- | --- | --- | --- | --- | --- |
| **51** | VRKDGSFECI | QAIKGGEADA | ITLDGGDIYT | AGLTNYGLQP | IIAEDYGEDS |
| **101** | DTCYYAVAVA | KKGTAFGFKT | LRGKKSCHTG | LGKSAGWNIP | IGTLVTESQI |
| **151** | RWAGIEDRPV | ESAVSDFFNA | SCAPGATMGS | KLCQLCKGDC | SR**SHKEPYYD** |
| **201** | **YAGAFQCLKD** | **GAGDVAFIKP** | **LAVPAAEK**AS | YELLCKDGTR | ASIDSYKTCH |
| **251** | LARVPAHAVV | SRKDPELANR | IYNKLVAVK**D** | **FNLFSSDGYA** | **AK**NLMFKDSA |
| **301** | QKLVQLPTTT | DSFLYLGAEY | MSTIRSLKKS | QATGASSRAI | KWCAVGHAEK |
| **351** | GKCDTWTINS | FADGESKISC | QDAPTVEECI | KKIMR**KEADA** | **IAVDGGEVYT** |
| **401** | **AGK**CGLVPVM | VEQYDADLCS | APGEASSYYA | VAVAKKGSGL | TWKTLKGKRS |
| **451** | CHTGLGRTAG | WNIPMGLIHQ | ETNDCDFTKY | FSKGCAPGSE | VGSPFCAQCK |
| **501** | GSGKARGGDE | DRCKAR**SEEQ** | **YYGYTGAFRC** | **LVEDAGDVAF** | **IK**HTIVPEST |
| **551** | DGNGPDWAKD | LK**SSDFELLC** | **QDGTTQPVTK** | FSECHLAKVP | AHAVITRPET |
| **601** | R**GDVVSILLE** | **LQAK**FGSSGS | DSSFRMFQSS | VEKNLLFKDS | TKCLQEIPKG |
| **651** | TKYQDFLGK**E** | **YMIAMQSLR**K | CSDSTSDLEK | ACTFHSCQQK | E |

**SPOT # 224**

**Protein View: P79825_ONCMY**

**Hemopexin-like protein (Fragment).- Oncorhynchus mykiss (Rainbow trout) (Salmo gairdneri).**

Top of Form

| **Database:** | MSDB |
| --- | --- |
| **Score:** | 150 |
| **Nominal mass (M_r_):** | 51106 |
| **Calculated pI:** | 5.61 |
| **Taxonomy:** | [Oncorhynchus mykiss](http://www.ncbi.nlm.nih.gov/Taxonomy/Browser/wwwtax.cgi?lvl=0&id=8022) |

This protein sequence matches the following other entries:

- CAA92147 from [Oncorhynchus mykiss](http://www.ncbi.nlm.nih.gov/Taxonomy/Browser/wwwtax.cgi?lvl=0&id=8022)

Sequence similarity is available as [an NCBI BLAST search of P79825_ONCMY against nr](http://www.ncbi.nlm.nih.gov/blast/Blast.cgi?ALIGNMENTS=50;ALIGNMENT_VIEW=Pairwise;AUTO_FORMAT=Semiauto;CDD_SEARCH=on;CLIENT=web;COMPOSITION_BASED_STATISTICS=on;DATABASE=nr;DESCRIPTIONS=100;ENTREZ_QUERY=%28none%29;EXPECT=10;FILTER=L;FORMAT_BLOCK_ON_RESPAGE=None;FORMAT_OBJECT=Alignment;FORMAT_TYPE=HTML;GAPCOSTS=11%201;I_THRESH=0.001;LAYOUT=TwoWindows;MATRIX_NAME=BLOSUM62;NCBI_GI=on;PAGE=Proteins;PROGRAM=blastp;QUERY=TMKPLSQTLCLCLVLALSHAHHHAGHQGGEDEGHEGHDHGHHEGLLLDRCQGIEMDAVAVTEEGIPYFFKGGHVFKGFHGKAELSNESFAELDDHHHLGHVDAAFLMHFPDKPTEHDHIFFMLDTKVFSYYKHQLETGFPKDISEVFPGIPDHLDAAVVCPAPDCEEDAVIFFKGDEIYHYNVKTKKVEEKKFEGMPNCTSAFRFMEHYYCFHGHQFSKFDPKTGEVHGRYPKEARDYFMKCSKFGDTTDHIERERCSRVHLDAITSDDAGNIYAFRGHHFLEQDAGNDTWAADTIESDFKELHSEVDATFSYENHLYMVKDDKVYIYKVGDSHTHLDGSPKPLKEVLGVEGPIDAAFVCQDHHIAHVIKGQTVYDVDLKASPPVPVKEGSFTLFNKVDAAMCGPEGVKLFKGNHYFHFQSVKVMLMAKAIPEEHKTALELFGCDH;SERVICE=plain;SET_DEFAULTS.x=9;SET_DEFAULTS.y=5;SHOW_OVERVIEW=on;WORD_SIZE=3;END_OF_HTTPGET=Yes).

**Search parameters**

| **MS data file:** | H:\MBA1-G6\63_BF3_01_1695.mgf |
| --- | --- |
| **Enzyme:** | Trypsin: cuts C-term side of KR unless next residue is P. |
| **Fixed modifications:** | [Carbamidomethyl (C)](http://fun-gen1.ibls.gla.ac.uk/mascot/cgi/client.pl?modification;file=..%2Fdata%2F20110930%2FF112316.dat;mod_name=Carbamidomethyl%20%28C%29) |
| **Variable modifications:** | [Oxidation (M)](http://fun-gen1.ibls.gla.ac.uk/mascot/cgi/client.pl?modification;file=..%2Fdata%2F20110930%2FF112316.dat;mod_name=Oxidation%20%28M%29) |

**Protein sequence coverage: 16%**

Matched peptides shown in ***bold red***.

| **1** | TMKPLSQTLC | LCLVLALSHA | HHHAGHQGGE | DEGHEGHDHG | HHEGLLLDRC |
| --- | --- | --- | --- | --- | --- |
| **51** | QGIEMDAVAV | TEEGIPYFFK | GGHVFKGFHG | KAELSNESFA | ELDDHHHLGH |
| **101** | VDAAFLMHFP | DKPTEHDHIF | FMLDTKVFSY | YKHQLETGFP | KDISEVFPGI |
| **151** | PDHLDAAVVC | PAPDCEEDAV | IFFK**GDEIYH** | **YNVK**TKKVEE | KKFEGMPNCT |
| **201** | SAFR**FMEHYY** | **CFHGHQFSK**F | DPKTGEVHGR | YPKEARDYFM | KCSK**FGDTTD** |
| **251** | **HIER**ERCSR**V** | **HLDAITSDDA** | **GNIYAFR**GHH | FLEQDAGNDT | WAADTIESDF |
| **301** | KELHSEVDAT | FSYENHLYMV | KDDKVYIYKV | GDSHTHLDGS | PKPLKEVLGV |
| **351** | EGPIDAAFVC | QDHHIAHVIK | **GQTVYDVDLK** | ASPPVPVKEG | SFTLFNKVDA |
| **401** | AMCGPEGVKL | FKGNHYFHFQ | SVKVMLMAKA | IPEEHK**TALE** | **LFGCDH** |

**SPOT # 220**

**Protein View: P79825_ONCMY**

**Hemopexin-like protein (Fragment).- Oncorhynchus mykiss (Rainbow trout) (Salmo gairdneri).**

Top of Form

| **Database:** | MSDB |
| --- | --- |
| **Score:** | 166 |
| **Nominal mass (M_r_):** | 51106 |
| **Calculated pI:** | 5.61 |
| **Taxonomy:** | [Oncorhynchus mykiss](http://www.ncbi.nlm.nih.gov/Taxonomy/Browser/wwwtax.cgi?lvl=0&id=8022) |

This protein sequence matches the following other entries:

- CAA92147 from [Oncorhynchus mykiss](http://www.ncbi.nlm.nih.gov/Taxonomy/Browser/wwwtax.cgi?lvl=0&id=8022)

Sequence similarity is available as [an NCBI BLAST search of P79825_ONCMY against nr](http://www.ncbi.nlm.nih.gov/blast/Blast.cgi?ALIGNMENTS=50;ALIGNMENT_VIEW=Pairwise;AUTO_FORMAT=Semiauto;CDD_SEARCH=on;CLIENT=web;COMPOSITION_BASED_STATISTICS=on;DATABASE=nr;DESCRIPTIONS=100;ENTREZ_QUERY=%28none%29;EXPECT=10;FILTER=L;FORMAT_BLOCK_ON_RESPAGE=None;FORMAT_OBJECT=Alignment;FORMAT_TYPE=HTML;GAPCOSTS=11%201;I_THRESH=0.001;LAYOUT=TwoWindows;MATRIX_NAME=BLOSUM62;NCBI_GI=on;PAGE=Proteins;PROGRAM=blastp;QUERY=TMKPLSQTLCLCLVLALSHAHHHAGHQGGEDEGHEGHDHGHHEGLLLDRCQGIEMDAVAVTEEGIPYFFKGGHVFKGFHGKAELSNESFAELDDHHHLGHVDAAFLMHFPDKPTEHDHIFFMLDTKVFSYYKHQLETGFPKDISEVFPGIPDHLDAAVVCPAPDCEEDAVIFFKGDEIYHYNVKTKKVEEKKFEGMPNCTSAFRFMEHYYCFHGHQFSKFDPKTGEVHGRYPKEARDYFMKCSKFGDTTDHIERERCSRVHLDAITSDDAGNIYAFRGHHFLEQDAGNDTWAADTIESDFKELHSEVDATFSYENHLYMVKDDKVYIYKVGDSHTHLDGSPKPLKEVLGVEGPIDAAFVCQDHHIAHVIKGQTVYDVDLKASPPVPVKEGSFTLFNKVDAAMCGPEGVKLFKGNHYFHFQSVKVMLMAKAIPEEHKTALELFGCDH;SERVICE=plain;SET_DEFAULTS.x=9;SET_DEFAULTS.y=5;SHOW_OVERVIEW=on;WORD_SIZE=3;END_OF_HTTPGET=Yes).

**Search parameters**

| **MS data file:** | H:\MBA1-G6\64_BF4_01_1696.mgf |
| --- | --- |
| **Enzyme:** | Trypsin: cuts C-term side of KR unless next residue is P. |
| **Fixed modifications:** | [Carbamidomethyl (C)](http://fun-gen1.ibls.gla.ac.uk/mascot/cgi/client.pl?modification;file=..%2Fdata%2F20110930%2FF112317.dat;mod_name=Carbamidomethyl%20%28C%29) |
| **Variable modifications:** | [Oxidation (M)](http://fun-gen1.ibls.gla.ac.uk/mascot/cgi/client.pl?modification;file=..%2Fdata%2F20110930%2FF112317.dat;mod_name=Oxidation%20%28M%29) |

**Protein sequence coverage: 8%**

Matched peptides shown in ***bold red***.

| **1** | TMKPLSQTLC | LCLVLALSHA | HHHAGHQGGE | DEGHEGHDHG | HHEGLLLDRC |
| --- | --- | --- | --- | --- | --- |
| **51** | QGIEMDAVAV | TEEGIPYFFK | GGHVFKGFHG | KAELSNESFA | ELDDHHHLGH |
| **101** | VDAAFLMHFP | DKPTEHDHIF | FMLDTKVFSY | YKHQLETGFP | KDISEVFPGI |
| **151** | PDHLDAAVVC | PAPDCEEDAV | IFFK**GDEIYH** | **YNVK**TKKVEE | KKFEGMPNCT |
| **201** | SAFRFMEHYY | CFHGHQFSKF | DPKTGEVHGR | YPKEARDYFM | KCSKFGDTTD |
| **251** | HIERERCSR**V** | **HLDAITSDDA** | **GNIYAFR**GHH | FLEQDAGNDT | WAADTIESDF |
| **301** | KELHSEVDAT | FSYENHLYMV | KDDKVYIYKV | GDSHTHLDGS | PKPLKEVLGV |
| **351** | EGPIDAAFVC | QDHHIAHVIK | **GQTVYDVDLK** | ASPPVPVKEG | SFTLFNKVDA |
| **401** | AMCGPEGVKL | FKGNHYFHFQ | SVKVMLMAKA | IPEEHKTALE | LFGCDH |

**Protein View: Q4U1U5_9SMEG**

**Beta-actin (Fragment).- Pungitius pungitius.**

Top of Form

| **Database:** | MSDB |
| --- | --- |
| **Score:** | 107 |
| **Nominal mass (M_r_):** | 40495 |
| **Calculated pI:** | 5.29 |
| **Taxonomy:** | [Pungitius pungitius](http://www.ncbi.nlm.nih.gov/Taxonomy/Browser/wwwtax.cgi?lvl=0&id=134920) |

This protein sequence matches the following other entries:

- AAY52025 from [Pungitius pungitius](http://www.ncbi.nlm.nih.gov/Taxonomy/Browser/wwwtax.cgi?lvl=0&id=134920)

Sequence similarity is available as [an NCBI BLAST search of Q4U1U5_9SMEG against nr](http://www.ncbi.nlm.nih.gov/blast/Blast.cgi?ALIGNMENTS=50;ALIGNMENT_VIEW=Pairwise;AUTO_FORMAT=Semiauto;CDD_SEARCH=on;CLIENT=web;COMPOSITION_BASED_STATISTICS=on;DATABASE=nr;DESCRIPTIONS=100;ENTREZ_QUERY=%28none%29;EXPECT=10;FILTER=L;FORMAT_BLOCK_ON_RESPAGE=None;FORMAT_OBJECT=Alignment;FORMAT_TYPE=HTML;GAPCOSTS=11%201;I_THRESH=0.001;LAYOUT=TwoWindows;MATRIX_NAME=BLOSUM62;NCBI_GI=on;PAGE=Proteins;PROGRAM=blastp;QUERY=LVVDNGSGMCKAGFAGDDAPRAVFPSIVGRPRHQGVMVGMGQKDSYVGDEAQSKRGILTLKYPIEHGIVTNWDDMEKIWHHTFYNELRVAPEEHPVLLTEAPLNPKANREKMTQIMFETFNTPAMYVAIQAVLSLYASGRTTGIVMDSGDGVTHTVPIYEGYALPHAILRLDLAGRDLTDYLMKILTERGYSFTTTAEREIVRDIKEKLCYVALDFEQEMGTAASSSSLEKSYELPDGQVITIGNERFRCPEALFQPSFLGMESCGIHETTYNSIMKCDVDIRKDLYANTVLSGGTTMYPGIADRMQKEITALAPSTMKIKIIAPPERKYSVWIGGSILASLSTFQQMWISKQEYDESGPSI;SERVICE=plain;SET_DEFAULTS.x=9;SET_DEFAULTS.y=5;SHOW_OVERVIEW=on;WORD_SIZE=3;END_OF_HTTPGET=Yes).

**Search parameters**

| **MS data file:** | H:\MBA1-G6\64_BF4_01_1696.mgf |
| --- | --- |
| **Enzyme:** | Trypsin: cuts C-term side of KR unless next residue is P. |
| **Fixed modifications:** | [Carbamidomethyl (C)](http://fun-gen1.ibls.gla.ac.uk/mascot/cgi/client.pl?modification;file=..%2Fdata%2F20110930%2FF112317.dat;mod_name=Carbamidomethyl%20%28C%29) |
| **Variable modifications:** | [Oxidation (M)](http://fun-gen1.ibls.gla.ac.uk/mascot/cgi/client.pl?modification;file=..%2Fdata%2F20110930%2FF112317.dat;mod_name=Oxidation%20%28M%29) |

**Protein sequence coverage: 12%**

Matched peptides shown in ***bold red***.

| **1** | LVVDNGSGMC | KAGFAGDDAP | RAVFPSIVGR | PRHQGVMVGM | GQKDSYVGDE |
| --- | --- | --- | --- | --- | --- |
| **51** | AQSKRGILTL | KYPIEHGIVT | NWDDMEKIWH | HTFYNELR**VA** | **PEEHPVLLTE** |
| **101** | **APLNPK**ANRE | KMTQIMFETF | NTPAMYVAIQ | AVLSLYASGR | TTGIVMDSGD |
| **151** | GVTHTVPIYE | GYALPHAILR | LDLAGRDLTD | YLMKILTERG | YSFTTTAERE |
| **201** | IVRDIKEKLC | YVALDFEQEM | GTAASSSSLE | K**SYELPDGQV** | **ITIGNER**FRC |
| **251** | PEALFQPSFL | GMESCGIHET | TYNSIMKCDV | DIRKDLYANT | VLSGGTTMYP |
| **301** | GIADRMQK**EI** | **TALAPSTMK**I | KIIAPPERKY | SVWIGGSILA | SLSTFQQMWI |
| **351** | SKQEYDESGP | SI |  |  |  |

**SPOT # 702**

**Protein View: Q9DFD7_ONCMY**

**Prostaglandine D synthase (Fragment).- Oncorhynchus mykiss (Rainbow trout) (Salmo gairdneri).**

Top of Form

| **Database:** | MSDB |
| --- | --- |
| **Score:** | 82 |
| **Nominal mass (M_r_):** | 19237 |
| **Calculated pI:** | 9.51 |
| **Taxonomy:** | [Oncorhynchus mykiss](http://www.ncbi.nlm.nih.gov/Taxonomy/Browser/wwwtax.cgi?lvl=0&id=8022) |

This protein sequence matches the following other entries:

- AAG30028 from [Oncorhynchus mykiss](http://www.ncbi.nlm.nih.gov/Taxonomy/Browser/wwwtax.cgi?lvl=0&id=8022)

Sequence similarity is available as [an NCBI BLAST search of Q9DFD7_ONCMY against nr](http://www.ncbi.nlm.nih.gov/blast/Blast.cgi?ALIGNMENTS=50;ALIGNMENT_VIEW=Pairwise;AUTO_FORMAT=Semiauto;CDD_SEARCH=on;CLIENT=web;COMPOSITION_BASED_STATISTICS=on;DATABASE=nr;DESCRIPTIONS=100;ENTREZ_QUERY=%28none%29;EXPECT=10;FILTER=L;FORMAT_BLOCK_ON_RESPAGE=None;FORMAT_OBJECT=Alignment;FORMAT_TYPE=HTML;GAPCOSTS=11%201;I_THRESH=0.001;LAYOUT=TwoWindows;MATRIX_NAME=BLOSUM62;NCBI_GI=on;PAGE=Proteins;PROGRAM=blastp;QUERY=GVLLCATLVACVNVMPQKDFNLEKMAGKWWVVGFATNAKWFMNRKAGMKMGTSMMLPTAGGSLDISSAMRNSDGSCWRMTELAKKTDIPGRFTFISQRWNNENDMRVVAVQYDDFALIHTIKTKHGVTDVLNKLFSRTPEVSADVQKKFMQFSLDTGILSGNIVFLPKER;SERVICE=plain;SET_DEFAULTS.x=9;SET_DEFAULTS.y=5;SHOW_OVERVIEW=on;WORD_SIZE=3;END_OF_HTTPGET=Yes).

**Search parameters**

| **MS data file:** | H:\MBA1-G6\65_BF5_01_1697.mgf |
| --- | --- |
| **Enzyme:** | Trypsin: cuts C-term side of KR unless next residue is P. |
| **Fixed modifications:** | [Carbamidomethyl (C)](http://fun-gen1.ibls.gla.ac.uk/mascot/cgi/client.pl?modification;file=..%2Fdata%2F20110930%2FF112318.dat;mod_name=Carbamidomethyl%20%28C%29) |
| **Variable modifications:** | [Oxidation (M)](http://fun-gen1.ibls.gla.ac.uk/mascot/cgi/client.pl?modification;file=..%2Fdata%2F20110930%2FF112318.dat;mod_name=Oxidation%20%28M%29) |

**Protein sequence coverage: 9%**

Matched peptides shown in ***bold red***.

| **1** | GVLLCATLVA | CVNVMPQKDF | NLEKMAGKWW | VVGFATNAKW | FMNRKAGMKM |
| --- | --- | --- | --- | --- | --- |
| **51** | GTSMMLPTAG | GSLDISSAMR | NSDGSCWRMT | ELAKKTDIPG | RFTFISQRWN |
| **101** | NENDMR**VVAV** | **QYDDFALIHT** | **IK**TKHGVTDV | LNKLFSRTPE | VSADVQKKFM |
| **151** | QFSLDTGILS | GNIVFLPKER |  |  |  |

**SPOT # 19**

**Protein View: TRF2_SALSA**

**Serotransferrin II precursor (Siderophilin II) (STF II).- Salmo salar (Atlantic salmon).**

Top of Form

| **Database:** | MSDB |
| --- | --- |
| **Score:** | 821 |
| **Nominal mass (M_r_):** | 76497 |
| **Calculated pI:** | 7.08 |
| **Taxonomy:** | [Salmo salar](http://www.ncbi.nlm.nih.gov/Taxonomy/Browser/wwwtax.cgi?lvl=0&id=8030) |

Sequence similarity is available as [an NCBI BLAST search of TRF2_SALSA against nr](http://www.ncbi.nlm.nih.gov/blast/Blast.cgi?ALIGNMENTS=50;ALIGNMENT_VIEW=Pairwise;AUTO_FORMAT=Semiauto;CDD_SEARCH=on;CLIENT=web;COMPOSITION_BASED_STATISTICS=on;DATABASE=nr;DESCRIPTIONS=100;ENTREZ_QUERY=%28none%29;EXPECT=10;FILTER=L;FORMAT_BLOCK_ON_RESPAGE=None;FORMAT_OBJECT=Alignment;FORMAT_TYPE=HTML;GAPCOSTS=11%201;I_THRESH=0.001;LAYOUT=TwoWindows;MATRIX_NAME=BLOSUM62;NCBI_GI=on;PAGE=Proteins;PROGRAM=blastp;QUERY=MKLLLLSALLGCLATAYAAPAEGIVKWCVKSEQELRKCHDLAAKVAEFSCVRKDGSFECIQAIKGGEADAITLDGGDIYTAGLTNYGLQPIIAEDYGEDSDTCYYAVAVAKKGTAFGFKTLRGKKSCHTGLGKSAGWNIPIGTLVTESQIRWAGIEDRPVESAVSDFFNASCAPGATMGSKLCQLCKGDCSRSHKEPYYDYAGAFQCLKDGAGDVAFIKPLAVPAAEKASYELLCKDGTRASIDSYKTCHLARVPAHAVVSRKDPELANRIYNKLVAVKDFNLFSSDGYAAKNLMFKDSAQKLVQLPTTTDSFLYLGAEYMSTIRSLKKSQATGASSRAIKWCAVGHAEKGKCDTWTINSFADGESKISCQDAPTVEECIKKIMRKEADAIAVDGGEVYTAGKCGLVPVMVEQYDADLCSAPGEASSYYAVAVAKKGSGLTWKTLKGKRSCHTGLGRTAGWNIPMGLIHQETNDCDFTKYFSKGCAPGSEVGSPFCAQCKGSGKARGGDEDRCKARSEEQYYGYTGAFRCLVEDAGDVAFIKHTIVPESTDGNGPDWAKDLKSSDFELLCQDGTTQPVTKFSECHLAKVPAHAVITRPETRGDVVSILLELQAKFGSSGSDSSFRMFQSSVEKNLLFKDSTKCLQEIPKGTKYQDFLGKEYMIAMQSLRKCSDSTSDLEKACTFHSCQQKE;SERVICE=plain;SET_DEFAULTS.x=9;SET_DEFAULTS.y=5;SHOW_OVERVIEW=on;WORD_SIZE=3;END_OF_HTTPGET=Yes).

**Search parameters**

| **MS data file:** | H:\MBA1-G6\66_BF6_01_1698.mgf |
| --- | --- |
| **Enzyme:** | Trypsin: cuts C-term side of KR unless next residue is P. |
| **Fixed modifications:** | [Carbamidomethyl (C)](http://fun-gen1.ibls.gla.ac.uk/mascot/cgi/client.pl?modification;file=..%2Fdata%2F20110930%2FF112319.dat;mod_name=Carbamidomethyl%20%28C%29) |
| **Variable modifications:** | [Oxidation (M)](http://fun-gen1.ibls.gla.ac.uk/mascot/cgi/client.pl?modification;file=..%2Fdata%2F20110930%2FF112319.dat;mod_name=Oxidation%20%28M%29) |

**Protein sequence coverage: 38%**

Matched peptides shown in ***bold red***.

| **1** | MKLLLLSALL | GCLATAYAAP | AEGIVKWCVK | SEQELRKCHD | LAAKVAEFSC |
| --- | --- | --- | --- | --- | --- |
| **51** | VR**KDGSFECI** | **QAIK**GGEADA | ITLDGGDIYT | AGLTNYGLQP | IIAEDYGEDS |
| **101** | DTCYYAVAVA | K**KGTAFGFK**T | LRGKKSCHTG | LGKSAGWNIP | IGTLVTESQI |
| **151** | RWAGIEDRPV | ESAVSDFFNA | SCAPGATMGS | KLCQLCKGDC | SR**SHKEPYYD** |
| **201** | **YAGAFQCLKD** | **GAGDVAFIKP** | **LAVPAAEK**AS | YELLCKDGTR | ASIDSYKTCH |
| **251** | LARVPAHAVV | SRKDPELANR | IYNKLVAVK**D** | **FNLFSSDGYA** | **AK**NLMFKDSA |
| **301** | QK**LVQLPTTT** | **DSFLYLGAEY** | **MSTIR**SLKKS | QATGASSRAI | KWCAVGHAEK |
| **351** | **GKCDTWTINS** | **FADGESK**ISC | QDAPTVEECI | KKIMR**KEADA** | **IAVDGGEVYT** |
| **401** | **AGK**CGLVPVM | VEQYDADLCS | APGEASSYYA | VAVAKKGSGL | TWKTLKGKRS |
| **451** | CHTGLGR**TAG** | **WNIPMGLIHQ** | **ETNDCDFTK**Y | FSK**GCAPGSE** | **VGSPFCAQCK** |
| **501** | GSGKARGGDE | DRCKAR**SEEQ** | **YYGYTGAFRC** | **LVEDAGDVAF** | **IKHTIVPEST** |
| **551** | **DGNGPDWAK**D | LK**SSDFELLC** | **QDGTTQPVTK** | FSECHLAK**VP** | **AHAVITRPET** |
| **601** | **R**GDVVSILLE | LQAKFGSSGS | DSSFRMFQSS | VEKNLLFKDS | TK**CLQEIPK**G |
| **651** | TKYQDFLGK**E** | **YMIAMQSLR**K | CSDSTSDLEK | **ACTFHSCQQK** | **E** |

**Protein View: A46533**

**Ig heavy chain C region - Atlantic salmon (fragment)**

Top of Form

| **Database:** | MSDB |
| --- | --- |
| **Score:** | 65 |
| **Nominal mass (M_r_):** | 50680 |
| **Calculated pI:** | 5.79 |
| **Taxonomy:** | [Salmo salar](http://www.ncbi.nlm.nih.gov/Taxonomy/Browser/wwwtax.cgi?lvl=0&id=8030) |

This protein sequence matches the following other entries:

- AAB24064 from [Salmo salar](http://www.ncbi.nlm.nih.gov/Taxonomy/Browser/wwwtax.cgi?lvl=0&id=8030)

Sequence similarity is available as [an NCBI BLAST search of A46533 against nr](http://www.ncbi.nlm.nih.gov/blast/Blast.cgi?ALIGNMENTS=50;ALIGNMENT_VIEW=Pairwise;AUTO_FORMAT=Semiauto;CDD_SEARCH=on;CLIENT=web;COMPOSITION_BASED_STATISTICS=on;DATABASE=nr;DESCRIPTIONS=100;ENTREZ_QUERY=%28none%29;EXPECT=10;FILTER=L;FORMAT_BLOCK_ON_RESPAGE=None;FORMAT_OBJECT=Alignment;FORMAT_TYPE=HTML;GAPCOSTS=11%201;I_THRESH=0.001;LAYOUT=TwoWindows;MATRIX_NAME=BLOSUM62;NCBI_GI=on;PAGE=Proteins;PROGRAM=blastp;QUERY=ASSTAPTLFPLAQCGSGTGDMMTLGCIATGFTPASLTFKWNEQGGNSLTDFVQYPAVQTSGSYMGVSQLRVKRADWDSKIFECAVEHSAGSKTVPVKKQAEYLQHPSLYVMTPSKEEMAENMTASFACFANDFSPRTHTIKWMRMEQGIEKEVVSDFKSSCESEKKSDKTLYSTTSYLRVNESEWKSEEVAFTCVFENKAGNVRRTVGYTSSDAGPVHAHSVVIKITPPSLEDMLMNKKAELVCDVEELVPGFMSVKWENDNGKTLTSRKGVTDRIAILDITYEDWSNGTVFYCAVDHLENLGSLVKKPYKRETGGDPQRPSVFLLAPAEKTSDNTVTLTCYVKDFYPKEVLVAWLIDDEPVERTSSSALYQFNTTSQIQTGRTYSVYSQLTFSNDLWKNKEVVYSCVVYHESMIKSTKILMRTIDRTSNQPYLVNLSLNVPQSCKAQ;SERVICE=plain;SET_DEFAULTS.x=9;SET_DEFAULTS.y=5;SHOW_OVERVIEW=on;WORD_SIZE=3;END_OF_HTTPGET=Yes).

**Search parameters**

| **MS data file:** | H:\MBA1-G6\66_BF6_01_1698.mgf |
| --- | --- |
| **Enzyme:** | Trypsin: cuts C-term side of KR unless next residue is P. |
| **Fixed modifications:** | [Carbamidomethyl (C)](http://fun-gen1.ibls.gla.ac.uk/mascot/cgi/client.pl?modification;file=..%2Fdata%2F20110930%2FF112319.dat;mod_name=Carbamidomethyl%20%28C%29) |
| **Variable modifications:** | [Oxidation (M)](http://fun-gen1.ibls.gla.ac.uk/mascot/cgi/client.pl?modification;file=..%2Fdata%2F20110930%2FF112319.dat;mod_name=Oxidation%20%28M%29) |

**Protein sequence coverage: 5%**

Matched peptides shown in ***bold red***.

| **1** | ASSTAPTLFP | LAQCGSGTGD | MMTLGCIATG | FTPASLTFKW | NEQGGNSLTD |
| --- | --- | --- | --- | --- | --- |
| **51** | FVQYPAVQTS | GSYMGVSQLR | VKRADWDSK**I** | **FECAVEHSAG** | **SK**TVPVKKQA |
| **101** | EYLQHPSLYV | MTPSKEEMAE | NMTASFACFA | NDFSPRTHTI | KWMRMEQGIE |
| **151** | KEVVSDFKSS | CESEKKSDKT | LYSTTSYLRV | NESEWKSEEV | AFTCVFENKA |
| **201** | GNVRRTVGYT | SSDAGPVHAH | SVVIKITPPS | LEDMLMNKKA | ELVCDVEELV |
| **251** | PGFMSVKWEN | DNGKTLTSRK | GVTDRIAILD | ITYEDWSNGT | VFYCAVDHLE |
| **301** | NLGSLVKKPY | KRETGGDPQR | PSVFLLAPAE | K**TSDNTVTLT** | **CYVK**DFYPKE |
| **351** | VLVAWLIDDE | PVERTSSSAL | YQFNTTSQIQ | TGRTYSVYSQ | LTFSNDLWKN |
| **401** | KEVVYSCVVY | HESMIKSTKI | LMRTIDRTSN | QPYLVNLSLN | VPQSCKAQ |

**SPOT # 151**

**Protein View: T11749**

**transferrin - Atlantic salmon**

Top of Form

| **Database:** | MSDB |
| --- | --- |
| **Score:** | 967 |
| **Nominal mass (M_r_):** | 76431 |
| **Calculated pI:** | 7.08 |
| **Taxonomy:** | [Salmo salar](http://www.ncbi.nlm.nih.gov/Taxonomy/Browser/wwwtax.cgi?lvl=0&id=8030) |

This protein sequence matches the following other entries:

- AAA18838 from [Salmo salar](http://www.ncbi.nlm.nih.gov/Taxonomy/Browser/wwwtax.cgi?lvl=0&id=8030)
- TRF1_SALSA from [Salmo salar](http://www.ncbi.nlm.nih.gov/Taxonomy/Browser/wwwtax.cgi?lvl=0&id=8030)

Sequence similarity is available as [an NCBI BLAST search of T11749 against nr](http://www.ncbi.nlm.nih.gov/blast/Blast.cgi?ALIGNMENTS=50;ALIGNMENT_VIEW=Pairwise;AUTO_FORMAT=Semiauto;CDD_SEARCH=on;CLIENT=web;COMPOSITION_BASED_STATISTICS=on;DATABASE=nr;DESCRIPTIONS=100;ENTREZ_QUERY=%28none%29;EXPECT=10;FILTER=L;FORMAT_BLOCK_ON_RESPAGE=None;FORMAT_OBJECT=Alignment;FORMAT_TYPE=HTML;GAPCOSTS=11%201;I_THRESH=0.001;LAYOUT=TwoWindows;MATRIX_NAME=BLOSUM62;NCBI_GI=on;PAGE=Proteins;PROGRAM=blastp;QUERY=MKLLLLSALLGCLATAYAAPAEGIVKWCVKSEQELRKCHDLAAKVAEFSCVRKDGSFECIQAIKGGEADAITLDGGDIYTAGLTNYGLQPIIAEDYGEDSDTCYYAVAVAKKGTAFGFKTLRGKKSCHTGLGKSAGWNIPIGTLVTESQIRWAGIEDRPVESAVSDFFNASCAPGATMGSKLCQLCKGDCSRSHKEPYYDYAGAFQCLKDGAGDVAFIKPLAVPAAEKASYELLCKDGTRASIDSYKTCHLARVPAHAVVSRKDPELANRIYNKLVAVKDFNLFSSDGYAAKNLMFKDSAQKLVQLPTTTDSFLYLGAEYMSTIRSLKKSQATGASSRAIKWCAVGHAEKGKCDTWTINSFADGESKISCQDAPTVEECIKKIMRKEADAIAVDGGEVYTAGKCGLVPVMVEQYDADLCSAPGEASSYYAVAVAKKGSGLTWKTLKGKRSCHTGLGRTAGWNIPMGLIHQETNDCDFTKYFSKGCAPGSEVGSPFCAQCKGSGKAVGDEYRCKARSEEQYYGYTGAFRCLVEDAGDVAFIKHTIVPESTDGNGPDWAKDLKSSDFELLCQDGTTQPVTKFSECHLAKVPAHAVITRPETRGDVVSILLELQAKFGSSGSDSSFRMFQSSVEKNLLFKDSTKCLQEIPKGTKYQDFLGKEYMIAMQSLRKCSDSTSDLEKACTFHSCQQKE;SERVICE=plain;SET_DEFAULTS.x=9;SET_DEFAULTS.y=5;SHOW_OVERVIEW=on;WORD_SIZE=3;END_OF_HTTPGET=Yes).

**Search parameters**

| **MS data file:** | H:\MBA1-G6\67_BF7_01_1699.mgf |
| --- | --- |
| **Enzyme:** | Trypsin: cuts C-term side of KR unless next residue is P. |
| **Fixed modifications:** | [Carbamidomethyl (C)](http://fun-gen1.ibls.gla.ac.uk/mascot/cgi/client.pl?modification;file=..%2Fdata%2F20110930%2FF112320.dat;mod_name=Carbamidomethyl%20%28C%29) |
| **Variable modifications:** | [Oxidation (M)](http://fun-gen1.ibls.gla.ac.uk/mascot/cgi/client.pl?modification;file=..%2Fdata%2F20110930%2FF112320.dat;mod_name=Oxidation%20%28M%29) |

**Protein sequence coverage: 35%**

Matched peptides shown in ***bold red***.

| **1** | MKLLLLSALL | GCLATAYAAP | AEGIVKWCVK | SEQELRKCHD | LAAKVAEFSC |
| --- | --- | --- | --- | --- | --- |
| **51** | VR**KDGSFECI** | **QAIK**GGEADA | ITLDGGDIYT | AGLTNYGLQP | IIAEDYGEDS |
| **101** | DTCYYAVAVA | K**KGTAFGFK**T | LRGKKSCHTG | LGKSAGWNIP | IGTLVTESQI |
| **151** | RWAGIEDRPV | ESAVSDFFNA | SCAPGATMGS | KLCQLCKGDC | SR**SHKEPYYD** |
| **201** | **YAGAFQCLKD** | **GAGDVAFIKP** | **LAVPAAEK**AS | YELLCKDGTR | ASIDSYKTCH |
| **251** | LARVPAHAVV | SRKDPELANR | IYNKLVAVK**D** | **FNLFSSDGYA** | **AK**NLMFKDSA |
| **301** | QK**LVQLPTTT** | **DSFLYLGAEY** | **MSTIR**SLKKS | QATGASSRAI | KWCAVGHAEK |
| **351** | GKCDTWTINS | FADGESKISC | QDAPTVEECI | KKIMR**KEADA** | **IAVDGGEVYT** |
| **401** | **AGK**CGLVPVM | VEQYDADLCS | APGEASSYYA | VAVAKKGSGL | TWKTLKGKRS |
| **451** | CHTGLGRTAG | WNIPMGLIHQ | ETNDCDFTKY | FSK**GCAPGSE** | **VGSPFCAQCK** |
| **501** | GSGK**AVGDEY** | **R**CK**ARSEEQY** | **YGYTGAFRCL** | **VEDAGDVAFI** | **K**HTIVPESTD |
| **551** | GNGPDWAKDL | K**SSDFELLCQ** | **DGTTQPVTK**F | SECHLAK**VPA** | **HAVITRPETR** |
| **601** | **GDVVSILLEL** | **QAKFGSSGSD** | **SSFR**MFQSSV | EKNLLFKDST | K**CLQEIPK**GT |
| **651** | KYQDFLGK**EY** | **MIAMQSLR**KC | SDSTSDLEK**A** | **CTFHSCQQKE** |  |

**Protein View: A46533**

**Ig heavy chain C region - Atlantic salmon (fragment)**

Top of Form

| **Database:** | MSDB |
| --- | --- |
| **Score:** | 139 |
| **Nominal mass (M_r_):** | 50680 |
| **Calculated pI:** | 5.79 |
| **Taxonomy:** | [Salmo salar](http://www.ncbi.nlm.nih.gov/Taxonomy/Browser/wwwtax.cgi?lvl=0&id=8030) |

This protein sequence matches the following other entries:

- AAB24064 from [Salmo salar](http://www.ncbi.nlm.nih.gov/Taxonomy/Browser/wwwtax.cgi?lvl=0&id=8030)

Sequence similarity is available as [an NCBI BLAST search of A46533 against nr](http://www.ncbi.nlm.nih.gov/blast/Blast.cgi?ALIGNMENTS=50;ALIGNMENT_VIEW=Pairwise;AUTO_FORMAT=Semiauto;CDD_SEARCH=on;CLIENT=web;COMPOSITION_BASED_STATISTICS=on;DATABASE=nr;DESCRIPTIONS=100;ENTREZ_QUERY=%28none%29;EXPECT=10;FILTER=L;FORMAT_BLOCK_ON_RESPAGE=None;FORMAT_OBJECT=Alignment;FORMAT_TYPE=HTML;GAPCOSTS=11%201;I_THRESH=0.001;LAYOUT=TwoWindows;MATRIX_NAME=BLOSUM62;NCBI_GI=on;PAGE=Proteins;PROGRAM=blastp;QUERY=ASSTAPTLFPLAQCGSGTGDMMTLGCIATGFTPASLTFKWNEQGGNSLTDFVQYPAVQTSGSYMGVSQLRVKRADWDSKIFECAVEHSAGSKTVPVKKQAEYLQHPSLYVMTPSKEEMAENMTASFACFANDFSPRTHTIKWMRMEQGIEKEVVSDFKSSCESEKKSDKTLYSTTSYLRVNESEWKSEEVAFTCVFENKAGNVRRTVGYTSSDAGPVHAHSVVIKITPPSLEDMLMNKKAELVCDVEELVPGFMSVKWENDNGKTLTSRKGVTDRIAILDITYEDWSNGTVFYCAVDHLENLGSLVKKPYKRETGGDPQRPSVFLLAPAEKTSDNTVTLTCYVKDFYPKEVLVAWLIDDEPVERTSSSALYQFNTTSQIQTGRTYSVYSQLTFSNDLWKNKEVVYSCVVYHESMIKSTKILMRTIDRTSNQPYLVNLSLNVPQSCKAQ;SERVICE=plain;SET_DEFAULTS.x=9;SET_DEFAULTS.y=5;SHOW_OVERVIEW=on;WORD_SIZE=3;END_OF_HTTPGET=Yes).

**Search parameters**

| **MS data file:** | H:\MBA1-G6\67_BF7_01_1699.mgf |
| --- | --- |
| **Enzyme:** | Trypsin: cuts C-term side of KR unless next residue is P. |
| **Fixed modifications:** | [Carbamidomethyl (C)](http://fun-gen1.ibls.gla.ac.uk/mascot/cgi/client.pl?modification;file=..%2Fdata%2F20110930%2FF112320.dat;mod_name=Carbamidomethyl%20%28C%29) |
| **Variable modifications:** | [Oxidation (M)](http://fun-gen1.ibls.gla.ac.uk/mascot/cgi/client.pl?modification;file=..%2Fdata%2F20110930%2FF112320.dat;mod_name=Oxidation%20%28M%29) |

**Protein sequence coverage: 12%**

Matched peptides shown in ***bold red***.

| **1** | ASSTAPTLFP | LAQCGSGTGD | MMTLGCIATG | FTPASLTFKW | NEQGGNSLTD |
| --- | --- | --- | --- | --- | --- |
| **51** | FVQYPAVQTS | GSYMGVSQLR | VKRADWDSK**I** | **FECAVEHSAG** | **SK**TVPVKKQA |
| **101** | EYLQHPSLYV | MTPSKEEMAE | NMTASFACFA | NDFSPRTHTI | KWMRMEQGIE |
| **151** | KEVVSDFKSS | CESEKKSDK**T** | **LYSTTSYLR**V | NESEWKSEEV | AFTCVFENKA |
| **201** | GNVRRTVGYT | SSDAGPVHAH | SVVIKITPPS | LEDMLMNKKA | ELVCDVEELV |
| **251** | PGFMSVKWEN | DNGKTLTSRK | GVTDRIAILD | ITYEDWSNGT | VFYCAVDHLE |
| **301** | NLGSLVKKPY | KR**ETGGDPQR** | **PSVFLLAPAE** | **KTSDNTVTLT** | **CYVK**DFYPKE |
| **351** | VLVAWLIDDE | PVERTSSSAL | YQFNTTSQIQ | TGRTYSVYSQ | LTFSNDLWKN |
| **401** | KEVVYSCVVY | HESMIKSTKI | LMRTIDRTSN | QPYLVNLSLN | VPQSCKAQ |

**SPOT #613**

**Protein View: ABONS2**

**serum albumin 2 precursor - Atlantic salmon**

Top of Form

| **Database:** | MSDB |
| --- | --- |
| **Score:** | 488 |
| **Nominal mass (M_r_):** | 69124 |
| **Calculated pI:** | 5.44 |
| **Taxonomy:** | [Salmo salar](http://www.ncbi.nlm.nih.gov/Taxonomy/Browser/wwwtax.cgi?lvl=0&id=8030) |

This protein sequence matches the following other entries:

- CAA43187 from [Salmo salar](http://www.ncbi.nlm.nih.gov/Taxonomy/Browser/wwwtax.cgi?lvl=0&id=8030)
- ALBU2_SALSA from [Salmo salar](http://www.ncbi.nlm.nih.gov/Taxonomy/Browser/wwwtax.cgi?lvl=0&id=8030)

Sequence similarity is available as [an NCBI BLAST search of ABONS2 against nr](http://www.ncbi.nlm.nih.gov/blast/Blast.cgi?ALIGNMENTS=50;ALIGNMENT_VIEW=Pairwise;AUTO_FORMAT=Semiauto;CDD_SEARCH=on;CLIENT=web;COMPOSITION_BASED_STATISTICS=on;DATABASE=nr;DESCRIPTIONS=100;ENTREZ_QUERY=%28none%29;EXPECT=10;FILTER=L;FORMAT_BLOCK_ON_RESPAGE=None;FORMAT_OBJECT=Alignment;FORMAT_TYPE=HTML;GAPCOSTS=11%201;I_THRESH=0.001;LAYOUT=TwoWindows;MATRIX_NAME=BLOSUM62;NCBI_GI=on;PAGE=Proteins;PROGRAM=blastp;QUERY=MQWLSVCSLLVLLSVLSRSQAQNQICTIFTEAKEDGFKSLILVGLAQNLPDSTLGDLVPLIAEALAMGVKCCSDTPPEDCERDVADLFQSAVCSSETLVEKNDLKMCCEKTAAERTHCFVDHKAKIPRDLSLKAELPAADQCEDFKKDHKAFVGRFIFKFSKSNPMLPPHVVLAIAKGYGEVLTTCCGEAEAQTCFDTKKATFQHAIAKRVAELKSLCIVHKKYGDRVVKAKKLVQYSQKMPQASFQEMAGMVDKIVATVAPCCSGDMVTCMKERKTLVDEVCADESVLSRAAGLSACCKEDAVHRGSCVEAMKPDPKPDGLSEHYDVHADIAAVCQTFTKTPDVAMGKLVYEISVRHPESSQQVILRFAKEAEQALLQCCDMEDHAECVKTALAGSDIDKKITDETDYYKKMCAAEAAVSDDNFEKSMMVYYTRIMPQASFDQLHMVSETVHDVLHACCKDEPGHFVLPCAEEKLTDAIDATCDDYDPSSINPHIAHCCNQSYSMRRHCILAIQPDTEFTPPELDASSFHMGPELCTKDSKDLLLSGKKLLYGVVRHKTTITEDHLKTISTKYHTMKDKCCAAEDQAACFTEEAPKLVSESAELVKV;SERVICE=plain;SET_DEFAULTS.x=9;SET_DEFAULTS.y=5;SHOW_OVERVIEW=on;WORD_SIZE=3;END_OF_HTTPGET=Yes).

**Search parameters**

| **MS data file:** | H:\MBA1-G6\68_BF8_01_1700.mgf |
| --- | --- |
| **Enzyme:** | Trypsin: cuts C-term side of KR unless next residue is P. |
| **Fixed modifications:** | [Carbamidomethyl (C)](http://fun-gen1.ibls.gla.ac.uk/mascot/cgi/client.pl?modification;file=..%2Fdata%2F20110930%2FF112321.dat;mod_name=Carbamidomethyl%20%28C%29) |
| **Variable modifications:** | [Oxidation (M)](http://fun-gen1.ibls.gla.ac.uk/mascot/cgi/client.pl?modification;file=..%2Fdata%2F20110930%2FF112321.dat;mod_name=Oxidation%20%28M%29) |

**Protein sequence coverage: 23%**

Matched peptides shown in ***bold red***.

| **1** | MQWLSVCSLL | VLLSVLSRSQ | AQNQICTIFT | EAKEDGFKSL | ILVGLAQNLP |
| --- | --- | --- | --- | --- | --- |
| **51** | DSTLGDLVPL | IAEALAMGVK | CCSDTPPEDC | ERDVADLFQS | AVCSSETLVE |
| **101** | KNDLKMCCEK | TAAERTHCFV | DHKAKIPRDL | SLKAELPAAD | QCEDFKKDHK |
| **151** | AFVGRFIFKF | SKSNPMLPPH | VVLAIAKGYG | EVLTTCCGEA | EAQTCFDTKK |
| **201** | ATFQHAIAKR | VAELKSLCIV | HKKYGDRVVK | AKKLVQYSQK | **MPQASFQEMA** |
| **251** | **GMVDKIVATV** | **APCCSGDMVT** | **CMK**ER**KTLVD** | **EVCADESVLS** | **R**AAGLSACCK |
| **301** | EDAVHRGSCV | EAMKPDPKPD | GLSEHYDVHA | DIAAVCQTFT | KTPDVAMGKL |
| **351** | VYEISVR**HPE** | **SSQQVILR**FA | K**EAEQALLQC** | **CDMEDHAECV** | **K**TALAGSDID |
| **401** | K**KITDETDYY** | **KKMCAAEAAV** | **SDDNFEK**SMM | VYYTRIMPQA | SFDQLHMVSE |
| **451** | TVHDVLHACC | KDEPGHFVLP | CAEEKLTDAI | DATCDDYDPS | SINPHIAHCC |
| **501** | NQSYSMRRHC | ILAIQPDTEF | TPPELDASSF | HMGPELCTKD | SKDLLLSGKK |
| **551** | **LLYGVVR**HKT | TITEDHLKTI | STKYHTMKDK | **CCAAEDQAAC** | **FTEEAPKLVS** |
| **601** | **ESAELVKV** |  |  |  |  |

**SPOT #260**

**Protein View: ABONS2**

**serum albumin 2 precursor - Atlantic salmon**

Top of Form

| **Database:** | MSDB |
| --- | --- |
| **Score:** | 364 |
| **Nominal mass (M_r_):** | 69124 |
| **Calculated pI:** | 5.44 |
| **Taxonomy:** | [Salmo salar](http://www.ncbi.nlm.nih.gov/Taxonomy/Browser/wwwtax.cgi?lvl=0&id=8030) |

This protein sequence matches the following other entries:

- CAA43187 from [Salmo salar](http://www.ncbi.nlm.nih.gov/Taxonomy/Browser/wwwtax.cgi?lvl=0&id=8030)
- ALBU2_SALSA from [Salmo salar](http://www.ncbi.nlm.nih.gov/Taxonomy/Browser/wwwtax.cgi?lvl=0&id=8030)

Sequence similarity is available as [an NCBI BLAST search of ABONS2 against nr](http://www.ncbi.nlm.nih.gov/blast/Blast.cgi?ALIGNMENTS=50;ALIGNMENT_VIEW=Pairwise;AUTO_FORMAT=Semiauto;CDD_SEARCH=on;CLIENT=web;COMPOSITION_BASED_STATISTICS=on;DATABASE=nr;DESCRIPTIONS=100;ENTREZ_QUERY=%28none%29;EXPECT=10;FILTER=L;FORMAT_BLOCK_ON_RESPAGE=None;FORMAT_OBJECT=Alignment;FORMAT_TYPE=HTML;GAPCOSTS=11%201;I_THRESH=0.001;LAYOUT=TwoWindows;MATRIX_NAME=BLOSUM62;NCBI_GI=on;PAGE=Proteins;PROGRAM=blastp;QUERY=MQWLSVCSLLVLLSVLSRSQAQNQICTIFTEAKEDGFKSLILVGLAQNLPDSTLGDLVPLIAEALAMGVKCCSDTPPEDCERDVADLFQSAVCSSETLVEKNDLKMCCEKTAAERTHCFVDHKAKIPRDLSLKAELPAADQCEDFKKDHKAFVGRFIFKFSKSNPMLPPHVVLAIAKGYGEVLTTCCGEAEAQTCFDTKKATFQHAIAKRVAELKSLCIVHKKYGDRVVKAKKLVQYSQKMPQASFQEMAGMVDKIVATVAPCCSGDMVTCMKERKTLVDEVCADESVLSRAAGLSACCKEDAVHRGSCVEAMKPDPKPDGLSEHYDVHADIAAVCQTFTKTPDVAMGKLVYEISVRHPESSQQVILRFAKEAEQALLQCCDMEDHAECVKTALAGSDIDKKITDETDYYKKMCAAEAAVSDDNFEKSMMVYYTRIMPQASFDQLHMVSETVHDVLHACCKDEPGHFVLPCAEEKLTDAIDATCDDYDPSSINPHIAHCCNQSYSMRRHCILAIQPDTEFTPPELDASSFHMGPELCTKDSKDLLLSGKKLLYGVVRHKTTITEDHLKTISTKYHTMKDKCCAAEDQAACFTEEAPKLVSESAELVKV;SERVICE=plain;SET_DEFAULTS.x=9;SET_DEFAULTS.y=5;SHOW_OVERVIEW=on;WORD_SIZE=3;END_OF_HTTPGET=Yes).

**Search parameters**

| **MS data file:** | H:\MBA1-G6\69_BF9_01_1701.mgf |
| --- | --- |
| **Enzyme:** | Trypsin: cuts C-term side of KR unless next residue is P. |
| **Fixed modifications:** | [Carbamidomethyl (C)](http://fun-gen1.ibls.gla.ac.uk/mascot/cgi/client.pl?modification;file=..%2Fdata%2F20110930%2FF112322.dat;mod_name=Carbamidomethyl%20%28C%29) |
| **Variable modifications:** | [Oxidation (M)](http://fun-gen1.ibls.gla.ac.uk/mascot/cgi/client.pl?modification;file=..%2Fdata%2F20110930%2FF112322.dat;mod_name=Oxidation%20%28M%29) |

**Protein sequence coverage: 24%**

Matched peptides shown in ***bold red***.

| **1** | MQWLSVCSLL | VLLSVLSRSQ | AQNQICTIFT | EAKEDGFKSL | ILVGLAQNLP |
| --- | --- | --- | --- | --- | --- |
| **51** | DSTLGDLVPL | IAEALAMGVK | CCSDTPPEDC | ERDVADLFQS | AVCSSETLVE |
| **101** | KNDLKMCCEK | TAAERTHCFV | DHKAKIPRDL | SLKAELPAAD | QCEDFKKDHK |
| **151** | AFVGRFIFKF | SKSNPMLPPH | VVLAIAKGYG | EVLTTCCGEA | EAQTCFDTKK |
| **201** | ATFQHAIAKR | VAELKSLCIV | HKKYGDRVVK | AKKLVQYSQK | **MPQASFQEMA** |
| **251** | **GMVDKIVATV** | **APCCSGDMVT** | **CMK**ER**KTLVD** | **EVCADESVLS** | **R**AAGLSACCK |
| **301** | EDAVHRGSCV | EAMKPDPKPD | GLSEHYDVHA | DIAAVCQTFT | KTPDVAMGKL |
| **351** | VYEISVR**HPE** | **SSQQVILR**FA | K**EAEQALLQC** | **CDMEDHAECV** | **K**TALAGSDID |
| **401** | KK**ITDETDYY** | **KKMCAAEAAV** | **SDDNFEK**SMM | VYYTRIMPQA | SFDQLHMVSE |
| **451** | TVHDVLHACC | KDEPGHFVLP | CAEEKLTDAI | DATCDDYDPS | SINPHIAHCC |
| **501** | NQSYSMRRHC | ILAIQPDTEF | TPPELDASSF | HMGPELCTKD | SKDLLLSGKK |
| **551** | LLYGVVR**HKT** | **TITEDHLK**TI | STKYHTMK**DK** | **CCAAEDQAAC** | **FTEEAPKLVS** |
| **601** | **ESAELVKV** |  |  |  |  |

**Protein View: ABONS1**

**serum albumin 1 precursor - Atlantic salmon**

Top of Form

| **Database:** | MSDB |
| --- | --- |
| **Score:** | 320 |
| **Nominal mass (M_r_):** | 69216 |
| **Calculated pI:** | 5.44 |
| **Taxonomy:** | [Salmo salar](http://www.ncbi.nlm.nih.gov/Taxonomy/Browser/wwwtax.cgi?lvl=0&id=8030) |

This protein sequence matches the following other entries:

- CAA36643 from [Salmo salar](http://www.ncbi.nlm.nih.gov/Taxonomy/Browser/wwwtax.cgi?lvl=0&id=8030)
- ALBU1_SALSA from [Salmo salar](http://www.ncbi.nlm.nih.gov/Taxonomy/Browser/wwwtax.cgi?lvl=0&id=8030)

Sequence similarity is available as [an NCBI BLAST search of ABONS1 against nr](http://www.ncbi.nlm.nih.gov/blast/Blast.cgi?ALIGNMENTS=50;ALIGNMENT_VIEW=Pairwise;AUTO_FORMAT=Semiauto;CDD_SEARCH=on;CLIENT=web;COMPOSITION_BASED_STATISTICS=on;DATABASE=nr;DESCRIPTIONS=100;ENTREZ_QUERY=%28none%29;EXPECT=10;FILTER=L;FORMAT_BLOCK_ON_RESPAGE=None;FORMAT_OBJECT=Alignment;FORMAT_TYPE=HTML;GAPCOSTS=11%201;I_THRESH=0.001;LAYOUT=TwoWindows;MATRIX_NAME=BLOSUM62;NCBI_GI=on;PAGE=Proteins;PROGRAM=blastp;QUERY=MQWLSVCSLLVLLSVLSRSQAQNQICTIFTEAKEDGFKSLILVGLAQNLPDSTLGDLVPLIAEALAMGVKCCSDTPPEDCERDVADLFQSAVCSSETLVEKNDLKMCCEKTAAERTHCFVDHKAKIPRDLSLKAELPAADQCEDFKKDHKAFVGRFIFKFSKSNPMLPPHVVLAIAKGYGEVLTTCCGEAEAQTCFDTKKATFQHAVMKRVAELRSLCIVHKKYGDRVVKAKKLVQYSQKMPQASFQEMGGMVDKIVATVAPCCSGDMVTCMKERKTLVDEVCADESVLSRAAGLSACCKEDAVHRGSCVEAMKPDPKPDGLSEHYDIHADIAAVCQTFTKTPDVAMGKLVYEISVRHPESSQQVILRFAKEAEQALLQCCDMEDHAECVKTALAGSDIDKKITDETDYYKKMCAAEAAVSDDSFEKSMMVYYTRIMPQASFDQLHMVSETVHDVLHACCKDEQGHFVLPCAEEKLTDAIDATCDDYDPSSINPHIAHCCNQSYSMRRHCILAIQPDTEFTPPELDASSFHMGPELCTKDSKDLLLSGKKLLYGVVRHKTTITEDHLKTISTKYHTMKEKCCAAEDQAACFTEEAPKLVSESAELVKV;SERVICE=plain;SET_DEFAULTS.x=9;SET_DEFAULTS.y=5;SHOW_OVERVIEW=on;WORD_SIZE=3;END_OF_HTTPGET=Yes).

**Search parameters**

| **MS data file:** | H:\MBA1-G6\69_BF9_01_1701.mgf |
| --- | --- |
| **Enzyme:** | Trypsin: cuts C-term side of KR unless next residue is P. |
| **Fixed modifications:** | [Carbamidomethyl (C)](http://fun-gen1.ibls.gla.ac.uk/mascot/cgi/client.pl?modification;file=..%2Fdata%2F20110930%2FF112322.dat;mod_name=Carbamidomethyl%20%28C%29) |
| **Variable modifications:** | [Oxidation (M)](http://fun-gen1.ibls.gla.ac.uk/mascot/cgi/client.pl?modification;file=..%2Fdata%2F20110930%2FF112322.dat;mod_name=Oxidation%20%28M%29) |

**Protein sequence coverage: 20%**

Matched peptides shown in ***bold red***.

| **1** | MQWLSVCSLL | VLLSVLSRSQ | AQNQICTIFT | EAKEDGFKSL | ILVGLAQNLP |
| --- | --- | --- | --- | --- | --- |
| **51** | DSTLGDLVPL | IAEALAMGVK | CCSDTPPEDC | ERDVADLFQS | AVCSSETLVE |
| **101** | KNDLKMCCEK | TAAERTHCFV | DHKAKIPRDL | SLKAELPAAD | QCEDFKKDHK |
| **151** | AFVGRFIFKF | SKSNPMLPPH | VVLAIAKGYG | EVLTTCCGEA | EAQTCFDTKK |
| **201** | ATFQHAVMKR | VAELRSLCIV | HKKYGDRVVK | AKKLVQYSQK | **MPQASFQEMG** |
| **251** | **GMVDKIVATV** | **APCCSGDMVT** | **CMK**ER**KTLVD** | **EVCADESVLS** | **R**AAGLSACCK |
| **301** | EDAVHRGSCV | EAMKPDPKPD | GLSEHYDIHA | DIAAVCQTFT | KTPDVAMGKL |
| **351** | VYEISVR**HPE** | **SSQQVILR**FA | K**EAEQALLQC** | **CDMEDHAECV** | **K**TALAGSDID |
| **401** | KK**ITDETDYY** | **KK**MCAAEAAV | SDDSFEKSMM | VYYTRIMPQA | SFDQLHMVSE |
| **451** | TVHDVLHACC | K**DEQGHFVLP** | **CAEEK**LTDAI | DATCDDYDPS | SINPHIAHCC |
| **501** | NQSYSMRRHC | ILAIQPDTEF | TPPELDASSF | HMGPELCTKD | SKDLLLSGKK |
| **551** | LLYGVVR**HKT** | **TITEDHLK**TI | STKYHTMKEK | CCAAEDQAAC | FTEEAPK**LVS** |
| **601** | **ESAELVKV** |  |  |  |  |

**SPOT #598**

**Protein View: JH0472**

**apolipoprotein A-I precursor - Atlantic salmon**

Top of Form

| **Database:** | MSDB |
| --- | --- |
| **Score:** | 257 |
| **Nominal mass (M_r_):** | 29557 |
| **Calculated pI:** | 8.48 |
| **Taxonomy:** | [Salmo salar](http://www.ncbi.nlm.nih.gov/Taxonomy/Browser/wwwtax.cgi?lvl=0&id=8030) |

Sequence similarity is available as [an NCBI BLAST search of JH0472 against nr](http://www.ncbi.nlm.nih.gov/blast/Blast.cgi?ALIGNMENTS=50;ALIGNMENT_VIEW=Pairwise;AUTO_FORMAT=Semiauto;CDD_SEARCH=on;CLIENT=web;COMPOSITION_BASED_STATISTICS=on;DATABASE=nr;DESCRIPTIONS=100;ENTREZ_QUERY=%28none%29;EXPECT=10;FILTER=L;FORMAT_BLOCK_ON_RESPAGE=None;FORMAT_OBJECT=Alignment;FORMAT_TYPE=HTML;GAPCOSTS=11%201;I_THRESH=0.001;LAYOUT=TwoWindows;MATRIX_NAME=BLOSUM62;NCBI_GI=on;PAGE=Proteins;PROGRAM=blastp;QUERY=MKFLVLALTILLAAGTQAFPMQADAPSQLEHVKAALNMYIAQVKLTAQRSIDLLDDTEYKEYKMQLSQSLDNLQQFADSTSKSWPPTPRSSAPSCDATATVRAEVMKDVEDVRTQLEPKRAELREVLNKHIDEYRKKLEPLIKEHIELRRTEMDAFRAKIEPVVEEMRAKVAVNVEETKTKLMPIVEIVRAKLTERLEELRTLAAPYAEEYKEQMFKAVGEVREKVAPLSEDFKARWAPPPRRPSKSSWLSTRPSARP;SERVICE=plain;SET_DEFAULTS.x=9;SET_DEFAULTS.y=5;SHOW_OVERVIEW=on;WORD_SIZE=3;END_OF_HTTPGET=Yes).

**Search parameters**

| **MS data file:** | H:\MBA1-G6\70_BF10_01_1702.mgf |
| --- | --- |
| **Enzyme:** | Trypsin: cuts C-term side of KR unless next residue is P. |
| **Fixed modifications:** | [Carbamidomethyl (C)](http://fun-gen1.ibls.gla.ac.uk/mascot/cgi/client.pl?modification;file=..%2Fdata%2F20110930%2FF112323.dat;mod_name=Carbamidomethyl%20%28C%29) |
| **Variable modifications:** | [Oxidation (M)](http://fun-gen1.ibls.gla.ac.uk/mascot/cgi/client.pl?modification;file=..%2Fdata%2F20110930%2FF112323.dat;mod_name=Oxidation%20%28M%29) |

**Protein sequence coverage: 42%**

Matched peptides shown in ***bold red***.

| **1** | MKFLVLALTI | LLAAGTQAFP | MQADAPSQLE | HVK**AALNMYI** | **AQVK**LTAQR**S** |
| --- | --- | --- | --- | --- | --- |
| **51** | **IDLLDDTEYK** | **EYKMQLSQSL** | **DNLQQFADST** | **SK**SWPPTPRS | SAPSCDATAT |
| **101** | VRAEVMKDVE | DVRTQLEPKR | AELR**EVLNKH** | **IDEYR**K**KLEP** | **LIK**EHIELRR |
| **151** | TEMDAFR**AKI** | **EPVVEEMR**AK | VAVNVEETK**T** | **KLMPIVEIVR** | AK**LTERLEEL** |
| **201** | **RTLAAPYAEE** | **YKEQMFK**AVG | EVREKVAPLS | EDFKARWAPP | PRRPSKSSWL |
| **251** | STRPSARP |  |  |  |  |

**SPOT #227**

**Protein View: P79825_ONCMY**

**Hemopexin-like protein (Fragment).- Oncorhynchus mykiss (Rainbow trout) (Salmo gairdneri).**

Top of Form

| **Database:** | MSDB |
| --- | --- |
| **Score:** | 128 |
| **Nominal mass (M_r_):** | 51106 |
| **Calculated pI:** | 5.61 |
| **Taxonomy:** | [Oncorhynchus mykiss](http://www.ncbi.nlm.nih.gov/Taxonomy/Browser/wwwtax.cgi?lvl=0&id=8022) |

This protein sequence matches the following other entries:

- CAA92147 from [Oncorhynchus mykiss](http://www.ncbi.nlm.nih.gov/Taxonomy/Browser/wwwtax.cgi?lvl=0&id=8022)

Sequence similarity is available as [an NCBI BLAST search of P79825_ONCMY against nr](http://www.ncbi.nlm.nih.gov/blast/Blast.cgi?ALIGNMENTS=50;ALIGNMENT_VIEW=Pairwise;AUTO_FORMAT=Semiauto;CDD_SEARCH=on;CLIENT=web;COMPOSITION_BASED_STATISTICS=on;DATABASE=nr;DESCRIPTIONS=100;ENTREZ_QUERY=%28none%29;EXPECT=10;FILTER=L;FORMAT_BLOCK_ON_RESPAGE=None;FORMAT_OBJECT=Alignment;FORMAT_TYPE=HTML;GAPCOSTS=11%201;I_THRESH=0.001;LAYOUT=TwoWindows;MATRIX_NAME=BLOSUM62;NCBI_GI=on;PAGE=Proteins;PROGRAM=blastp;QUERY=TMKPLSQTLCLCLVLALSHAHHHAGHQGGEDEGHEGHDHGHHEGLLLDRCQGIEMDAVAVTEEGIPYFFKGGHVFKGFHGKAELSNESFAELDDHHHLGHVDAAFLMHFPDKPTEHDHIFFMLDTKVFSYYKHQLETGFPKDISEVFPGIPDHLDAAVVCPAPDCEEDAVIFFKGDEIYHYNVKTKKVEEKKFEGMPNCTSAFRFMEHYYCFHGHQFSKFDPKTGEVHGRYPKEARDYFMKCSKFGDTTDHIERERCSRVHLDAITSDDAGNIYAFRGHHFLEQDAGNDTWAADTIESDFKELHSEVDATFSYENHLYMVKDDKVYIYKVGDSHTHLDGSPKPLKEVLGVEGPIDAAFVCQDHHIAHVIKGQTVYDVDLKASPPVPVKEGSFTLFNKVDAAMCGPEGVKLFKGNHYFHFQSVKVMLMAKAIPEEHKTALELFGCDH;SERVICE=plain;SET_DEFAULTS.x=9;SET_DEFAULTS.y=5;SHOW_OVERVIEW=on;WORD_SIZE=3;END_OF_HTTPGET=Yes).

**Search parameters**

| **MS data file:** | H:\MBA1-G6\71_BF11_01_1703.mgf |
| --- | --- |
| **Enzyme:** | Trypsin: cuts C-term side of KR unless next residue is P. |
| **Fixed modifications:** | [Carbamidomethyl (C)](http://fun-gen1.ibls.gla.ac.uk/mascot/cgi/client.pl?modification;file=..%2Fdata%2F20110930%2FF112324.dat;mod_name=Carbamidomethyl%20%28C%29) |
| **Variable modifications:** | [Oxidation (M)](http://fun-gen1.ibls.gla.ac.uk/mascot/cgi/client.pl?modification;file=..%2Fdata%2F20110930%2FF112324.dat;mod_name=Oxidation%20%28M%29) |

**Protein sequence coverage: 14%**

Matched peptides shown in ***bold red***.

| **1** | TMKPLSQTLC | LCLVLALSHA | HHHAGHQGGE | DEGHEGHDHG | HHEGLLLDRC |
| --- | --- | --- | --- | --- | --- |
| **51** | QGIEMDAVAV | TEEGIPYFFK | GGHVFKGFHG | KAELSNESFA | ELDDHHHLGH |
| **101** | VDAAFLMHFP | DKPTEHDHIF | FMLDTK**VFSY** | **YK**HQLETGFP | KDISEVFPGI |
| **151** | PDHLDAAVVC | PAPDCEEDAV | IFFK**GDEIYH** | **YNVK**TKKVEE | KKFEGMPNCT |
| **201** | SAFRFMEHYY | CFHGHQFSKF | DPKTGEVHGR | YPKEARDYFM | KCSK**FGDTTD** |
| **251** | **HIERER**CSR**V** | **HLDAITSDDA** | **GNIYAFR**GHH | FLEQDAGNDT | WAADTIESDF |
| **301** | KELHSEVDAT | FSYENHLYMV | KDDKVYIYKV | GDSHTHLDGS | PKPLKEVLGV |
| **351** | EGPIDAAFVC | QDHHIAHVIK | **GQTVYDVDLK** | ASPPVPVKEG | SFTLFNKVDA |
| **401** | AMCGPEGVKL | FKGNHYFHFQ | SVKVMLMAKA | IPEEHK**TALE** | **LFGCDH** |

**SPOT #381**

**Protein View: Q9DDG6_SALTR**

**Alpha-2 enolase-1 (Fragment).- Salmo trutta (Brown trout).**

Top of Form

| **Database:** | MSDB |
| --- | --- |
| **Score:** | 704 |
| **Nominal mass (M_r_):** | 39667 |
| **Calculated pI:** | 5.78 |
| **Taxonomy:** | [Salmo trutta](http://www.ncbi.nlm.nih.gov/Taxonomy/Browser/wwwtax.cgi?lvl=0&id=8032) |

This protein sequence matches the following other entries:

- AAG16311 from [Salmo trutta](http://www.ncbi.nlm.nih.gov/Taxonomy/Browser/wwwtax.cgi?lvl=0&id=8032)

Sequence similarity is available as [an NCBI BLAST search of Q9DDG6_SALTR against nr](http://www.ncbi.nlm.nih.gov/blast/Blast.cgi?ALIGNMENTS=50;ALIGNMENT_VIEW=Pairwise;AUTO_FORMAT=Semiauto;CDD_SEARCH=on;CLIENT=web;COMPOSITION_BASED_STATISTICS=on;DATABASE=nr;DESCRIPTIONS=100;ENTREZ_QUERY=%28none%29;EXPECT=10;FILTER=L;FORMAT_BLOCK_ON_RESPAGE=None;FORMAT_OBJECT=Alignment;FORMAT_TYPE=HTML;GAPCOSTS=11%201;I_THRESH=0.001;LAYOUT=TwoWindows;MATRIX_NAME=BLOSUM62;NCBI_GI=on;PAGE=Proteins;PROGRAM=blastp;QUERY=TKKGLFRAAVPSGASTGIYEALELRDNDKTRYLGKGVKRAVKHINEFLAPALCNQNVNVLEQEKVDKLMLDMDGTENKSKFGANAILGVSLAVCKAGAAEKGVPLYRHIADLAGNPNXILPCPAFNVINGGSHAGNKLAMQEFMILPIGASNFHEAMRIGAEVYHNLKNVIKAKYGKDATNVGDEGGFAPNILENNEALELLKSAIEKAGYPDKIIIGMDVAASEFYKAGKYDLDFKSPDDPARYITXDQLGDLYKSFIKGYPVQSIEDPFDQDDWAAWSKFTAAVDIQVVGDDLTVTNPKRIQQAVEKKACNCLLLKVNQIGSVTESIKACKLAQSNGWGVMVSHRSGETEDTFIADLVVGL;SERVICE=plain;SET_DEFAULTS.x=9;SET_DEFAULTS.y=5;SHOW_OVERVIEW=on;WORD_SIZE=3;END_OF_HTTPGET=Yes).

**Search parameters**

| **MS data file:** | H:\MBA1-G6\72_BF12_01_1704.mgf |
| --- | --- |
| **Enzyme:** | Trypsin: cuts C-term side of KR unless next residue is P. |
| **Fixed modifications:** | [Carbamidomethyl (C)](http://fun-gen1.ibls.gla.ac.uk/mascot/cgi/client.pl?modification;file=..%2Fdata%2F20110930%2FF112325.dat;mod_name=Carbamidomethyl%20%28C%29) |
| **Variable modifications:** | [Oxidation (M)](http://fun-gen1.ibls.gla.ac.uk/mascot/cgi/client.pl?modification;file=..%2Fdata%2F20110930%2FF112325.dat;mod_name=Oxidation%20%28M%29) |

**Protein sequence coverage: 28%**

Matched peptides shown in ***bold red***.

| **1** | TKKGLFRAAV | PSGASTGIYE | ALELRDNDKT | RYLGKGVKRA | VKHINEFLAP |
| --- | --- | --- | --- | --- | --- |
| **51** | ALCNQNVNVL | EQEKVDKLML | DMDGTENKSK | FGANAILGVS | LAVCKAGAAE |
| **101** | KGVPLYRHIA | DLAGNPNXIL | PCPAFNVING | GSHAGNK**LAM** | **QEFMILPIGA** |
| **151** | **SNFHEAMRIG** | **AEVYHNLK**NV | IKAKYGK**DAT** | **NVGDEGGFAP** | **NILENNEALE** |
| **201** | **LLK**SAIEKAG | YPDK**IIIGMD** | **VAASEFYK**AG | KYDLDFKSPD | DPAR**YITXDQ** |
| **251** | **LGDLYK**SFIK | GYPVQSIEDP | FDQDDWAAWS | KFTAAVDIQV | VGDDLTVTNP |
| **301** | KRIQQAVEK**K** | **ACNCLLLKVN** | **QIGSVTESIK** | ACKLAQSNGW | GVMVSHRSGE |
| **351** | TEDTFIADLV | VGL |  |  |  |

**Protein View: Q8JH38_OREMO**

**Muscle-type creatine kinase CKM2.- Oreochromis mossambicus (Mozambique tilapia) (Tilapia mossambica).**

Top of Form

| **Database:** | MSDB |
| --- | --- |
| **Score:** | 101 |
| **Nominal mass (M_r_):** | 42980 |
| **Calculated pI:** | 6.44 |
| **Taxonomy:** | [Oreochromis mossambicus](http://www.ncbi.nlm.nih.gov/Taxonomy/Browser/wwwtax.cgi?lvl=0&id=8127) |

This protein sequence matches the following other entries:

- AAK56448 from [Oreochromis mossambicus](http://www.ncbi.nlm.nih.gov/Taxonomy/Browser/wwwtax.cgi?lvl=0&id=8127)

Sequence similarity is available as [an NCBI BLAST search of Q8JH38_OREMO against nr](http://www.ncbi.nlm.nih.gov/blast/Blast.cgi?ALIGNMENTS=50;ALIGNMENT_VIEW=Pairwise;AUTO_FORMAT=Semiauto;CDD_SEARCH=on;CLIENT=web;COMPOSITION_BASED_STATISTICS=on;DATABASE=nr;DESCRIPTIONS=100;ENTREZ_QUERY=%28none%29;EXPECT=10;FILTER=L;FORMAT_BLOCK_ON_RESPAGE=None;FORMAT_OBJECT=Alignment;FORMAT_TYPE=HTML;GAPCOSTS=11%201;I_THRESH=0.001;LAYOUT=TwoWindows;MATRIX_NAME=BLOSUM62;NCBI_GI=on;PAGE=Proteins;PROGRAM=blastp;QUERY=MTKNCHNDYKMKFSVDDEFPDLTKHNNHMAKVLTKEIYAKLRSKSTPSGFTVDDVTQTGVDNPGHPFIMTGGCVAGDEESYEVFKDLLDPVISNRHGGYKPTDKHKTDLNFENLKGGDDLDPNYVLSSRVRTGRSIKGFTLPPHNSRGERRAIQNLSIEALSSLEGEFKGKYYPLDGMTDAEQEQLIADHFLFDKPVSPLLTCAGMARDWPDARGIWHNDNKTFLVWVNEEDHLRVISMQKGGNMKEVFRRFCVGLQKIEEIFKKHNHGFMWNEHLGYILTCPSNLGTGLRGGVHVKLPKLSTHPKFEEILTRLRLQKRGTGGVDTASVGGVFDISNADRLGSFEVEQVQLVVDGVKLMVEMEKKLEKGESIDGMIPAQK;SERVICE=plain;SET_DEFAULTS.x=9;SET_DEFAULTS.y=5;SHOW_OVERVIEW=on;WORD_SIZE=3;END_OF_HTTPGET=Yes).

**Search parameters**

| **MS data file:** | H:\MBA1-G6\72_BF12_01_1704.mgf |
| --- | --- |
| **Enzyme:** | Trypsin: cuts C-term side of KR unless next residue is P. |
| **Fixed modifications:** | [Carbamidomethyl (C)](http://fun-gen1.ibls.gla.ac.uk/mascot/cgi/client.pl?modification;file=..%2Fdata%2F20110930%2FF112325.dat;mod_name=Carbamidomethyl%20%28C%29) |
| **Variable modifications:** | [Oxidation (M)](http://fun-gen1.ibls.gla.ac.uk/mascot/cgi/client.pl?modification;file=..%2Fdata%2F20110930%2FF112325.dat;mod_name=Oxidation%20%28M%29) |

**Protein sequence coverage: 14%**

Matched peptides shown in ***bold red***.

| **1** | MTKNCHNDYK | MKFSVDDEFP | DLTKHNNHMA | KVLTKEIYAK | LRSKSTPSGF |
| --- | --- | --- | --- | --- | --- |
| **51** | TVDDVTQTGV | DNPGHPFIMT | GGCVAGDEES | YEVFKDLLDP | VISNRHGGYK |
| **101** | PTDKHKTDLN | FENLK**GGDDL** | **DPNYVLSSR**V | RTGRSIKGFT | LPPHNSRGER |
| **151** | RAIQNLSIEA | LSSLEGEFKG | KYYPLDGMTD | AEQEQLIADH | FLFDKPVSPL |
| **201** | LTCAGMARDW | PDARGIWHND | NKTFLVWVNE | EDHLRVISMQ | KGGNMKEVFR |
| **251** | RFCVGLQKIE | EIFKKHNHGF | MWNEHLGYIL | TCPSNLGTGL | RGGVHVKLPK |
| **301** | LSTHPK**FEEI** | **LTR**LRLQKR**G** | **TGGVDTASVG** | **GVFDISNADR** | LGSFEVEQVQ |
| **351** | LVVDGVKLMV | EMEKKLEK**GE** | **SIDGMIPAQK** |  |  |

Bottom of Form

Bottom of Form

Bottom of Form

Bottom of Form

Bottom of Form

Bottom of Form

Bottom of Form

Bottom of Form

Bottom of Form

Bottom of Form
